# Correction: mTORC1/S6K1 signaling promotes sustained oncogenic translation through modulating CRL3^IBTK^-mediated ubiquitination of eIF4A1 in cancer cells

**DOI:** 10.7554/eLife.112313

**Published:** 2026-06-17

**Authors:** Dongyue Jiao, Huiru Sun, Xiaying Zhao, Yingji Chen, Zeheng Lv, Qing Shi, Yao Li, Chenji Wang, Kun Gao

**Keywords:** Human

Jiao D, Sun H, Zhao X, Chen Y, Lv Z, Shi Q, Li Y, Wang C, Gao K. 2024. mTORC1/S6K1 signaling promotes sustained oncogenic translation through modulating CRL3IBTK-mediated ubiquitination of eIF4A1 in cancer cells. *eLife* **12**:RP92236. doi: 10.7554/eLife.92236.

Published 13 May 2024

During a recent self-review of our article, we discovered that representative panels in Figure 4F for the cell invasion and migration assays were inadvertently duplicated.

This error originated from our figure preparation and assembly process. Specifically, during the execution of these assays, multiple microscopic fields of view were captured for each experimental well to ensure representativeness. Subsequently, during figure assembly in Adobe Illustrator, raw images from all three experimental groups were imported simultaneously into the workspace to select representative panels. Because these raw image files were visually similar and had not been pre-sorted by experimental condition, this parallel selection process inadvertently led to a layout mix-up. Consequently, representative images for several experimental groups were inadvertently mixed up during the final layout assembly. Detailed documentation cross-referencing the affected panels and their original raw data sources were provided for the editors’ consideration. Due to an oversight, this visual selection mix-up was not caught during our final review.

These errors affect only the specific representative images in panel 4 F noted below. They do not alter the figure captions, any data quantifications, or the main conclusions of the article. We apologize for this oversight and any confusion it may have caused.

The corrected Figure 4 (updated for panel 4 F) is shown here:

**Figure 1:**
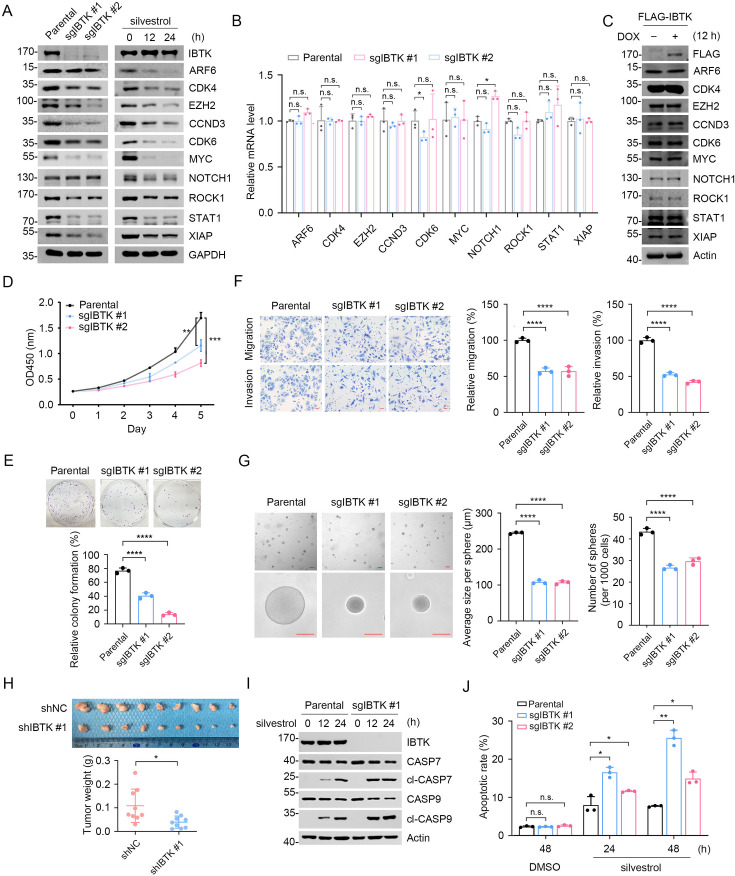


The originally published Figure 4 is shown for reference:

**Figure 2:**
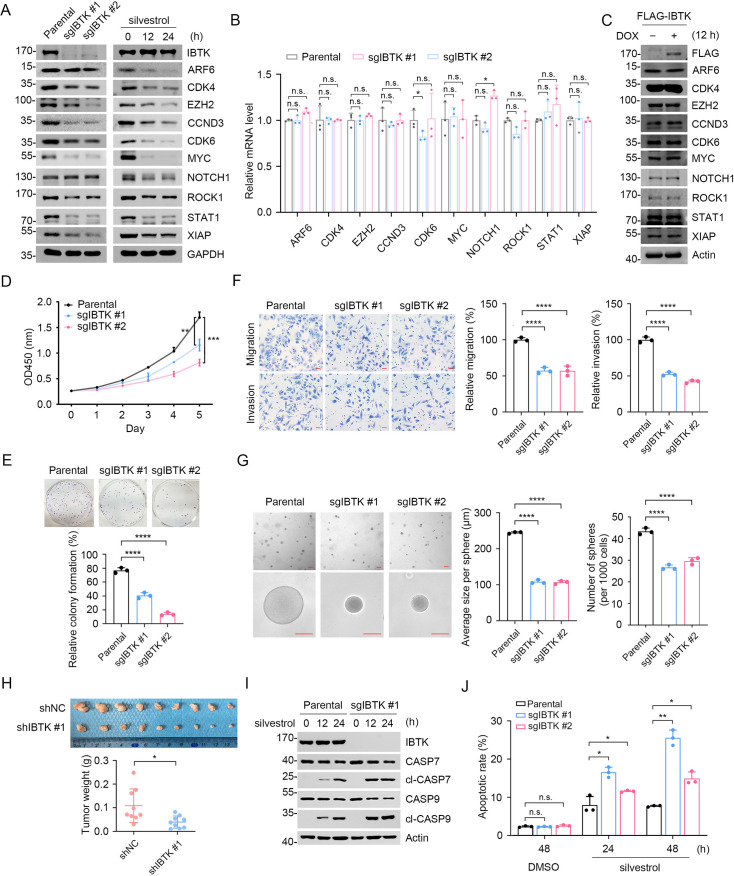


The article has been corrected accordingly.

