# Supplementary figures and images for: mTORC1/S6K1 signaling promotes sustained oncogenic translation through modulating CRL3IBTK-mediated ubiquitination of eIF4A1 in cancer cells (part 1 of 3)
Source: eLife. 2024 May 13;12:RP92236. doi: 10.7554/eLife.92236 (PMC11090508; doi:10.7554/eLife.92236)

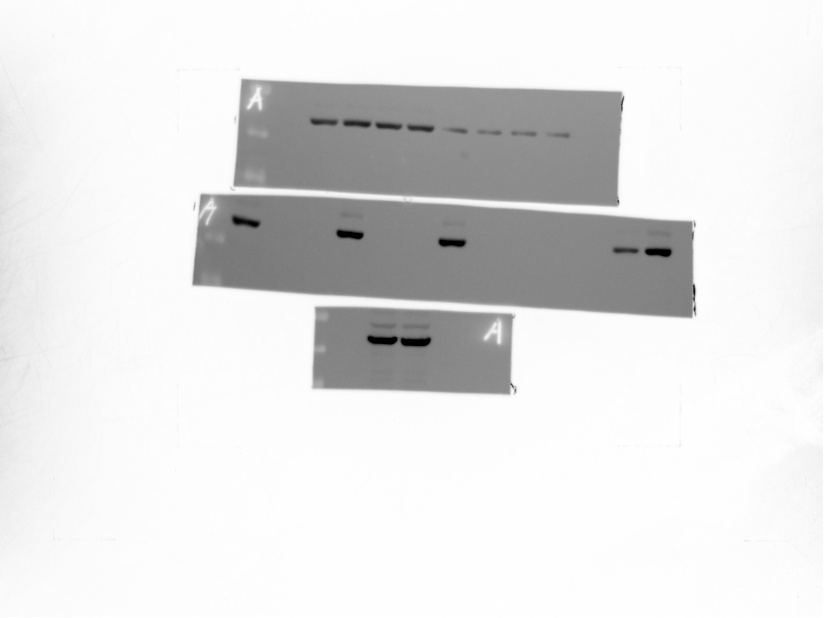

Supplement: Figure 1—source data 1. [file elife-92236-fig1-data1.zip › Figure_1-source_data_1/Figure_1-source_data_1_ Figure_1A_Actin.jpg]

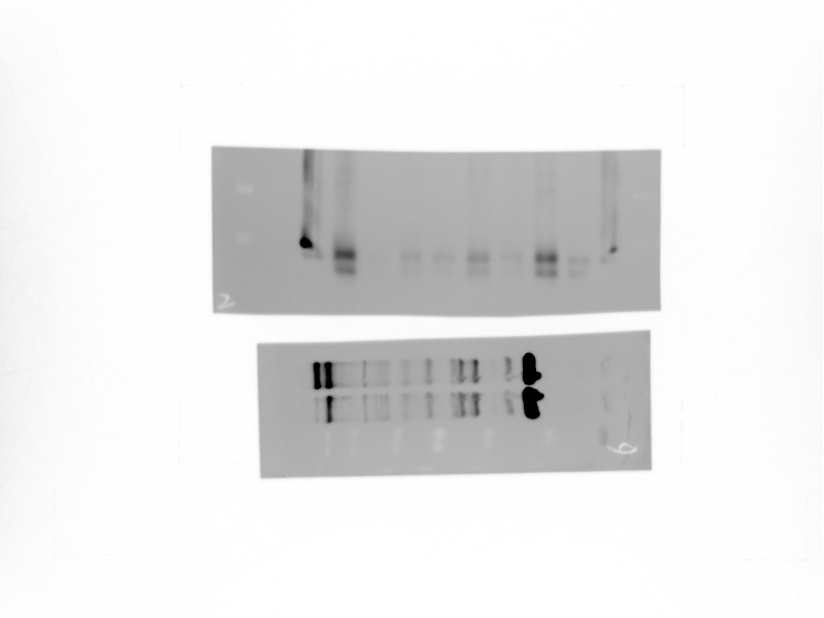

Supplement: Figure 1—source data 1. [file elife-92236-fig1-data1.zip › Figure_1-source_data_1/Figure_1-source_data_1_ Figure_1A_IBTK.jpg]

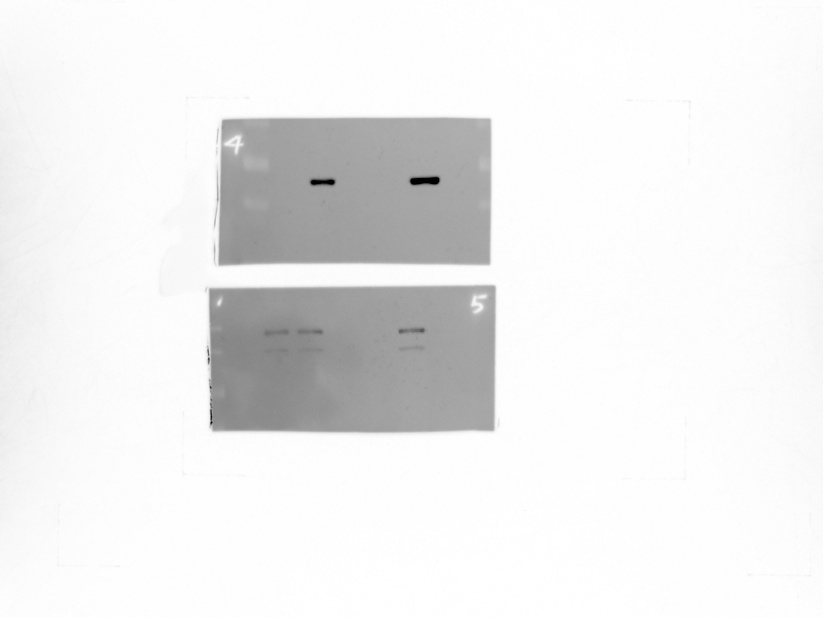

Supplement: Figure 1—source data 1. [file elife-92236-fig1-data1.zip › Figure_1-source_data_1/Figure_1-source_data_1_ Figure_1D_FLAG.jpg]

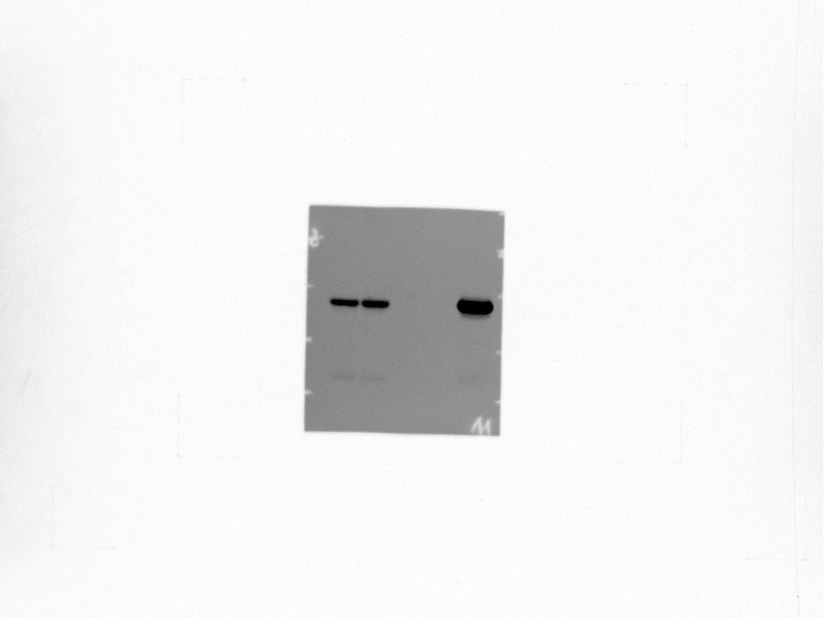

Supplement: Figure 1—source data 1. [file elife-92236-fig1-data1.zip › Figure_1-source_data_1/Figure_1-source_data_1_ Figure_1D_Myc.jpg]

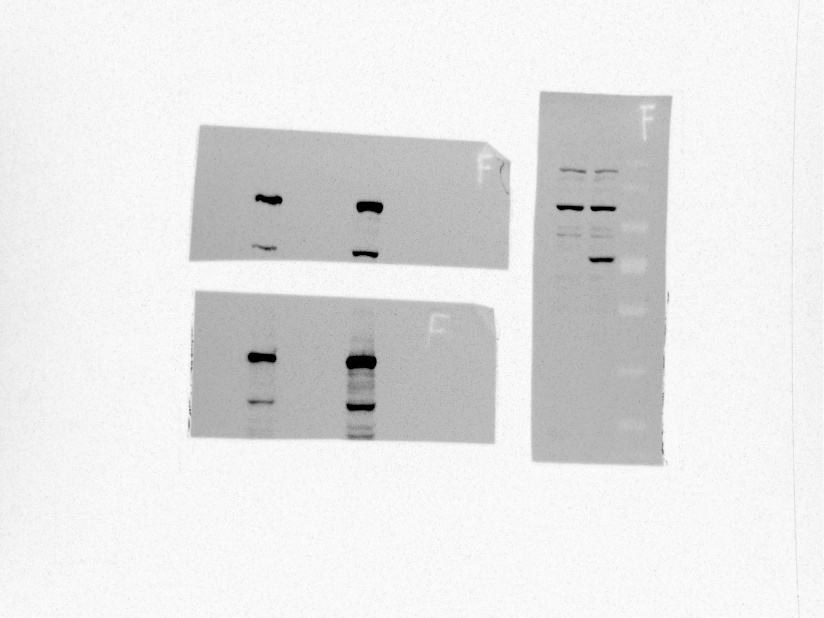

Supplement: Figure 1—source data 1. [file elife-92236-fig1-data1.zip › Figure_1-source_data_1/Figure_1-source_data_1_ Figure_1E_FLAG.jpg]

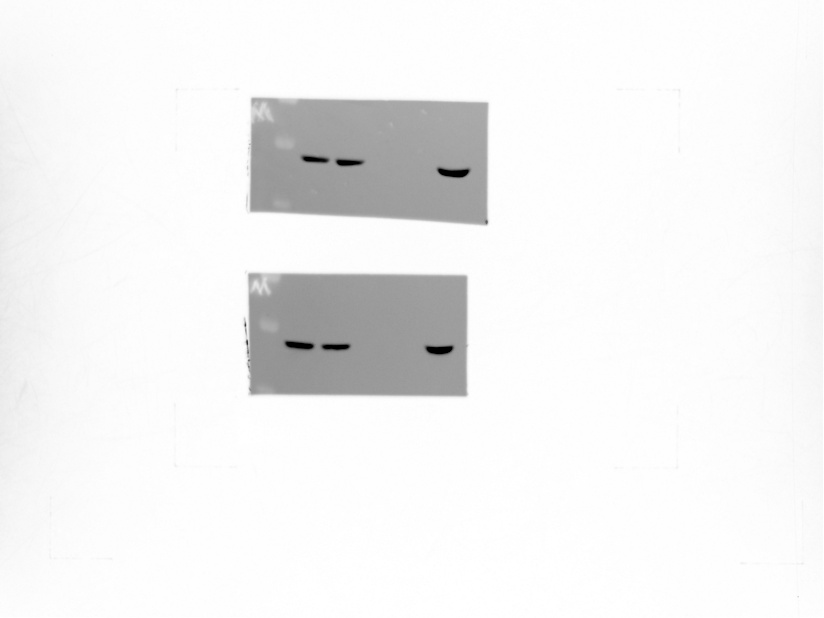

Supplement: Figure 1—source data 1. [file elife-92236-fig1-data1.zip › Figure_1-source_data_1/Figure_1-source_data_1_ Figure_1E_Myc.jpg]

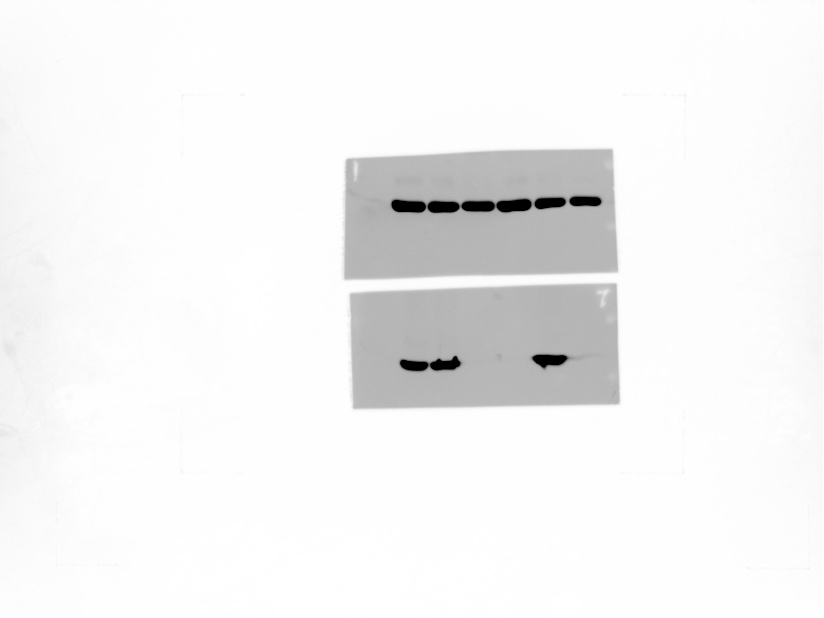

Supplement: Figure 1—source data 1. [file elife-92236-fig1-data1.zip › Figure_1-source_data_1/Figure_1-source_data_1_ Figure_1G_CUL3.jpg]

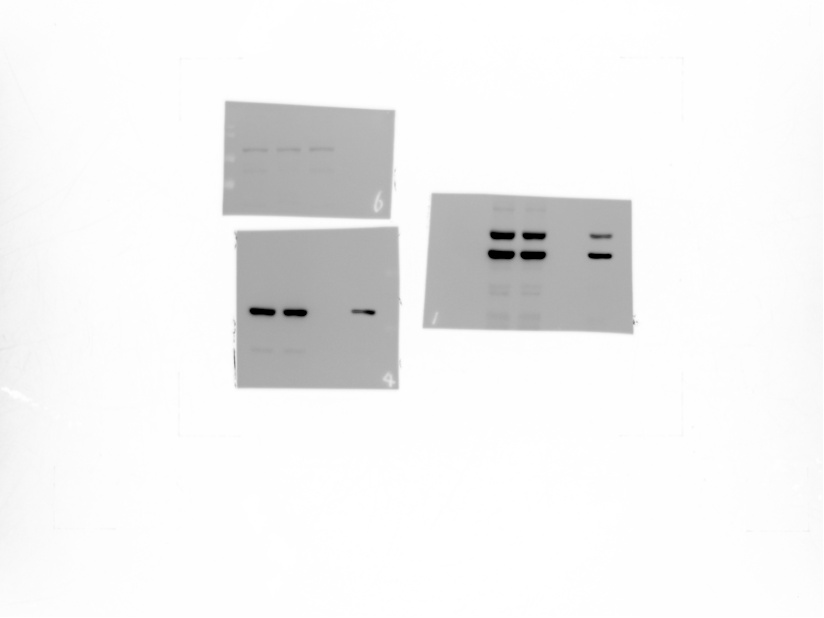

Supplement: Figure 1—source data 1. [file elife-92236-fig1-data1.zip › Figure_1-source_data_1/Figure_1-source_data_1_ Figure_1G_eIF2a┴.jpg]

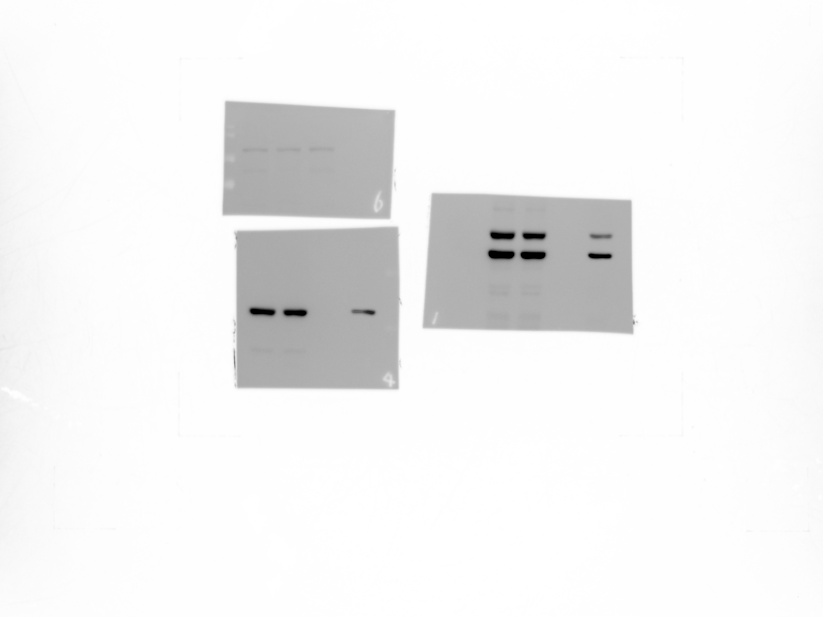

Supplement: Figure 1—source data 1. [file elife-92236-fig1-data1.zip › Figure_1-source_data_1/Figure_1-source_data_1_ Figure_1G_eIF3E.jpg]

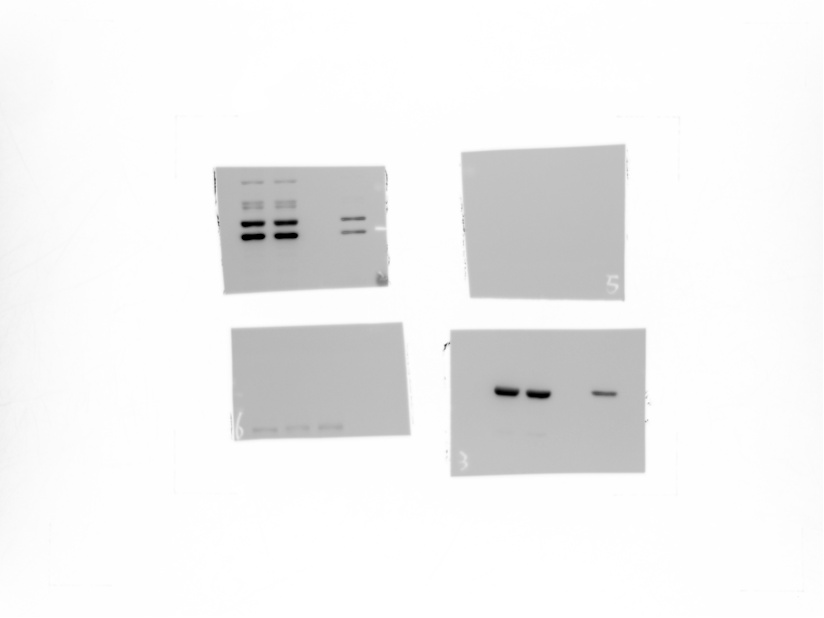

Supplement: Figure 1—source data 1. [file elife-92236-fig1-data1.zip › Figure_1-source_data_1/Figure_1-source_data_1_ Figure_1G_eIF3H.jpg]

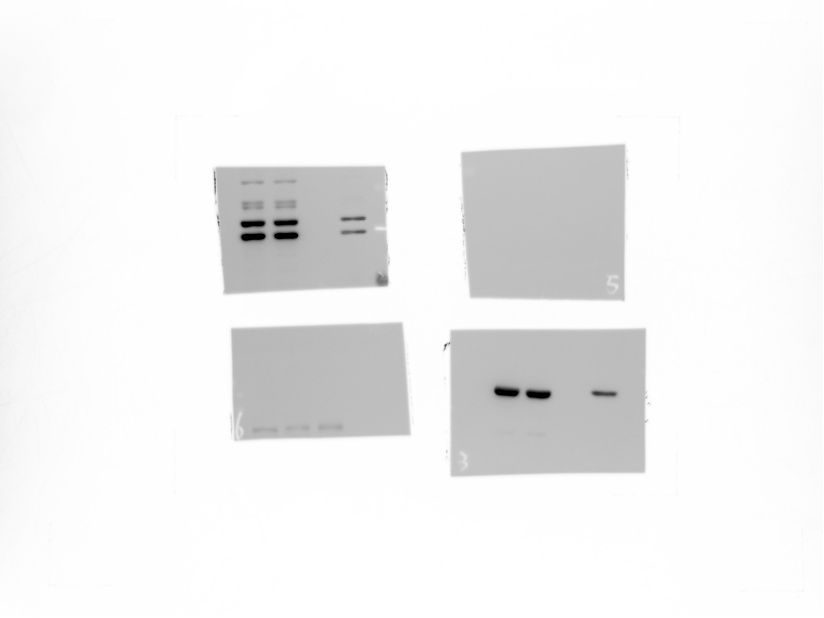

Supplement: Figure 1—source data 1. [file elife-92236-fig1-data1.zip › Figure_1-source_data_1/Figure_1-source_data_1_ Figure_1G_eIF3I.jpg]

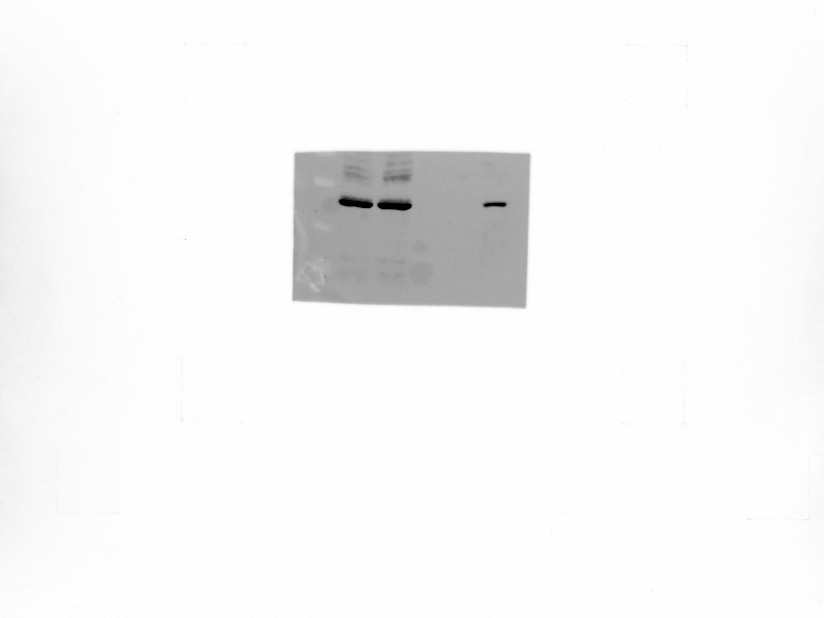

Supplement: Figure 1—source data 1. [file elife-92236-fig1-data1.zip › Figure_1-source_data_1/Figure_1-source_data_1_ Figure_1G_eIF4A1.jpg]

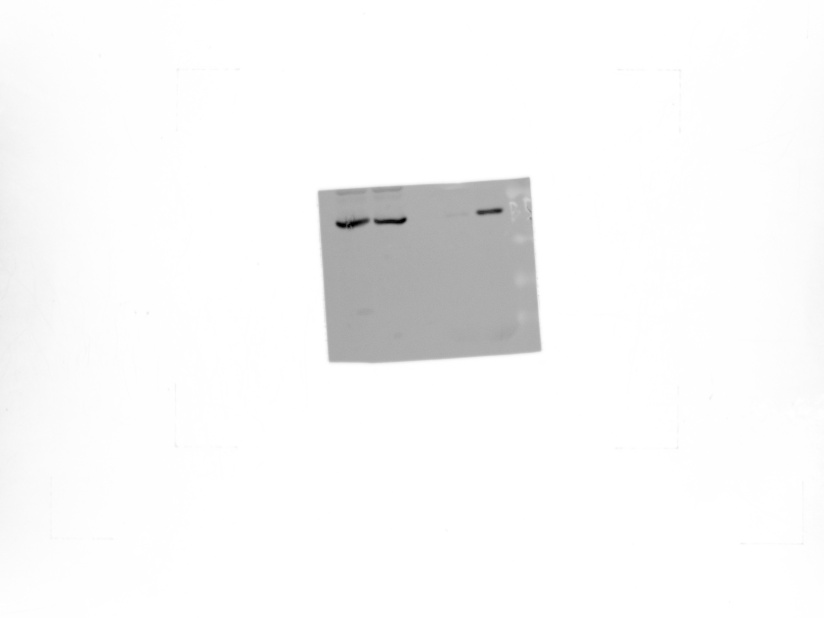

Supplement: Figure 1—source data 1. [file elife-92236-fig1-data1.zip › Figure_1-source_data_1/Figure_1-source_data_1_ Figure_1G_eIF4A2.jpg]

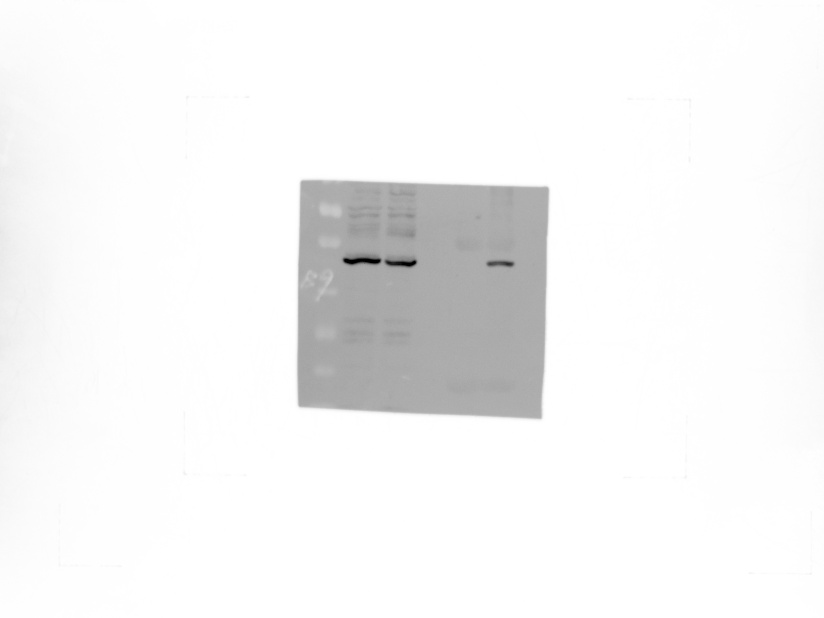

Supplement: Figure 1—source data 1. [file elife-92236-fig1-data1.zip › Figure_1-source_data_1/Figure_1-source_data_1_ Figure_1G_eIF4A3.jpg]

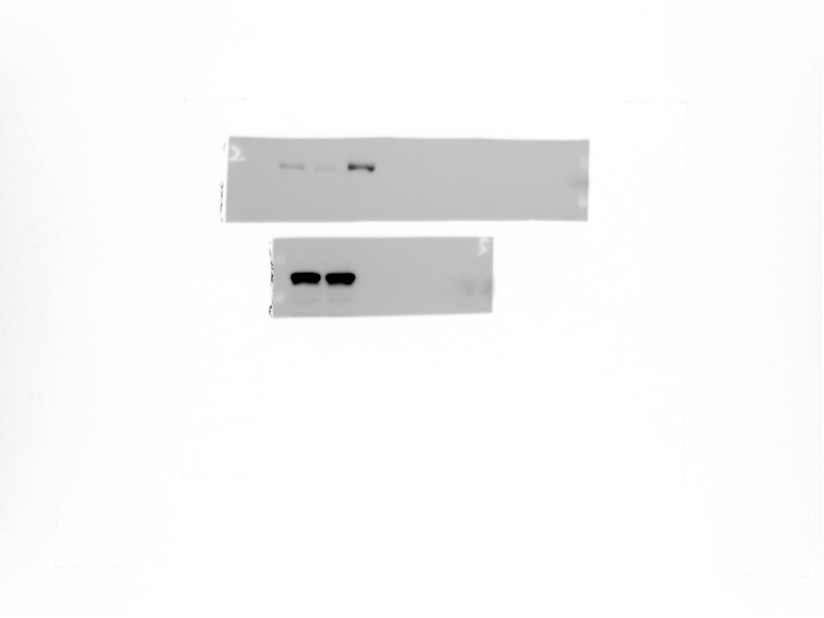

Supplement: Figure 1—source data 1. [file elife-92236-fig1-data1.zip › Figure_1-source_data_1/Figure_1-source_data_1_ Figure_1G_eIF4B.jpg]

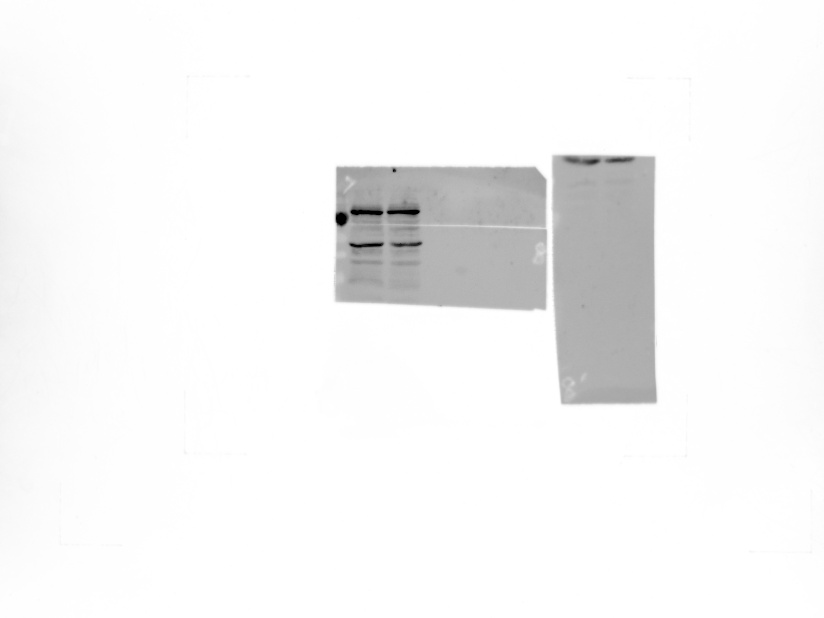

Supplement: Figure 1—source data 1. [file elife-92236-fig1-data1.zip › Figure_1-source_data_1/Figure_1-source_data_1_ Figure_1G_eIF4E.jpg]

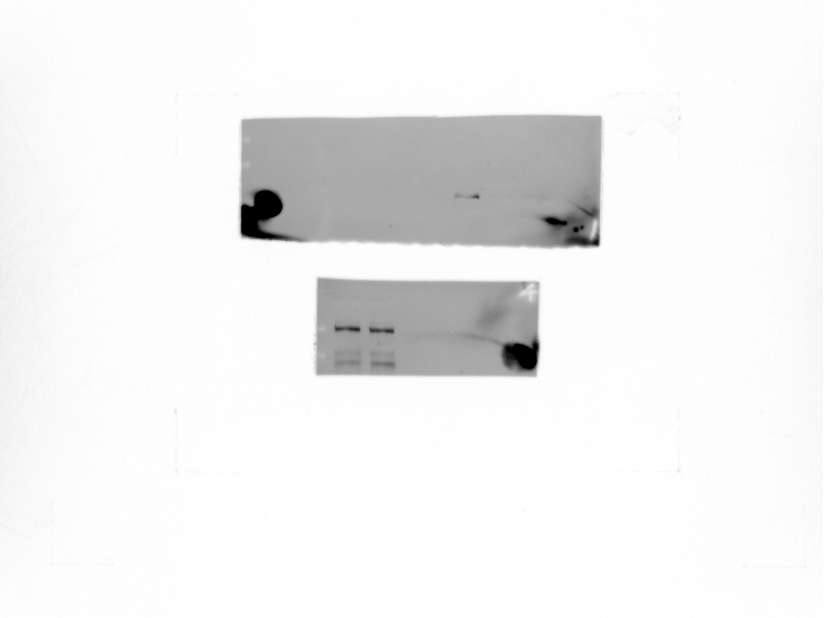

Supplement: Figure 1—source data 1. [file elife-92236-fig1-data1.zip › Figure_1-source_data_1/Figure_1-source_data_1_ Figure_1G_eIF4G.jpg]

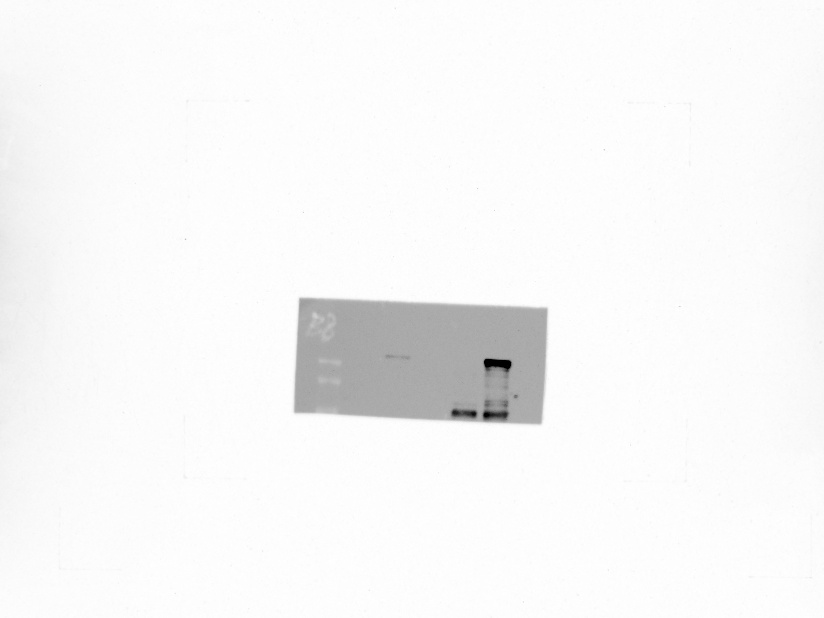

Supplement: Figure 1—source data 1. [file elife-92236-fig1-data1.zip › Figure_1-source_data_1/Figure_1-source_data_1_ Figure_1G_FLAG.jpg]

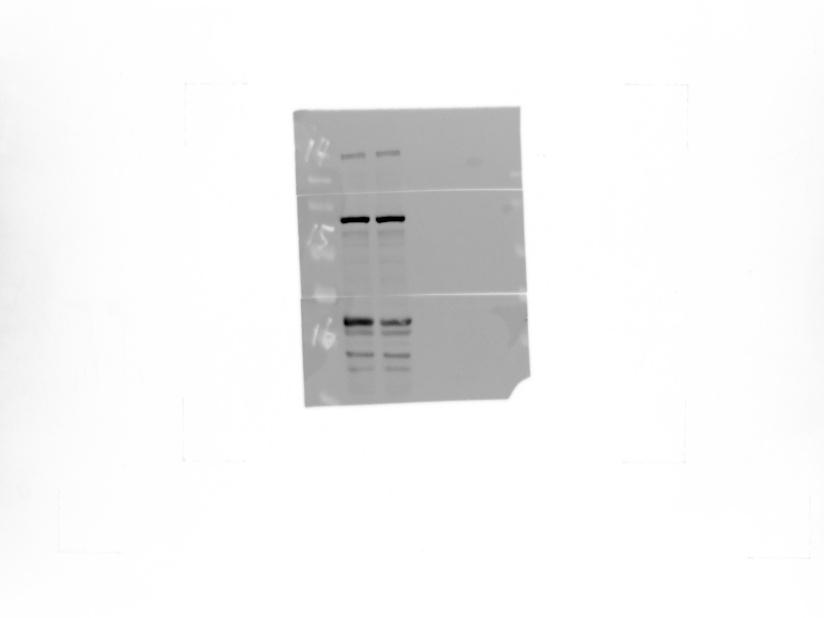

Supplement: Figure 1—source data 1. [file elife-92236-fig1-data1.zip › Figure_1-source_data_1/Figure_1-source_data_1_ Figure_1G_PCDC4.jpg]

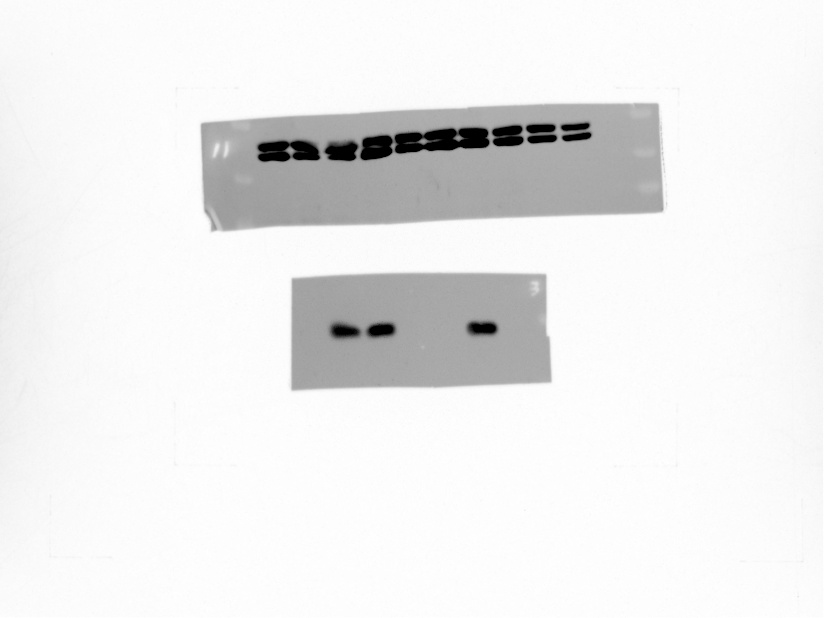

Supplement: Figure 1—source data 1. [file elife-92236-fig1-data1.zip › Figure_1-source_data_1/Figure_1-source_data_1_ Figure_1G_RBX1.jpg]

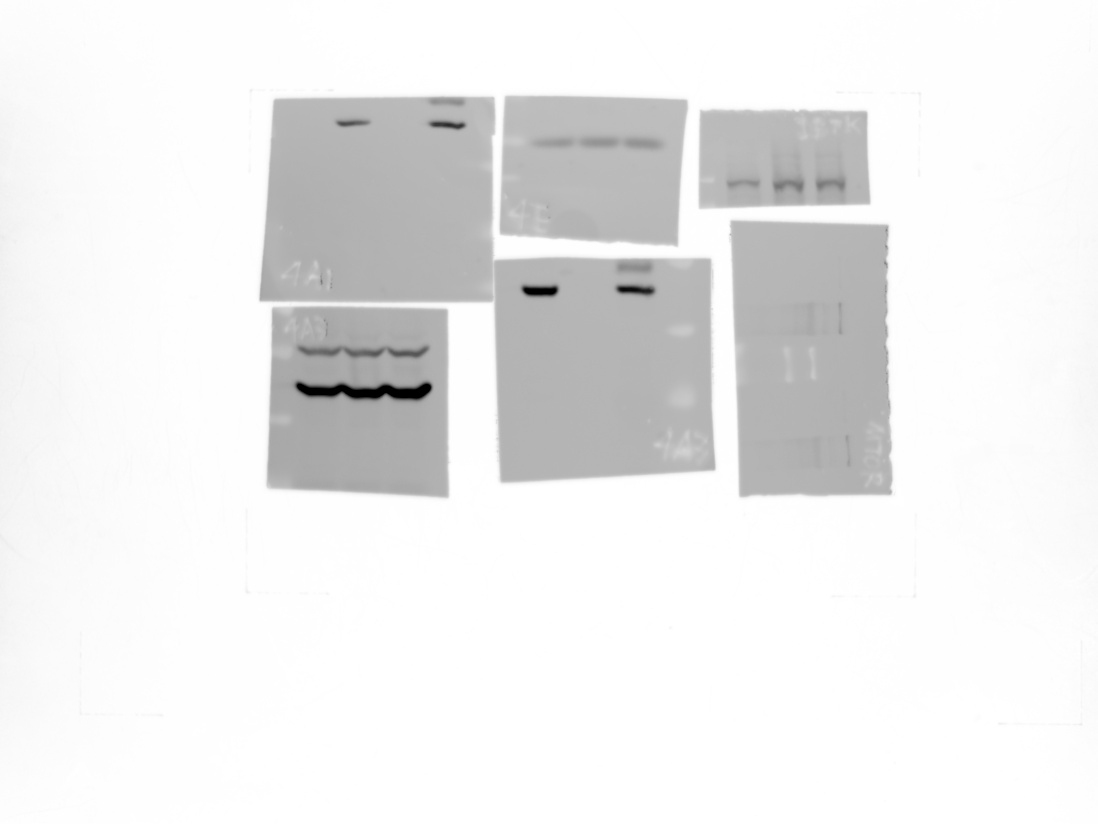

Supplement: Figure 1—source data 1. [file elife-92236-fig1-data1.zip › Figure_1-source_data_1/Figure_1-source_data_1_ Figure_1H_eIF4A1.jpg]

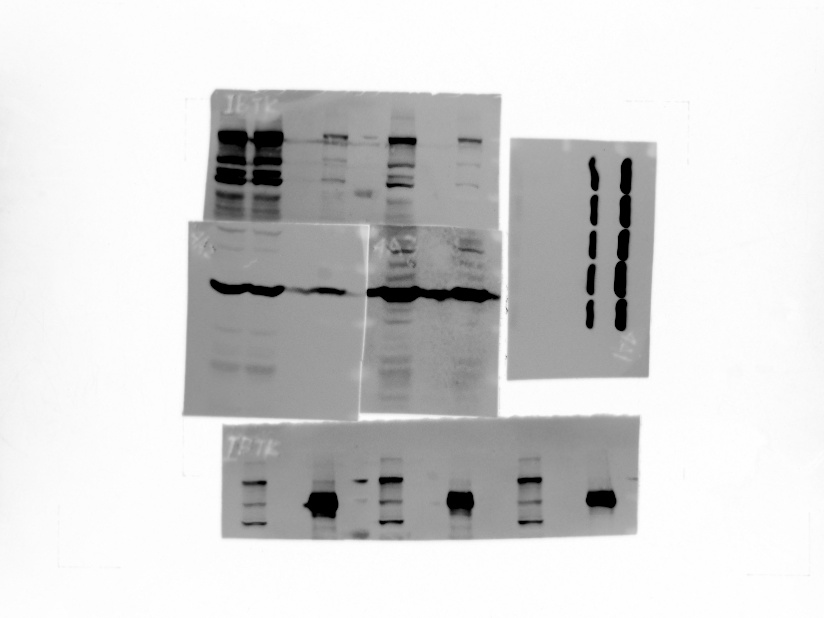

Supplement: Figure 1—source data 1. [file elife-92236-fig1-data1.zip › Figure_1-source_data_1/Figure_1-source_data_1_ Figure_1H_IBTK.jpg]

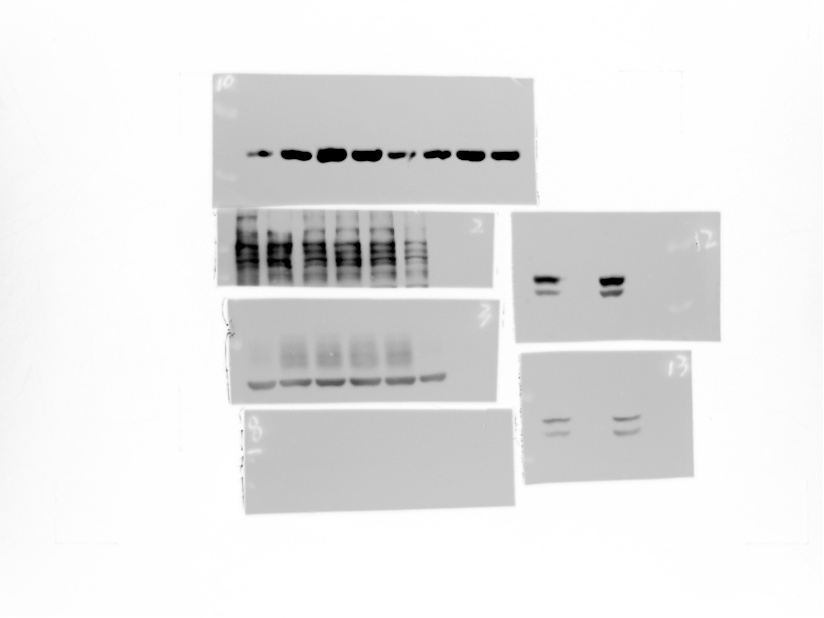

Supplement: Figure 1—source data 1. [file elife-92236-fig1-data1.zip › Figure_1-source_data_1/Figure_1-source_data_1_ Figure_1I_eIF4A2.jpg]

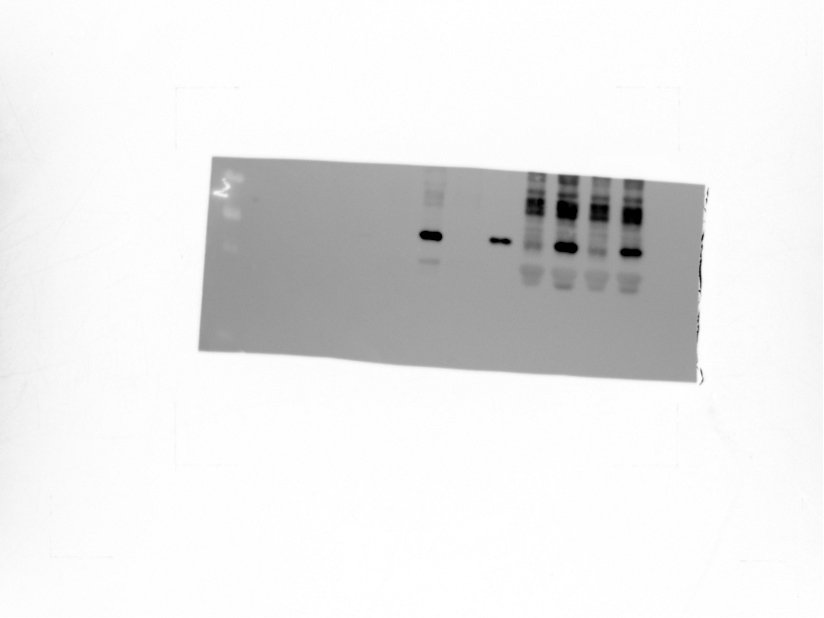

Supplement: Figure 1—source data 1. [file elife-92236-fig1-data1.zip › Figure_1-source_data_1/Figure_1-source_data_1_ Figure_1J_IBTK.jpg]

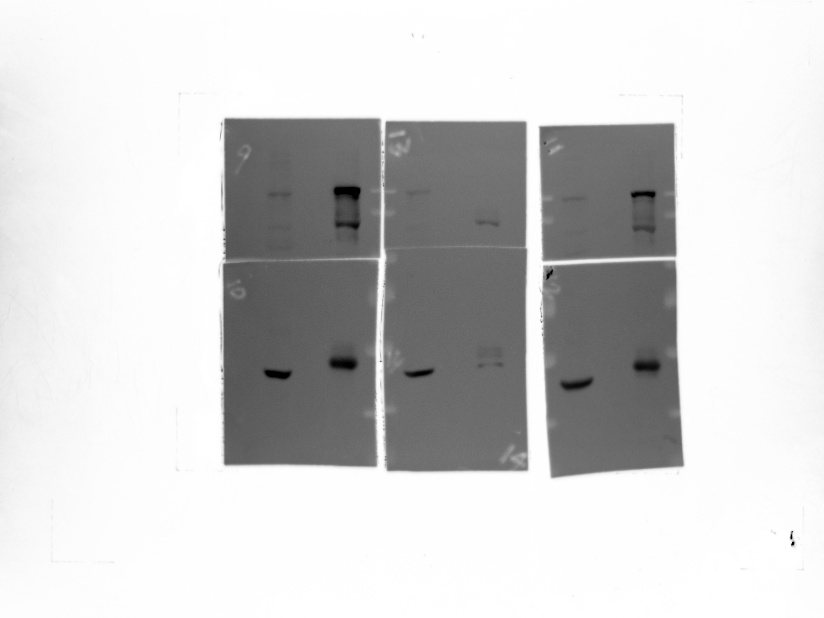

Supplement: Figure 1—source data 1. [file elife-92236-fig1-data1.zip › Figure_1-source_data_1/Figure_1-source_data_1_ Figure_1K_eIF4A1.jpg]

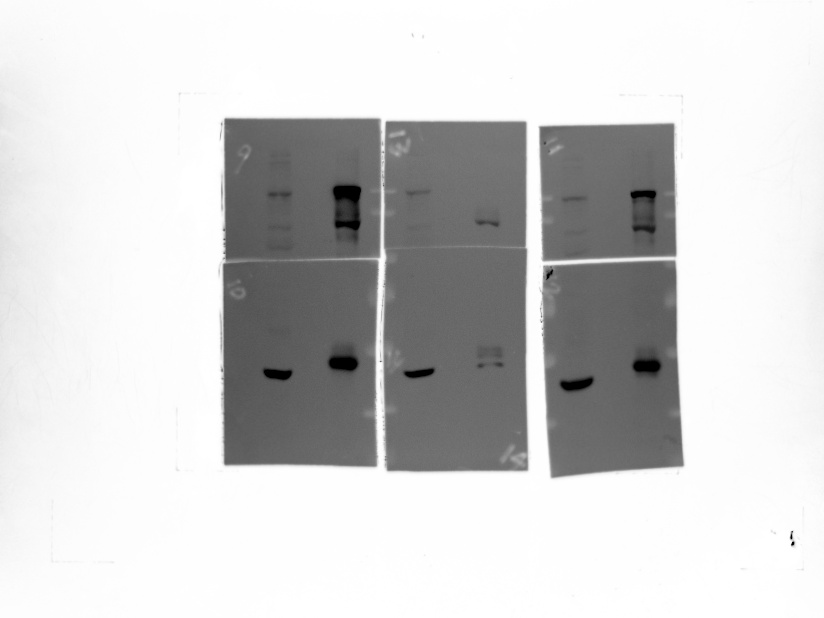

Supplement: Figure 1—source data 1. [file elife-92236-fig1-data1.zip › Figure_1-source_data_1/Figure_1-source_data_1_ Figure_1K_eIF4A2.jpg]

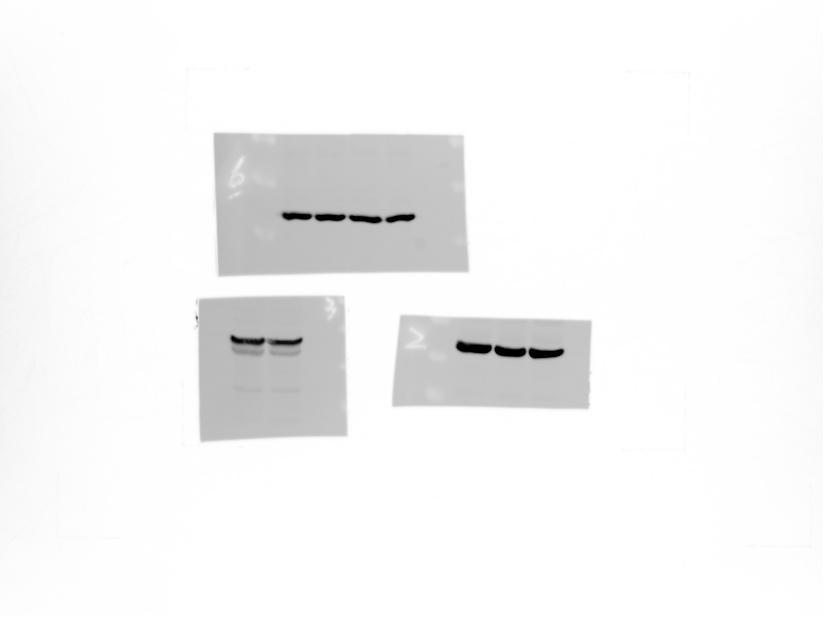

Supplement: Figure 2—source data 1. [file elife-92236-fig2-data1.zip › Figure_2-source_data_1/Figure_2-source_data_1_ Figure_2A_Actin.jpg]

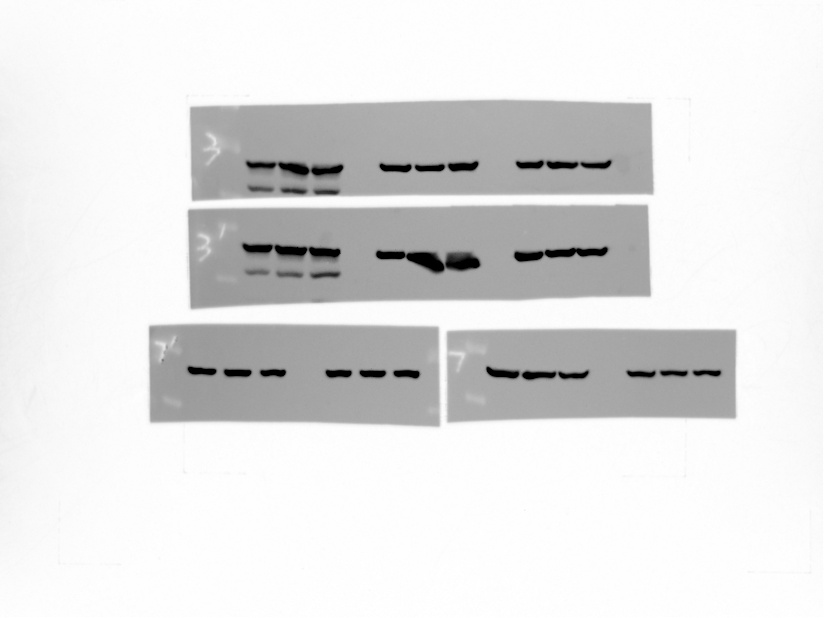

Supplement: Figure 2—source data 1. [file elife-92236-fig2-data1.zip › Figure_2-source_data_1/Figure_2-source_data_1_ Figure_2A_eIF4A1.jpg]

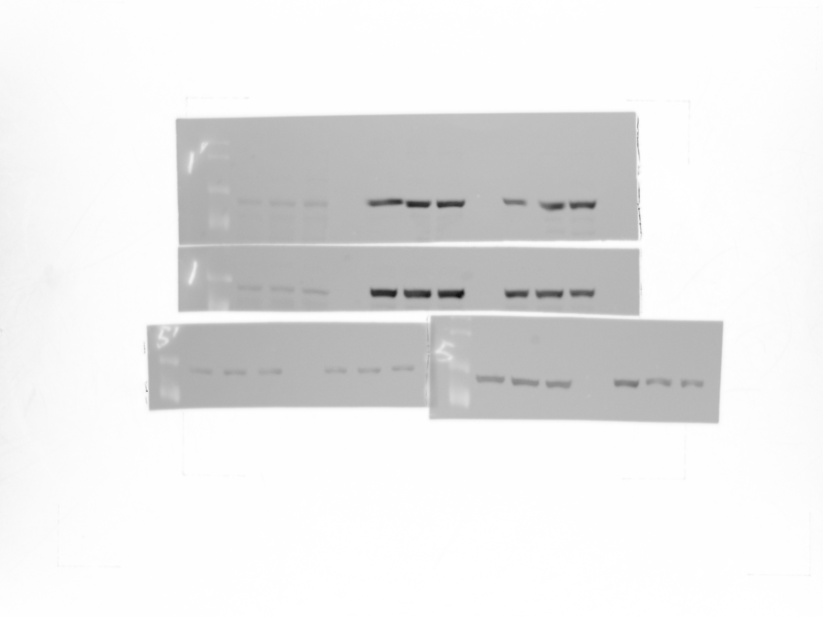

Supplement: Figure 2—source data 1. [file elife-92236-fig2-data1.zip › Figure_2-source_data_1/Figure_2-source_data_1_ Figure_2A_eIF4A2.jpg]

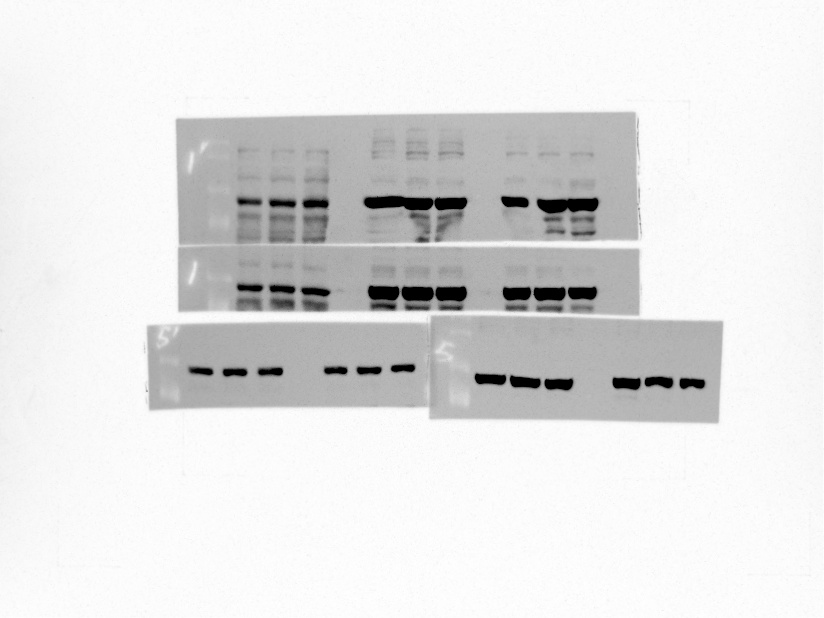

Supplement: Figure 2—source data 1. [file elife-92236-fig2-data1.zip › Figure_2-source_data_1/Figure_2-source_data_1_ Figure_2A_eIF4A3.jpg]

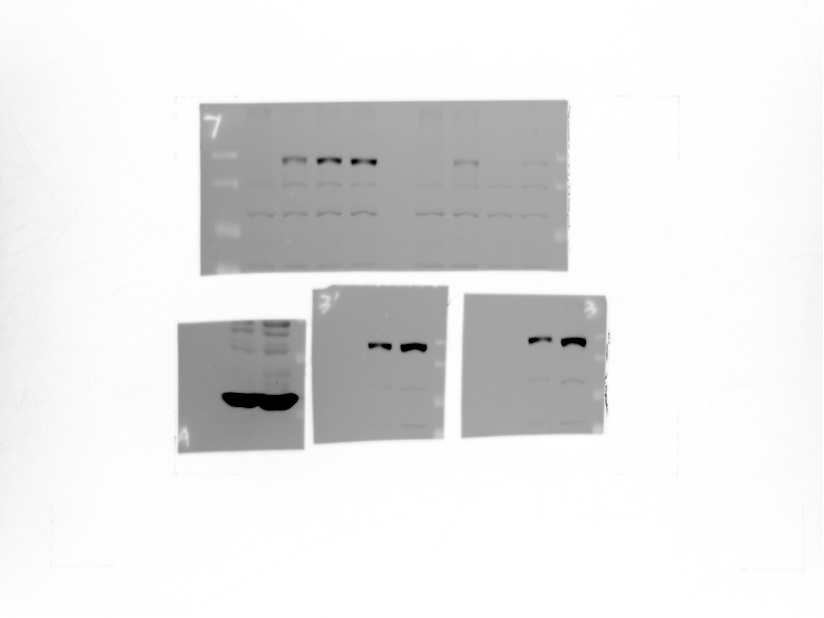

Supplement: Figure 2—source data 1. [file elife-92236-fig2-data1.zip › Figure_2-source_data_1/Figure_2-source_data_1_ Figure_2A_FLAG.jpg]

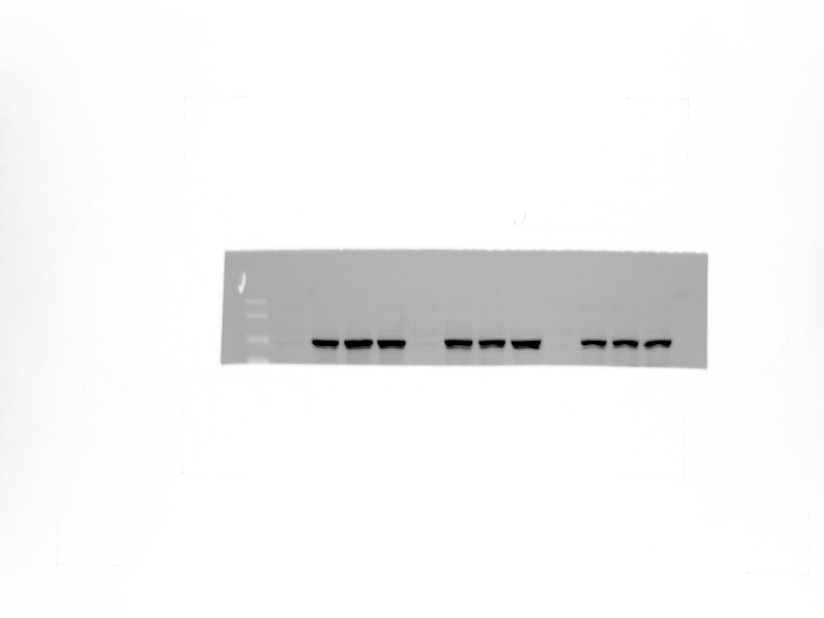

Supplement: Figure 2—source data 1. [file elife-92236-fig2-data1.zip › Figure_2-source_data_1/Figure_2-source_data_1_ Figure_2A_PDCD4.jpg]

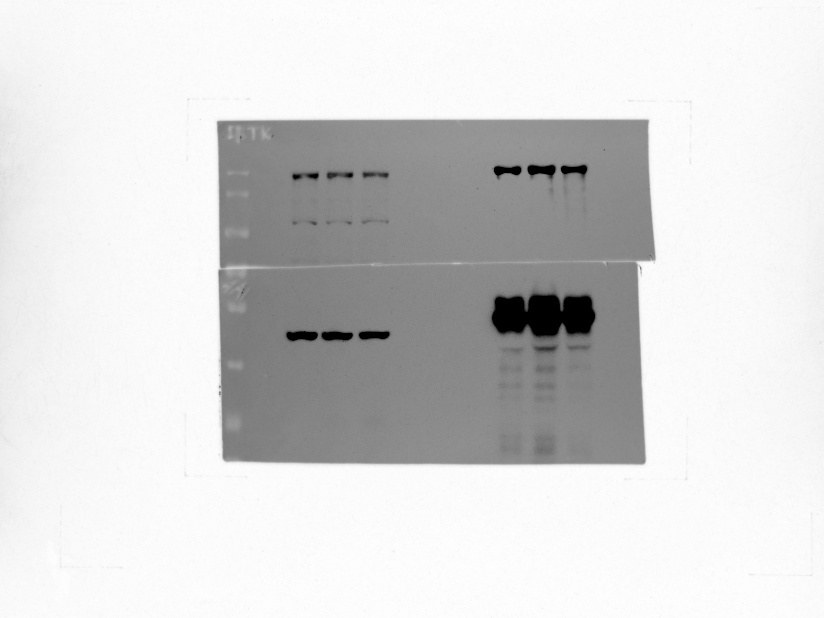

Supplement: Figure 2—source data 1. [file elife-92236-fig2-data1.zip › Figure_2-source_data_1/Figure_2-source_data_1_ Figure_2B_eIF4A1(293T).jpg]

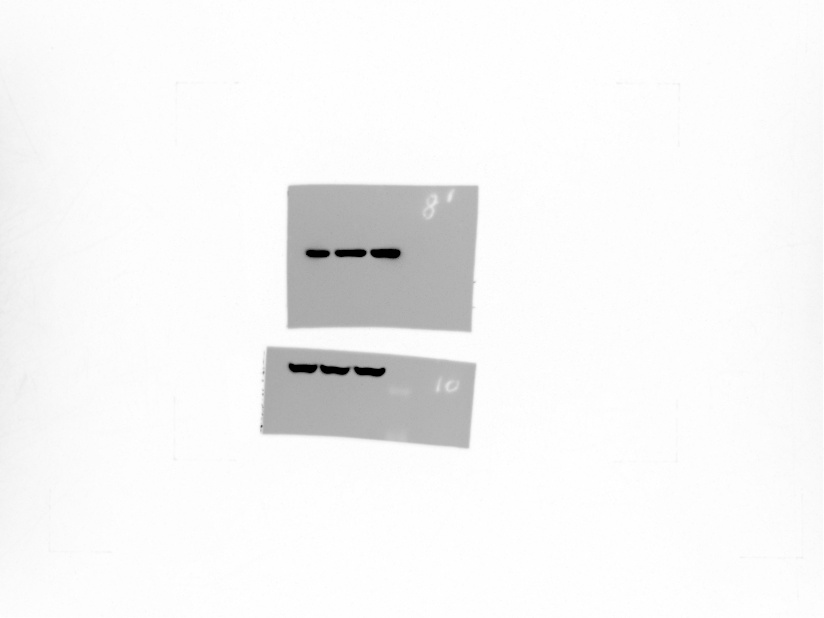

Supplement: Figure 2—source data 1. [file elife-92236-fig2-data1.zip › Figure_2-source_data_1/Figure_2-source_data_1_ Figure_2B_eIF4A2(H1299).jpg]

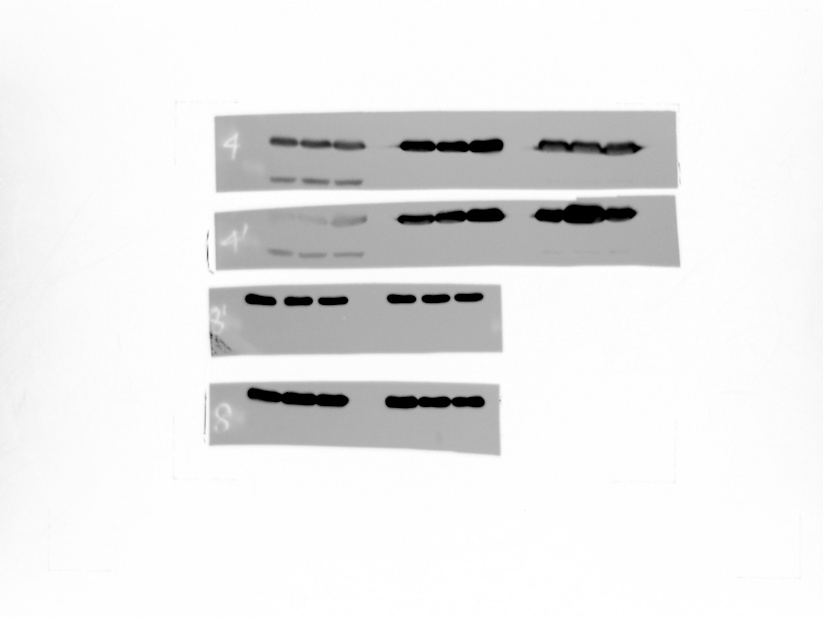

Supplement: Figure 2—source data 1. [file elife-92236-fig2-data1.zip › Figure_2-source_data_1/Figure_2-source_data_1_ Figure_2B_GAPDH(293T).jpg]

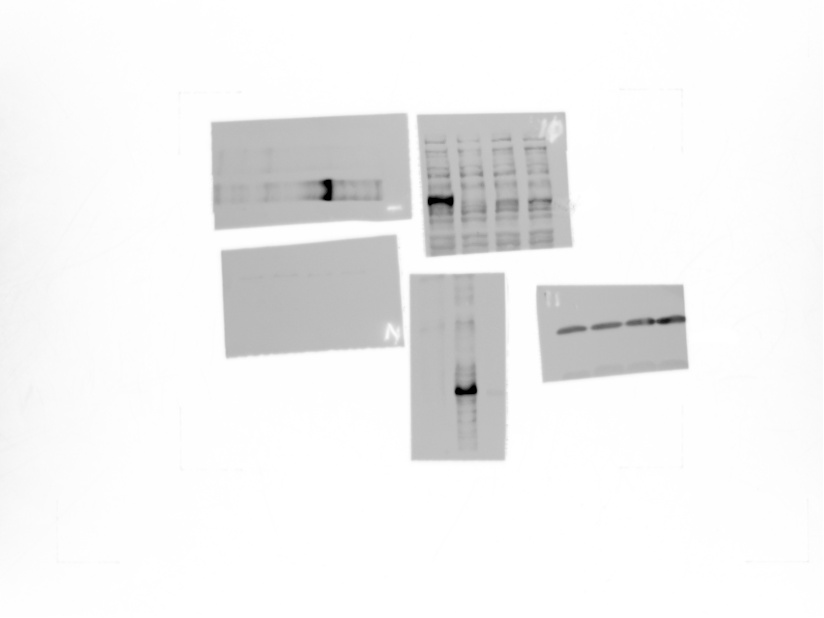

Supplement: Figure 2—source data 1. [file elife-92236-fig2-data1.zip › Figure_2-source_data_1/Figure_2-source_data_1_ Figure_2B_IBTK(293T).jpg]

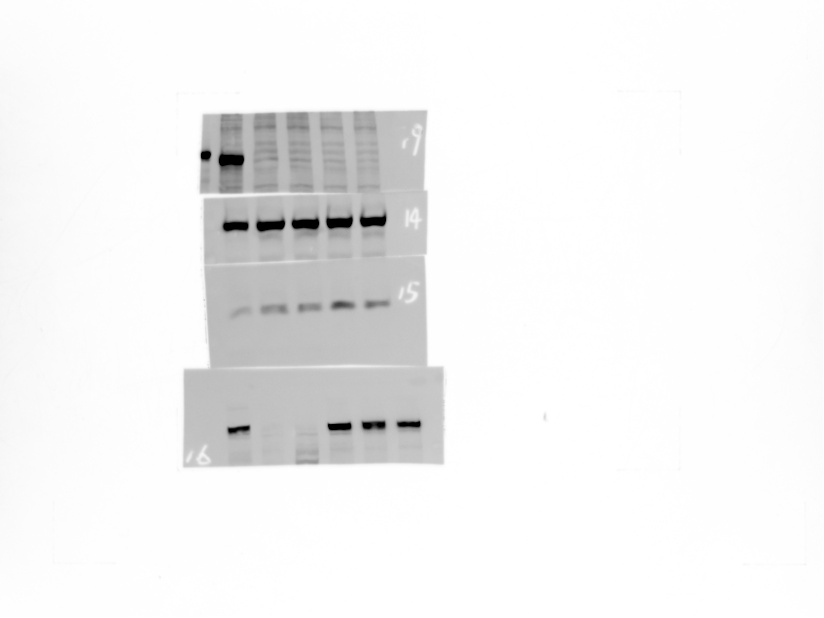

Supplement: Figure 2—source data 1. [file elife-92236-fig2-data1.zip › Figure_2-source_data_1/Figure_2-source_data_1_ Figure_2B_IBTK(H1299).jpg]

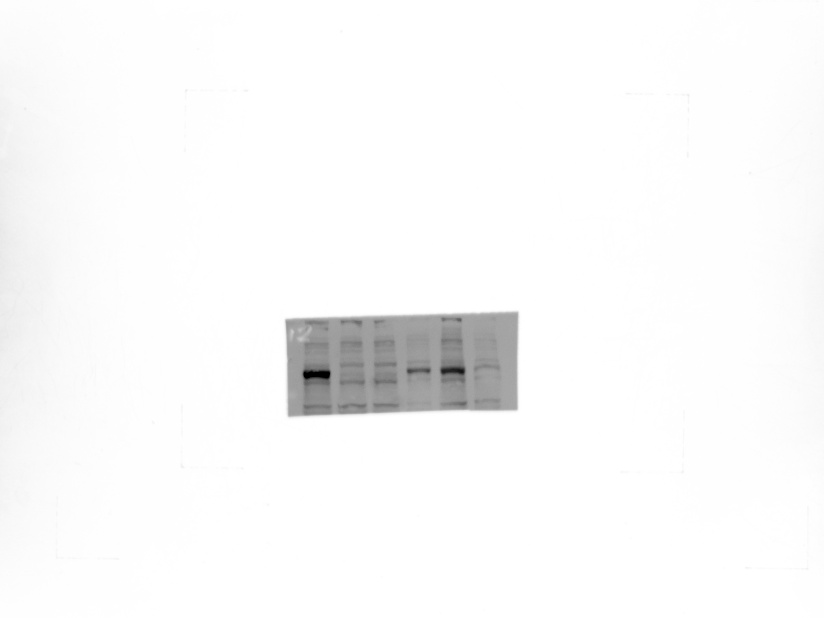

Supplement: Figure 2—source data 1. [file elife-92236-fig2-data1.zip › Figure_2-source_data_1/Figure_2-source_data_1_ Figure_2B_IBTK(SiHa).jpg]

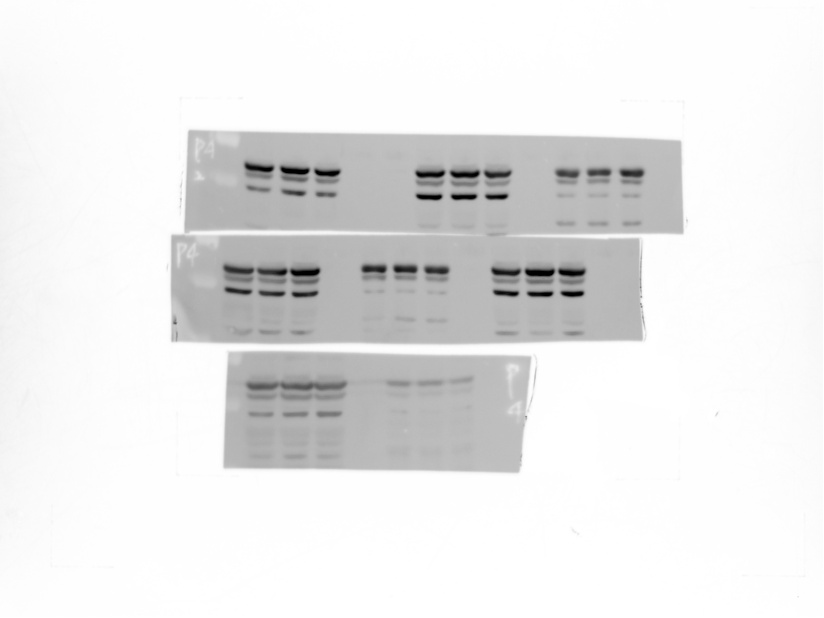

Supplement: Figure 2—source data 1. [file elife-92236-fig2-data1.zip › Figure_2-source_data_1/Figure_2-source_data_1_ Figure_2B_PDCD4(293T).jpg]

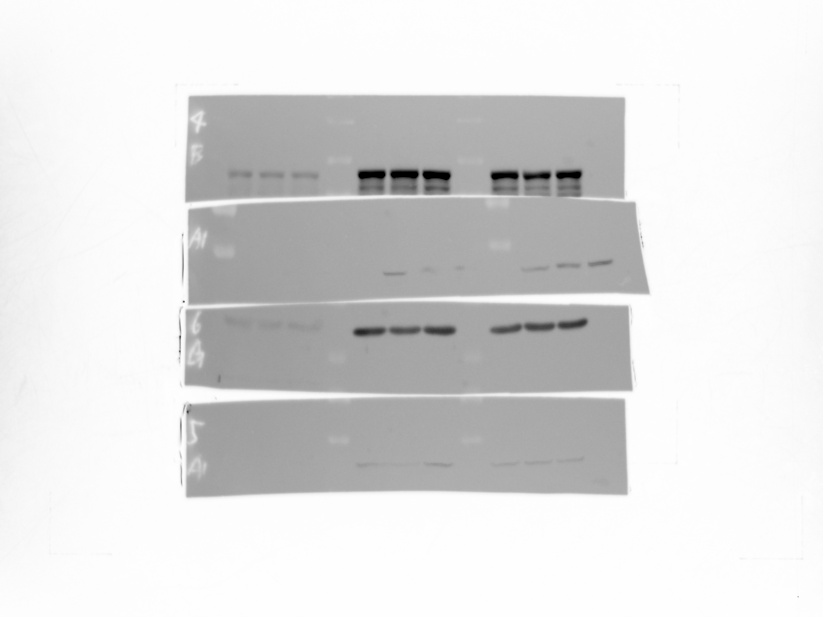

Supplement: Figure 2—source data 1. [file elife-92236-fig2-data1.zip › Figure_2-source_data_1/Figure_2-source_data_1_ Figure_2C_eIF4A1(HeLa).jpg]

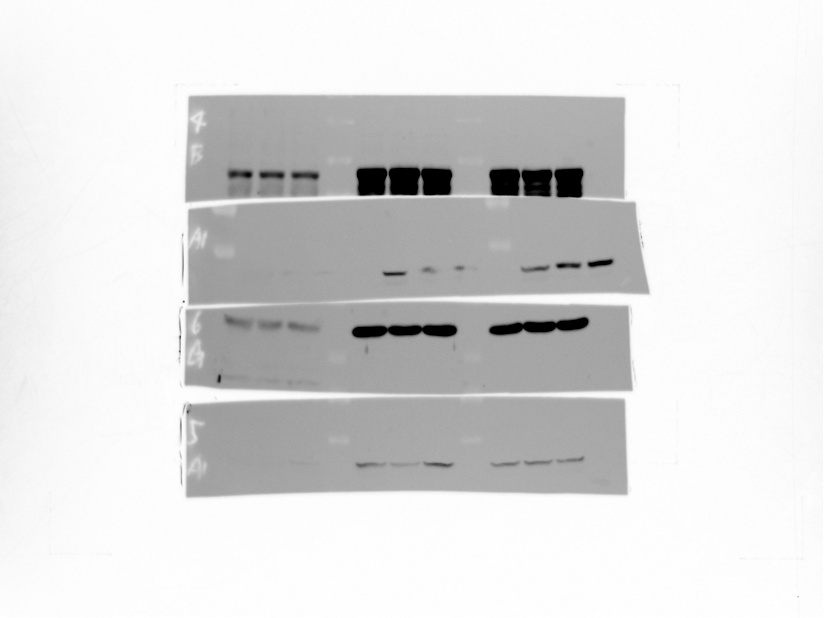

Supplement: Figure 2—source data 1. [file elife-92236-fig2-data1.zip › Figure_2-source_data_1/Figure_2-source_data_1_ Figure_2C_eIF4A1(SiHa).jpg]

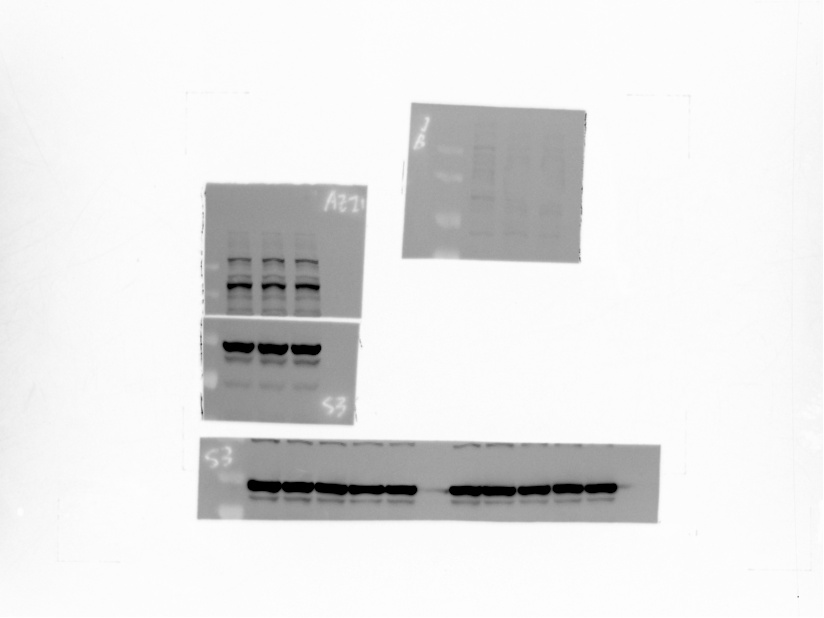

Supplement: Figure 2—source data 1. [file elife-92236-fig2-data1.zip › Figure_2-source_data_1/Figure_2-source_data_1_ Figure_2C_eIF4A2(SiHa).jpg]

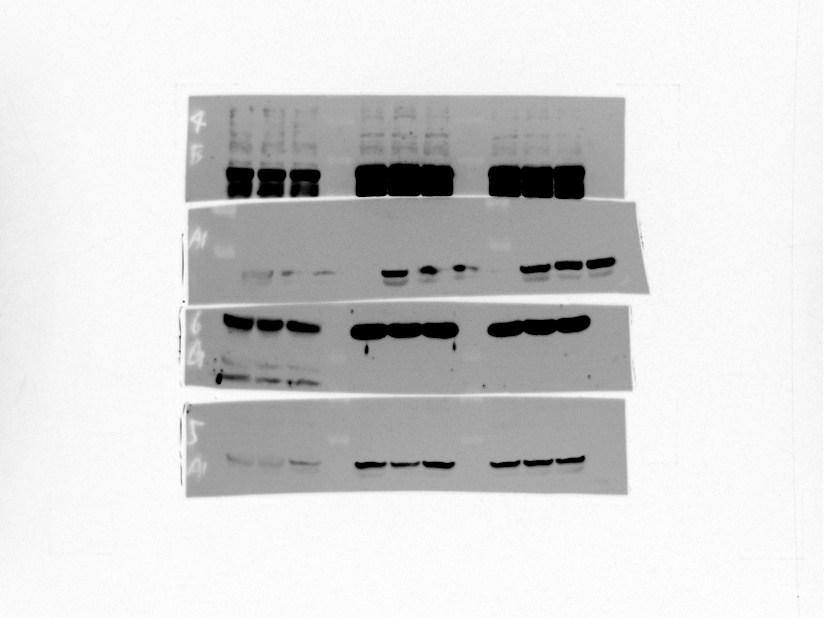

Supplement: Figure 2—source data 1. [file elife-92236-fig2-data1.zip › Figure_2-source_data_1/Figure_2-source_data_1_ Figure_2C_eIF4A3(HeLa).jpg]

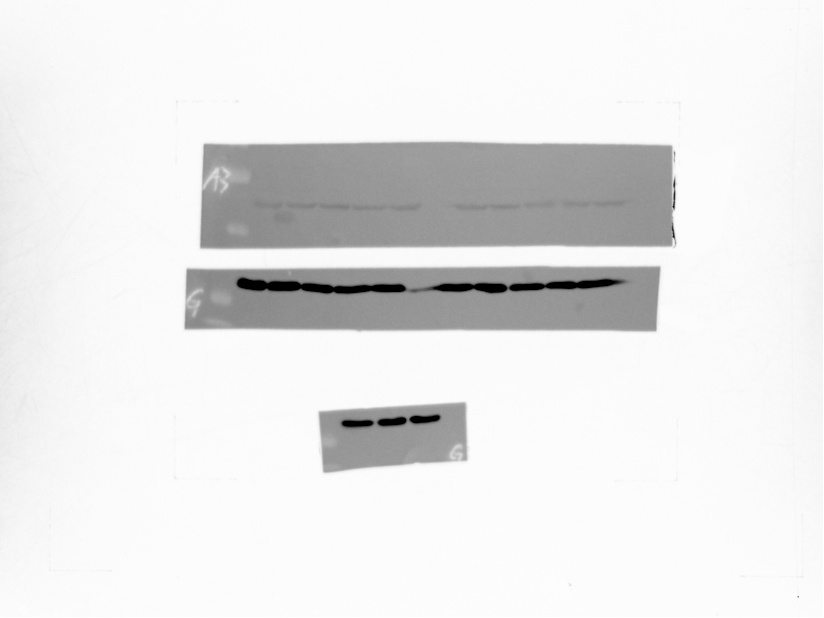

Supplement: Figure 2—source data 1. [file elife-92236-fig2-data1.zip › Figure_2-source_data_1/Figure_2-source_data_1_ Figure_2C_GAPDH(SiHa).jpg]

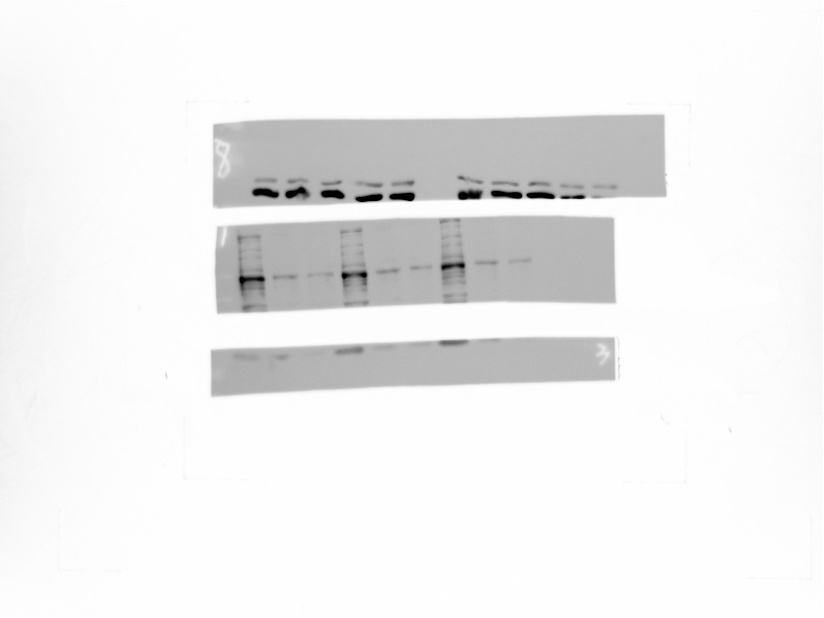

Supplement: Figure 2—source data 1. [file elife-92236-fig2-data1.zip › Figure_2-source_data_1/Figure_2-source_data_1_ Figure_2C_IBTK(HeLa).jpg]

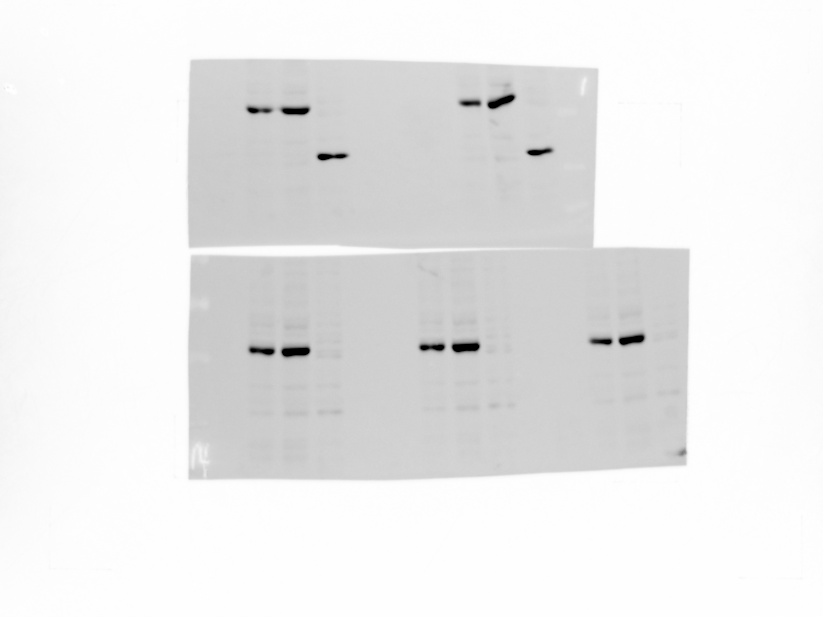

Supplement: Figure 2—source data 1. [file elife-92236-fig2-data1.zip › Figure_2-source_data_1/Figure_2-source_data_1_ Figure_2D_IP_FLAG(eIF4A1).jpg]

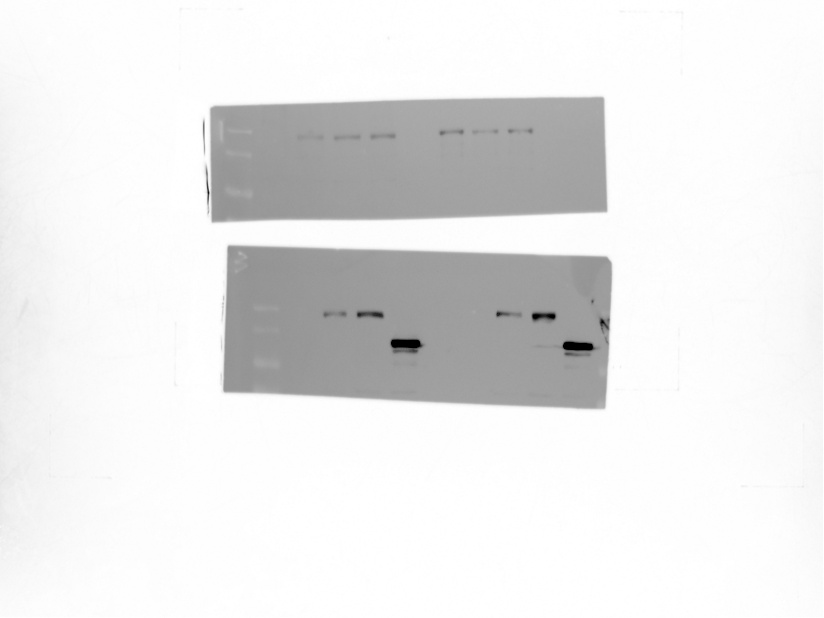

Supplement: Figure 2—source data 1. [file elife-92236-fig2-data1.zip › Figure_2-source_data_1/Figure_2-source_data_1_ Figure_2D_IP_FLAG(eIF4A2).jpg]

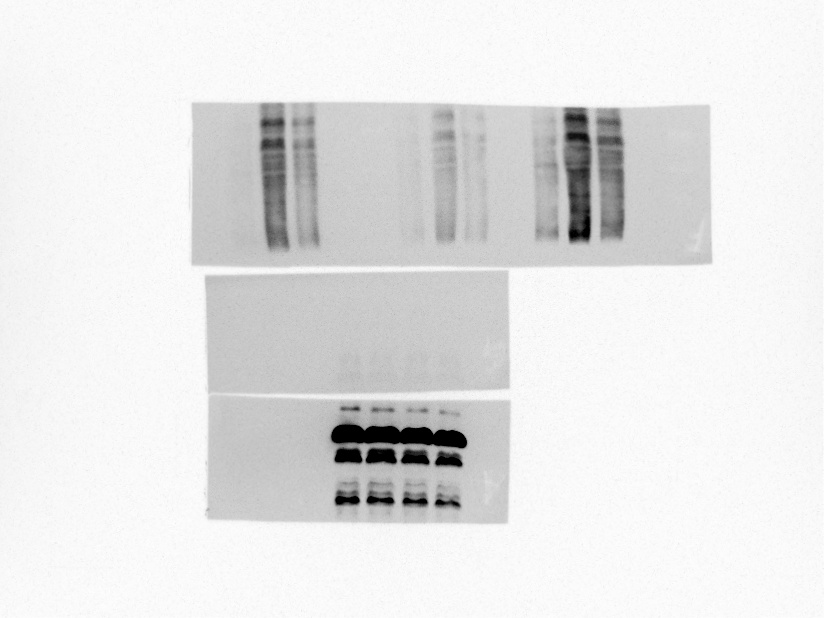

Supplement: Figure 2—source data 1. [file elife-92236-fig2-data1.zip › Figure_2-source_data_1/Figure_2-source_data_1_ Figure_2D_IP_HA(eIF4A1).jpg]

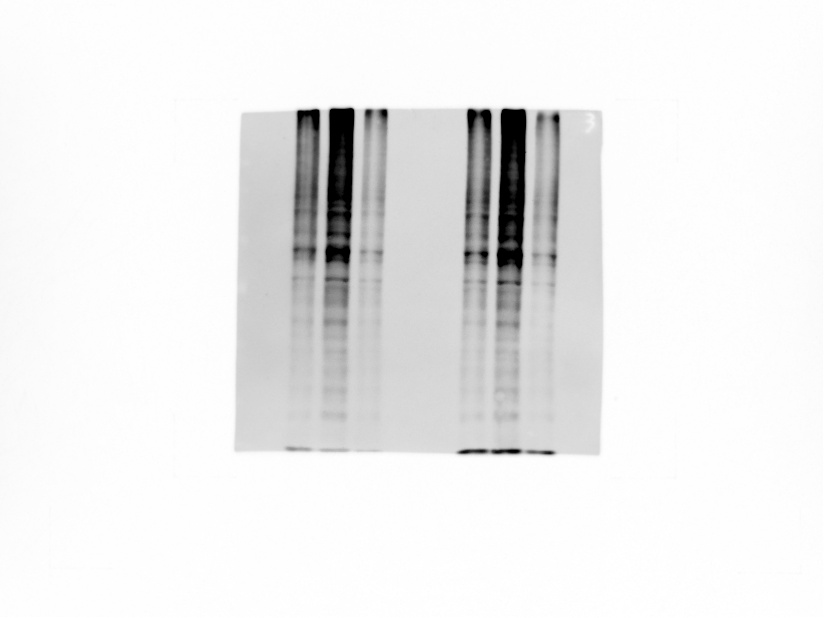

Supplement: Figure 2—source data 1. [file elife-92236-fig2-data1.zip › Figure_2-source_data_1/Figure_2-source_data_1_ Figure_2D_IP_HA(eIF4A2).jpg]

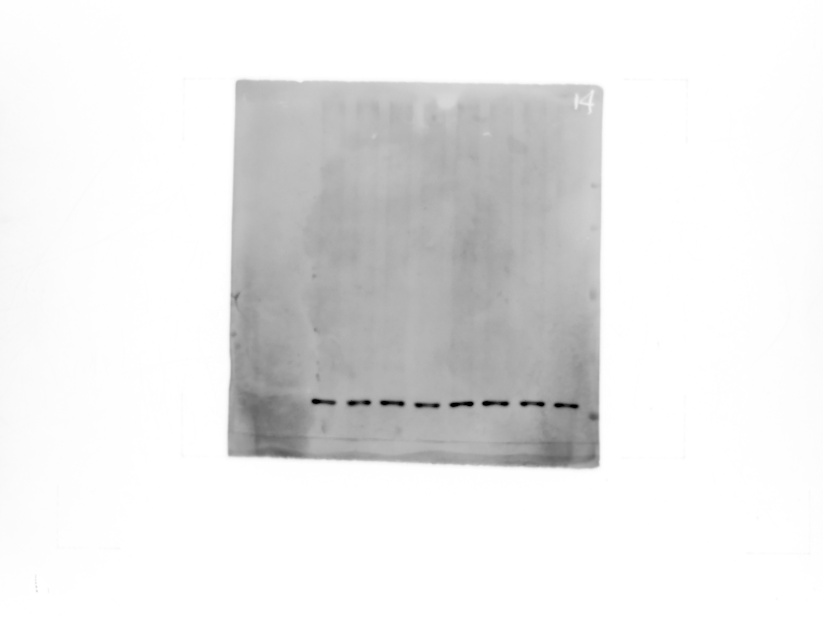

Supplement: Figure 2—source data 1. [file elife-92236-fig2-data1.zip › Figure_2-source_data_1/Figure_2-source_data_1_ Figure_2D_IP_HA(eIF4A3).jpg]

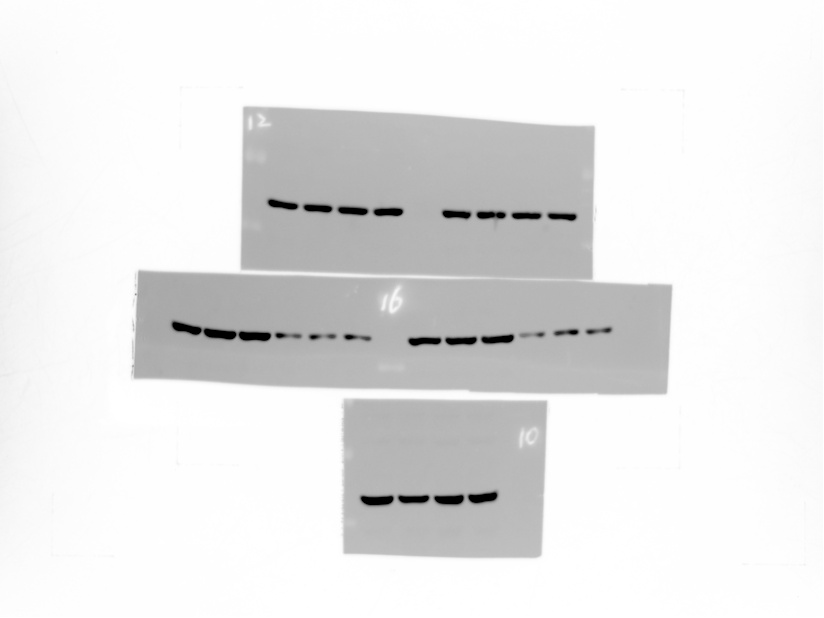

Supplement: Figure 2—source data 1. [file elife-92236-fig2-data1.zip › Figure_2-source_data_1/Figure_2-source_data_1_ Figure_2D_IP_PA(eIF4A1).jpg]

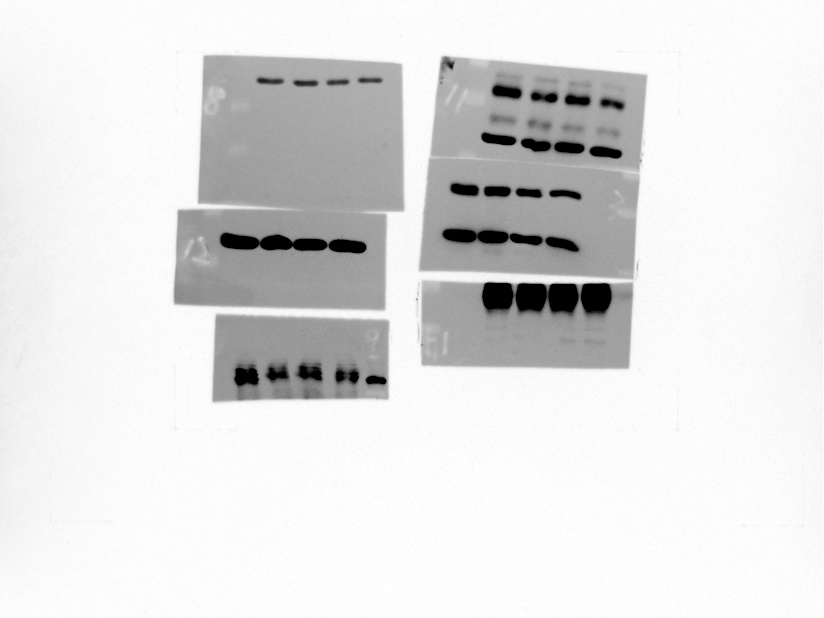

Supplement: Figure 2—source data 1. [file elife-92236-fig2-data1.zip › Figure_2-source_data_1/Figure_2-source_data_1_ Figure_2D_IP_PA(eIF4A2).jpg]

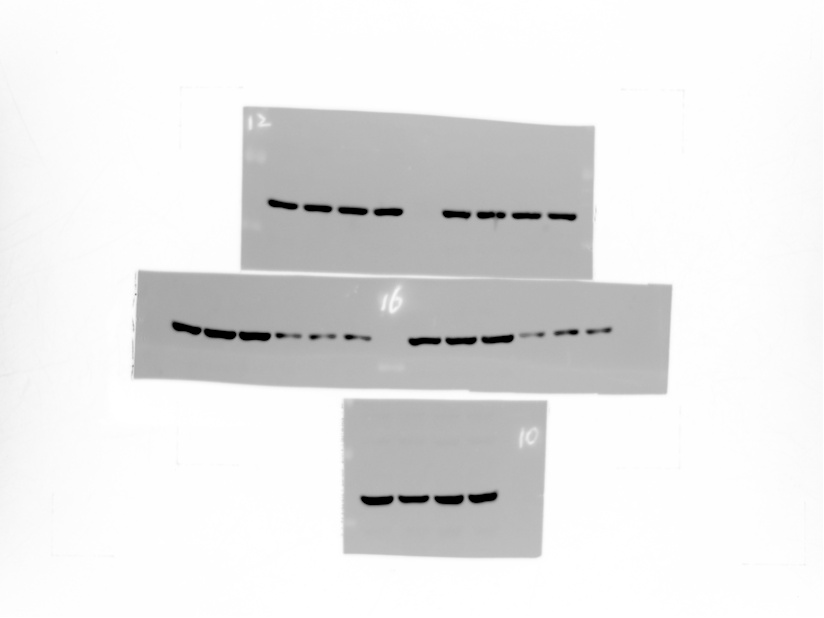

Supplement: Figure 2—source data 1. [file elife-92236-fig2-data1.zip › Figure_2-source_data_1/Figure_2-source_data_1_ Figure_2D_IP_PA(eIF4A3).jpg]

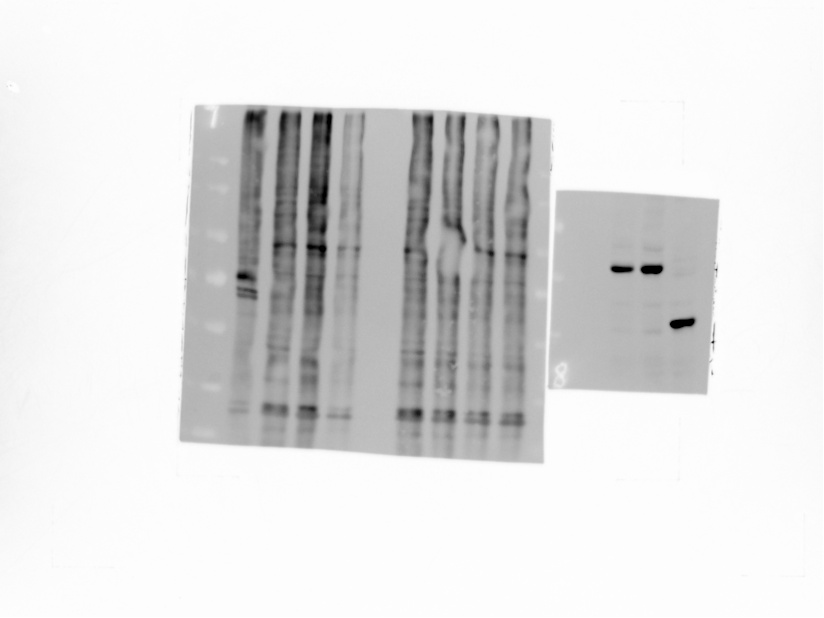

Supplement: Figure 2—source data 1. [file elife-92236-fig2-data1.zip › Figure_2-source_data_1/Figure_2-source_data_1_ Figure_2D_WCL_FLAG(eIF4A1).jpg]

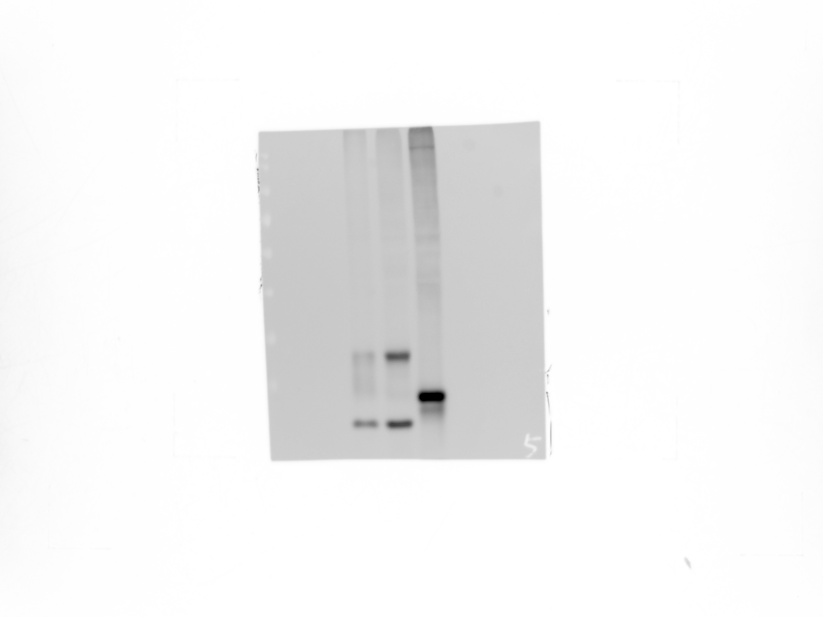

Supplement: Figure 2—source data 1. [file elife-92236-fig2-data1.zip › Figure_2-source_data_1/Figure_2-source_data_1_ Figure_2D_WCL_FLAG(eIF4A3).jpg]

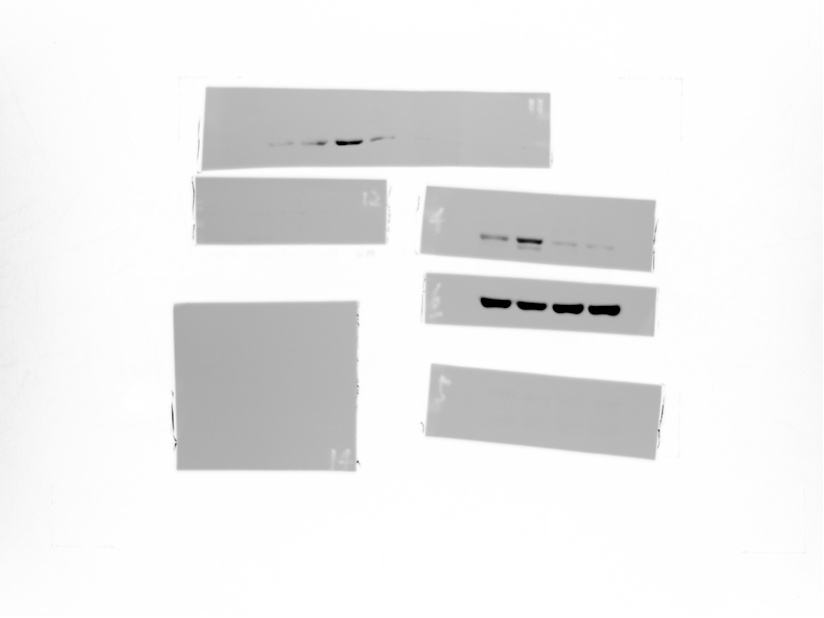

Supplement: Figure 2—source data 1. [file elife-92236-fig2-data1.zip › Figure_2-source_data_1/Figure_2-source_data_1_ Figure_2D_WCL_PA(eIF4A1).jpg]

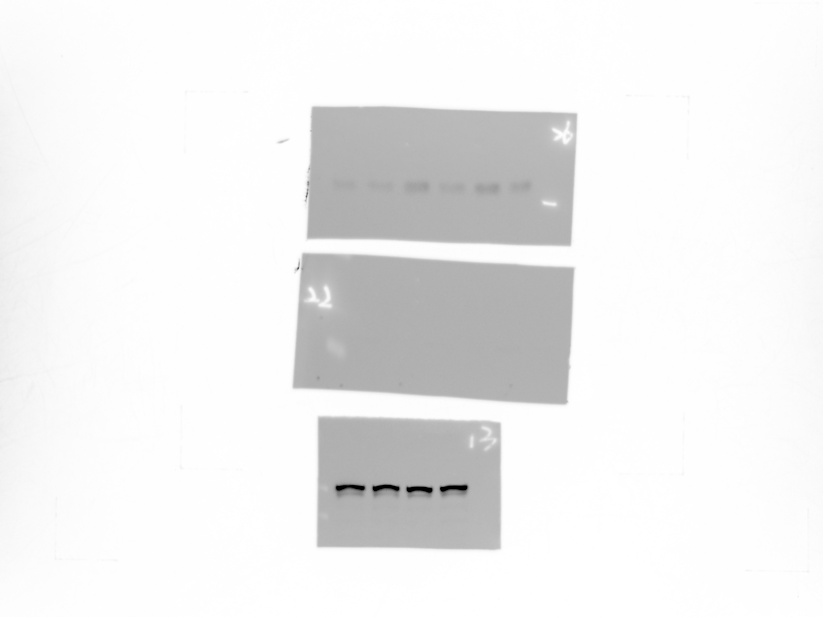

Supplement: Figure 2—source data 1. [file elife-92236-fig2-data1.zip › Figure_2-source_data_1/Figure_2-source_data_1_ Figure_2D_WCL_PA(eIF4A2).jpg]

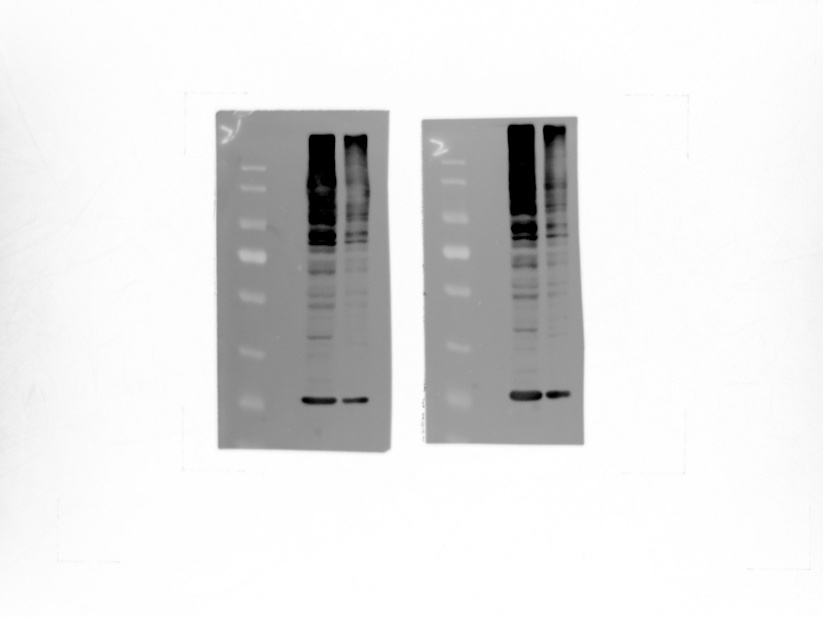

Supplement: Figure 2—source data 1. [file elife-92236-fig2-data1.zip › Figure_2-source_data_1/Figure_2-source_data_1_ Figure_2E_HA(eIF4A1).jpg]

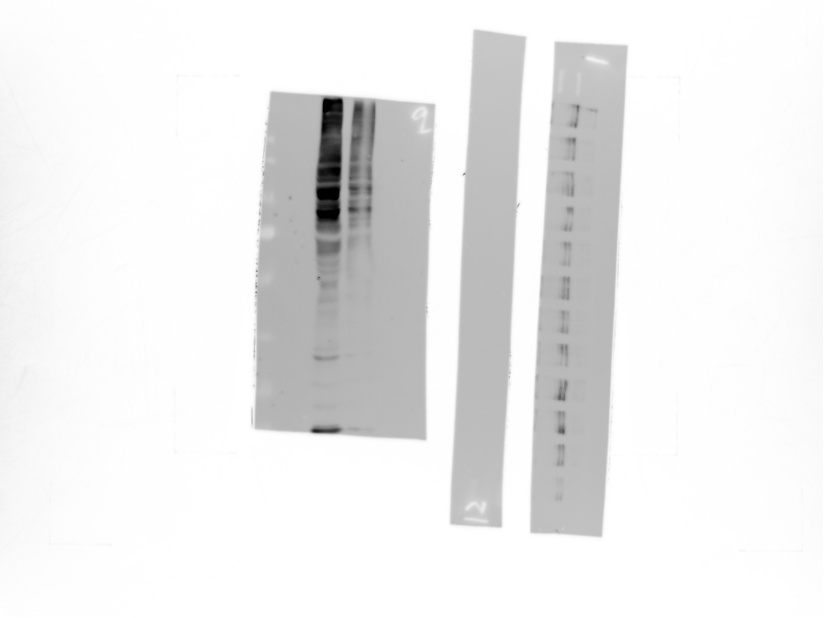

Supplement: Figure 2—source data 1. [file elife-92236-fig2-data1.zip › Figure_2-source_data_1/Figure_2-source_data_1_ Figure_2E_HA(eIF4A2).jpg]

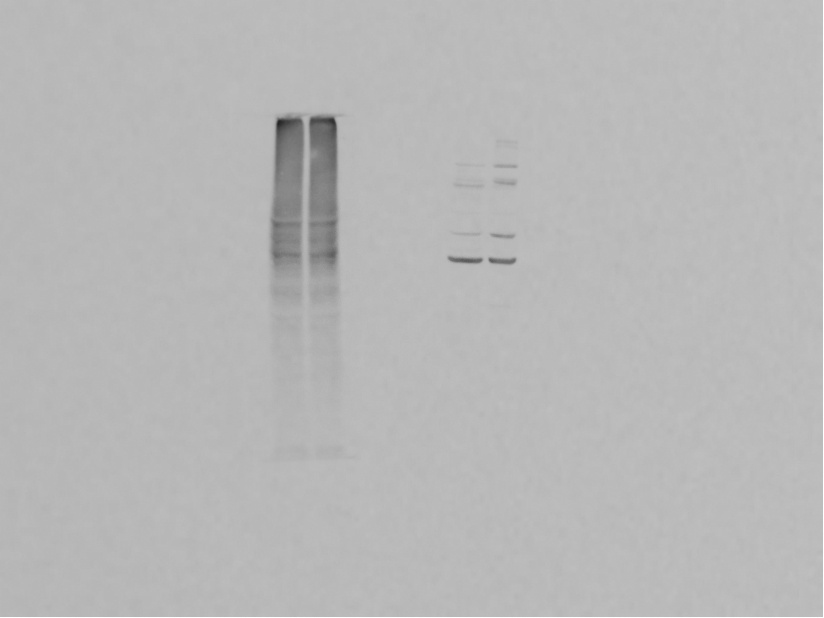

Supplement: Figure 2—source data 1. [file elife-92236-fig2-data1.zip › Figure_2-source_data_1/Figure_2-source_data_1_ Figure_2E_HA(eIF4A3).jpg]

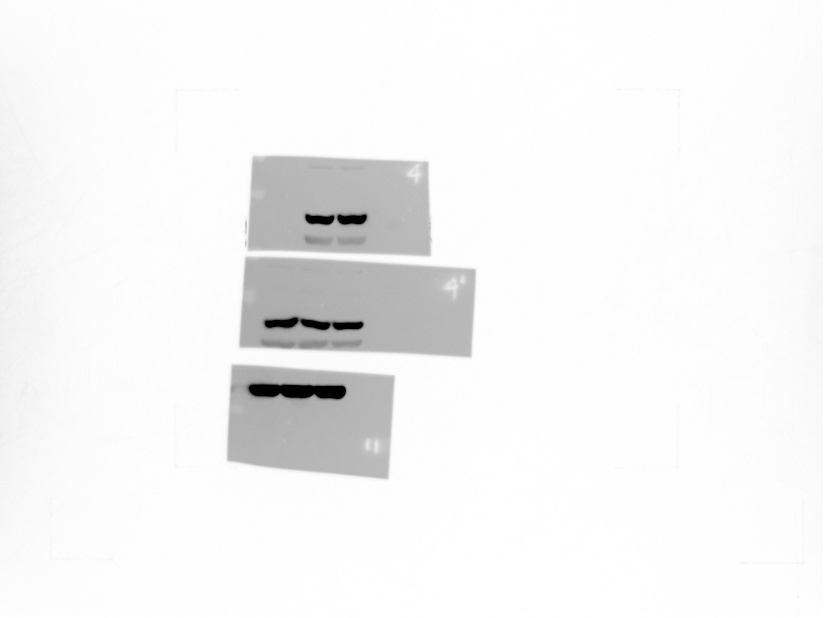

Supplement: Figure 2—source data 1. [file elife-92236-fig2-data1.zip › Figure_2-source_data_1/Figure_2-source_data_1_ Figure_2E_IP_eIF4A1.jpg]

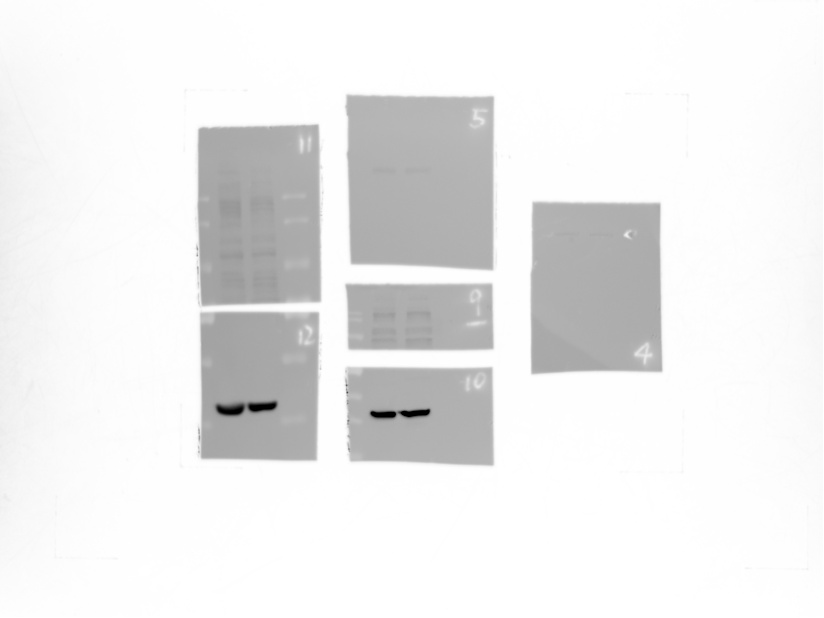

Supplement: Figure 2—source data 1. [file elife-92236-fig2-data1.zip › Figure_2-source_data_1/Figure_2-source_data_1_ Figure_2E_IP_eIF4A2.jpg]

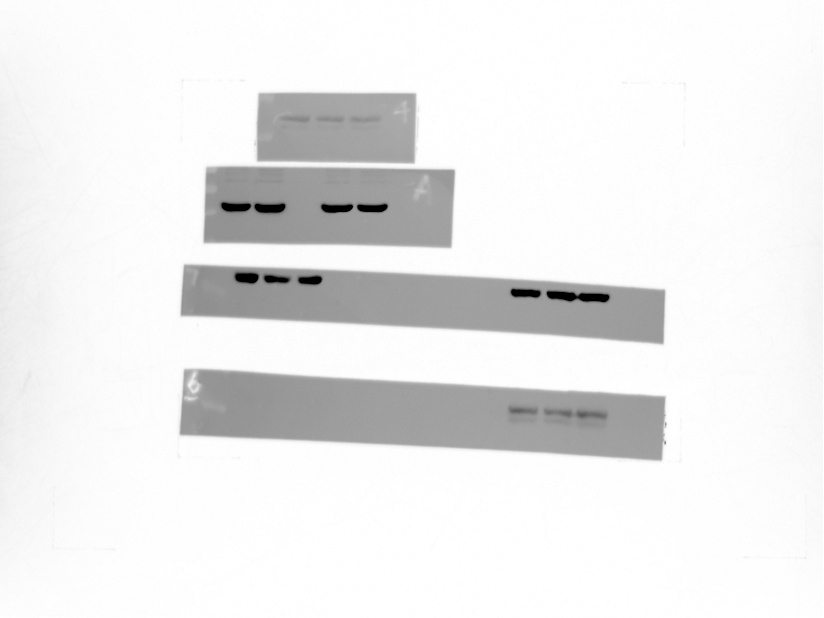

Supplement: Figure 2—source data 1. [file elife-92236-fig2-data1.zip › Figure_2-source_data_1/Figure_2-source_data_1_ Figure_2E_IP_eIF4A3.jpg]

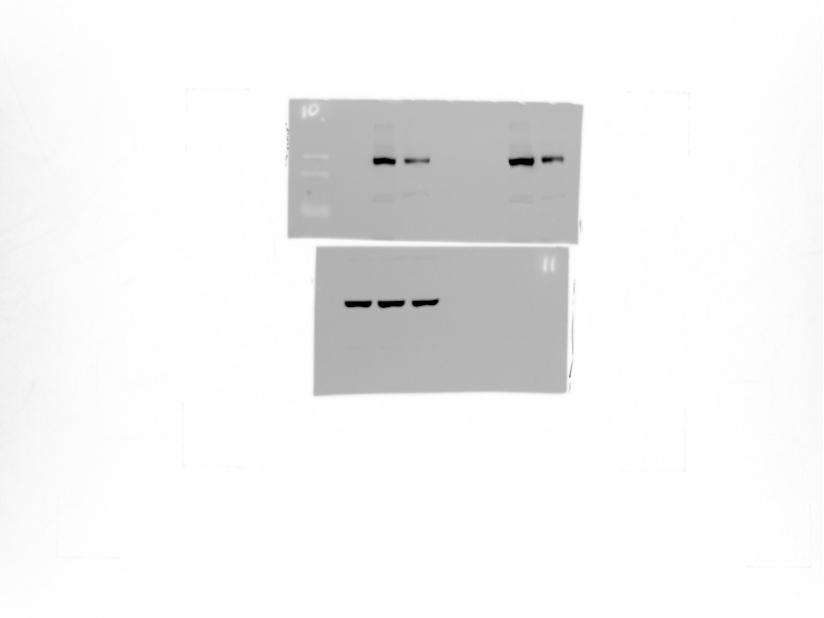

Supplement: Figure 2—source data 1. [file elife-92236-fig2-data1.zip › Figure_2-source_data_1/Figure_2-source_data_1_ Figure_2E_WCL_eIF4A2.jpg]

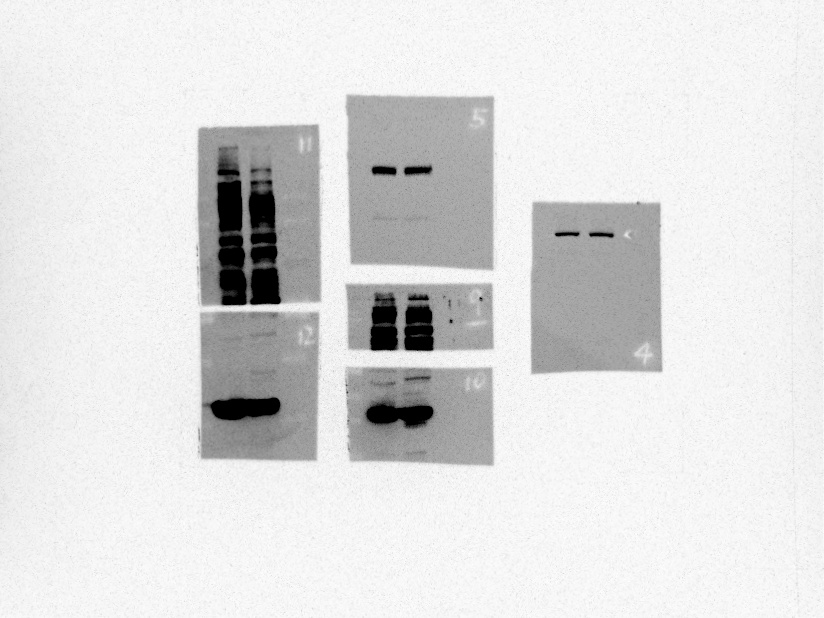

Supplement: Figure 2—source data 1. [file elife-92236-fig2-data1.zip › Figure_2-source_data_1/Figure_2-source_data_1_ Figure_2E_WCL_IBTK(eIF4A1).jpg]

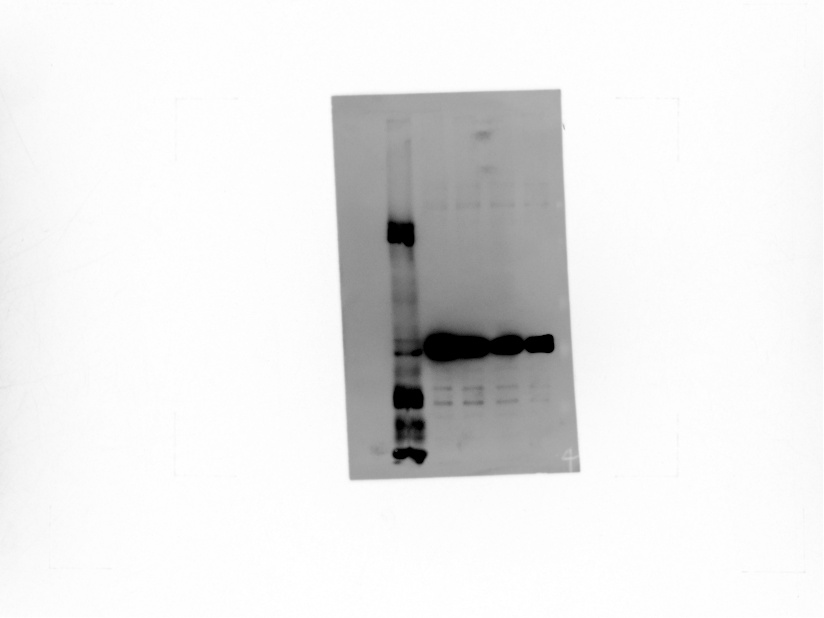

Supplement: Figure 2—source data 1. [file elife-92236-fig2-data1.zip › Figure_2-source_data_1/Figure_2-source_data_1_ Figure_2F_eIF4A1.jpg]

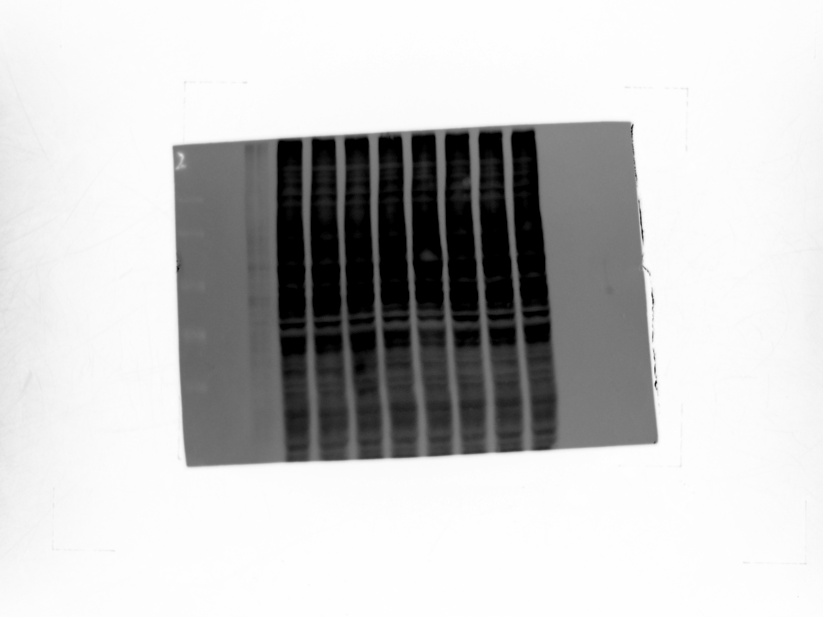

Supplement: Figure 2—source data 1. [file elife-92236-fig2-data1.zip › Figure_2-source_data_1/Figure_2-source_data_1_ Figure_2G_IP_HA(left panel).jpg]

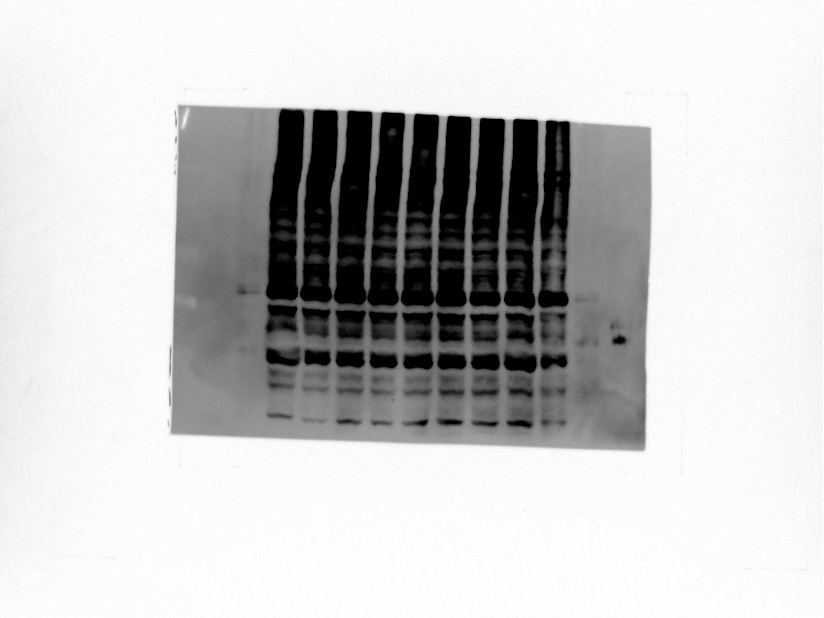

Supplement: Figure 2—source data 1. [file elife-92236-fig2-data1.zip › Figure_2-source_data_1/Figure_2-source_data_1_ Figure_2G_IP_HA(right panel).jpg]

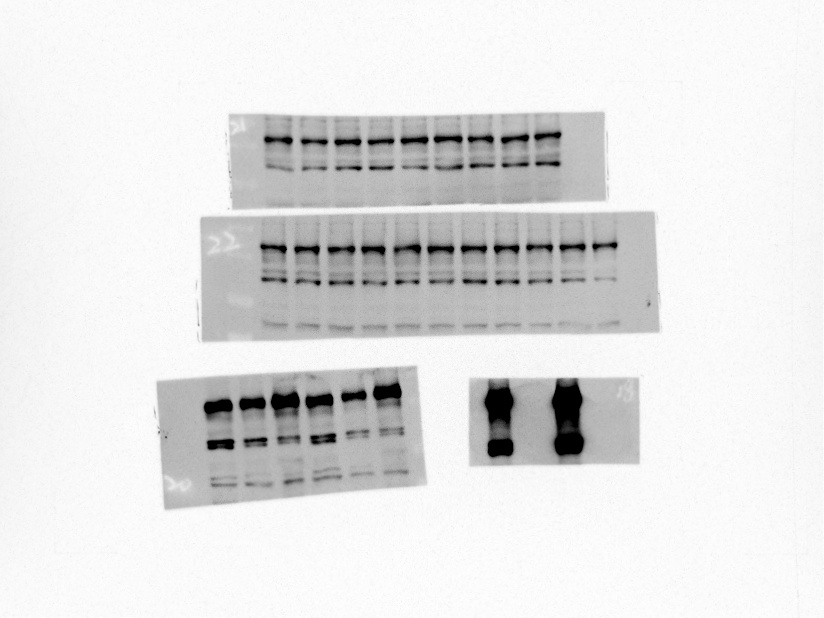

Supplement: Figure 2—source data 1. [file elife-92236-fig2-data1.zip › Figure_2-source_data_1/Figure_2-source_data_1_ Figure_2G_IP_PA(left panel).jpg]

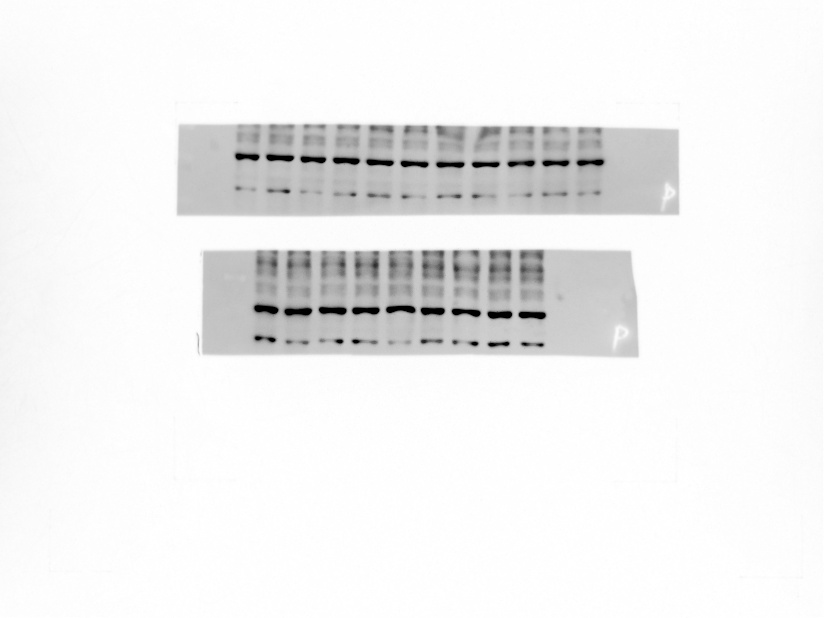

Supplement: Figure 2—source data 1. [file elife-92236-fig2-data1.zip › Figure_2-source_data_1/Figure_2-source_data_1_ Figure_2G_IP_PA(right panle).jpg]

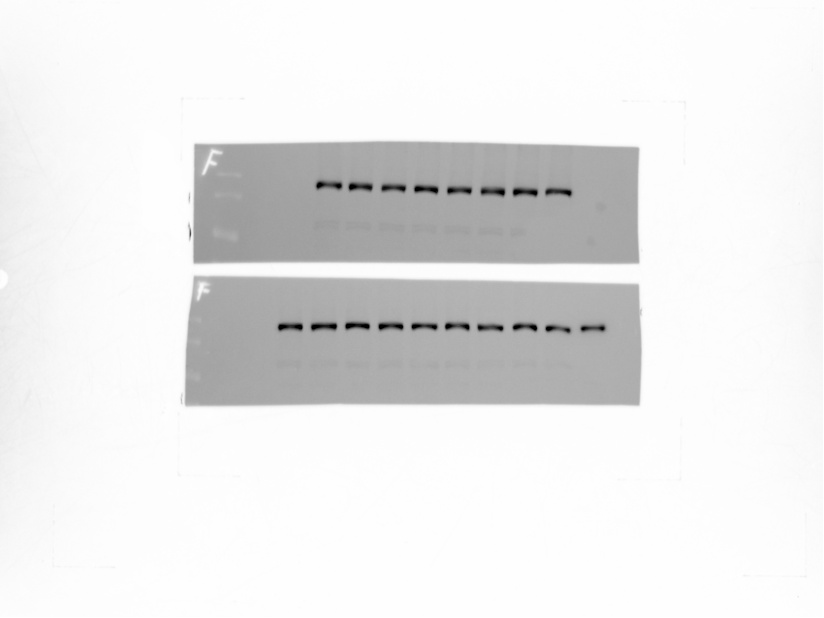

Supplement: Figure 2—source data 1. [file elife-92236-fig2-data1.zip › Figure_2-source_data_1/Figure_2-source_data_1_ Figure_2G_WCL_FLAG(left panel).jpg]

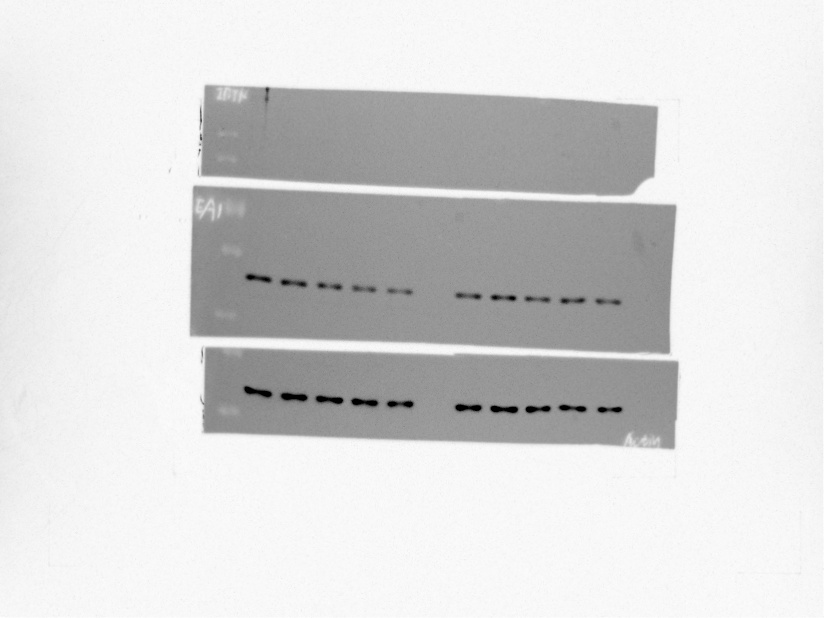

Supplement: Figure 2—figure supplement 1—source data 1. [file elife-92236-fig2-figsupp1-data1.zip › Figure_2-Figure Supplement_1-source_data_1/Figure_2-figure supplement_1_ source_data_1_ Figure_D_Actin.jpg]

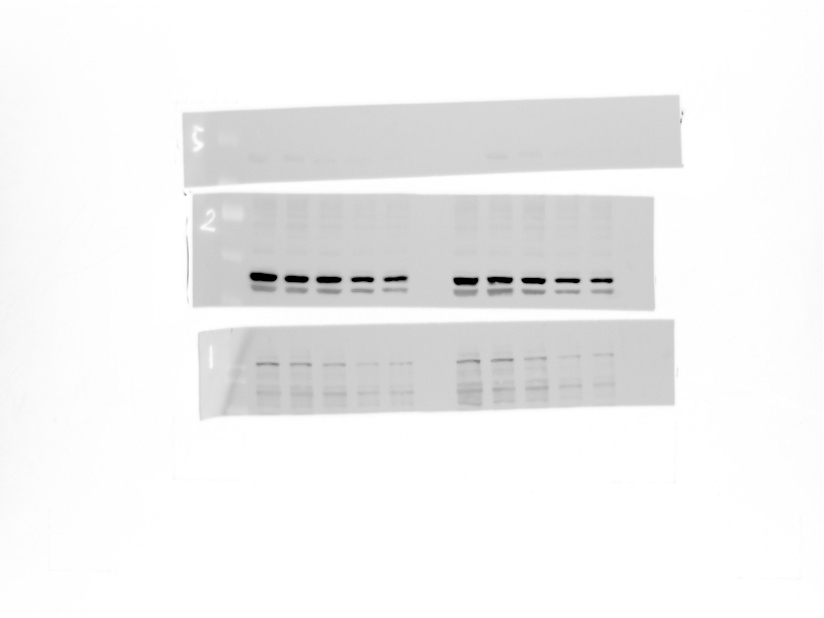

Supplement: Figure 2—figure supplement 1—source data 1. [file elife-92236-fig2-figsupp1-data1.zip › Figure_2-Figure Supplement_1-source_data_1/Figure_2-figure supplement_1_ source_data_1_ Figure_D_eIF4A1.jpg]

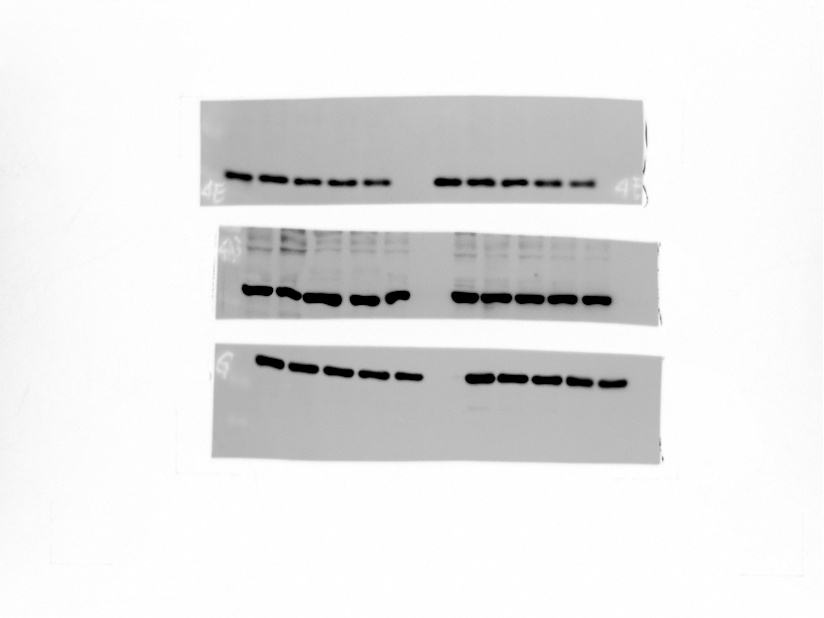

Supplement: Figure 2—figure supplement 1—source data 1. [file elife-92236-fig2-figsupp1-data1.zip › Figure_2-Figure Supplement_1-source_data_1/Figure_2-figure supplement_1_ source_data_1_ Figure_D_eIF4A2.jpg]

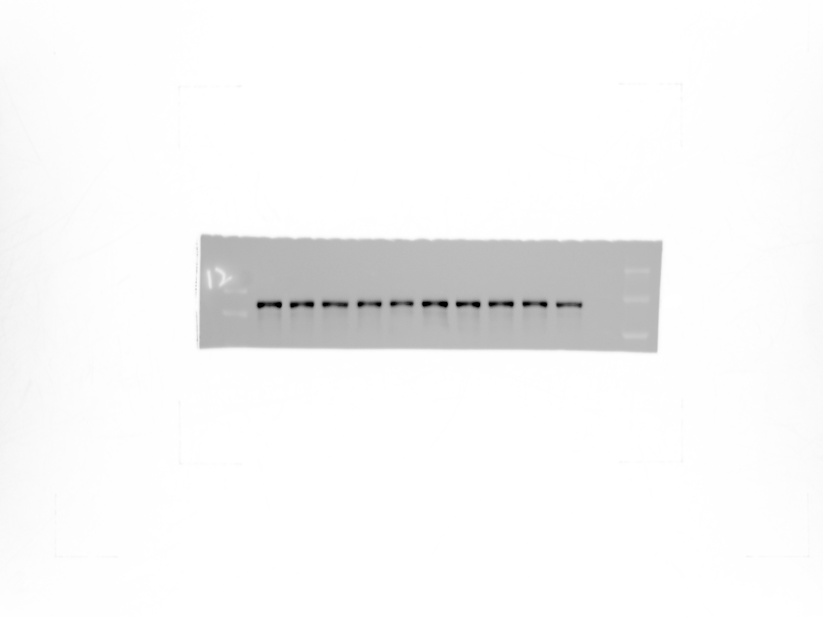

Supplement: Figure 2—figure supplement 1—source data 1. [file elife-92236-fig2-figsupp1-data1.zip › Figure_2-Figure Supplement_1-source_data_1/Figure_2-figure supplement_1_ source_data_1_ Figure_D_eIF4A3.jpg]

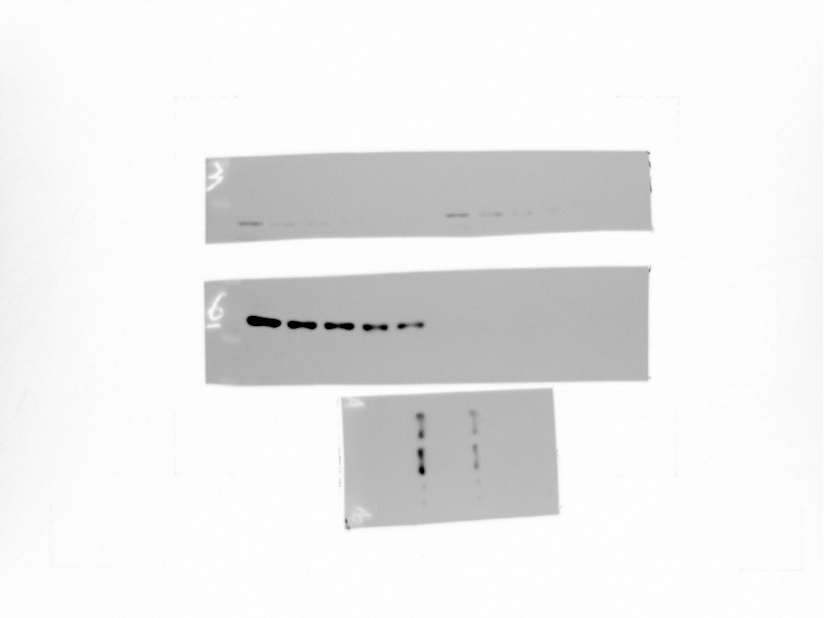

Supplement: Figure 2—figure supplement 1—source data 1. [file elife-92236-fig2-figsupp1-data1.zip › Figure_2-Figure Supplement_1-source_data_1/Figure_2-figure supplement_1_ source_data_1_ Figure_D_IBTK.jpg]

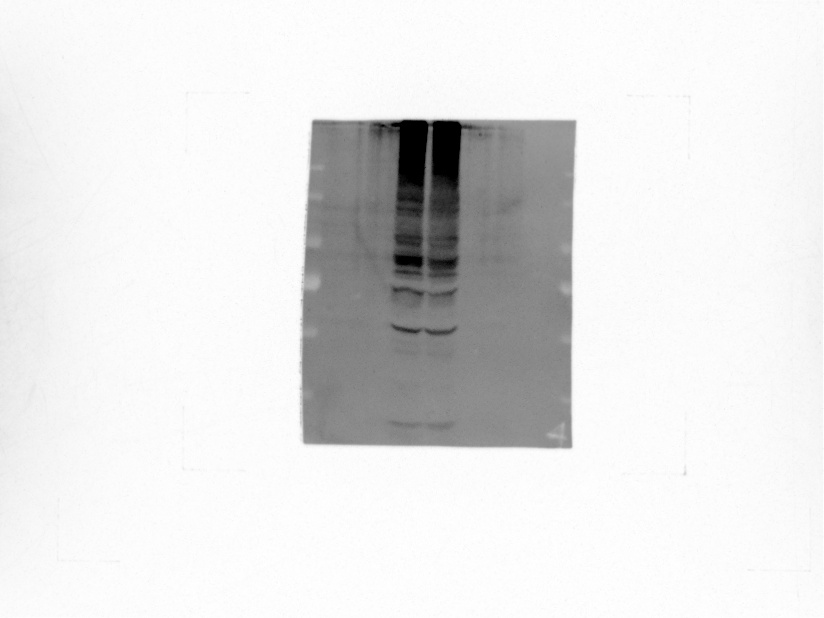

Supplement: Figure 2—figure supplement 1—source data 1. [file elife-92236-fig2-figsupp1-data1.zip › Figure_2-Figure Supplement_1-source_data_1/Figure_2-figure supplement_1_ source_data_1_ Figure_G_IP_HA.jpg]

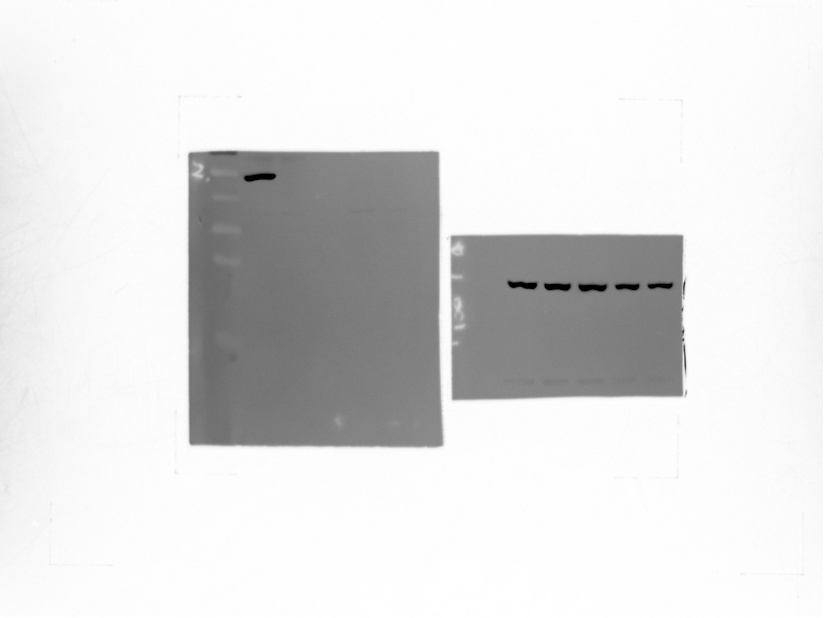

Supplement: Figure 2—figure supplement 1—source data 1. [file elife-92236-fig2-figsupp1-data1.zip › Figure_2-Figure Supplement_1-source_data_1/Figure_2-figure supplement_1_ source_data_1_ Figure_G_IP_PA.jpg]

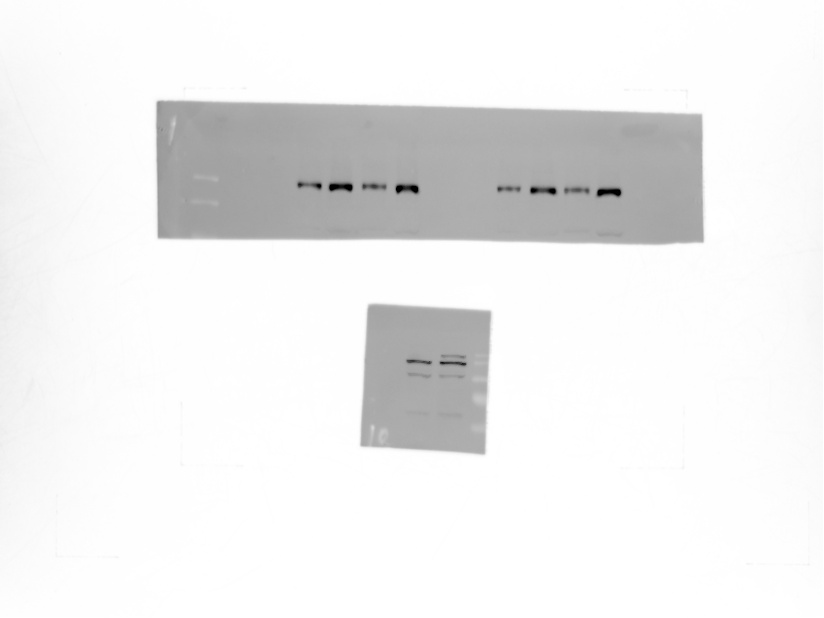

Supplement: Figure 2—figure supplement 1—source data 1. [file elife-92236-fig2-figsupp1-data1.zip › Figure_2-Figure Supplement_1-source_data_1/Figure_2-figure supplement_1_ source_data_1_ Figure_G_WCL_FLAG.jpg]

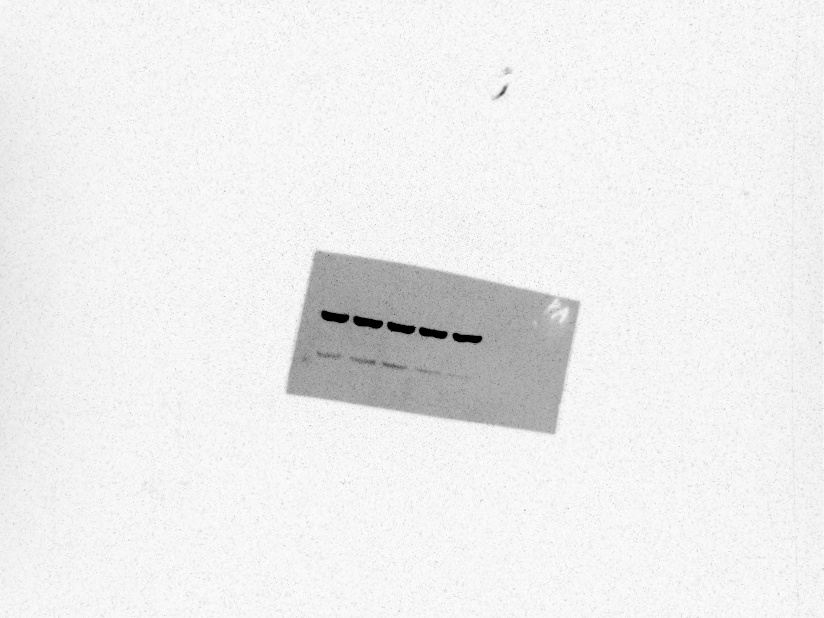

Supplement: Figure 2—figure supplement 1—source data 1. [file elife-92236-fig2-figsupp1-data1.zip › Figure_2-Figure Supplement_1-source_data_1/Figure_2-figure supplement_1_ source_data_1_ Figure_G_WCL_PA.jpg]

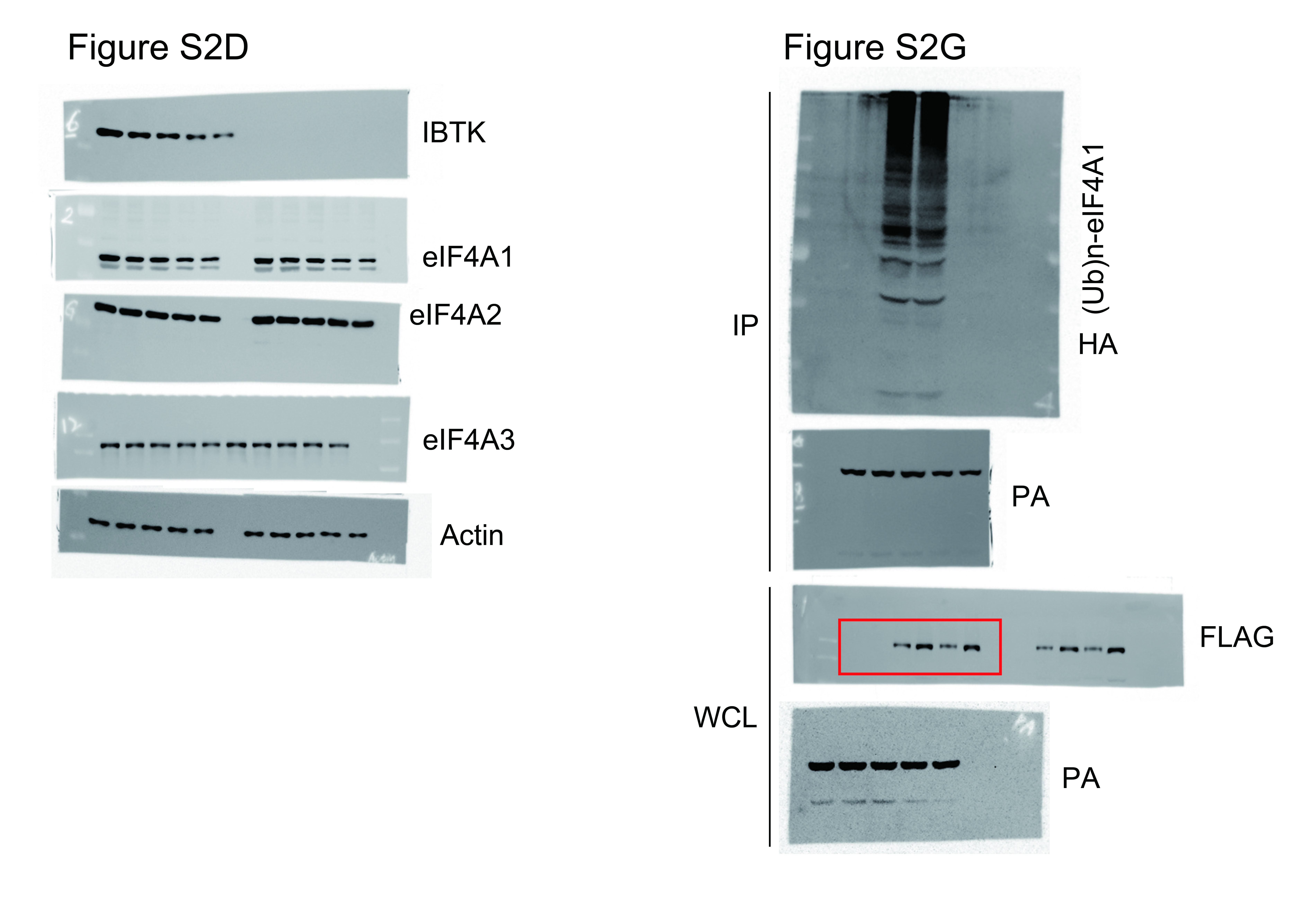

Supplement: Figure 2—figure supplement 1—source data 2. [file elife-92236-fig2-figsupp1-data2.zip › Figure_2-Figure Supplement_1-source_data_2.jpg]

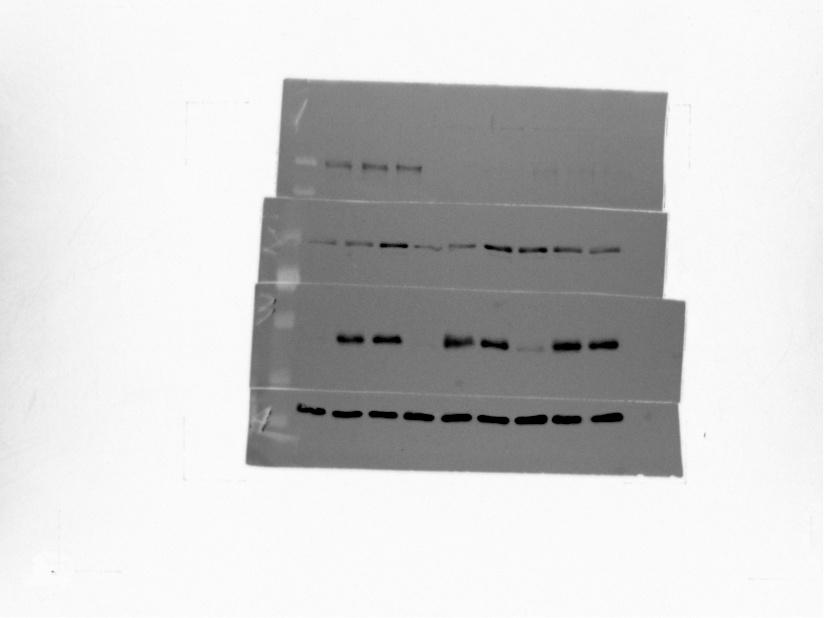

Supplement: Figure 3—source data 1. [file elife-92236-fig3-data1.zip › Figure_3-source_data_1/Figure3-source_data_1_ Figure_3A_GAPDH.jpg]

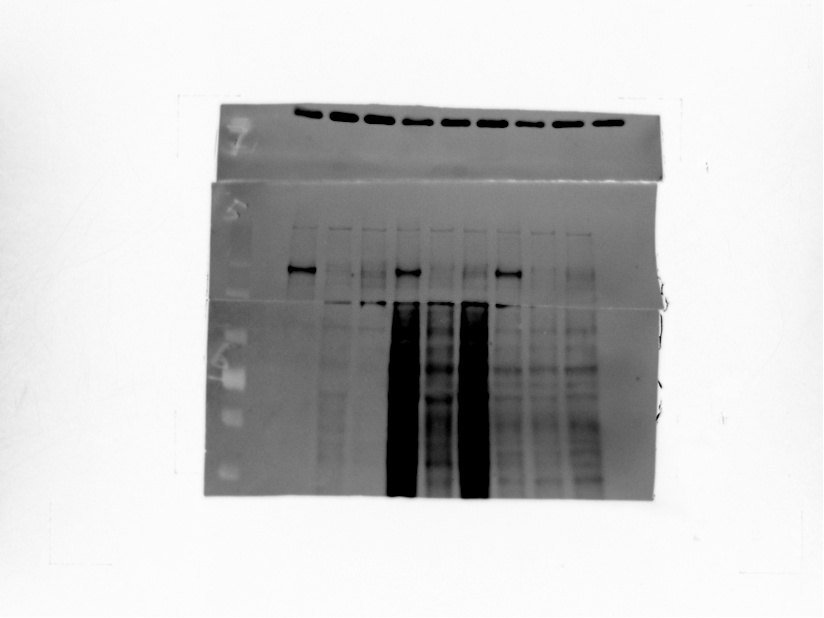

Supplement: Figure 3—source data 1. [file elife-92236-fig3-data1.zip › Figure_3-source_data_1/Figure3-source_data_1_ Figure_3A_IBTK.jpg]

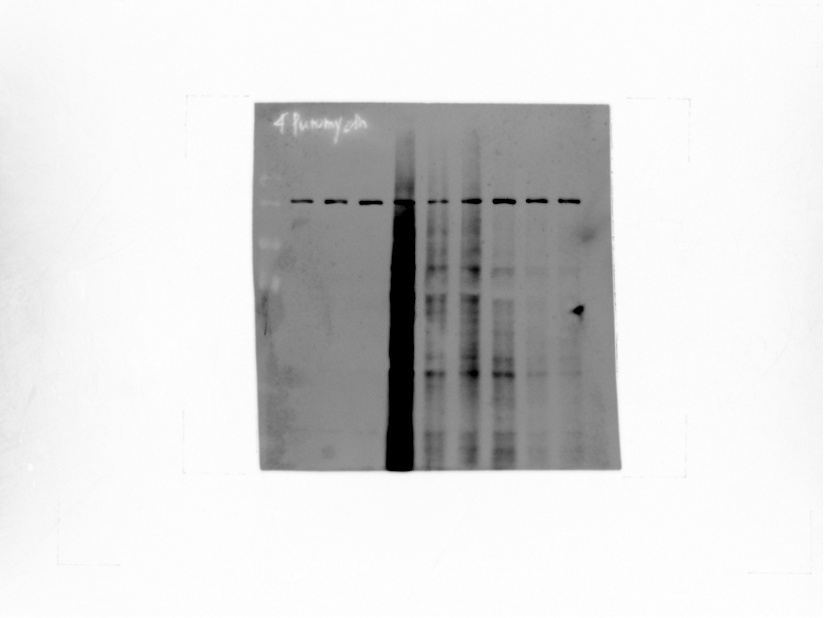

Supplement: Figure 3—source data 1. [file elife-92236-fig3-data1.zip › Figure_3-source_data_1/Figure3-source_data_1_ Figure_3A_Puromycin.jpg]

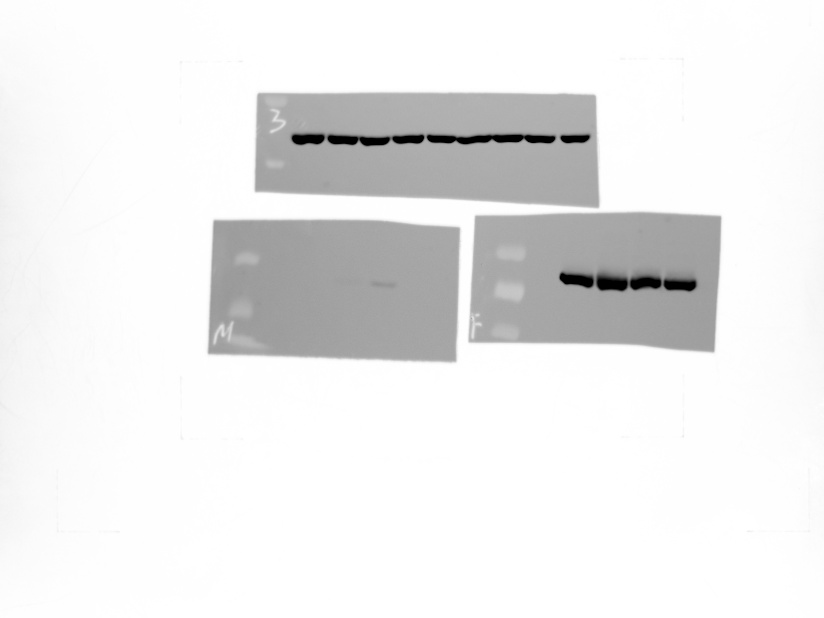

Supplement: Figure 3—source data 1. [file elife-92236-fig3-data1.zip › Figure_3-source_data_1/Figure3-source_data_1_ Figure_3B_Actin.jpg]

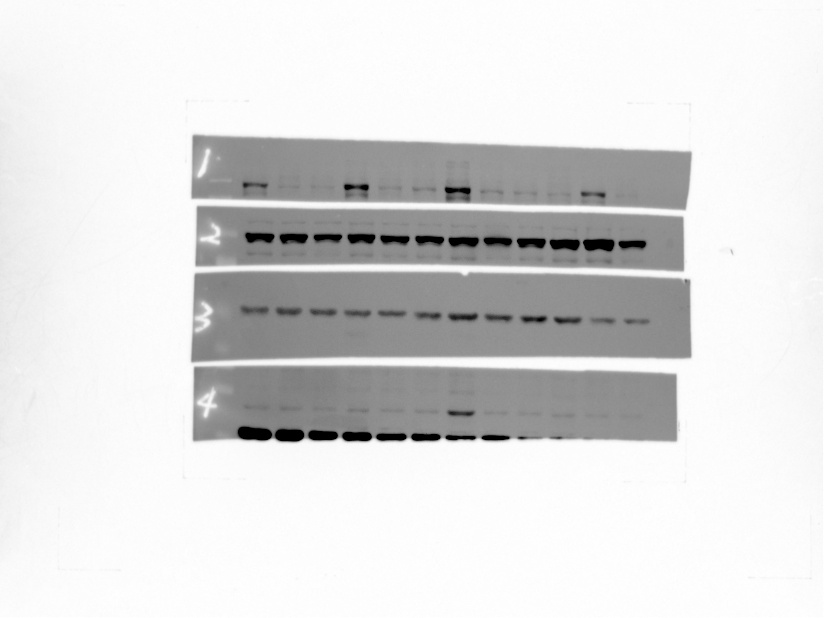

Supplement: Figure 3—source data 1. [file elife-92236-fig3-data1.zip › Figure_3-source_data_1/Figure3-source_data_1_ Figure_3B_IBTK.jpg]

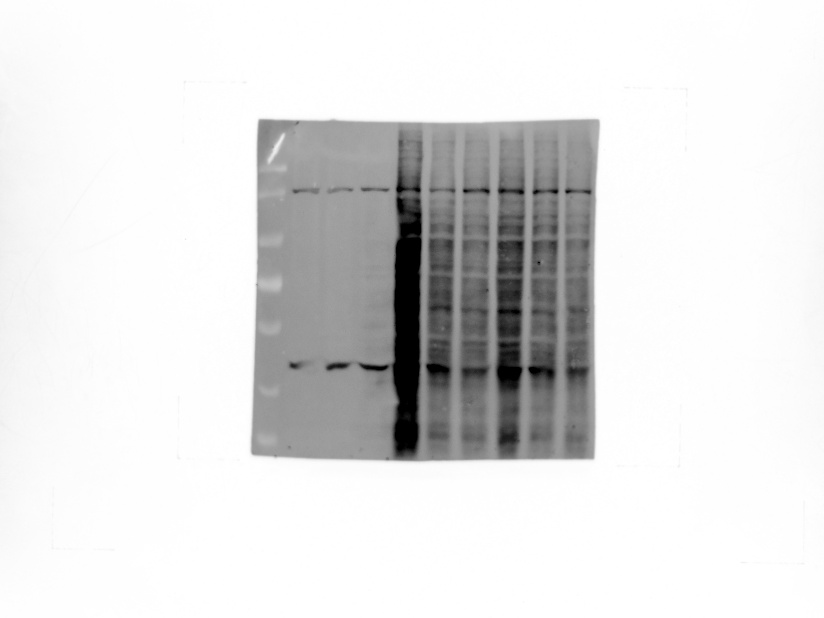

Supplement: Figure 3—source data 1. [file elife-92236-fig3-data1.zip › Figure_3-source_data_1/Figure3-source_data_1_ Figure_3B_Puromycin.jpg]

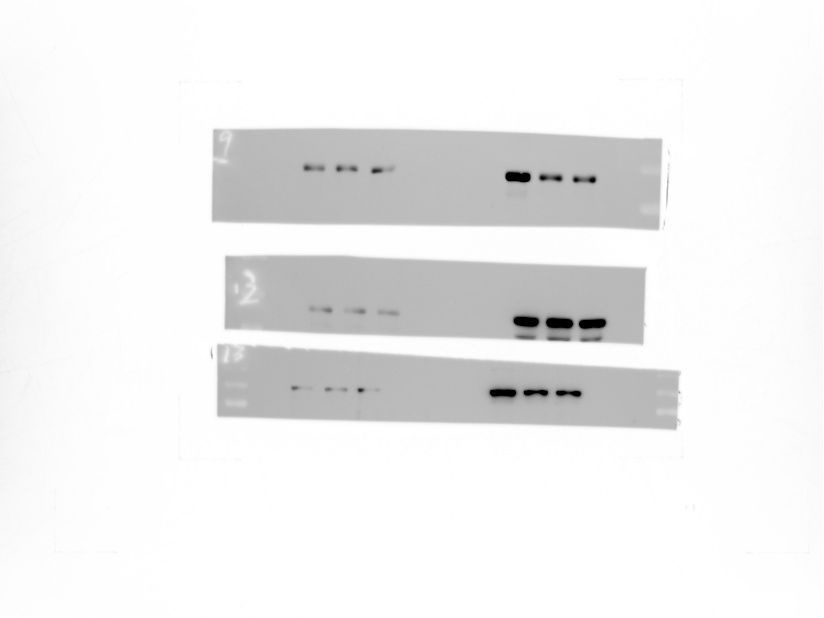

Supplement: Figure 3—source data 1. [file elife-92236-fig3-data1.zip › Figure_3-source_data_1/Figure3-source_data_1_ Figure_3H_eIF4A1.jpg]

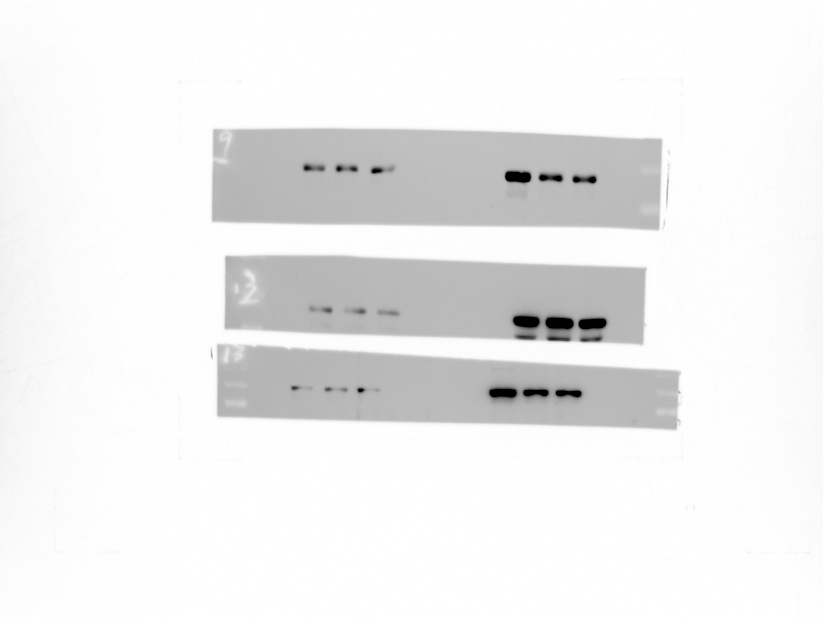

Supplement: Figure 3—source data 1. [file elife-92236-fig3-data1.zip › Figure_3-source_data_1/Figure3-source_data_1_ Figure_3H_eIF4B.jpg]

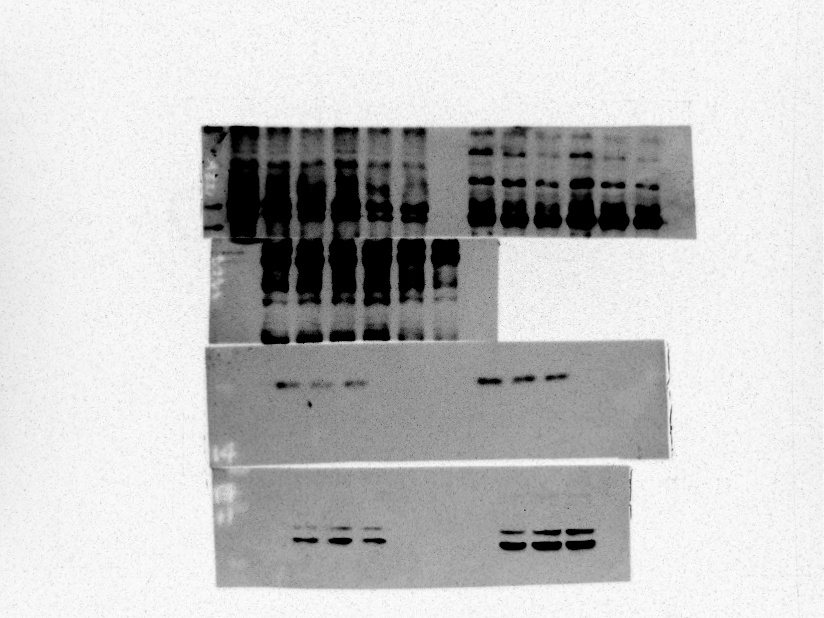

Supplement: Figure 3—source data 1. [file elife-92236-fig3-data1.zip › Figure_3-source_data_1/Figure3-source_data_1_ Figure_3H_eIF4E.jpg]

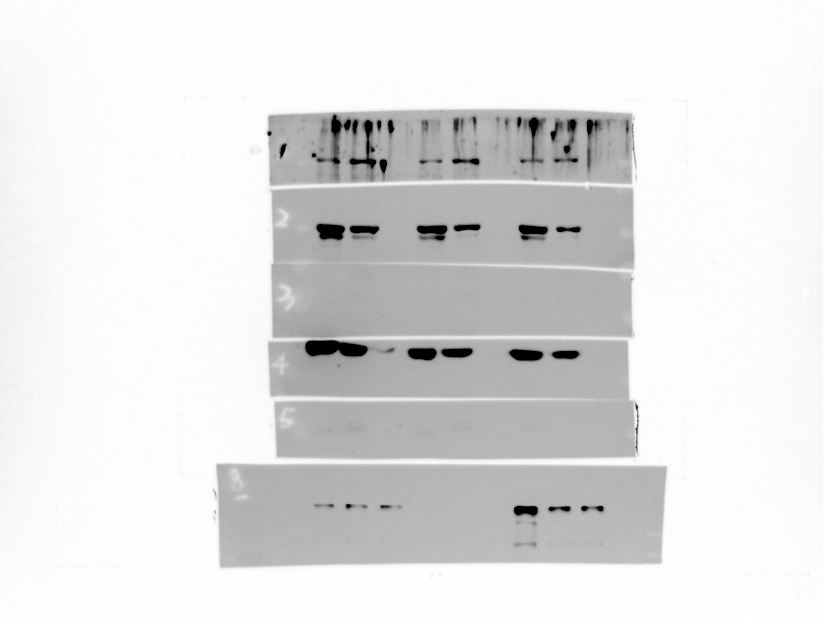

Supplement: Figure 3—source data 1. [file elife-92236-fig3-data1.zip › Figure_3-source_data_1/Figure3-source_data_1_ Figure_3H_eIF4G.jpg]

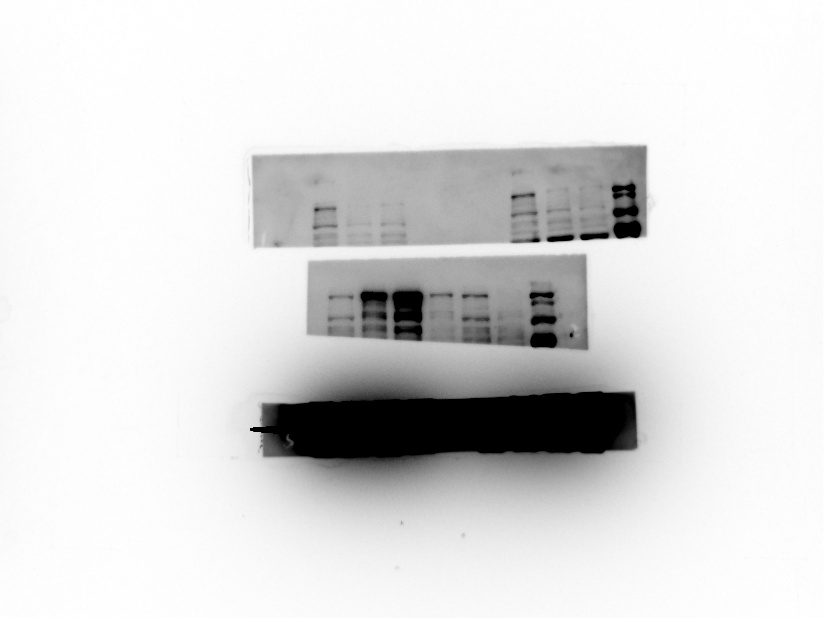

Supplement: Figure 3—source data 1. [file elife-92236-fig3-data1.zip › Figure_3-source_data_1/Figure3-source_data_1_ Figure_3H_IBTK.jpg]

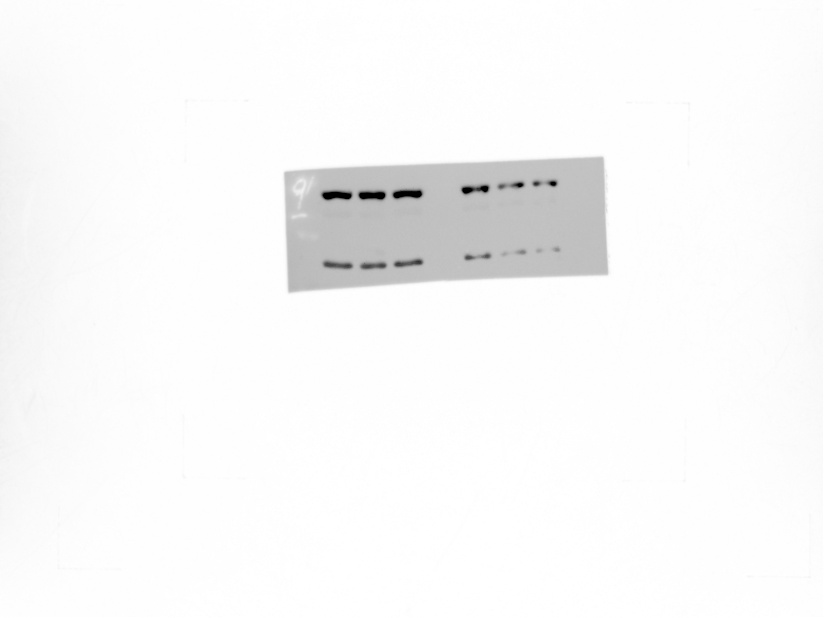

Supplement: Figure 3—source data 1. [file elife-92236-fig3-data1.zip › Figure_3-source_data_1/Figure3-source_data_1_ Figure_3I_eIF4A1.jpg]

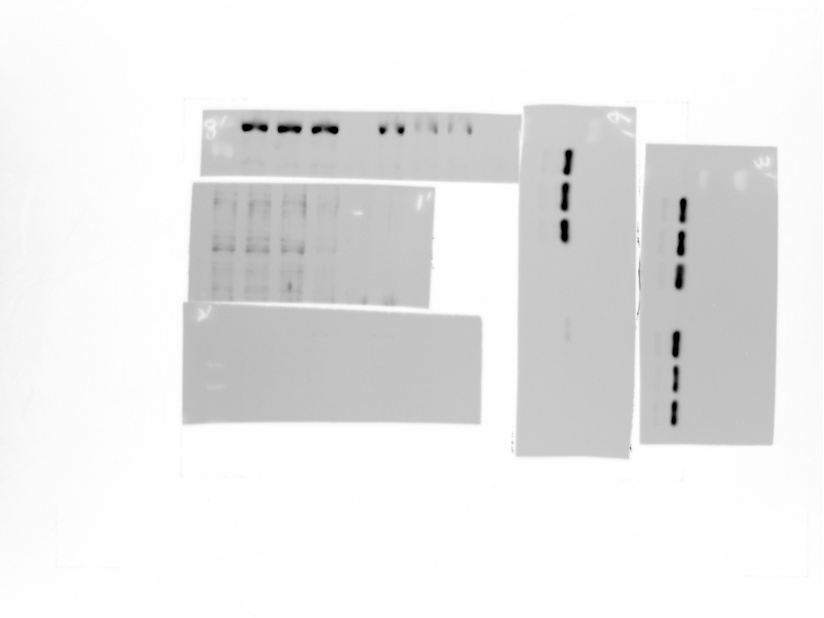

Supplement: Figure 3—source data 1. [file elife-92236-fig3-data1.zip › Figure_3-source_data_1/Figure3-source_data_1_ Figure_3I_eIF4B.jpg]

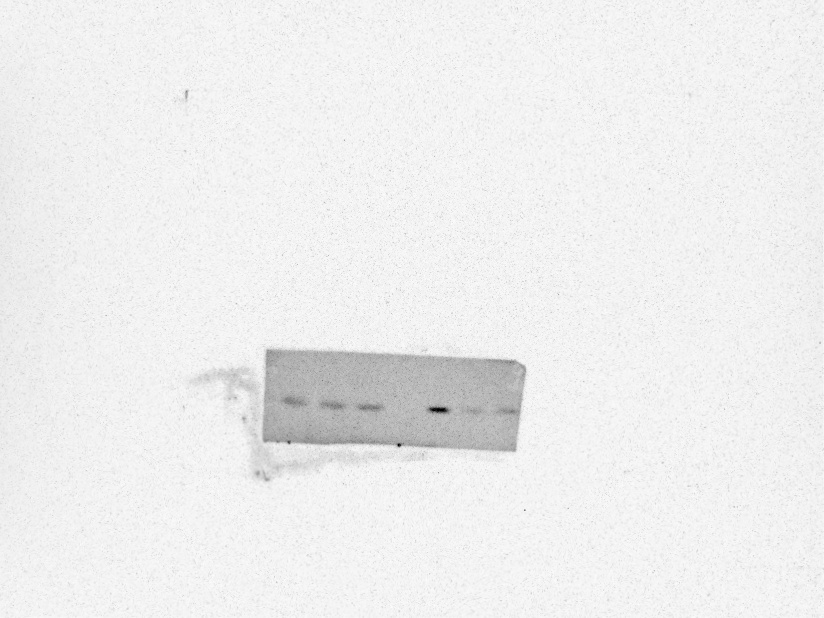

Supplement: Figure 3—source data 1. [file elife-92236-fig3-data1.zip › Figure_3-source_data_1/Figure3-source_data_1_ Figure_3I_eIF4E.jpg]

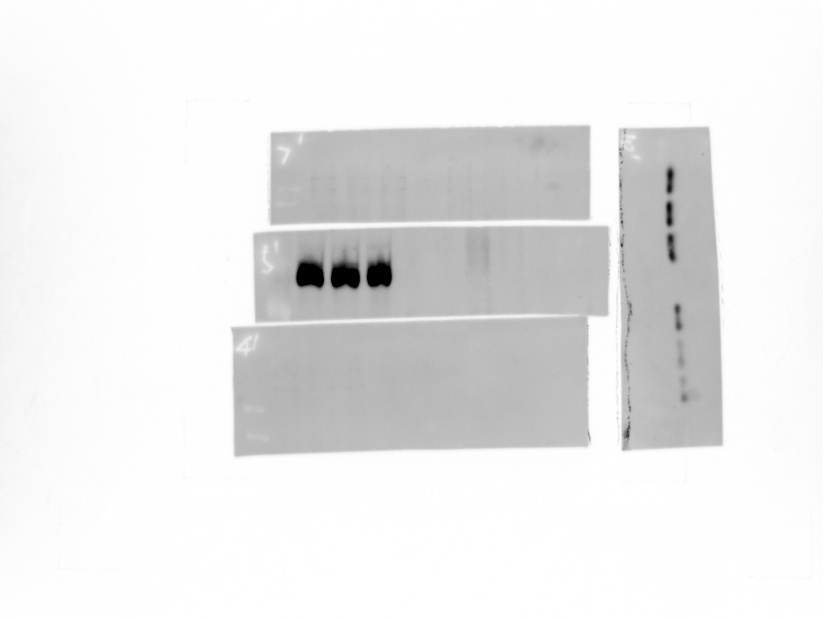

Supplement: Figure 3—source data 1. [file elife-92236-fig3-data1.zip › Figure_3-source_data_1/Figure3-source_data_1_ Figure_3I_eIF4G.jpg]

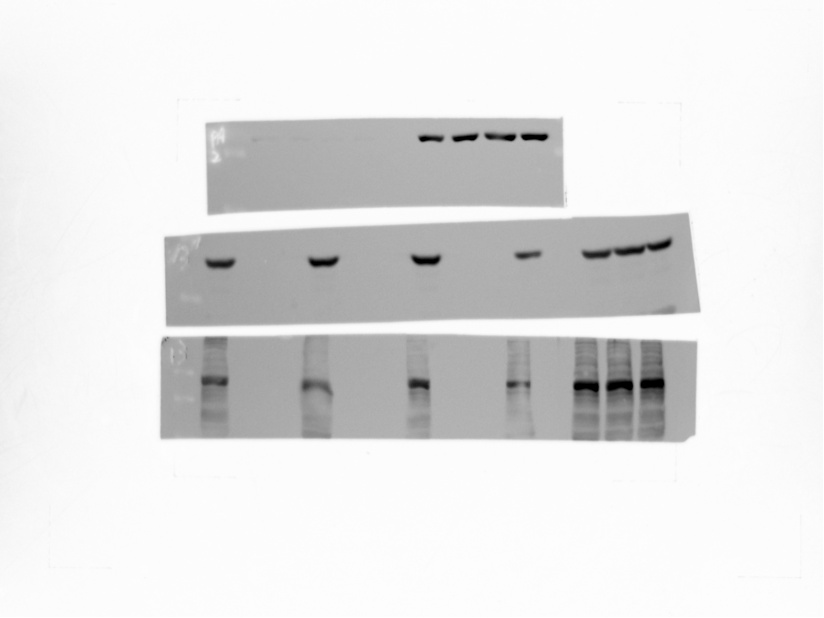

Supplement: Figure 3—source data 1. [file elife-92236-fig3-data1.zip › Figure_3-source_data_1/Figure3-source_data_1_ Figure_3I_IBTK.jpg]

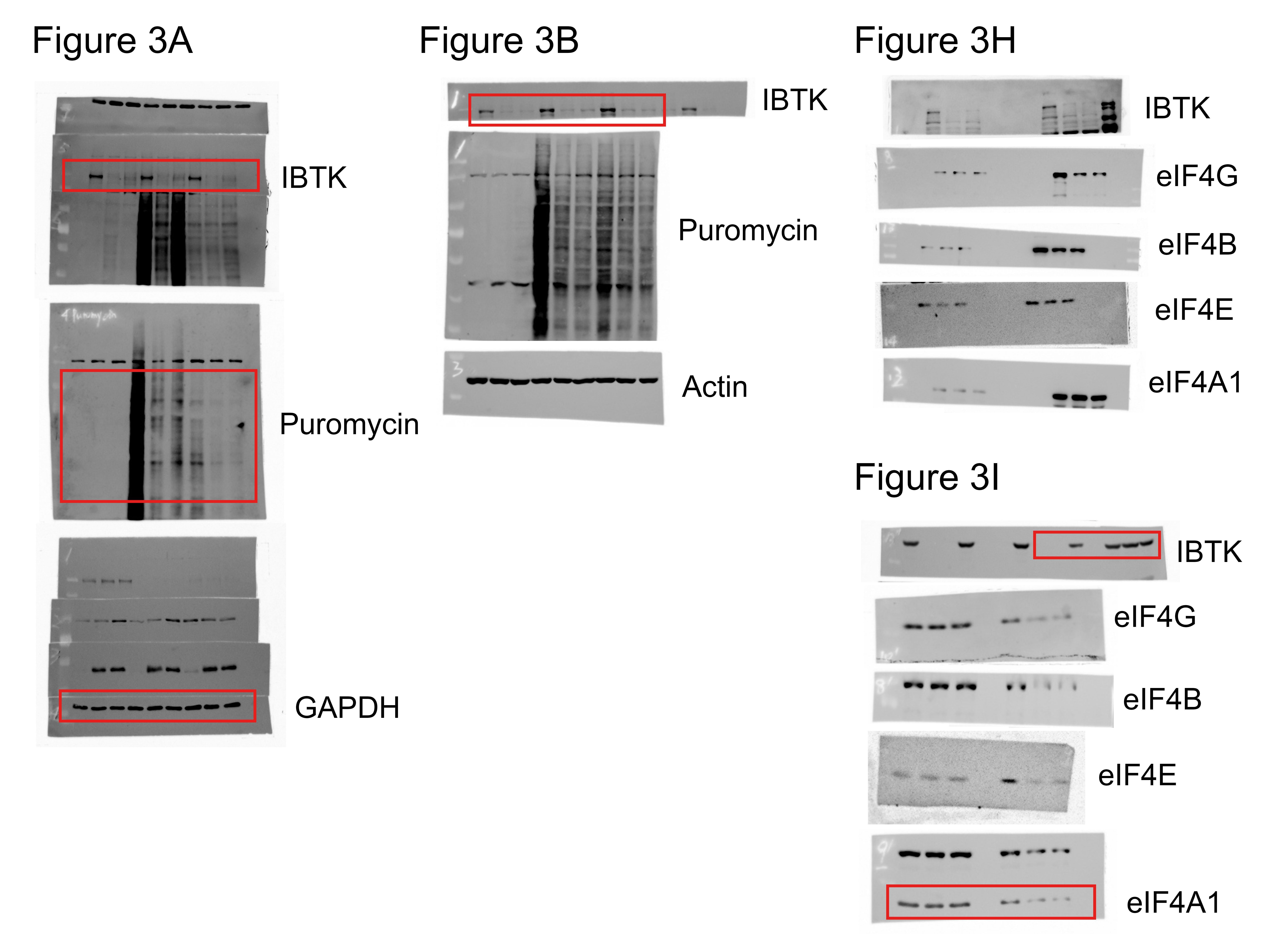

Supplement: Figure 3—source data 2. [file elife-92236-fig3-data2.zip › Figure_3-source_data_2.jpg]

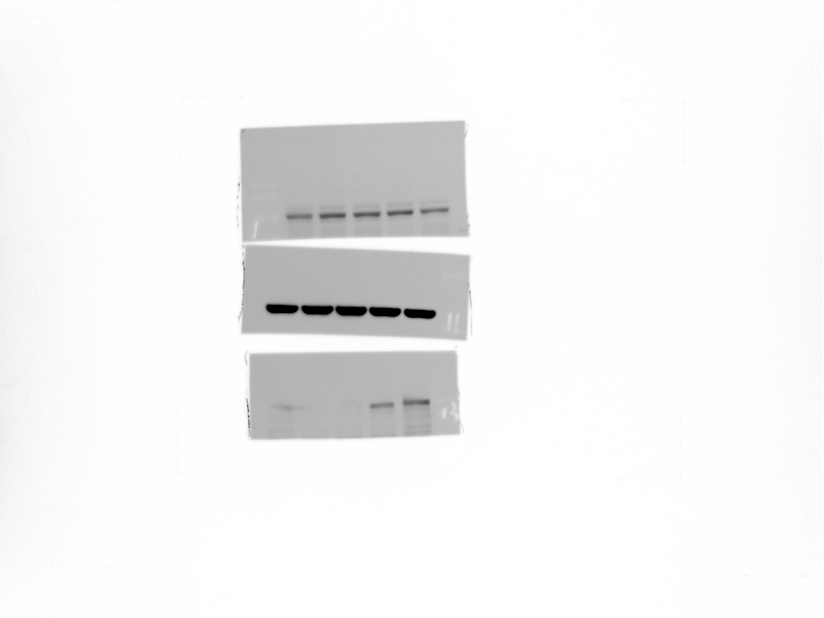

Supplement: Figure 3—figure supplement 1—source data 1. [file elife-92236-fig3-figsupp1-data1.zip › Figure_3-Figure Supplement_1-source_data_1/Figure_3-figure supplement_1_ source_data_1_ Figure_A_Actin.jpg]

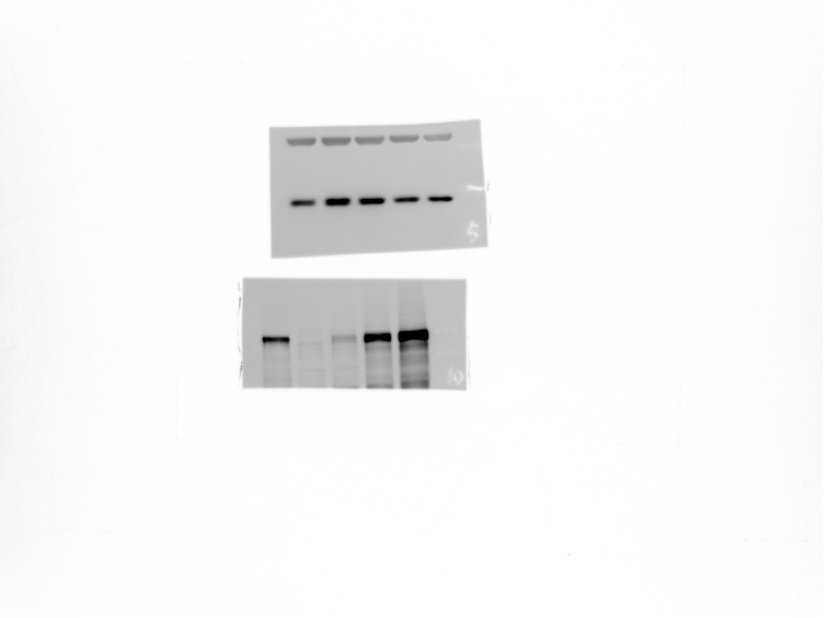

Supplement: Figure 3—figure supplement 1—source data 1. [file elife-92236-fig3-figsupp1-data1.zip › Figure_3-Figure Supplement_1-source_data_1/Figure_3-figure supplement_1_ source_data_1_ Figure_A_IBTK.jpg]
