# Supplementary figures and images for: mTORC1/S6K1 signaling promotes sustained oncogenic translation through modulating CRL3IBTK-mediated ubiquitination of eIF4A1 in cancer cells (part 2 of 3)
Source: eLife. 2024 May 13;12:RP92236. doi: 10.7554/eLife.92236 (PMC11090508; doi:10.7554/eLife.92236)

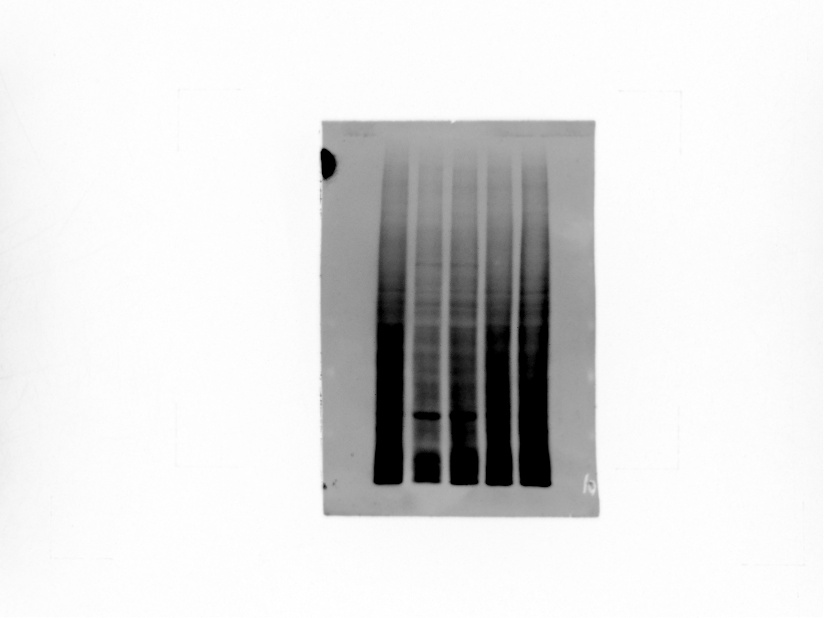

Supplement: Figure 3—figure supplement 1—source data 1. [file elife-92236-fig3-figsupp1-data1.zip › Figure_3-Figure Supplement_1-source_data_1/Figure_3-figure supplement_1_ source_data_1_ Figure_A_puromycin.jpg]

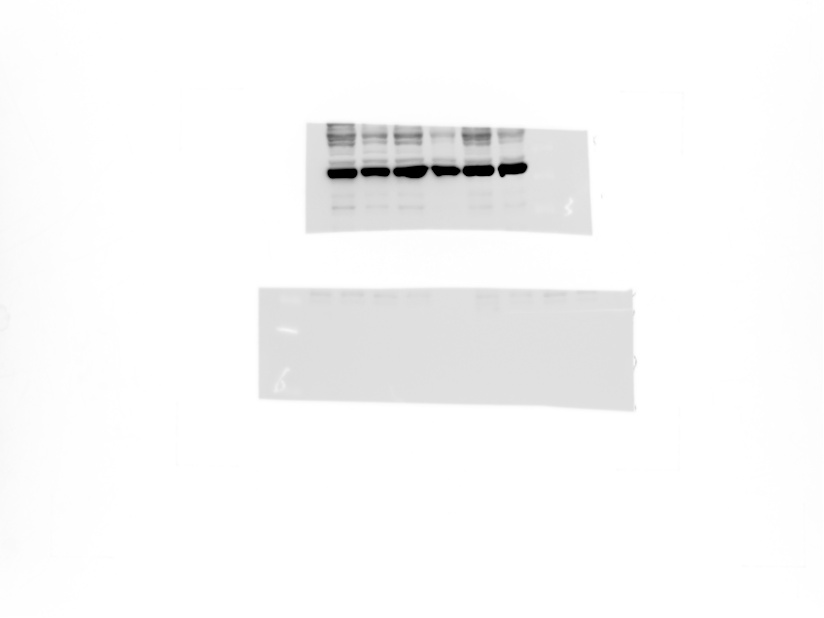

Supplement: Figure 3—figure supplement 1—source data 1. [file elife-92236-fig3-figsupp1-data1.zip › Figure_3-Figure Supplement_1-source_data_1/Figure_3-figure supplement_1_ source_data_1_ Figure_B_Actin.jpg]

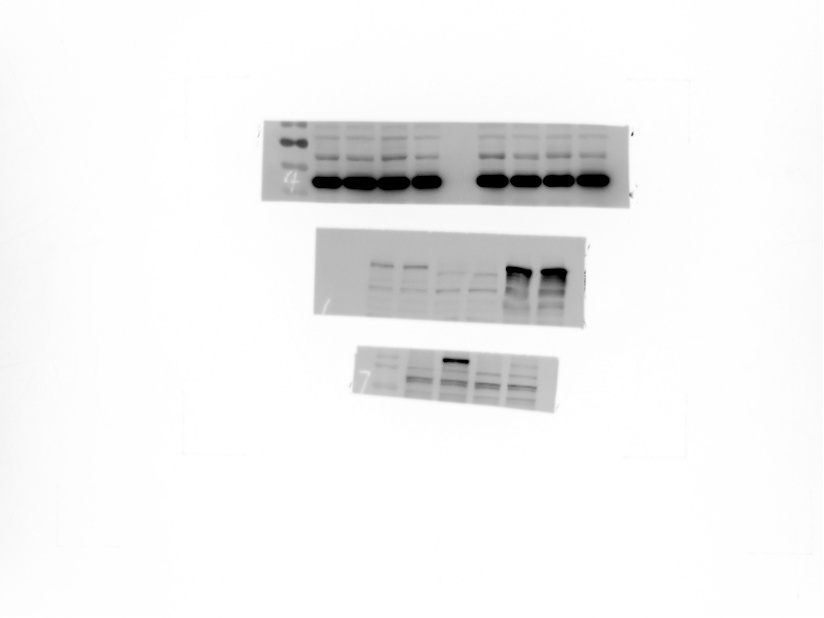

Supplement: Figure 3—figure supplement 1—source data 1. [file elife-92236-fig3-figsupp1-data1.zip › Figure_3-Figure Supplement_1-source_data_1/Figure_3-figure supplement_1_ source_data_1_ Figure_B_IBTK.jpg]

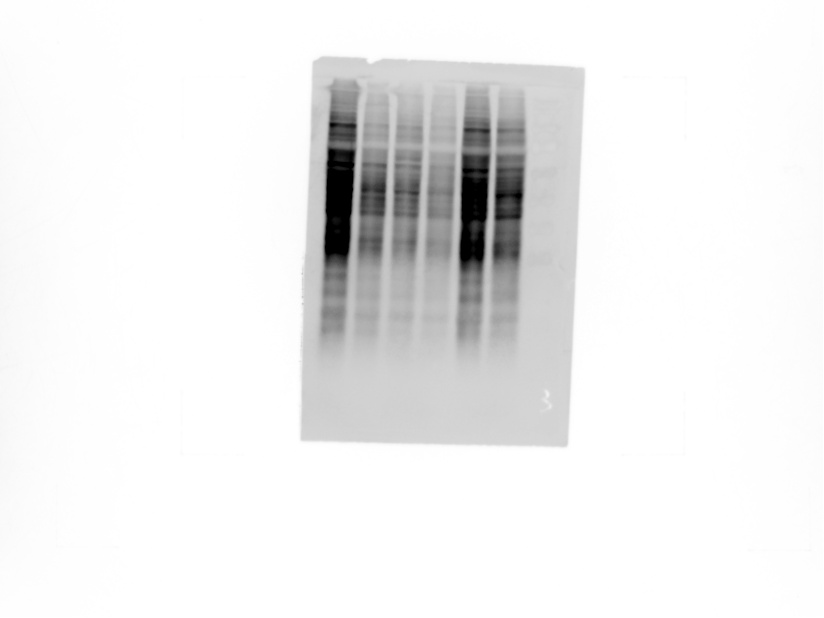

Supplement: Figure 3—figure supplement 1—source data 1. [file elife-92236-fig3-figsupp1-data1.zip › Figure_3-Figure Supplement_1-source_data_1/Figure_3-figure supplement_1_ source_data_1_ Figure_B_puromycin.jpg]

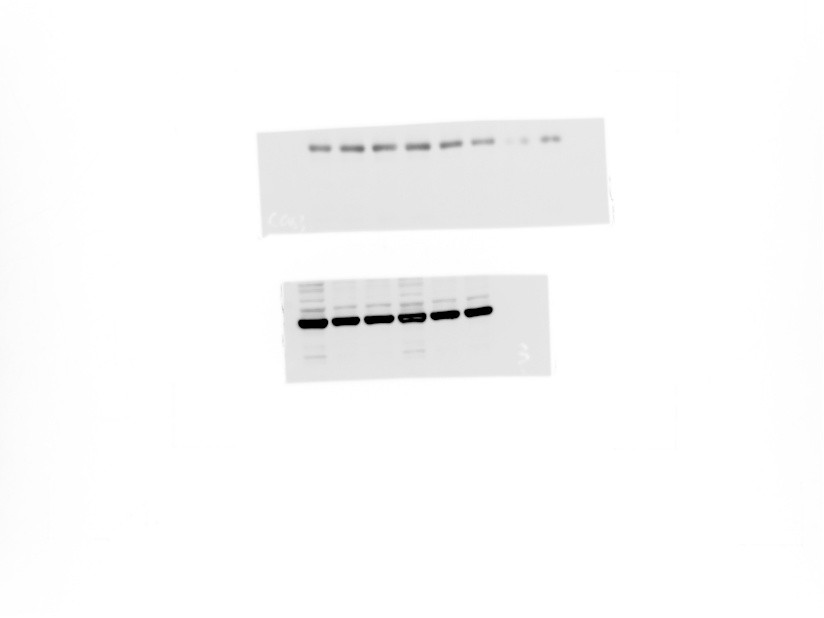

Supplement: Figure 3—figure supplement 1—source data 1. [file elife-92236-fig3-figsupp1-data1.zip › Figure_3-Figure Supplement_1-source_data_1/Figure_3-figure supplement_1_ source_data_1_ Figure_C_Actin.jpg]

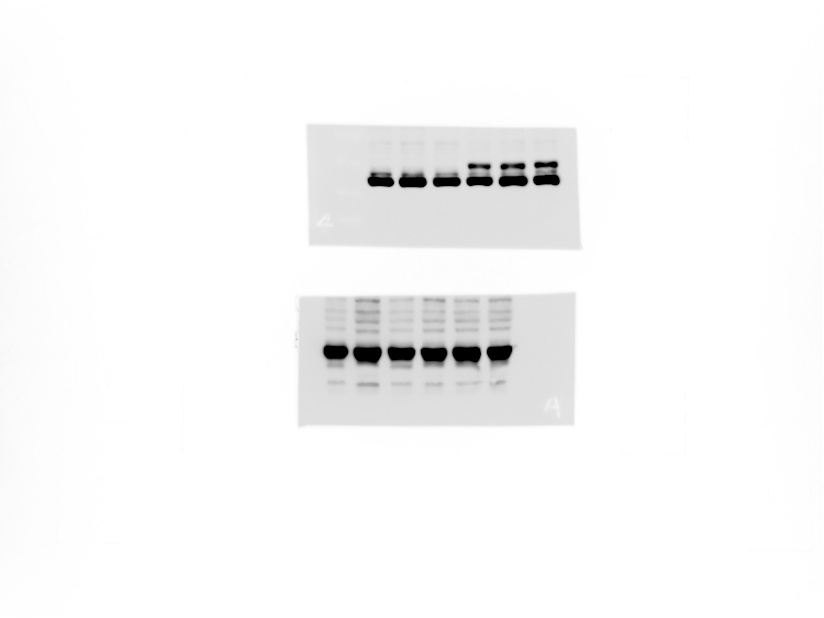

Supplement: Figure 3—figure supplement 1—source data 1. [file elife-92236-fig3-figsupp1-data1.zip › Figure_3-Figure Supplement_1-source_data_1/Figure_3-figure supplement_1_ source_data_1_ Figure_C_eIF4A1.jpg]

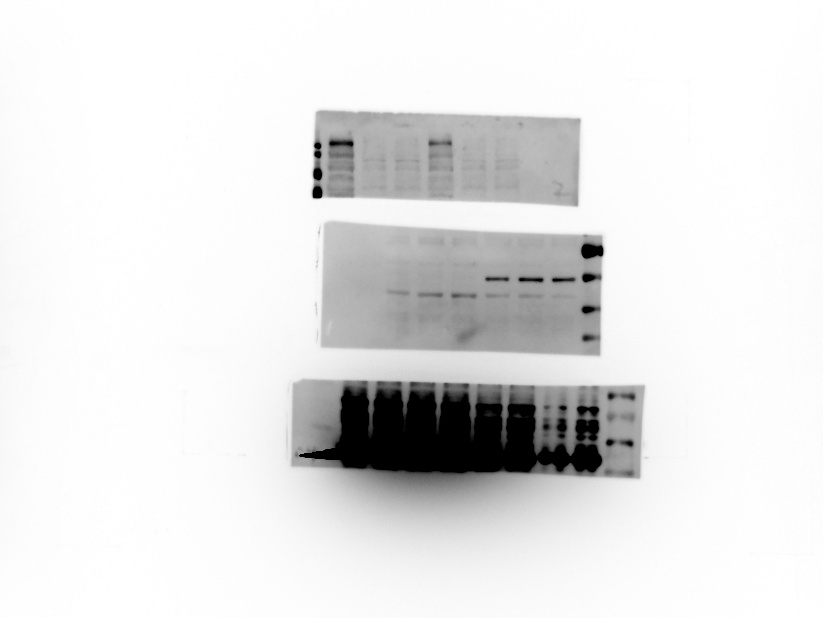

Supplement: Figure 3—figure supplement 1—source data 1. [file elife-92236-fig3-figsupp1-data1.zip › Figure_3-Figure Supplement_1-source_data_1/Figure_3-figure supplement_1_ source_data_1_ Figure_C_IBTK.jpg]

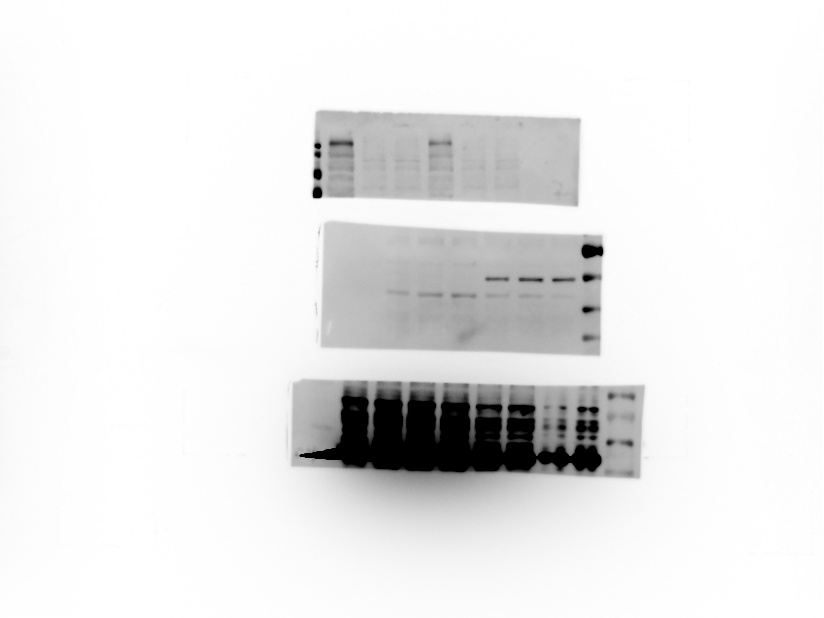

Supplement: Figure 3—figure supplement 1—source data 1. [file elife-92236-fig3-figsupp1-data1.zip › Figure_3-Figure Supplement_1-source_data_1/Figure_3-figure supplement_1_ source_data_1_ Figure_C_Myc.jpg]

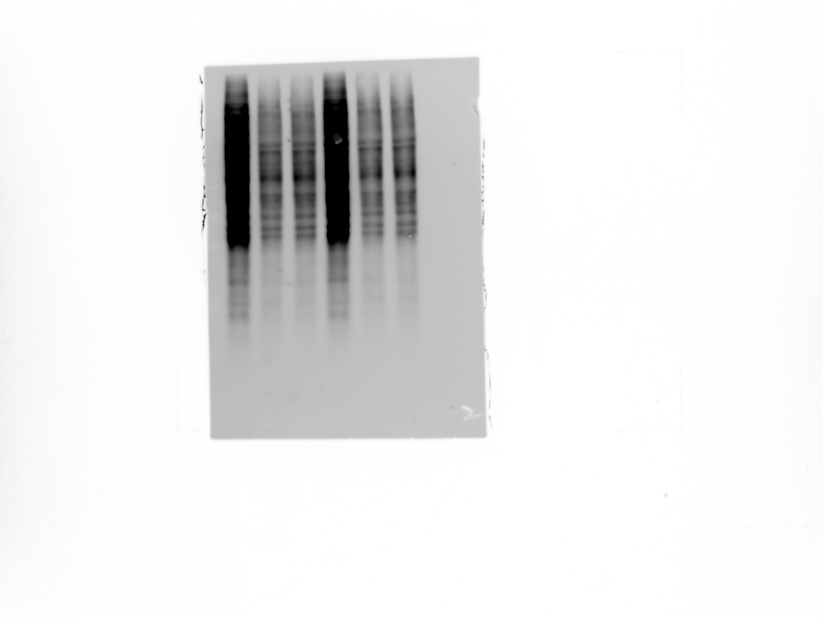

Supplement: Figure 3—figure supplement 1—source data 1. [file elife-92236-fig3-figsupp1-data1.zip › Figure_3-Figure Supplement_1-source_data_1/Figure_3-figure supplement_1_ source_data_1_ Figure_C_puromycin.jpg]

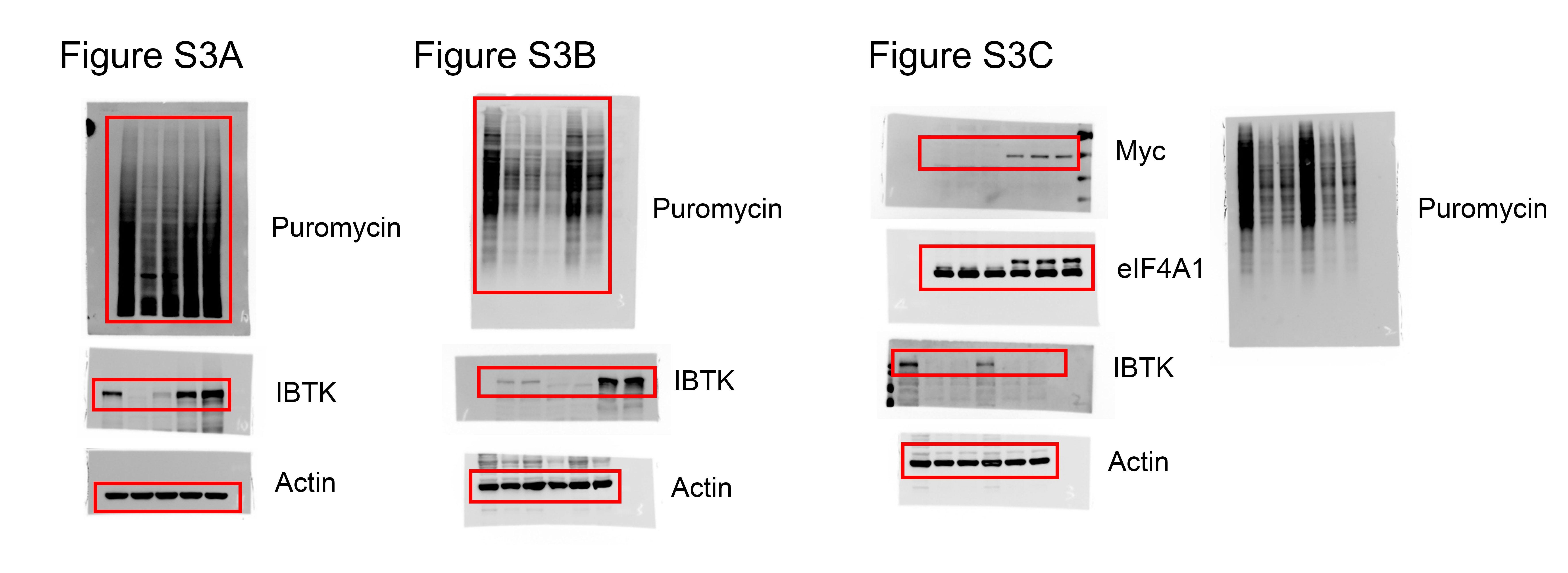

Supplement: Figure 3—figure supplement 1—source data 2. [file elife-92236-fig3-figsupp1-data2.zip › Figure_3-Figure supplement_1-source_data_2.jpg]

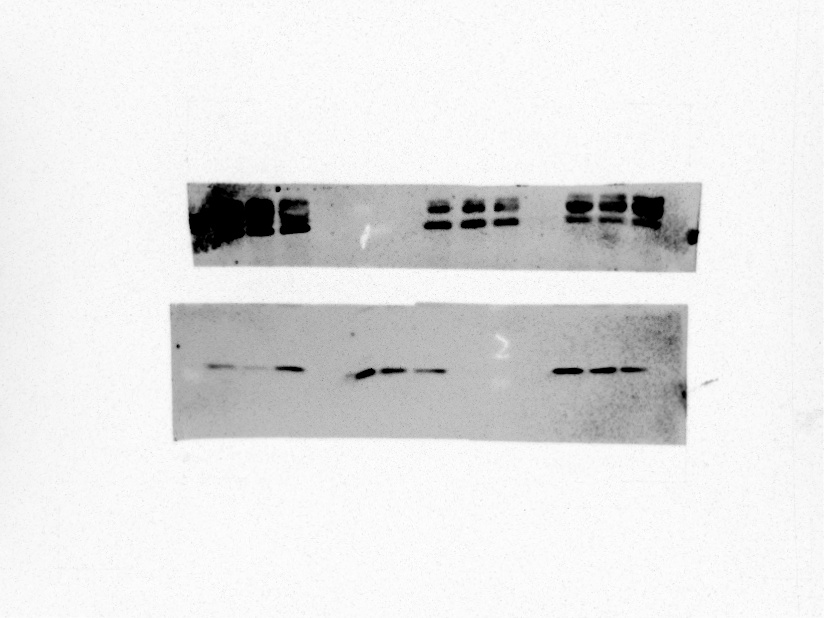

Supplement: Figure 4—source data 1. [file elife-92236-fig4-data1.zip › Figure_4-source_data_1/Figure_4-source_data_1_ Figure_4A_ARF6(left panel).jpg]

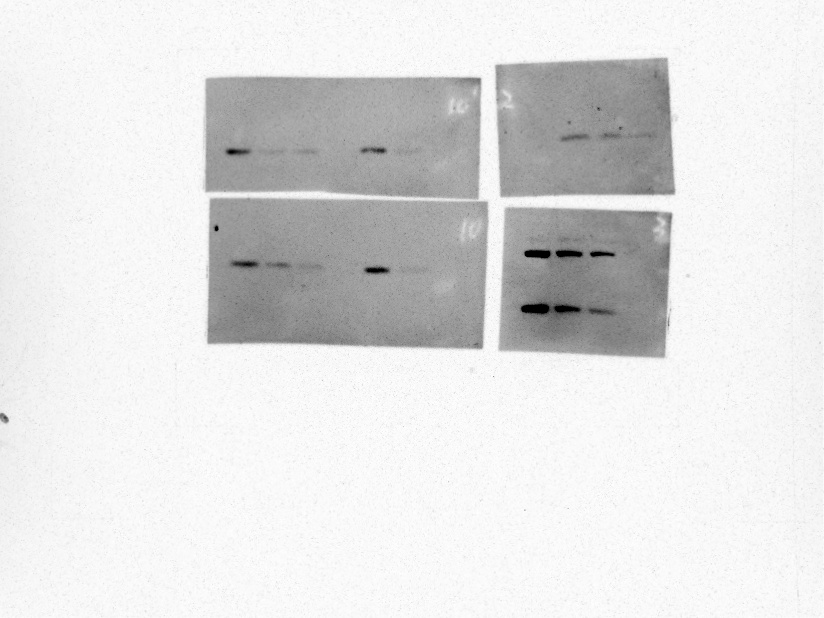

Supplement: Figure 4—source data 1. [file elife-92236-fig4-data1.zip › Figure_4-source_data_1/Figure_4-source_data_1_ Figure_4A_ARF6(right panel).jpg]

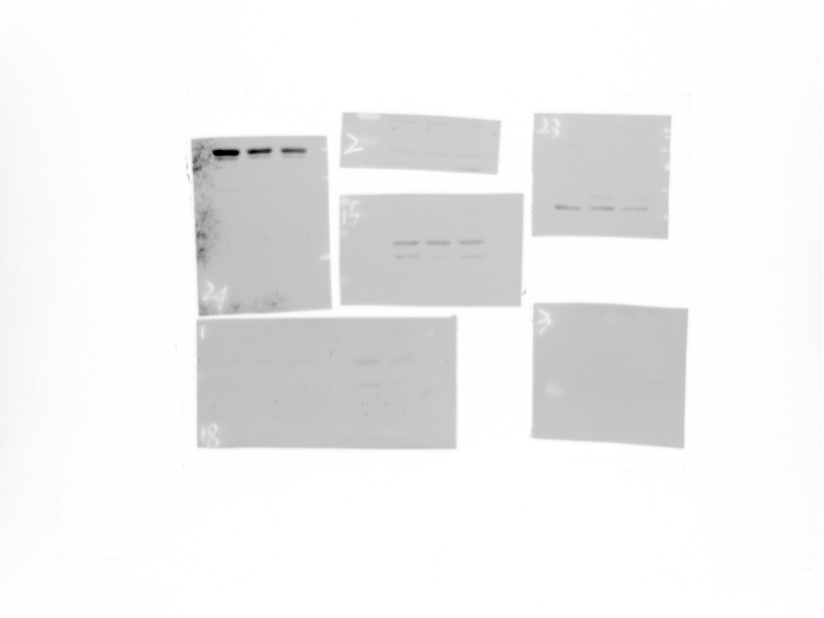

Supplement: Figure 4—source data 1. [file elife-92236-fig4-data1.zip › Figure_4-source_data_1/Figure_4-source_data_1_ Figure_4A_CCND3(left panel).jpg]

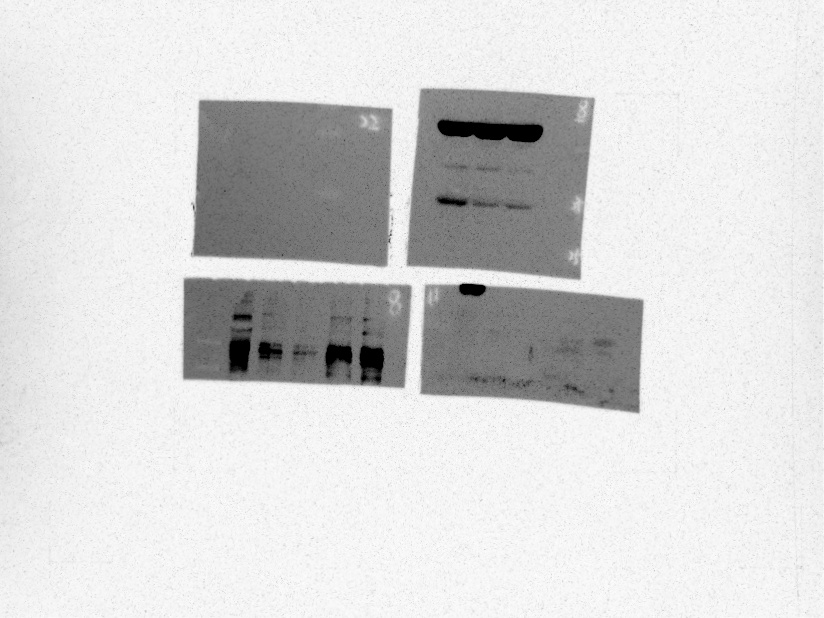

Supplement: Figure 4—source data 1. [file elife-92236-fig4-data1.zip › Figure_4-source_data_1/Figure_4-source_data_1_ Figure_4A_CCND3(right panel).jpg]

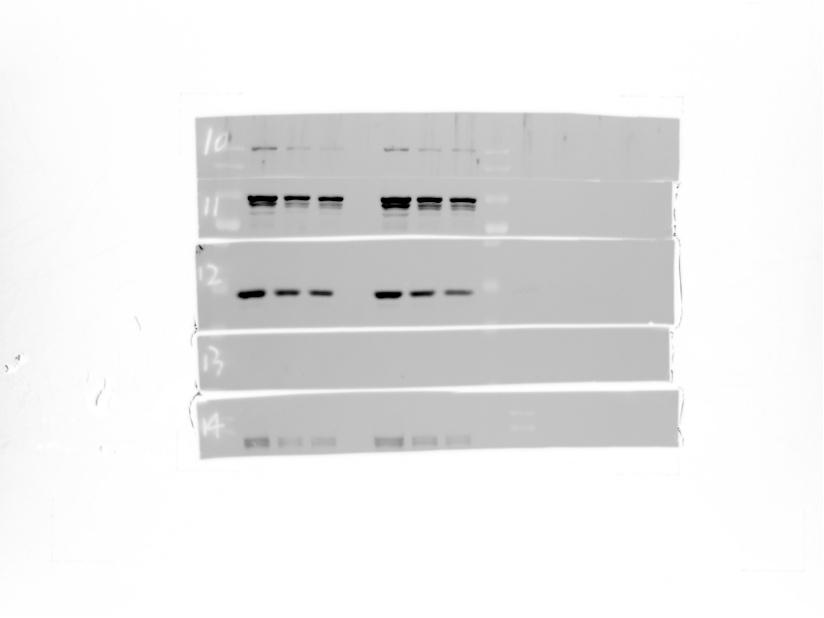

Supplement: Figure 4—source data 1. [file elife-92236-fig4-data1.zip › Figure_4-source_data_1/Figure_4-source_data_1_ Figure_4A_CDK4(left panel).jpg]

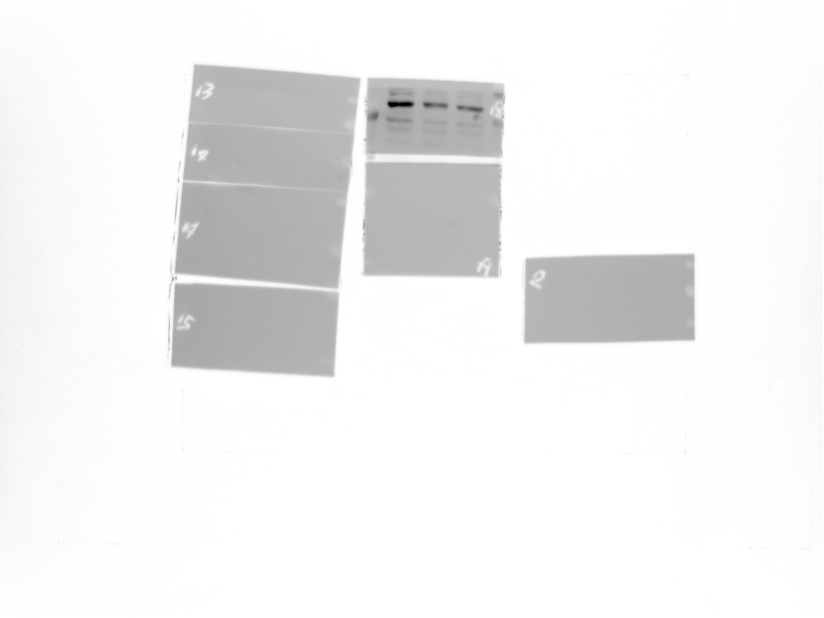

Supplement: Figure 4—source data 1. [file elife-92236-fig4-data1.zip › Figure_4-source_data_1/Figure_4-source_data_1_ Figure_4A_CDK4(right panel).jpg]

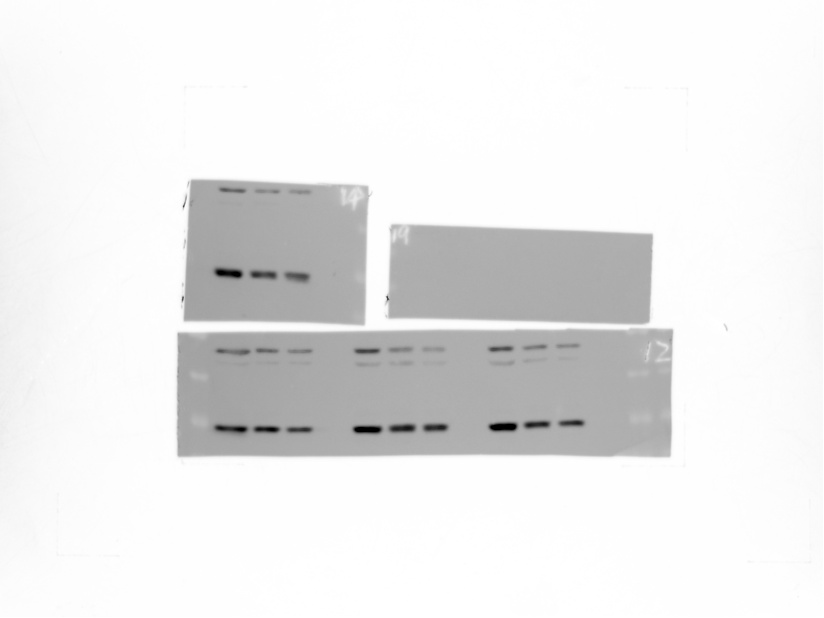

Supplement: Figure 4—source data 1. [file elife-92236-fig4-data1.zip › Figure_4-source_data_1/Figure_4-source_data_1_ Figure_4A_CDK6(left panel).jpg]

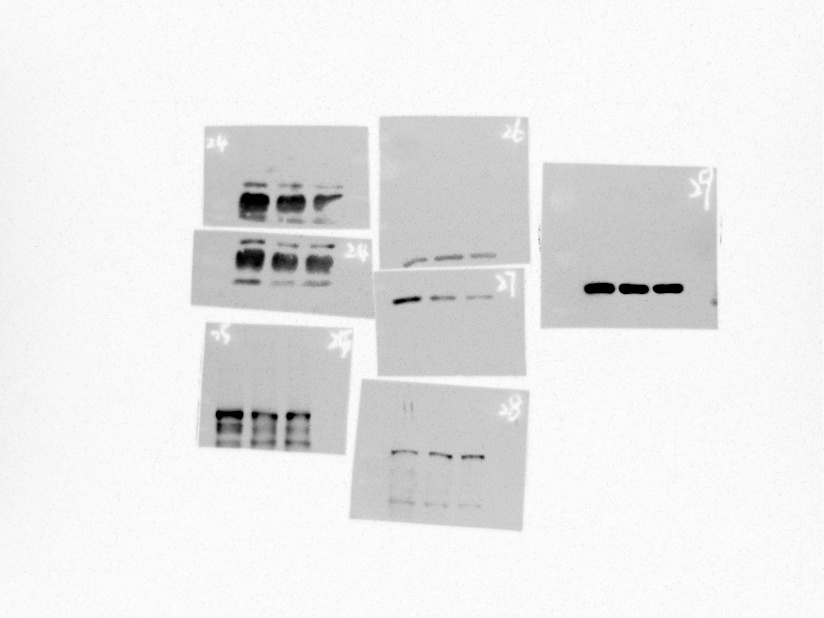

Supplement: Figure 4—source data 1. [file elife-92236-fig4-data1.zip › Figure_4-source_data_1/Figure_4-source_data_1_ Figure_4A_CDK6(right panel).jpg]

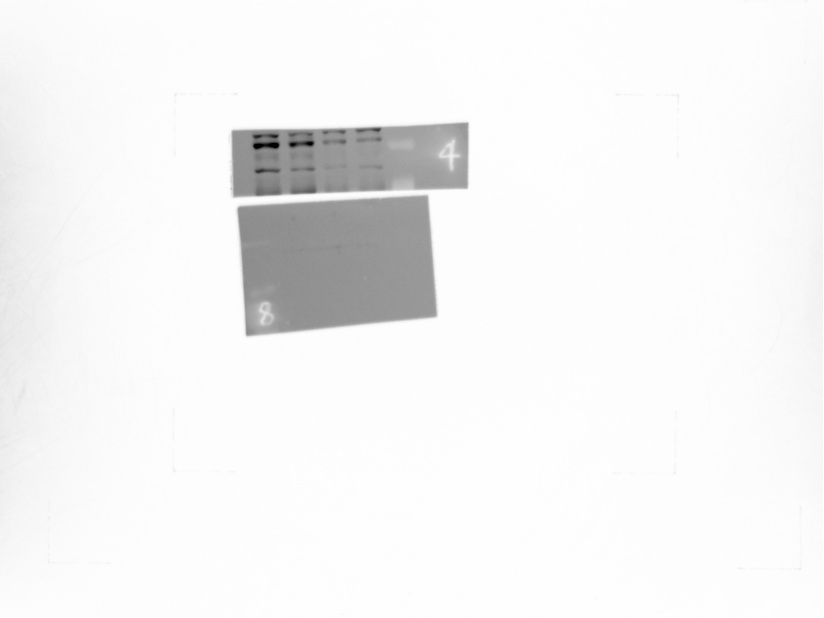

Supplement: Figure 4—source data 1. [file elife-92236-fig4-data1.zip › Figure_4-source_data_1/Figure_4-source_data_1_ Figure_4A_EZH2(left panel).jpg]

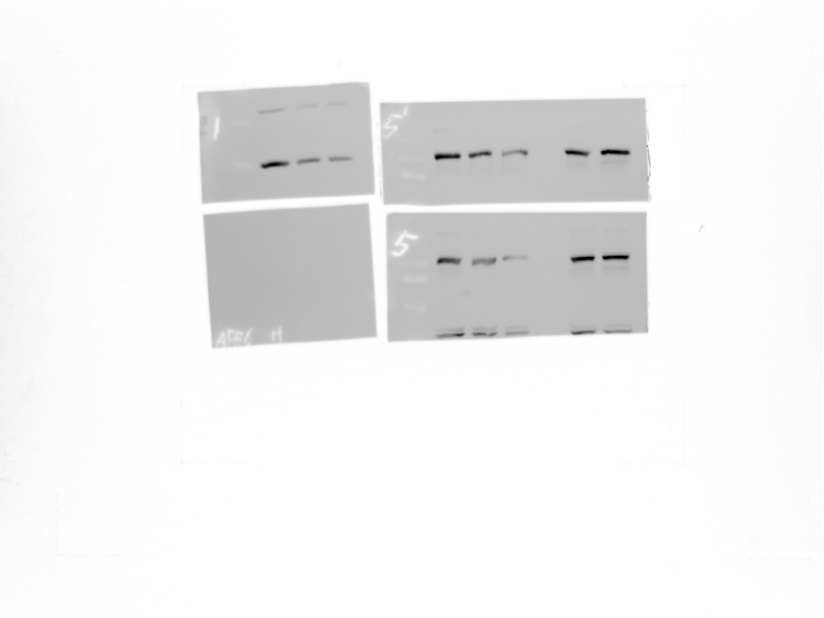

Supplement: Figure 4—source data 1. [file elife-92236-fig4-data1.zip › Figure_4-source_data_1/Figure_4-source_data_1_ Figure_4A_EZH2(right panel).jpg]

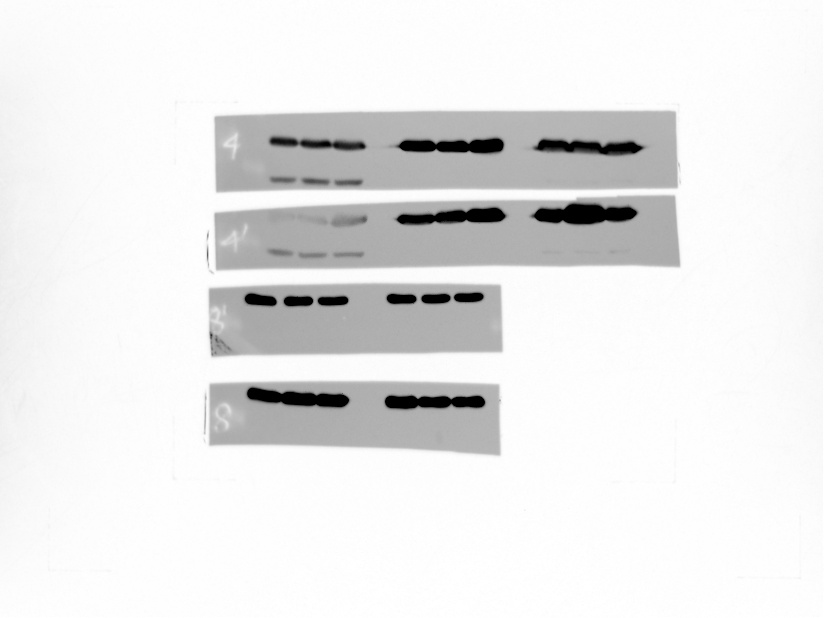

Supplement: Figure 4—source data 1. [file elife-92236-fig4-data1.zip › Figure_4-source_data_1/Figure_4-source_data_1_ Figure_4A_GAPDH(left panel).jpg]

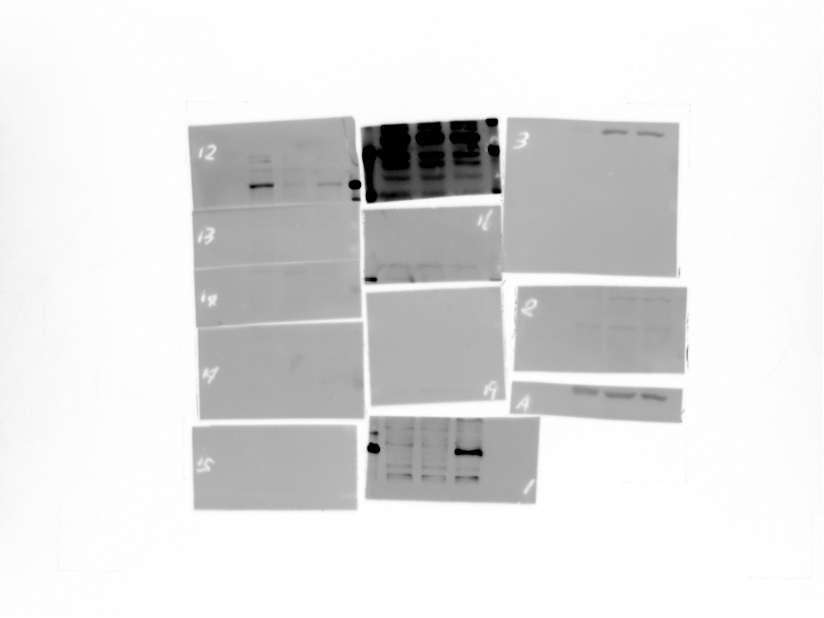

Supplement: Figure 4—source data 1. [file elife-92236-fig4-data1.zip › Figure_4-source_data_1/Figure_4-source_data_1_ Figure_4A_IBTK(left panel).jpg]

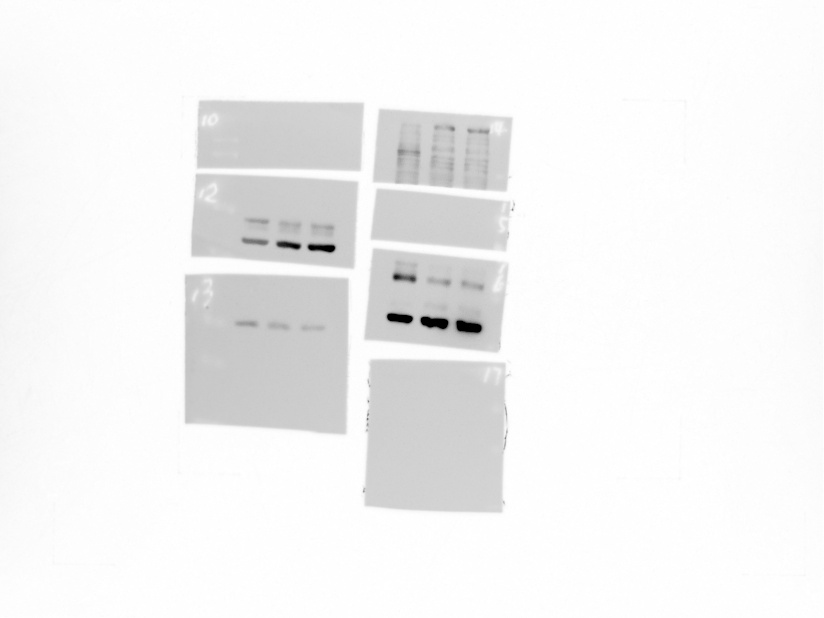

Supplement: Figure 4—source data 1. [file elife-92236-fig4-data1.zip › Figure_4-source_data_1/Figure_4-source_data_1_ Figure_4A_MYC(left panel).jpg]

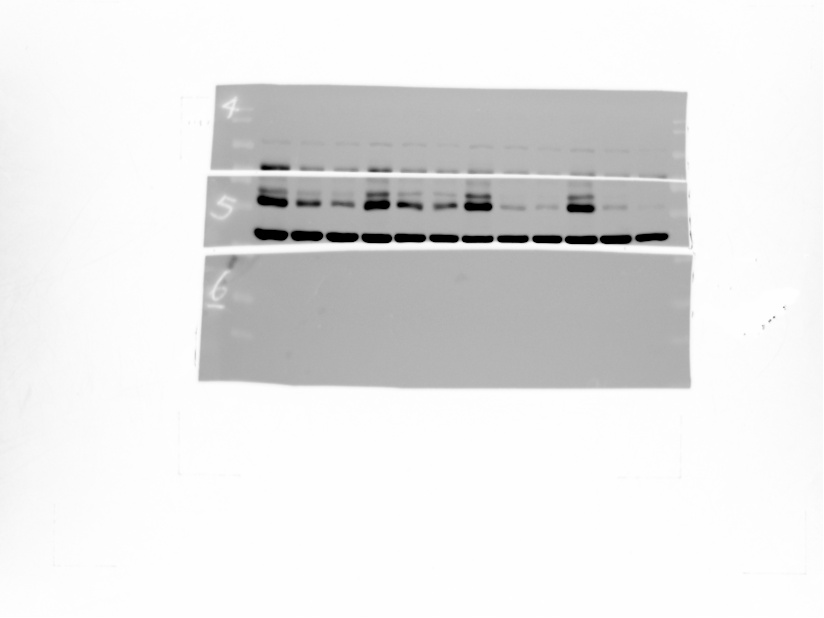

Supplement: Figure 4—source data 1. [file elife-92236-fig4-data1.zip › Figure_4-source_data_1/Figure_4-source_data_1_ Figure_4A_MYC(right panel).jpg]

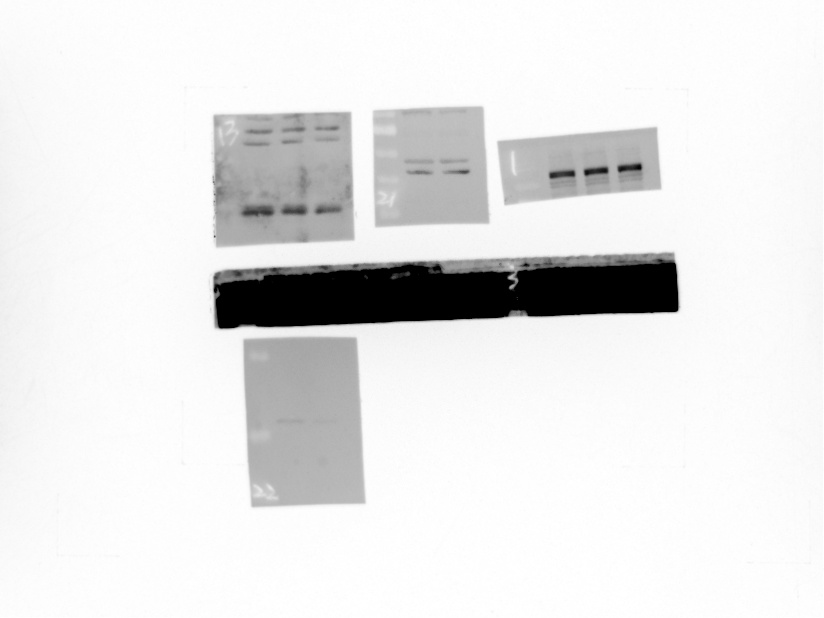

Supplement: Figure 4—source data 1. [file elife-92236-fig4-data1.zip › Figure_4-source_data_1/Figure_4-source_data_1_ Figure_4A_NOTCH1(left panel).jpg]

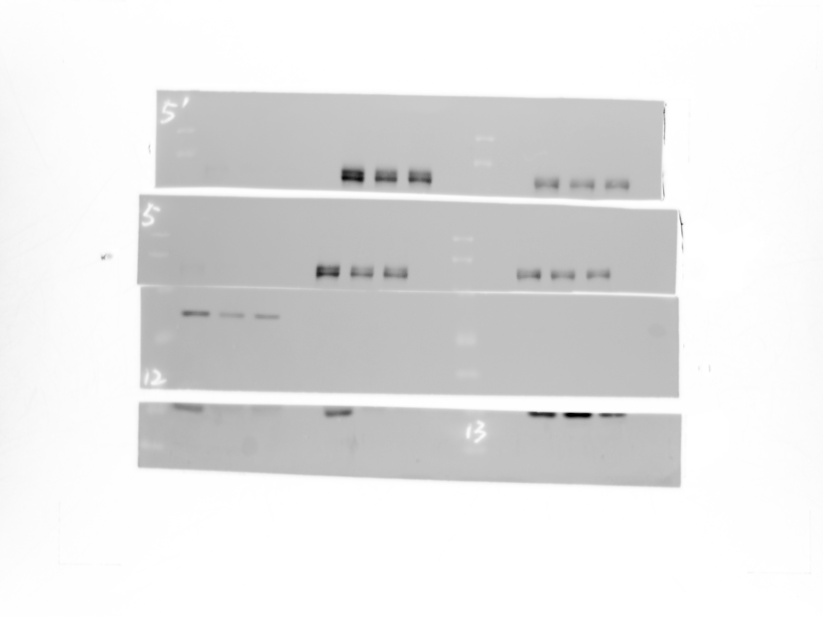

Supplement: Figure 4—source data 1. [file elife-92236-fig4-data1.zip › Figure_4-source_data_1/Figure_4-source_data_1_ Figure_4A_NOTCH1(right panel).jpg]

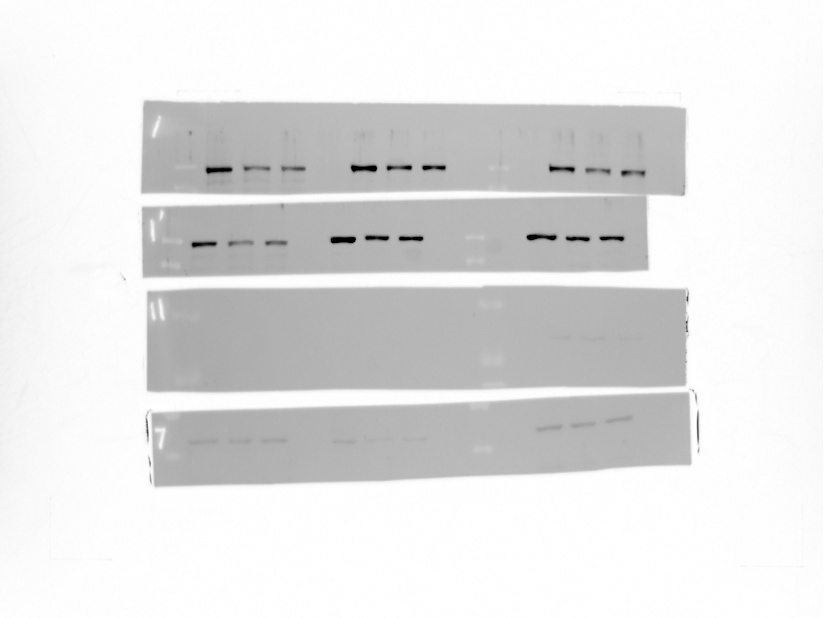

Supplement: Figure 4—source data 1. [file elife-92236-fig4-data1.zip › Figure_4-source_data_1/Figure_4-source_data_1_ Figure_4A_ROCK1(left panel).jpg]

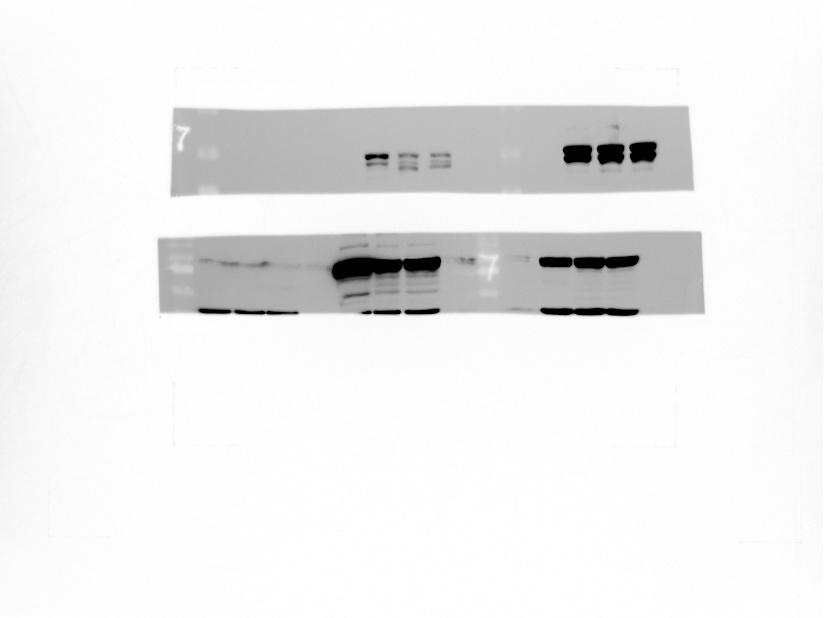

Supplement: Figure 4—source data 1. [file elife-92236-fig4-data1.zip › Figure_4-source_data_1/Figure_4-source_data_1_ Figure_4A_STAT1(left panel).jpg]

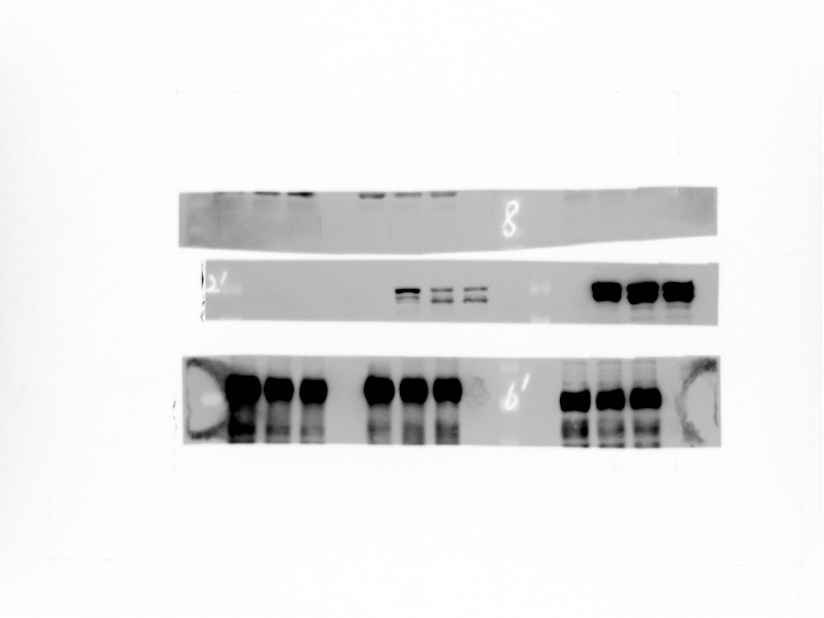

Supplement: Figure 4—source data 1. [file elife-92236-fig4-data1.zip › Figure_4-source_data_1/Figure_4-source_data_1_ Figure_4A_STAT1(right panel).jpg]

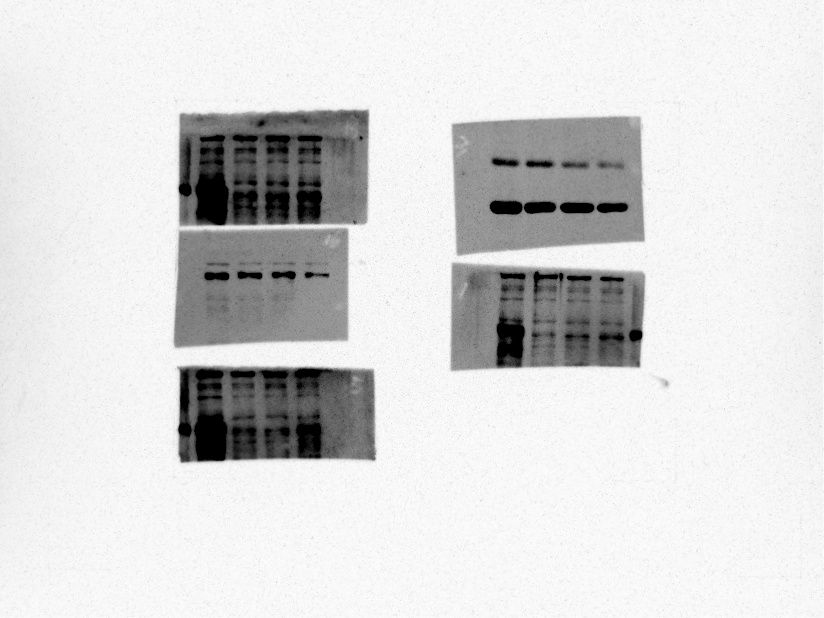

Supplement: Figure 4—source data 1. [file elife-92236-fig4-data1.zip › Figure_4-source_data_1/Figure_4-source_data_1_ Figure_4A_XIAP(left panel).jpg]

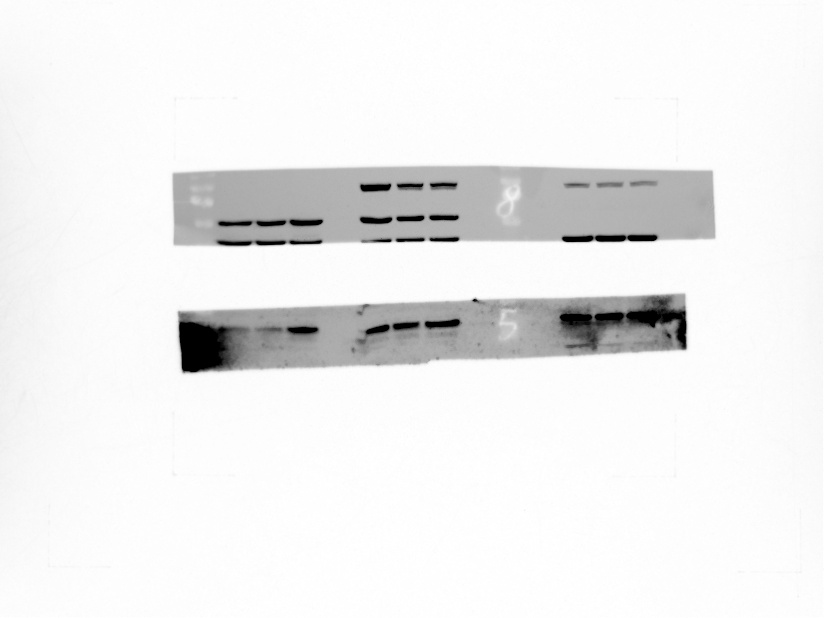

Supplement: Figure 4—source data 1. [file elife-92236-fig4-data1.zip › Figure_4-source_data_1/Figure_4-source_data_1_ Figure_4A_XIAP(right panel).jpg]

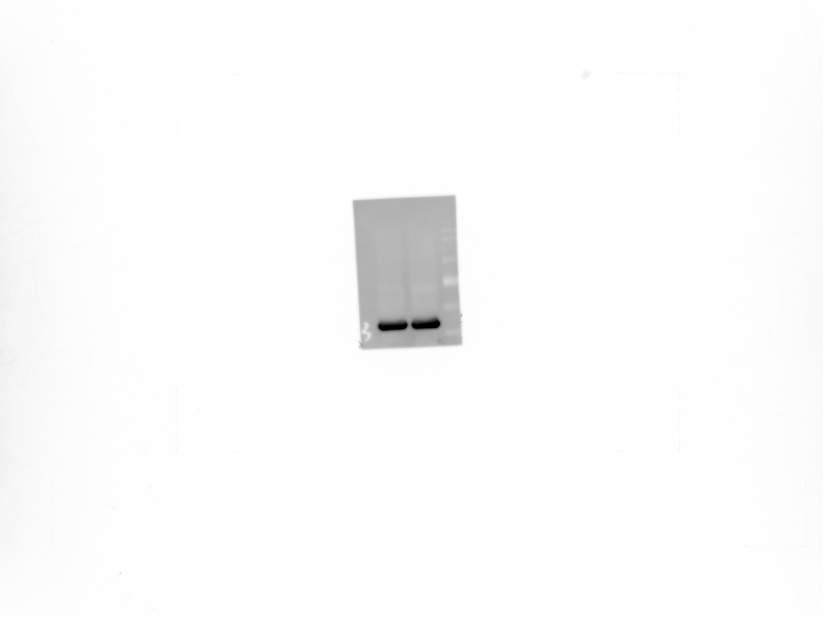

Supplement: Figure 4—source data 1. [file elife-92236-fig4-data1.zip › Figure_4-source_data_1/Figure_4-source_data_1_ Figure_4C_Actin.jpg]

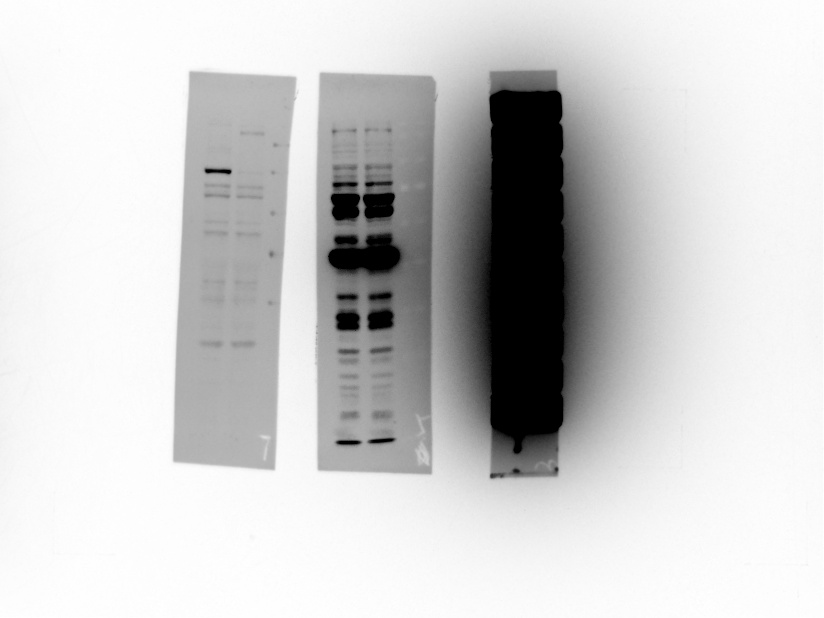

Supplement: Figure 4—source data 1. [file elife-92236-fig4-data1.zip › Figure_4-source_data_1/Figure_4-source_data_1_ Figure_4C_ARF6.jpg]

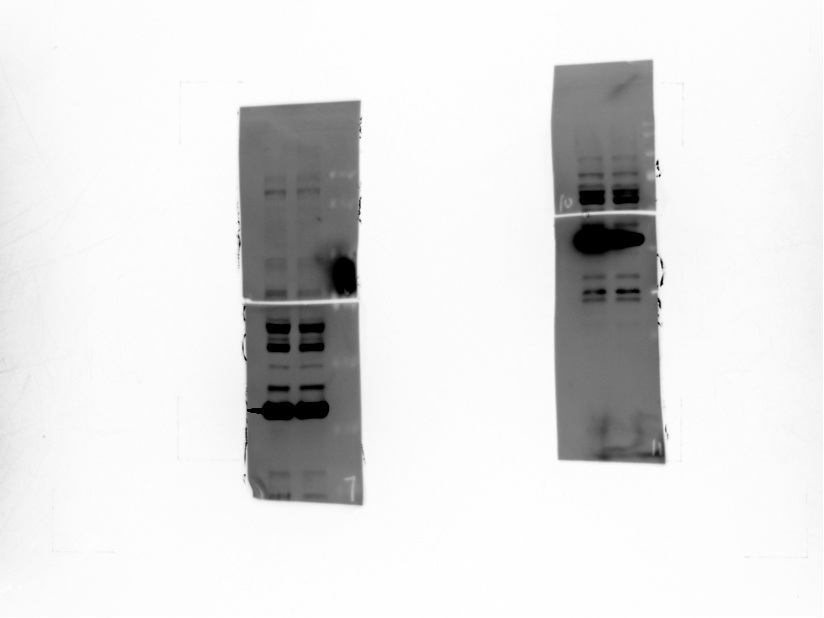

Supplement: Figure 4—source data 1. [file elife-92236-fig4-data1.zip › Figure_4-source_data_1/Figure_4-source_data_1_ Figure_4C_CCND3.jpg]

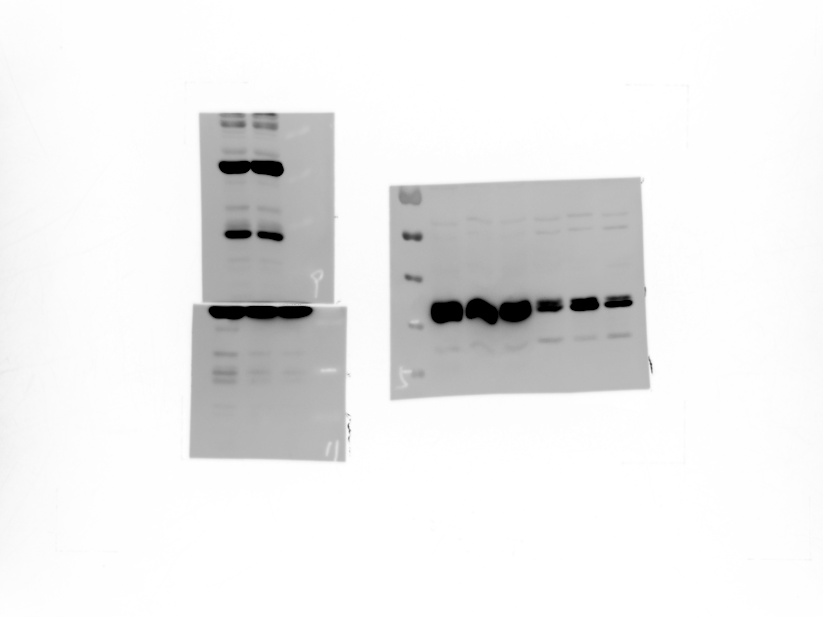

Supplement: Figure 4—source data 1. [file elife-92236-fig4-data1.zip › Figure_4-source_data_1/Figure_4-source_data_1_ Figure_4C_CDK4.jpg]

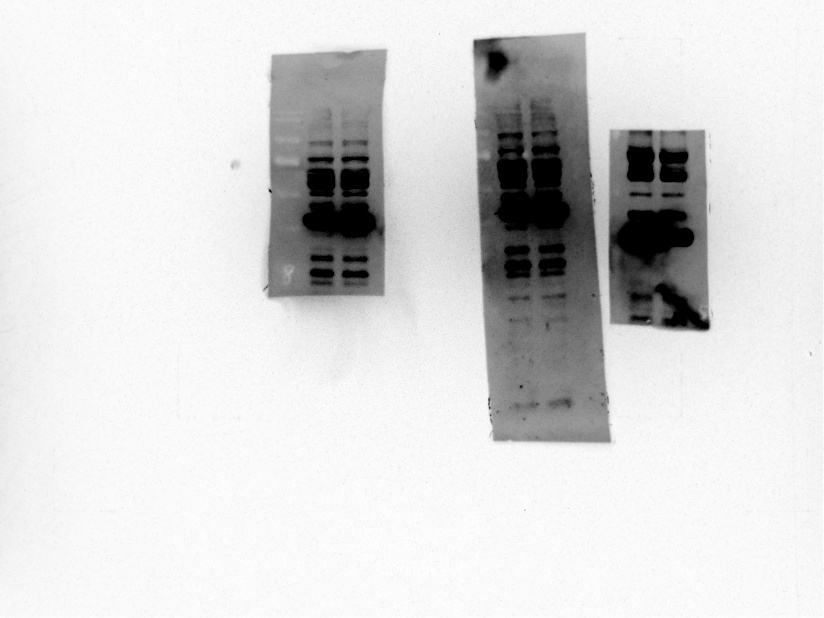

Supplement: Figure 4—source data 1. [file elife-92236-fig4-data1.zip › Figure_4-source_data_1/Figure_4-source_data_1_ Figure_4C_CDK6.jpg]

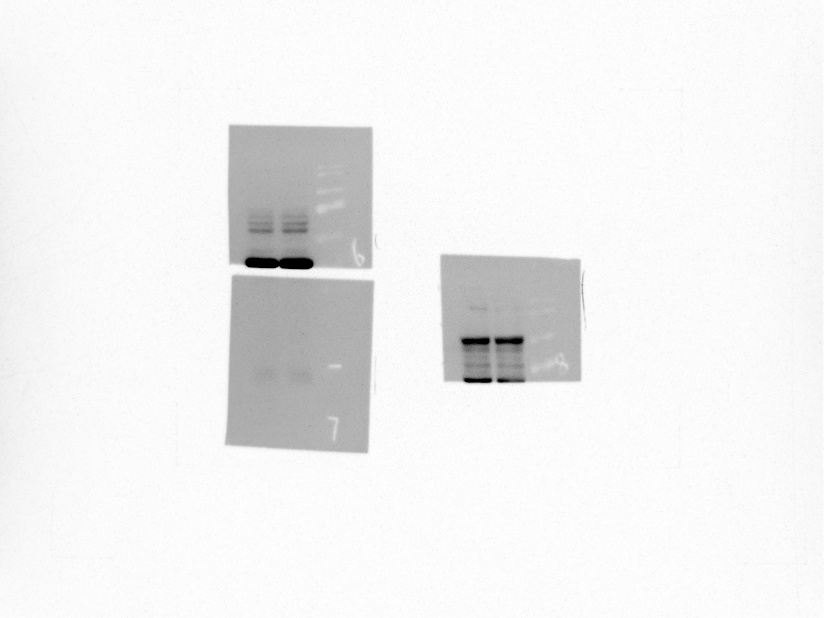

Supplement: Figure 4—source data 1. [file elife-92236-fig4-data1.zip › Figure_4-source_data_1/Figure_4-source_data_1_ Figure_4C_EZH2.jpg]

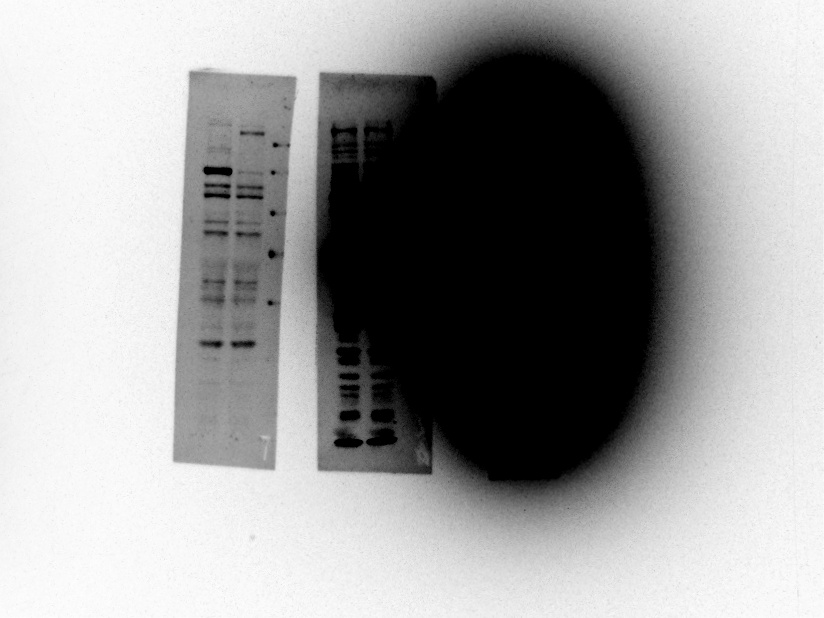

Supplement: Figure 4—source data 1. [file elife-92236-fig4-data1.zip › Figure_4-source_data_1/Figure_4-source_data_1_ Figure_4C_FLAG.jpg]

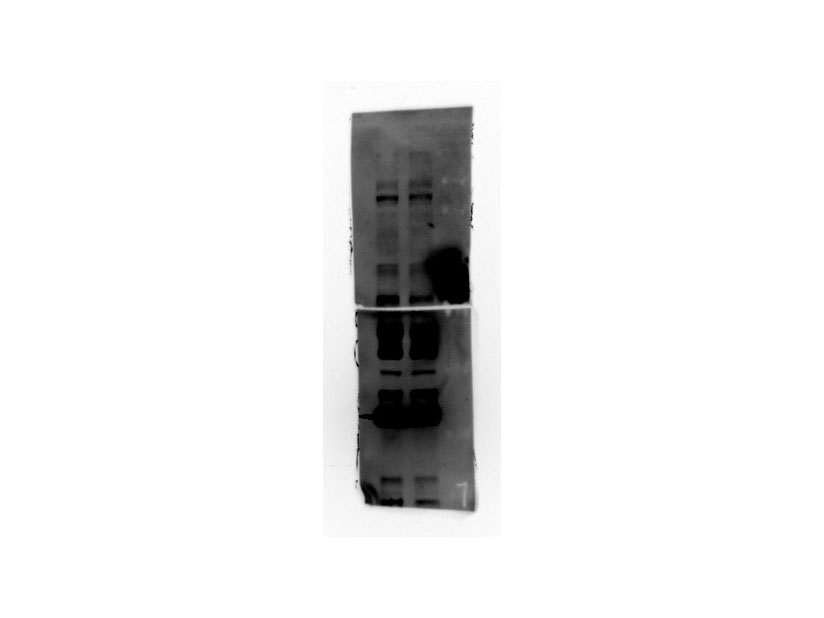

Supplement: Figure 4—source data 1. [file elife-92236-fig4-data1.zip › Figure_4-source_data_1/Figure_4-source_data_1_ Figure_4C_MYC.jpg]

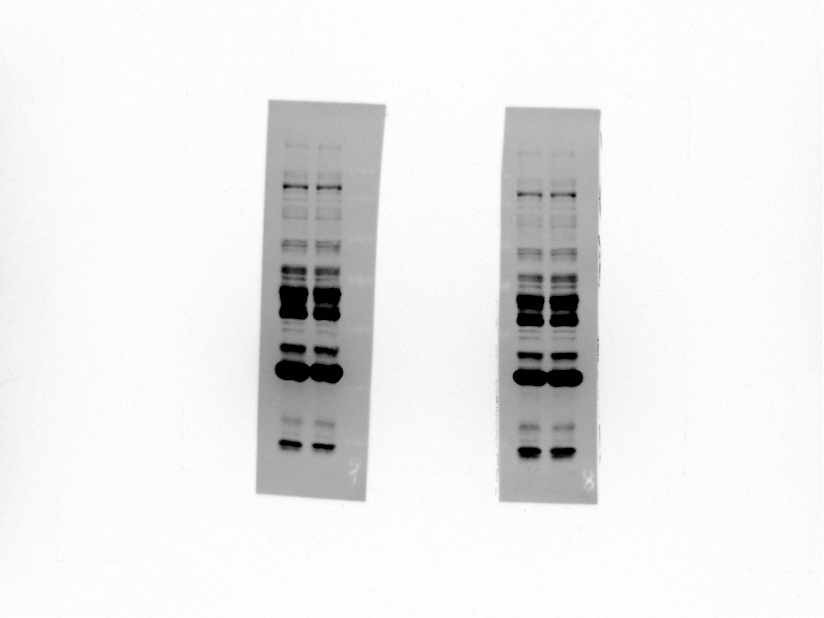

Supplement: Figure 4—source data 1. [file elife-92236-fig4-data1.zip › Figure_4-source_data_1/Figure_4-source_data_1_ Figure_4C_NOTCH1.jpg]

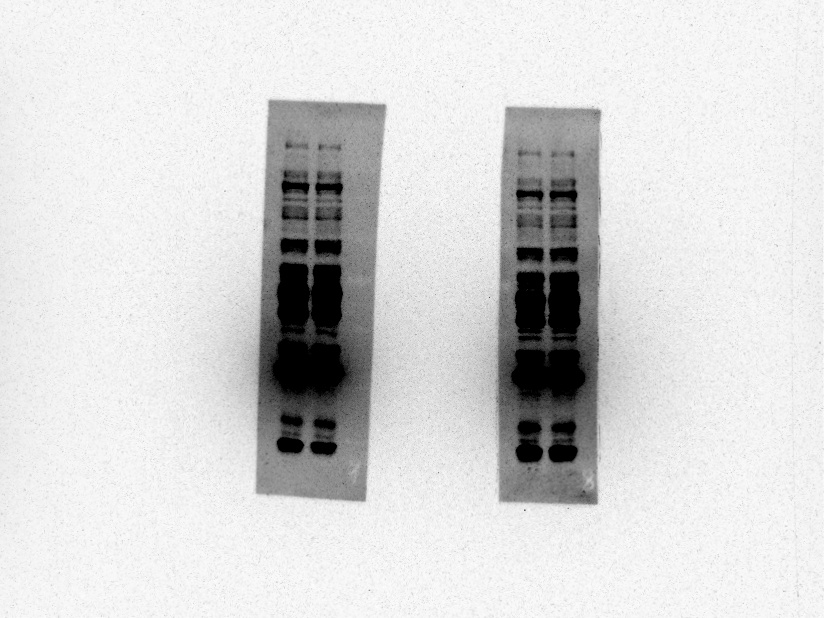

Supplement: Figure 4—source data 1. [file elife-92236-fig4-data1.zip › Figure_4-source_data_1/Figure_4-source_data_1_ Figure_4C_ROCK1.jpg]

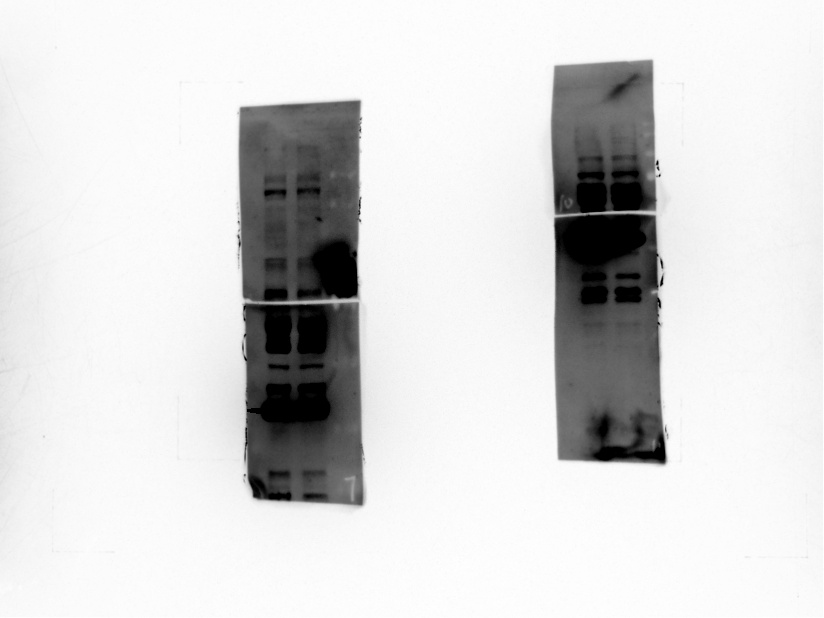

Supplement: Figure 4—source data 1. [file elife-92236-fig4-data1.zip › Figure_4-source_data_1/Figure_4-source_data_1_ Figure_4C_STAT1.jpg]

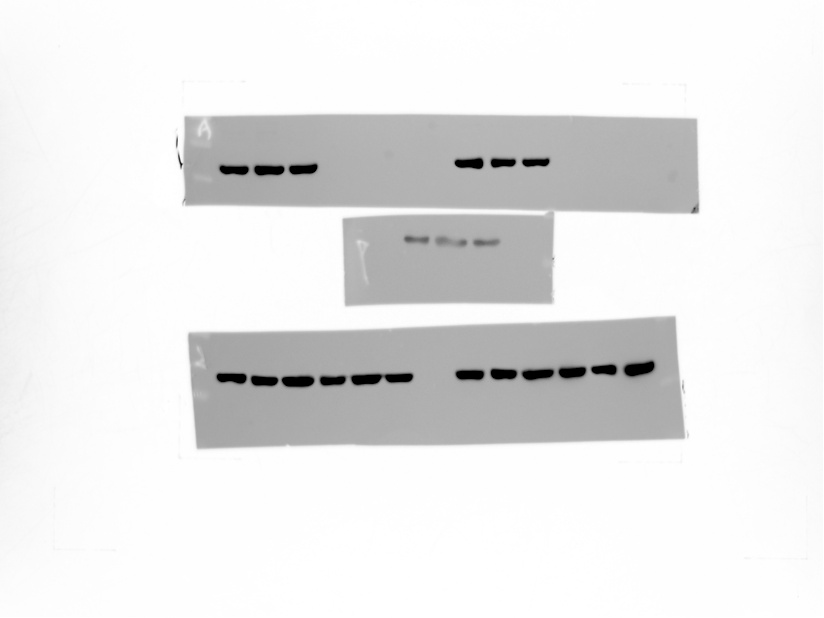

Supplement: Figure 4—source data 1. [file elife-92236-fig4-data1.zip › Figure_4-source_data_1/Figure_4-source_data_1_ Figure_4I_Actin.jpg]

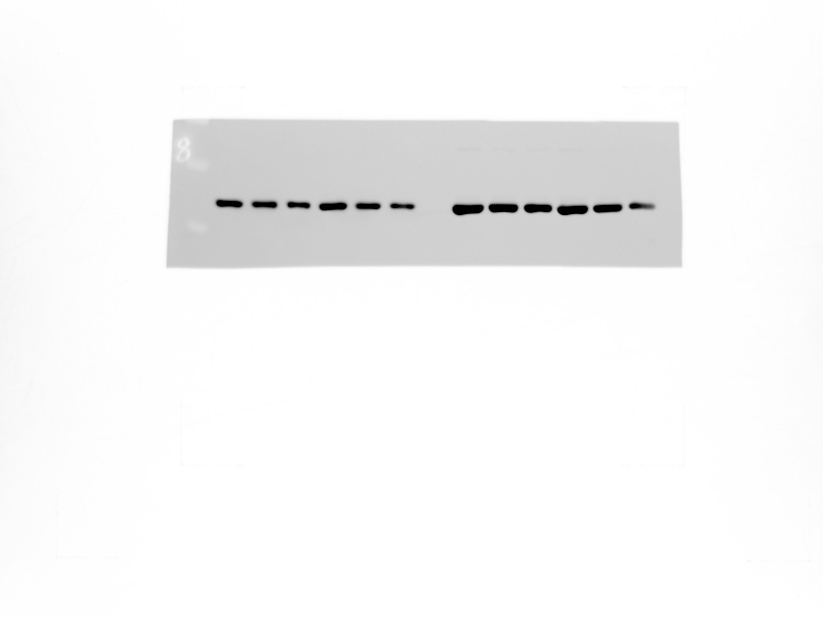

Supplement: Figure 4—source data 1. [file elife-92236-fig4-data1.zip › Figure_4-source_data_1/Figure_4-source_data_1_ Figure_4I_CASP7.jpg]

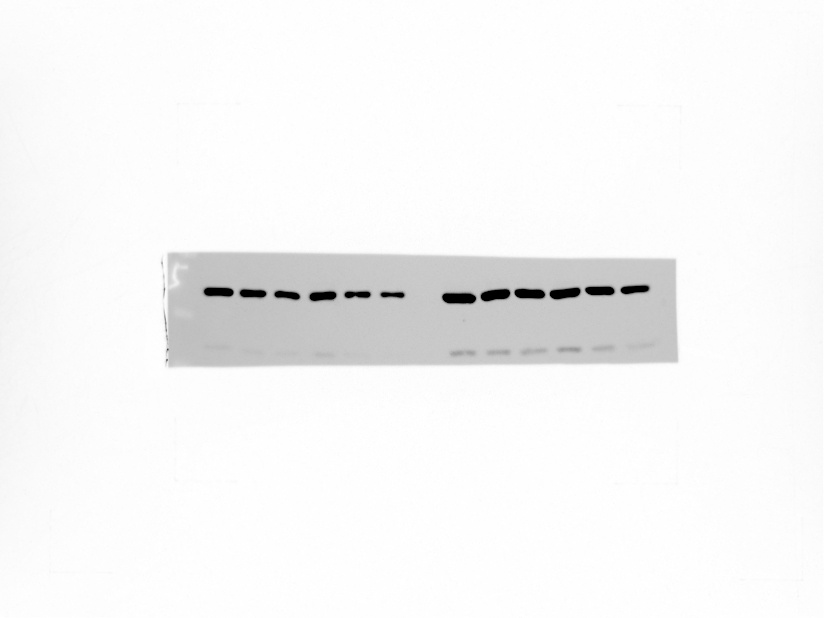

Supplement: Figure 4—source data 1. [file elife-92236-fig4-data1.zip › Figure_4-source_data_1/Figure_4-source_data_1_ Figure_4I_CASP9.jpg]

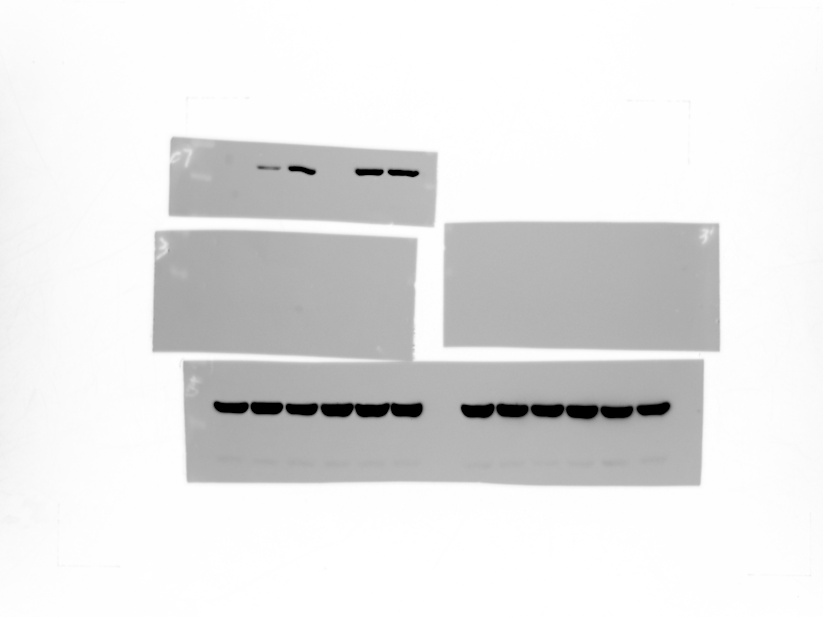

Supplement: Figure 4—source data 1. [file elife-92236-fig4-data1.zip › Figure_4-source_data_1/Figure_4-source_data_1_ Figure_4I_cl-CASP7.jpg]

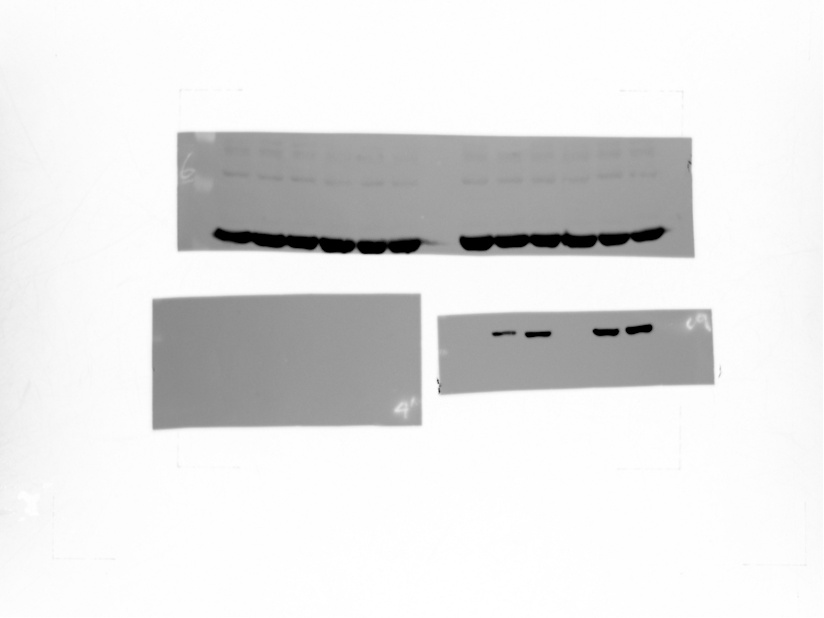

Supplement: Figure 4—source data 1. [file elife-92236-fig4-data1.zip › Figure_4-source_data_1/Figure_4-source_data_1_ Figure_4I_cl-CASP9.jpg]

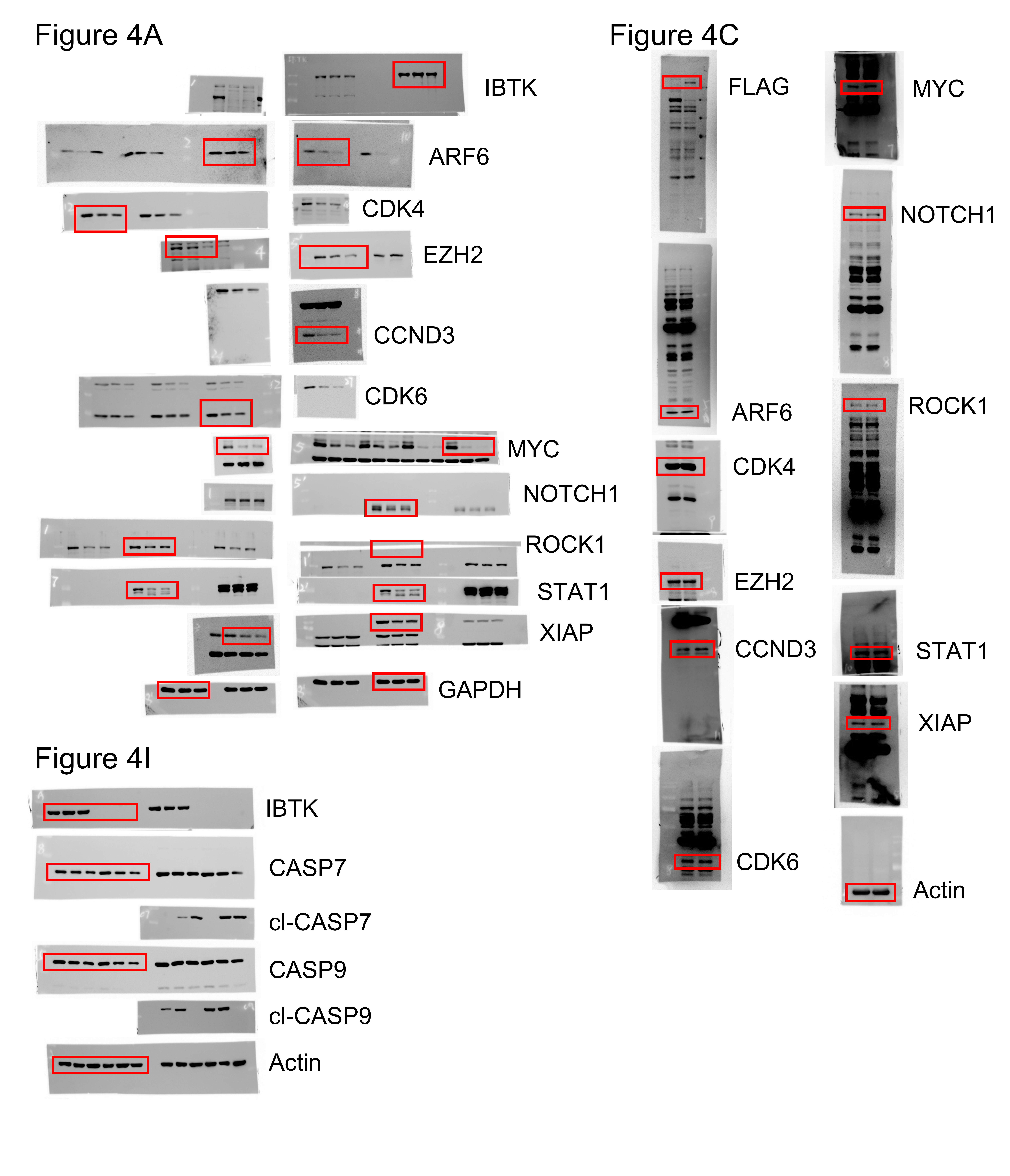

Supplement: Figure 4—source data 2. [file elife-92236-fig4-data2.zip › Figure_4-source_data_2.jpg]

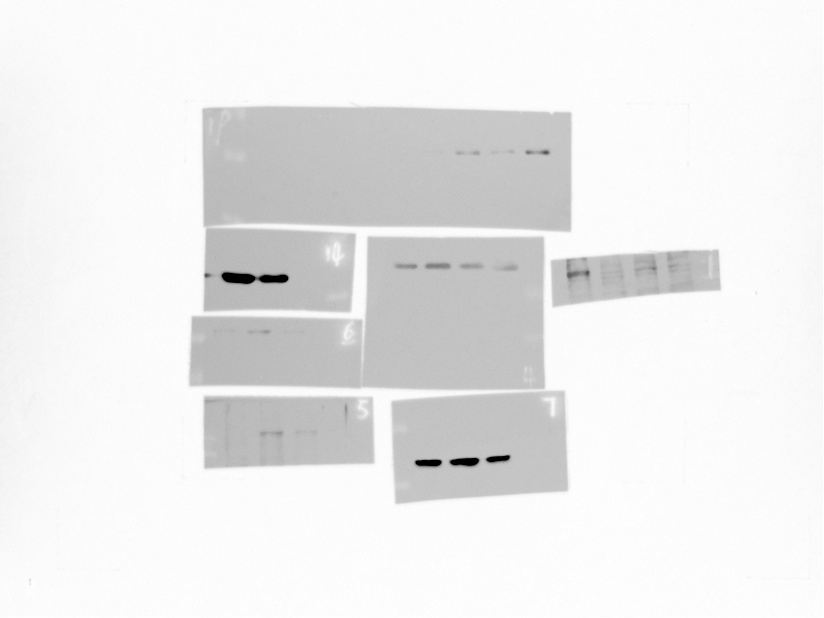

Supplement: Figure 4—figure supplement 1—source data 1. [file elife-92236-fig4-figsupp1-data1.zip › Figure_4-Figure Supplement_1-source_data_1/Figure_4-figure supplement_1_ source_data_1_ Figure_A_Actin(left panel).jpg]

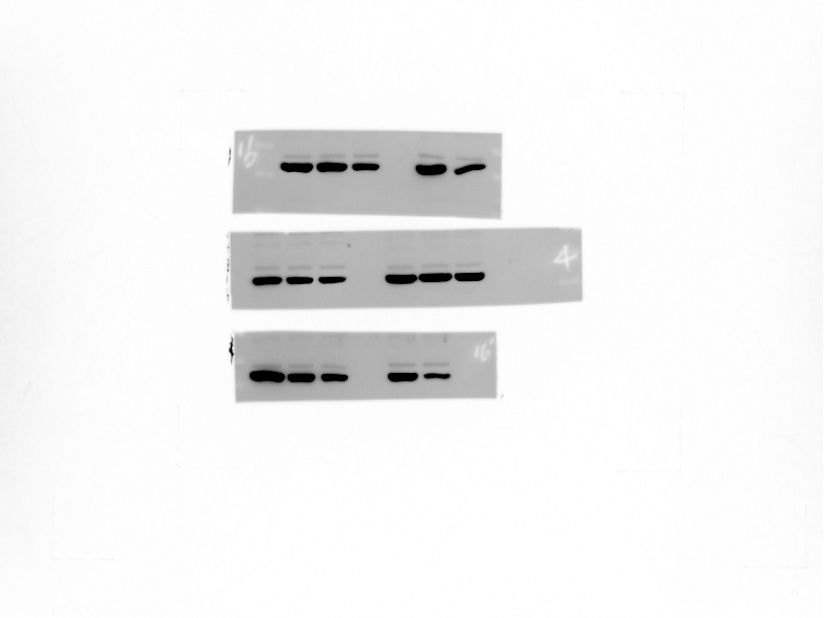

Supplement: Figure 4—figure supplement 1—source data 1. [file elife-92236-fig4-figsupp1-data1.zip › Figure_4-Figure Supplement_1-source_data_1/Figure_4-figure supplement_1_ source_data_1_ Figure_A_Actin(right panel).jpg]

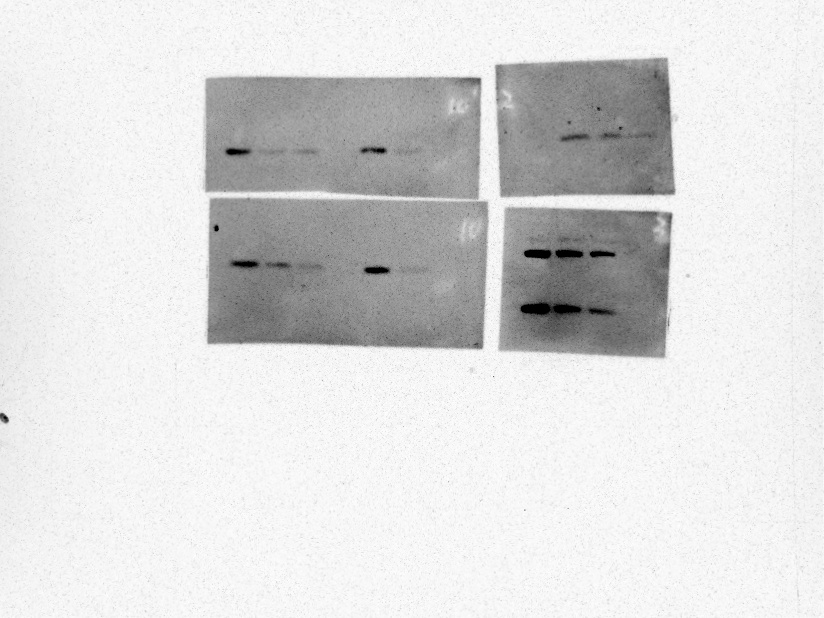

Supplement: Figure 4—figure supplement 1—source data 1. [file elife-92236-fig4-figsupp1-data1.zip › Figure_4-Figure Supplement_1-source_data_1/Figure_4-figure supplement_1_ source_data_1_ Figure_A_ARF6(left panel).jpg]

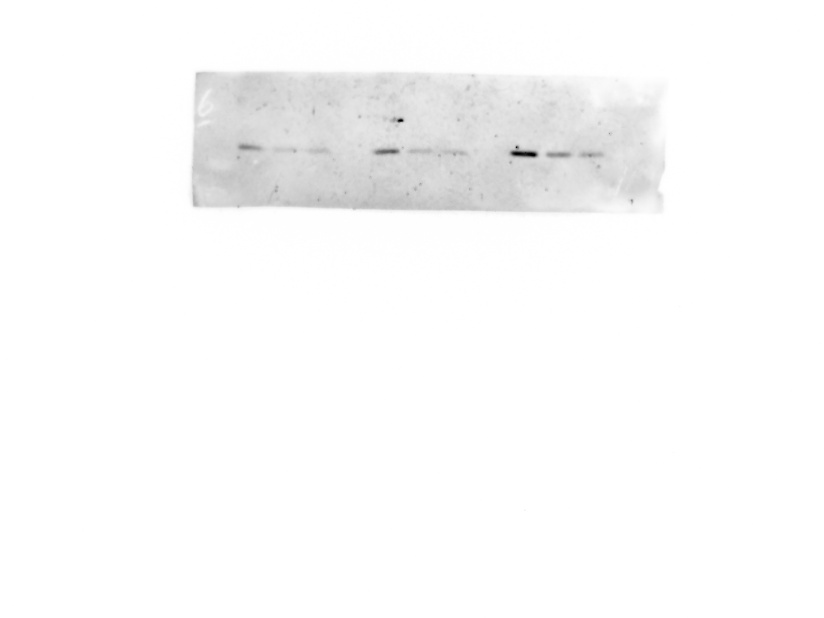

Supplement: Figure 4—figure supplement 1—source data 1. [file elife-92236-fig4-figsupp1-data1.zip › Figure_4-Figure Supplement_1-source_data_1/Figure_4-figure supplement_1_ source_data_1_ Figure_A_ARF6(right panel).jpg]

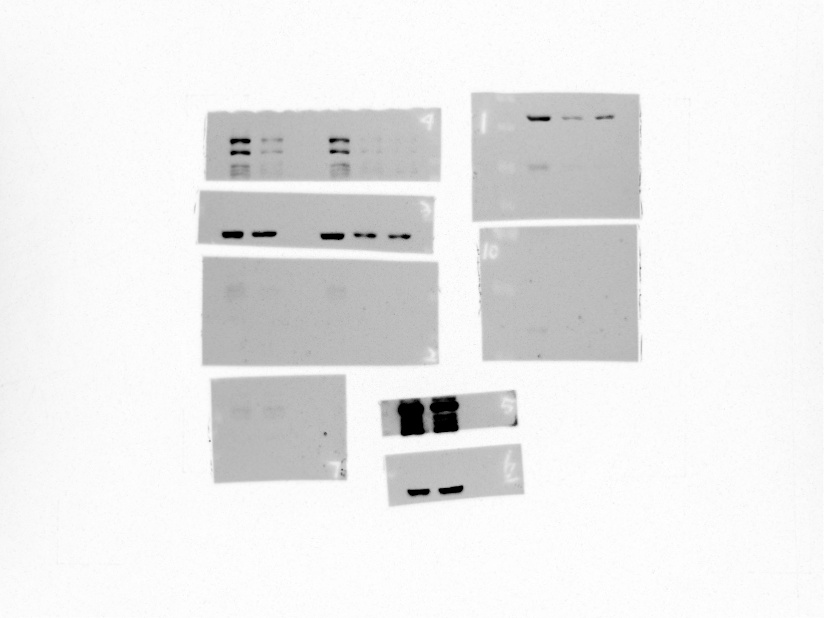

Supplement: Figure 4—figure supplement 1—source data 1. [file elife-92236-fig4-figsupp1-data1.zip › Figure_4-Figure Supplement_1-source_data_1/Figure_4-figure supplement_1_ source_data_1_ Figure_A_CCND3(left panel).jpg]

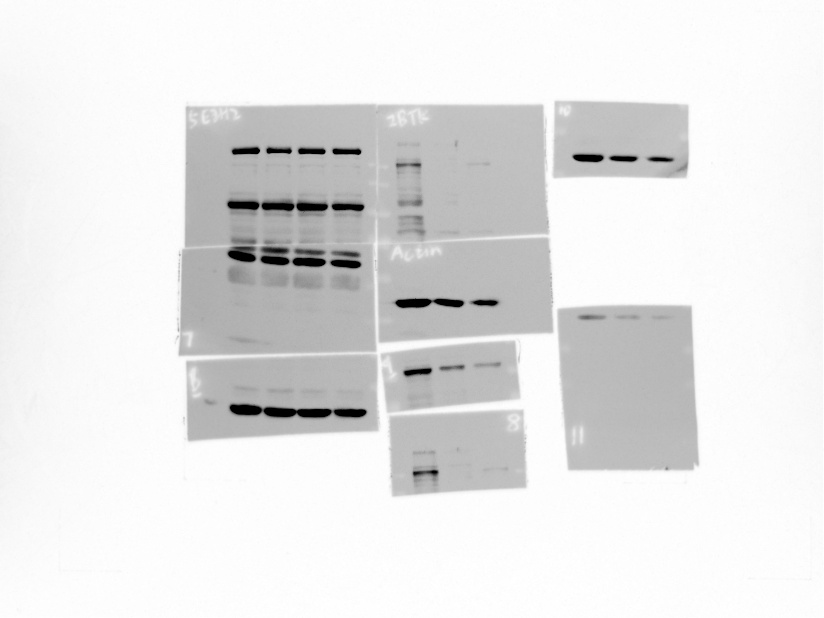

Supplement: Figure 4—figure supplement 1—source data 1. [file elife-92236-fig4-figsupp1-data1.zip › Figure_4-Figure Supplement_1-source_data_1/Figure_4-figure supplement_1_ source_data_1_ Figure_A_CCND3(right panel).jpg]

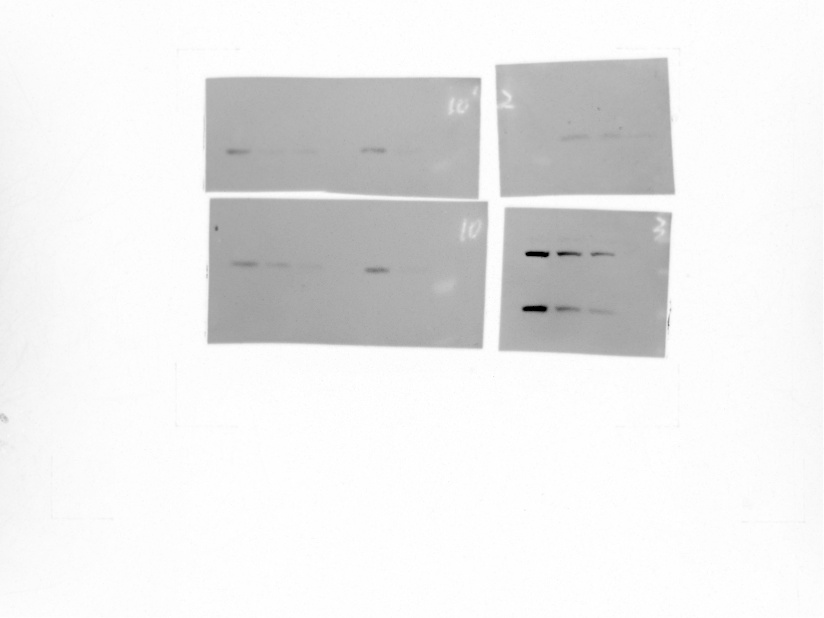

Supplement: Figure 4—figure supplement 1—source data 1. [file elife-92236-fig4-figsupp1-data1.zip › Figure_4-Figure Supplement_1-source_data_1/Figure_4-figure supplement_1_ source_data_1_ Figure_A_CDK4(right panel).jpg]

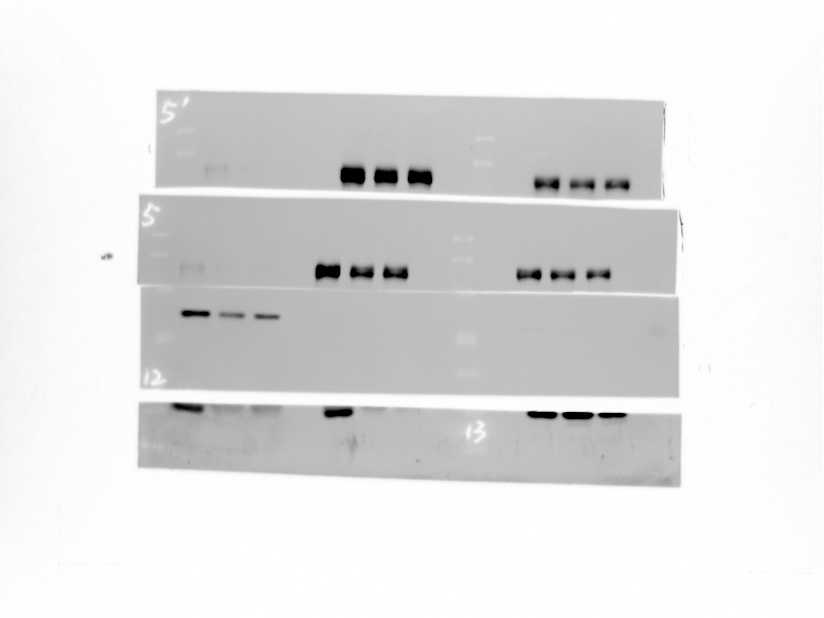

Supplement: Figure 4—figure supplement 1—source data 1. [file elife-92236-fig4-figsupp1-data1.zip › Figure_4-Figure Supplement_1-source_data_1/Figure_4-figure supplement_1_ source_data_1_ Figure_A_CDK6(left panel).jpg]

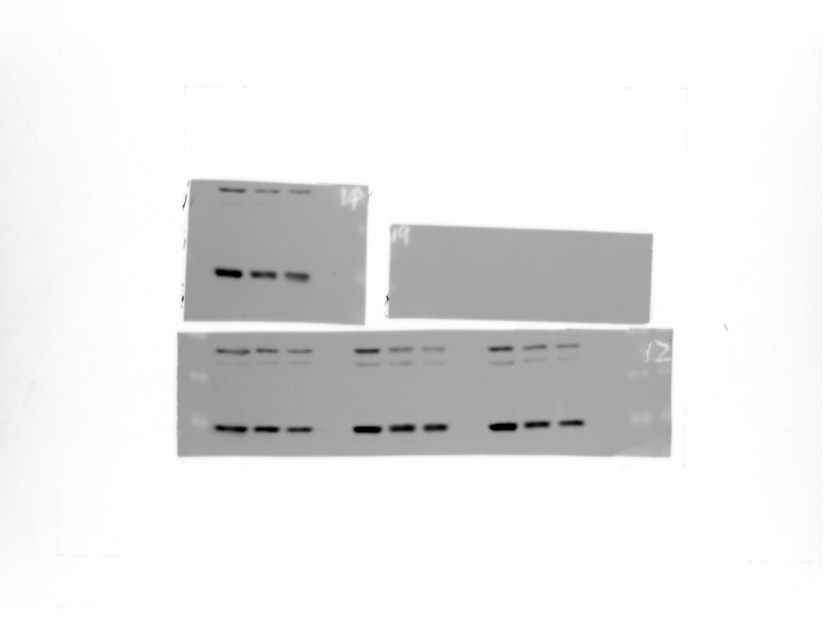

Supplement: Figure 4—figure supplement 1—source data 1. [file elife-92236-fig4-figsupp1-data1.zip › Figure_4-Figure Supplement_1-source_data_1/Figure_4-figure supplement_1_ source_data_1_ Figure_A_CDK6(right panel).jpg]

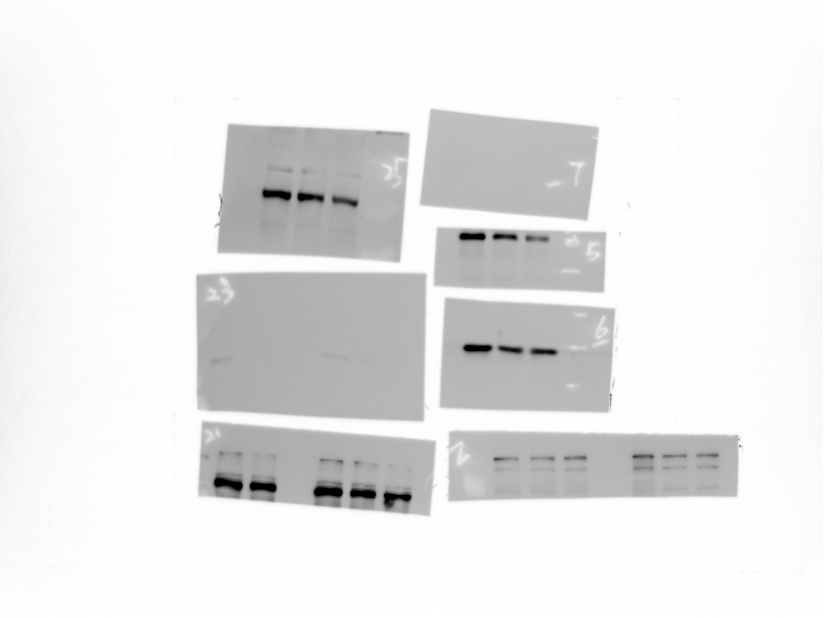

Supplement: Figure 4—figure supplement 1—source data 1. [file elife-92236-fig4-figsupp1-data1.zip › Figure_4-Figure Supplement_1-source_data_1/Figure_4-figure supplement_1_ source_data_1_ Figure_A_EZH2(left panel).jpg]

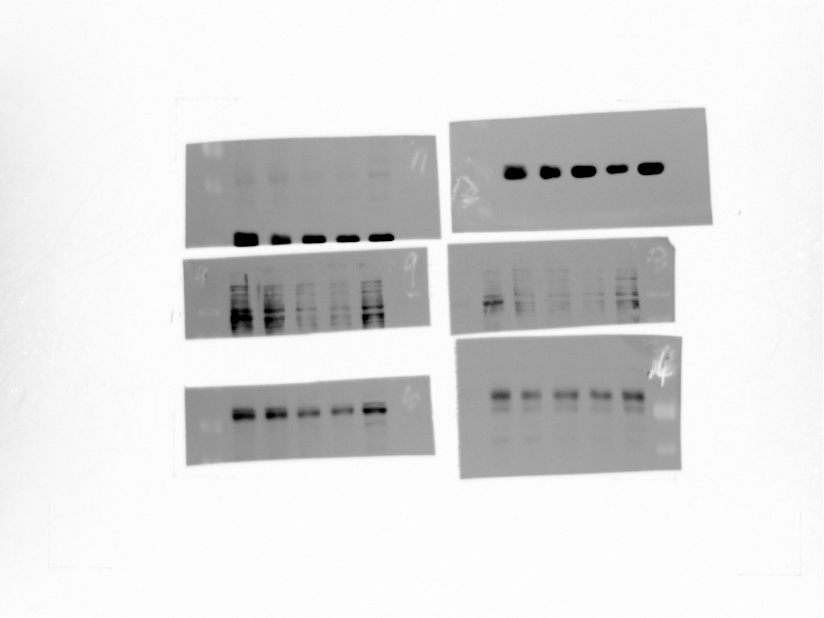

Supplement: Figure 4—figure supplement 1—source data 1. [file elife-92236-fig4-figsupp1-data1.zip › Figure_4-Figure Supplement_1-source_data_1/Figure_4-figure supplement_1_ source_data_1_ Figure_A_EZH2(right panel).jpg]

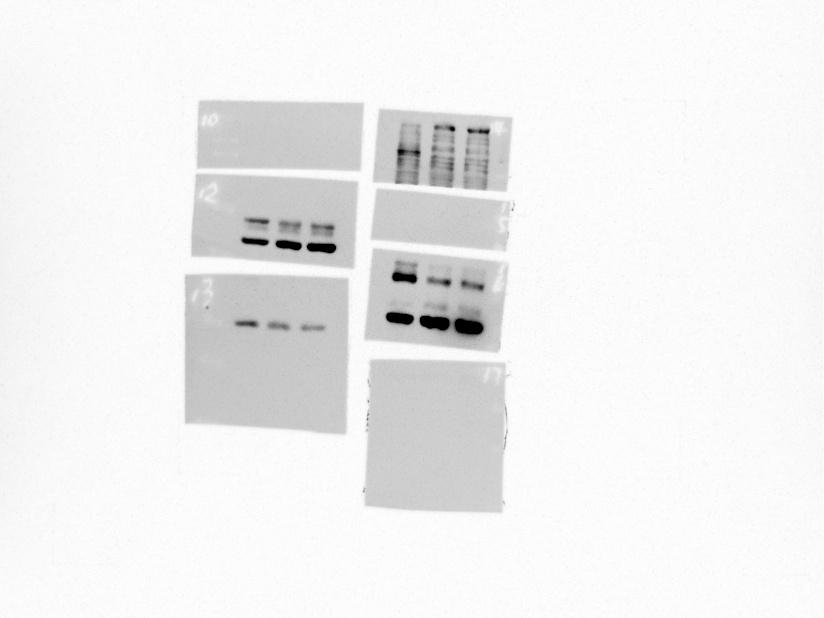

Supplement: Figure 4—figure supplement 1—source data 1. [file elife-92236-fig4-figsupp1-data1.zip › Figure_4-Figure Supplement_1-source_data_1/Figure_4-figure supplement_1_ source_data_1_ Figure_A_IBTK (left panel).jpg]

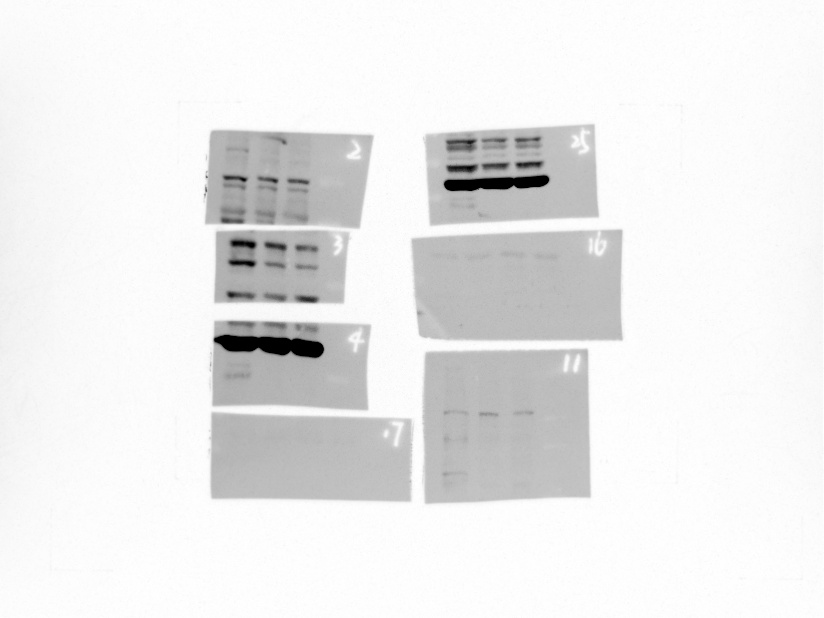

Supplement: Figure 4—figure supplement 1—source data 1. [file elife-92236-fig4-figsupp1-data1.zip › Figure_4-Figure Supplement_1-source_data_1/Figure_4-figure supplement_1_ source_data_1_ Figure_A_MYC(left panel).jpg]

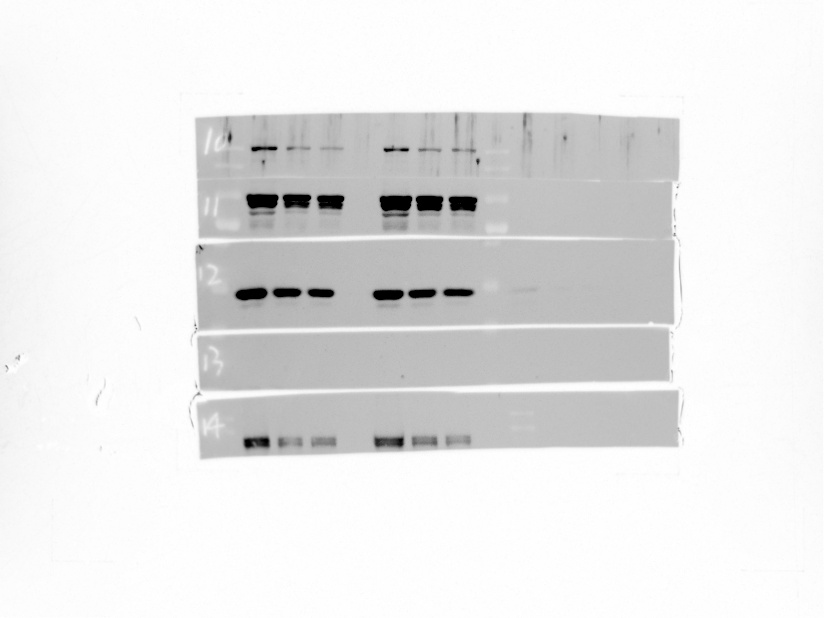

Supplement: Figure 4—figure supplement 1—source data 1. [file elife-92236-fig4-figsupp1-data1.zip › Figure_4-Figure Supplement_1-source_data_1/Figure_4-figure supplement_1_ source_data_1_ Figure_A_NOTCH1(left panel).jpg]

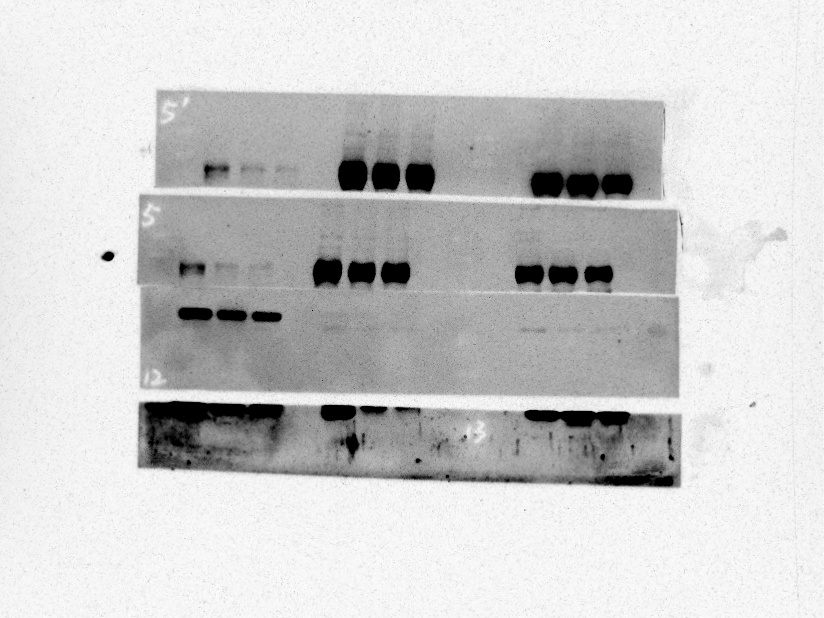

Supplement: Figure 4—figure supplement 1—source data 1. [file elife-92236-fig4-figsupp1-data1.zip › Figure_4-Figure Supplement_1-source_data_1/Figure_4-figure supplement_1_ source_data_1_ Figure_A_NOTCH1(right panel).jpg]

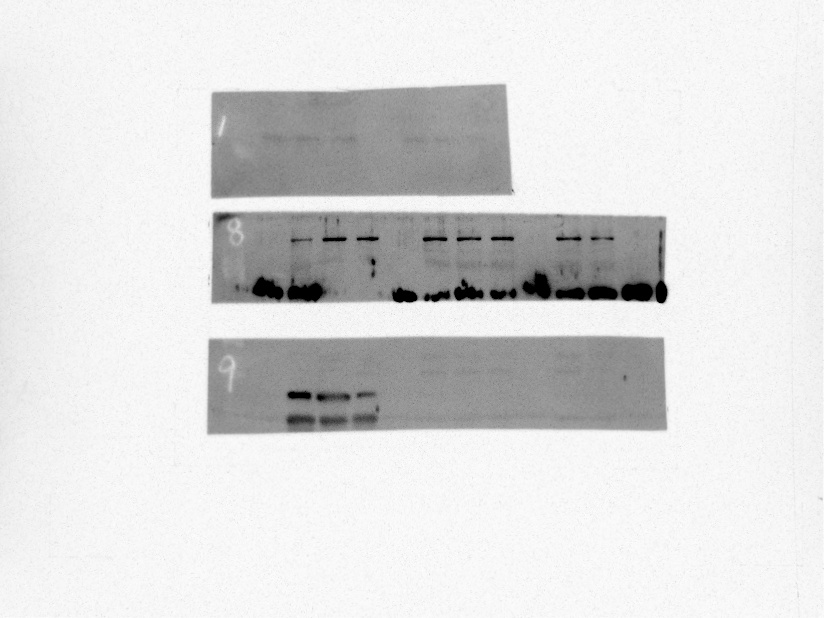

Supplement: Figure 4—figure supplement 1—source data 1. [file elife-92236-fig4-figsupp1-data1.zip › Figure_4-Figure Supplement_1-source_data_1/Figure_4-figure supplement_1_ source_data_1_ Figure_A_ROCK1(left panel).jpg]

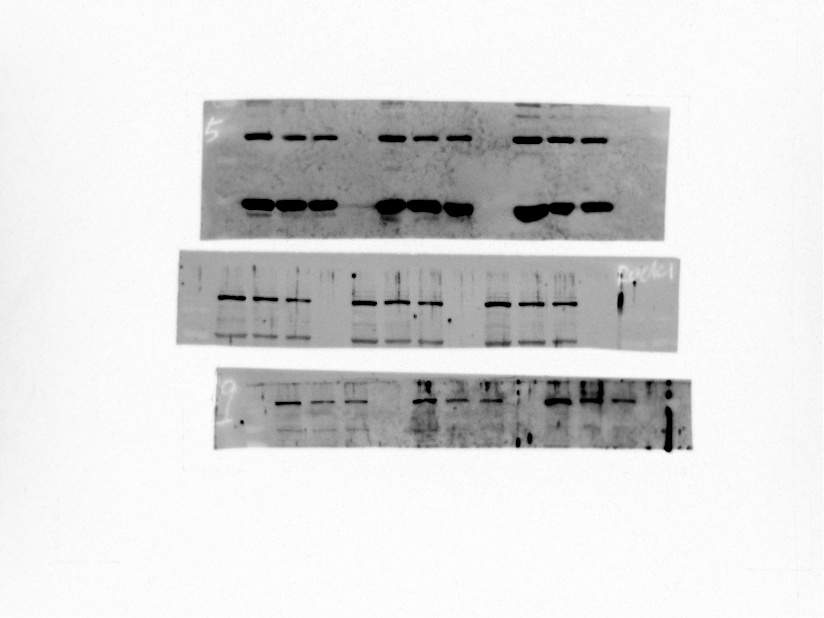

Supplement: Figure 4—figure supplement 1—source data 1. [file elife-92236-fig4-figsupp1-data1.zip › Figure_4-Figure Supplement_1-source_data_1/Figure_4-figure supplement_1_ source_data_1_ Figure_A_ROCK1(right panel).jpg]

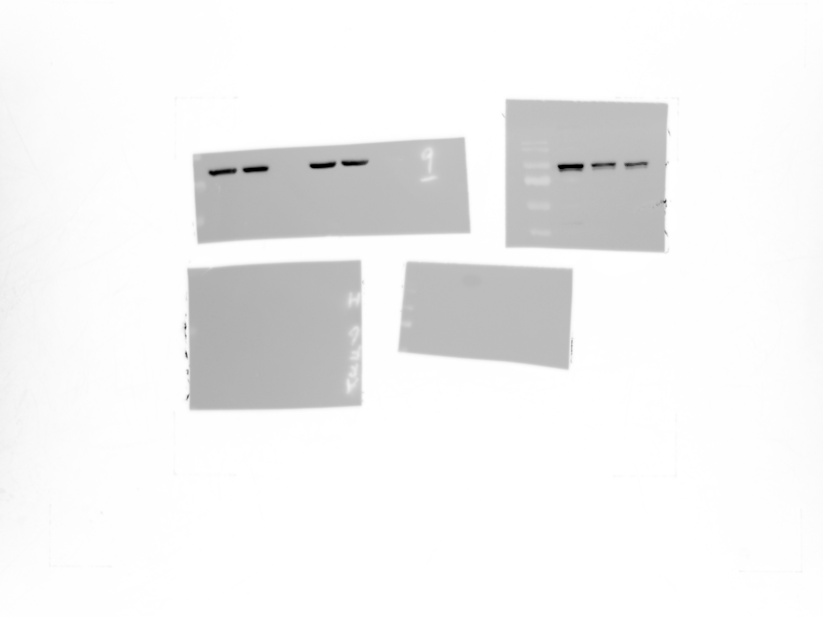

Supplement: Figure 4—figure supplement 1—source data 1. [file elife-92236-fig4-figsupp1-data1.zip › Figure_4-Figure Supplement_1-source_data_1/Figure_4-figure supplement_1_ source_data_1_ Figure_A_STAT1(left panel).jpg]

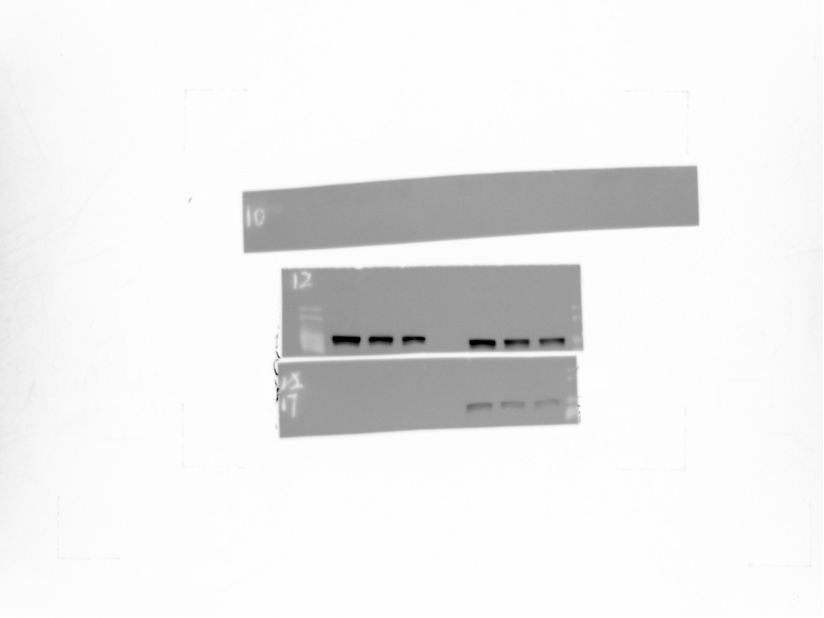

Supplement: Figure 4—figure supplement 1—source data 1. [file elife-92236-fig4-figsupp1-data1.zip › Figure_4-Figure Supplement_1-source_data_1/Figure_4-figure supplement_1_ source_data_1_ Figure_A_STAT1(right panel).jpg]

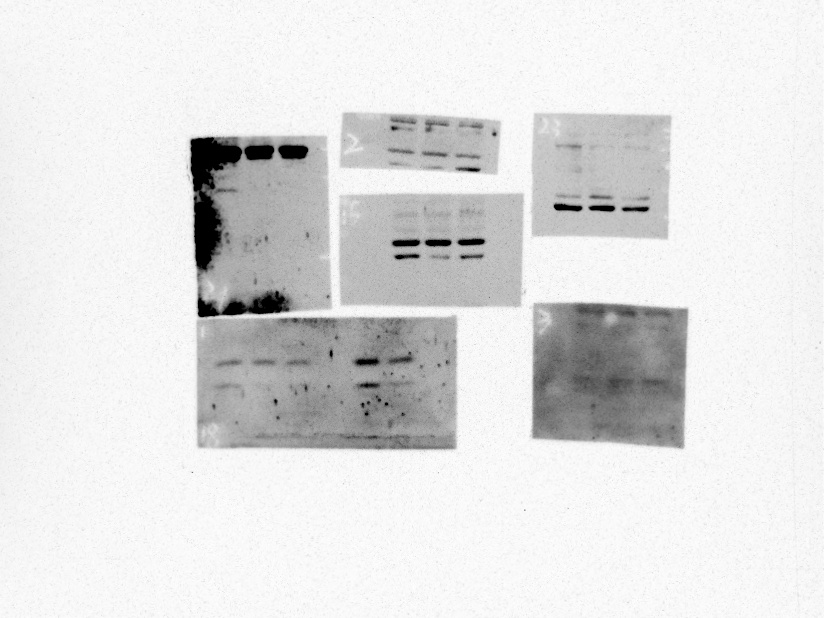

Supplement: Figure 4—figure supplement 1—source data 1. [file elife-92236-fig4-figsupp1-data1.zip › Figure_4-Figure Supplement_1-source_data_1/Figure_4-figure supplement_1_ source_data_1_ Figure_A_XIAP(left panel).jpg]

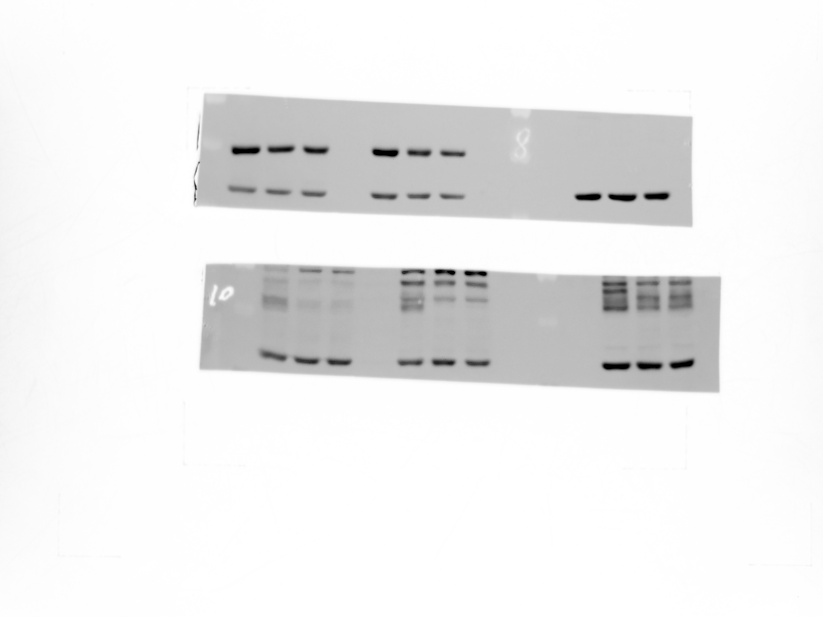

Supplement: Figure 4—figure supplement 1—source data 1. [file elife-92236-fig4-figsupp1-data1.zip › Figure_4-Figure Supplement_1-source_data_1/Figure_4-figure supplement_1_ source_data_1_ Figure_A_XIAP(right panel).jpg]

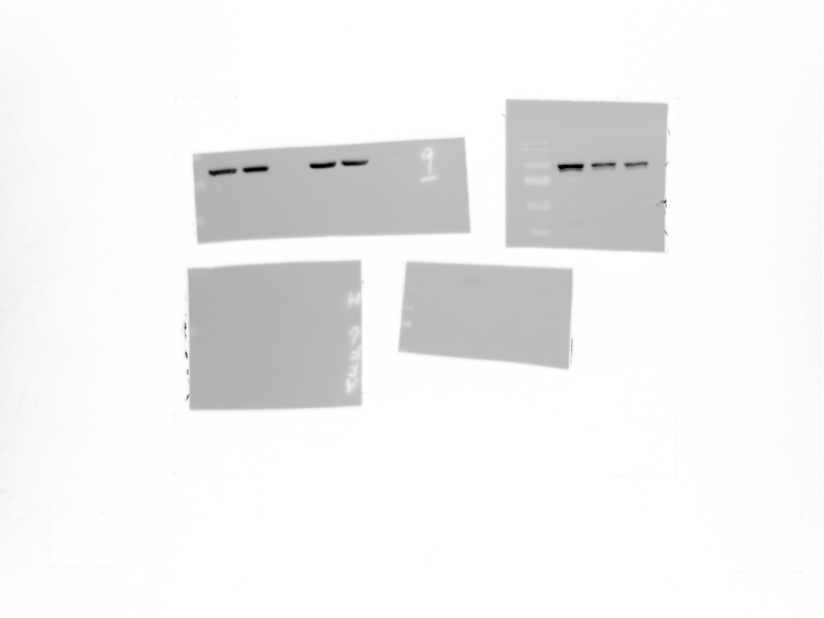

Supplement: Figure 4—figure supplement 1—source data 1. [file elife-92236-fig4-figsupp1-data1.zip › Figure_4-Figure Supplement_1-source_data_1/Figure_4-figure supplement_1_ source_data_1_ Figure_C_Actin(left panel).jpg]

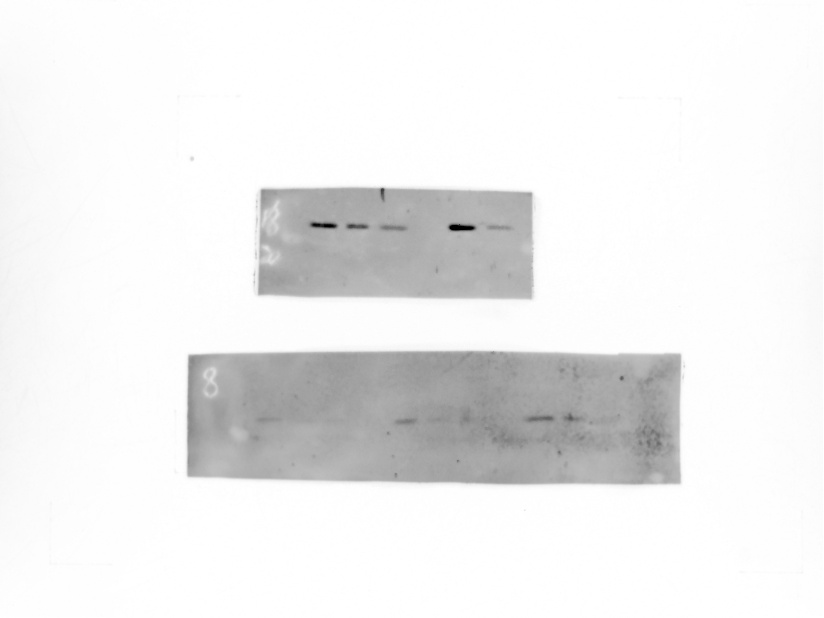

Supplement: Figure 4—figure supplement 1—source data 1. [file elife-92236-fig4-figsupp1-data1.zip › Figure_4-Figure Supplement_1-source_data_1/Figure_4-figure supplement_1_ source_data_1_ Figure_C_AFR6(left panel).jpg]

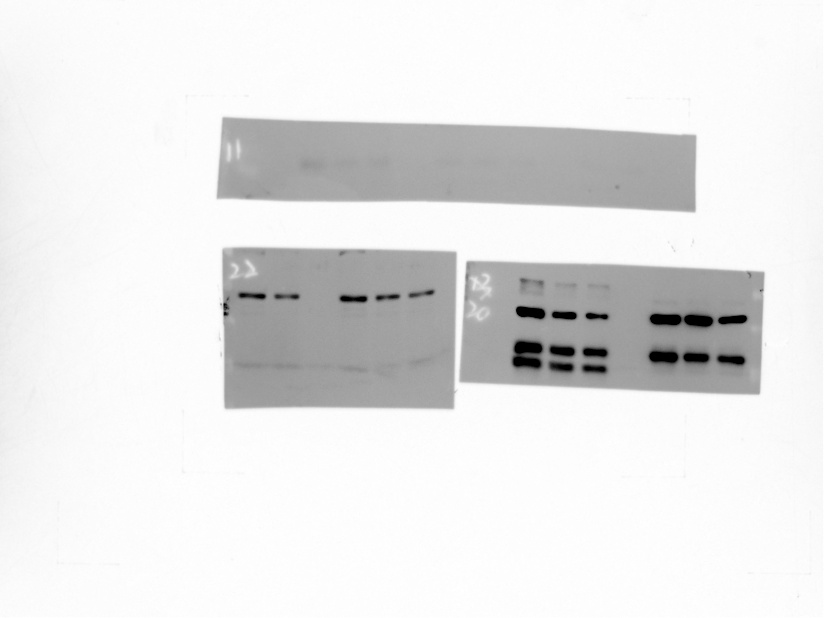

Supplement: Figure 4—figure supplement 1—source data 1. [file elife-92236-fig4-figsupp1-data1.zip › Figure_4-Figure Supplement_1-source_data_1/Figure_4-figure supplement_1_ source_data_1_ Figure_C_CCND3(left panel).jpg]

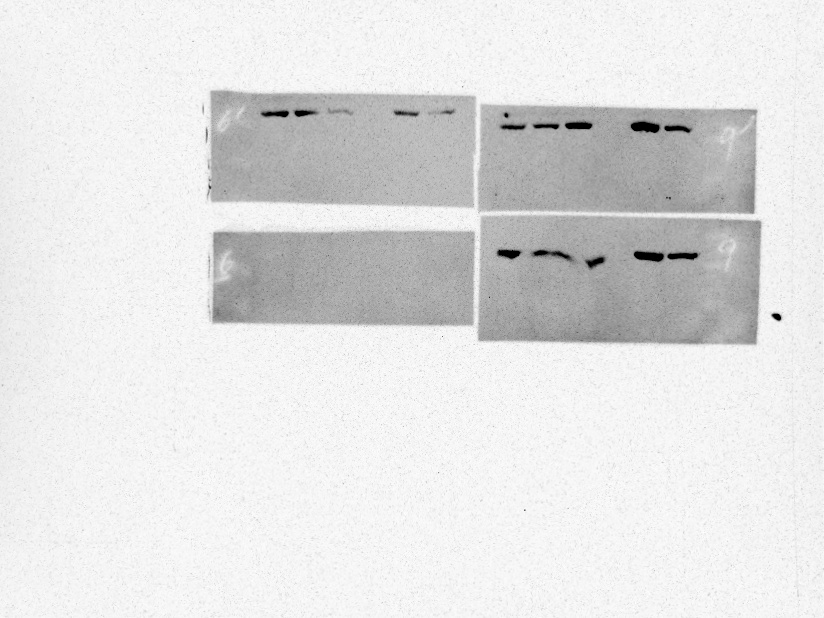

Supplement: Figure 4—figure supplement 1—source data 1. [file elife-92236-fig4-figsupp1-data1.zip › Figure_4-Figure Supplement_1-source_data_1/Figure_4-figure supplement_1_ source_data_1_ Figure_C_CDK4(left panel).jpg]

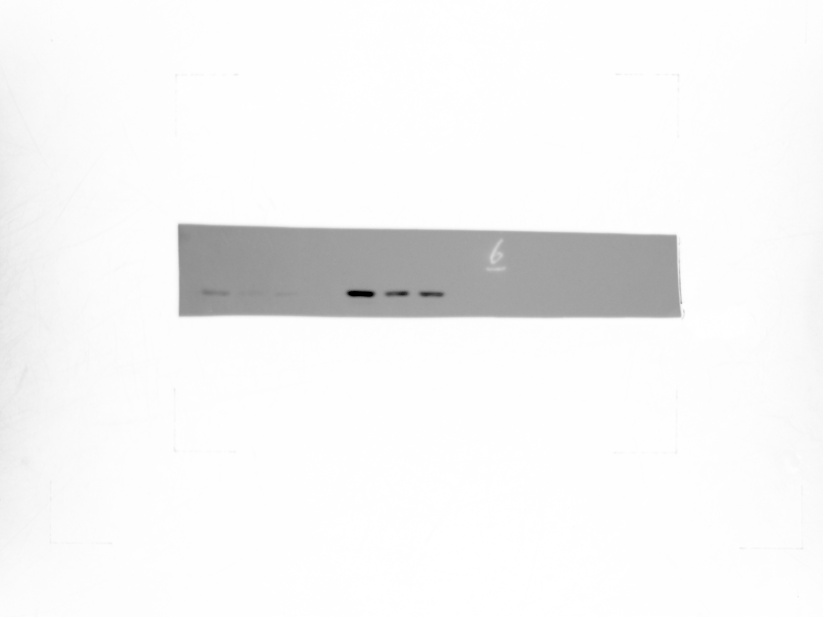

Supplement: Figure 4—figure supplement 1—source data 1. [file elife-92236-fig4-figsupp1-data1.zip › Figure_4-Figure Supplement_1-source_data_1/Figure_4-figure supplement_1_ source_data_1_ Figure_C_CDK6(right panel).jpg]

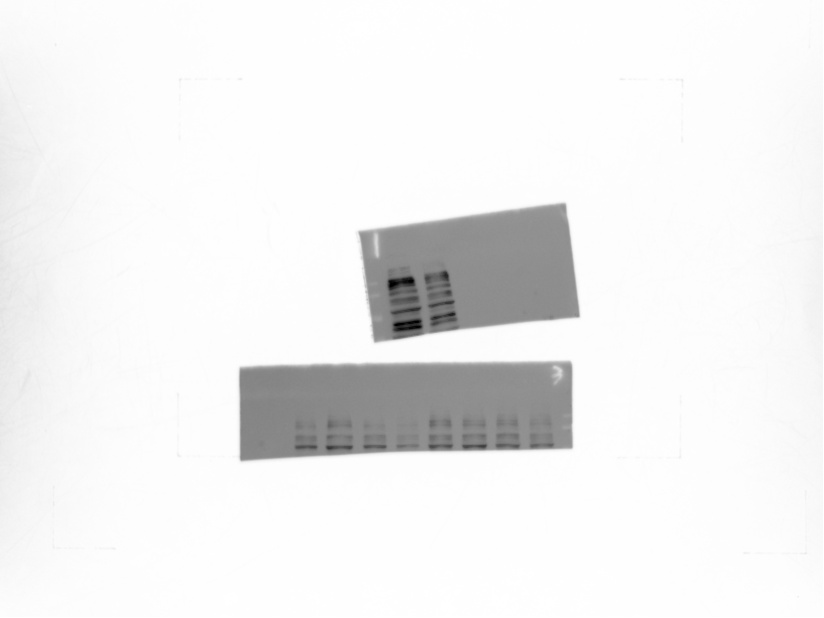

Supplement: Figure 4—figure supplement 1—source data 1. [file elife-92236-fig4-figsupp1-data1.zip › Figure_4-Figure Supplement_1-source_data_1/Figure_4-figure supplement_1_ source_data_1_ Figure_C_IBTK (left panel).jpg]

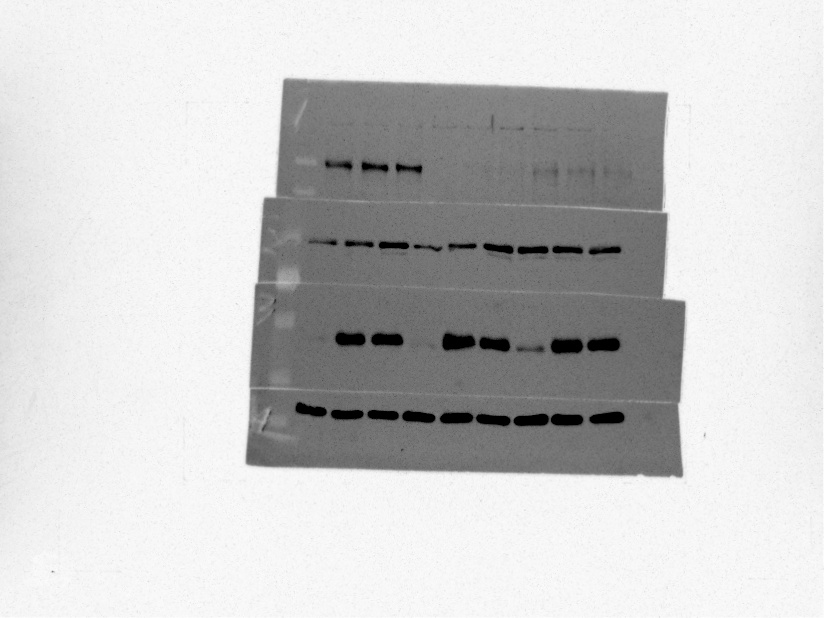

Supplement: Figure 4—figure supplement 1—source data 1. [file elife-92236-fig4-figsupp1-data1.zip › Figure_4-Figure Supplement_1-source_data_1/Figure_4-figure supplement_1_ source_data_1_ Figure_C_IBTK(right panel).jpg]

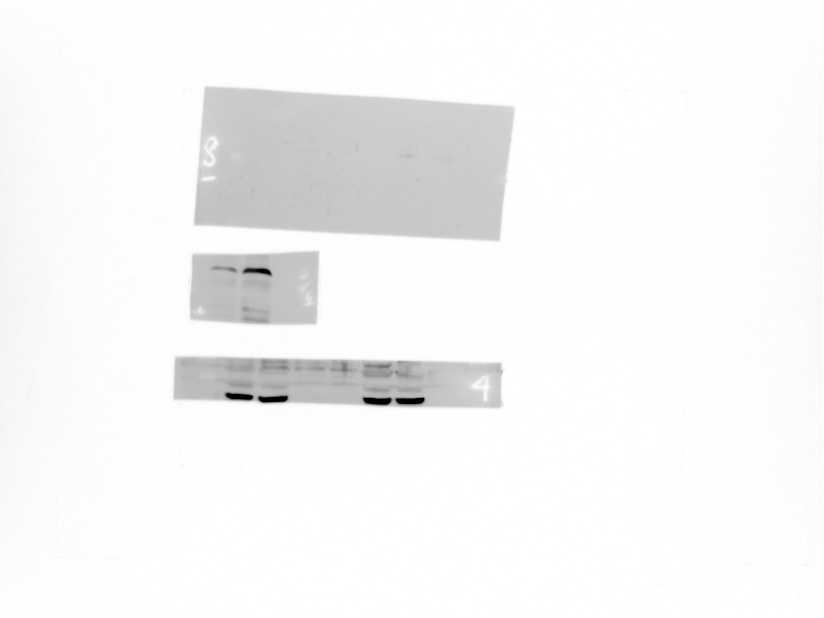

Supplement: Figure 4—figure supplement 1—source data 1. [file elife-92236-fig4-figsupp1-data1.zip › Figure_4-Figure Supplement_1-source_data_1/Figure_4-figure supplement_1_ source_data_1_ Figure_C_MYC(left panel).jpg]

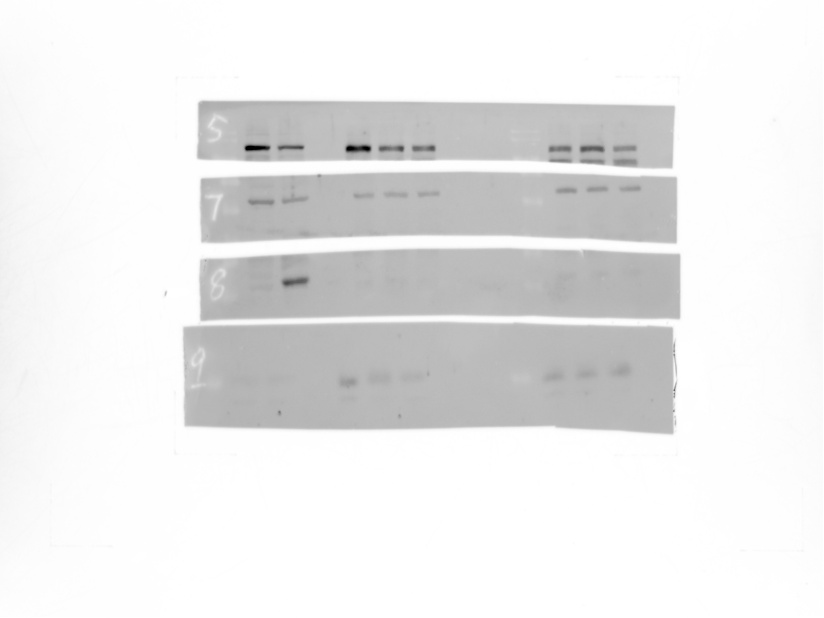

Supplement: Figure 4—figure supplement 1—source data 1. [file elife-92236-fig4-figsupp1-data1.zip › Figure_4-Figure Supplement_1-source_data_1/Figure_4-figure supplement_1_ source_data_1_ Figure_C_NOTCH1(left panel).jpg]

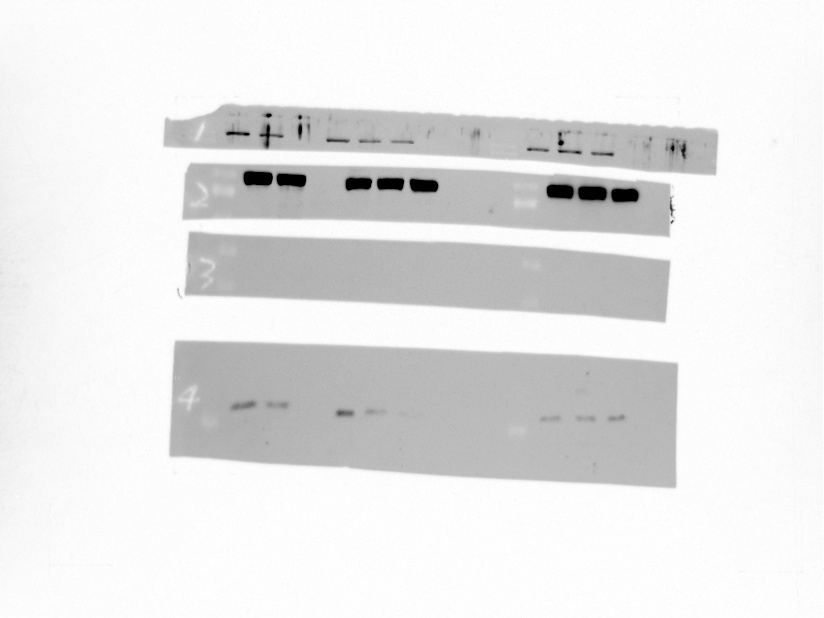

Supplement: Figure 4—figure supplement 1—source data 1. [file elife-92236-fig4-figsupp1-data1.zip › Figure_4-Figure Supplement_1-source_data_1/Figure_4-figure supplement_1_ source_data_1_ Figure_C_ROCK1(right panel).jpg]

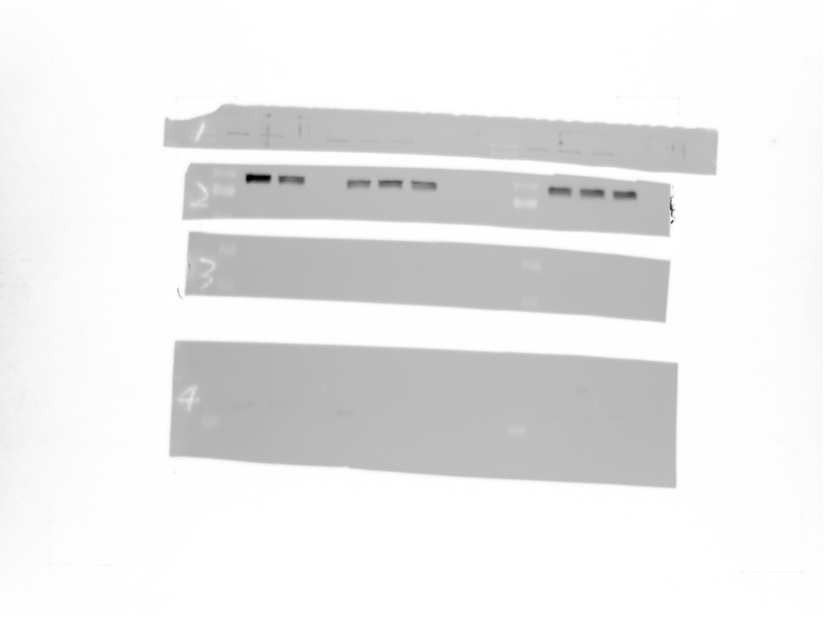

Supplement: Figure 4—figure supplement 1—source data 1. [file elife-92236-fig4-figsupp1-data1.zip › Figure_4-Figure Supplement_1-source_data_1/Figure_4-figure supplement_1_ source_data_1_ Figure_C_STAT1(left panel).jpg]

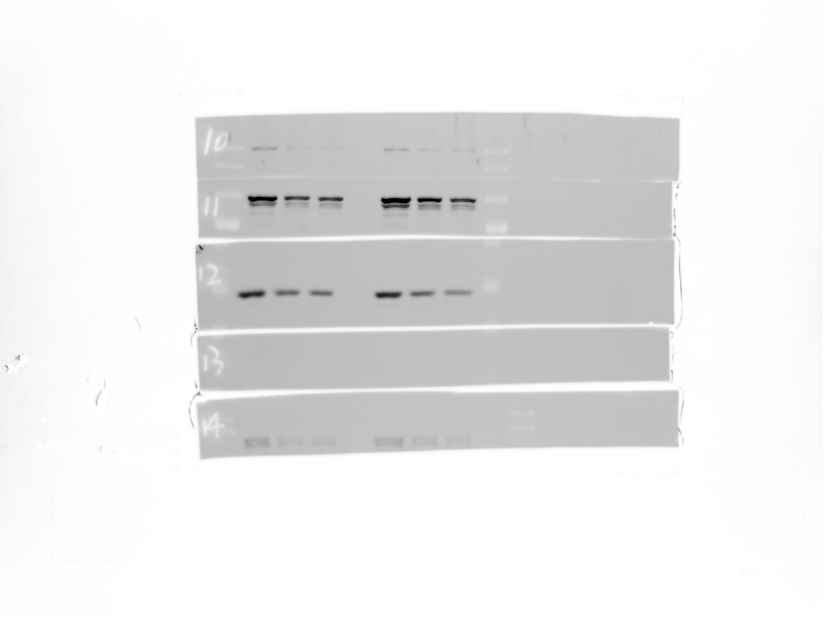

Supplement: Figure 4—figure supplement 1—source data 1. [file elife-92236-fig4-figsupp1-data1.zip › Figure_4-Figure Supplement_1-source_data_1/Figure_4-figure supplement_1_ source_data_1_ Figure_C_STAT1(right panel) (2).jpg]

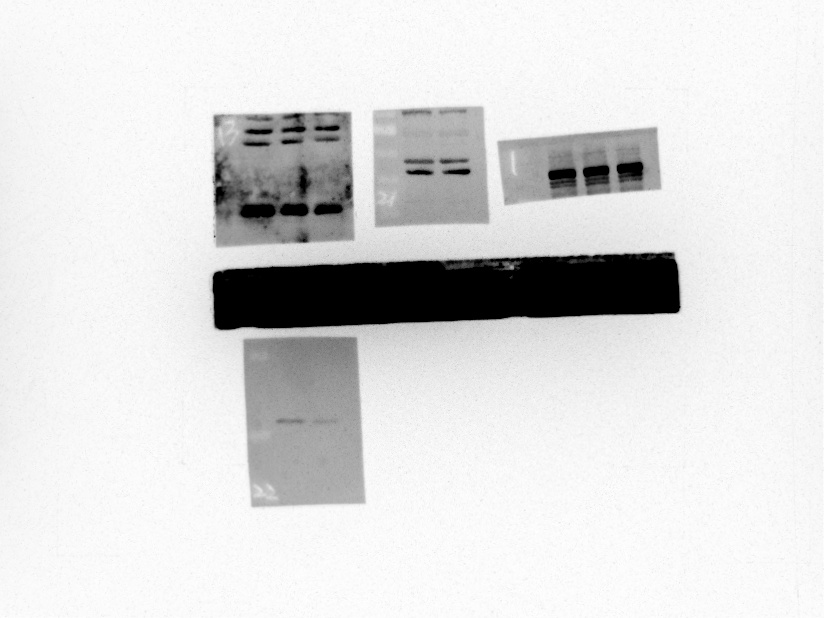

Supplement: Figure 4—figure supplement 1—source data 1. [file elife-92236-fig4-figsupp1-data1.zip › Figure_4-Figure Supplement_1-source_data_1/Figure_4-figure supplement_1_ source_data_1_ Figure_C_XIAP(left panel).jpg]

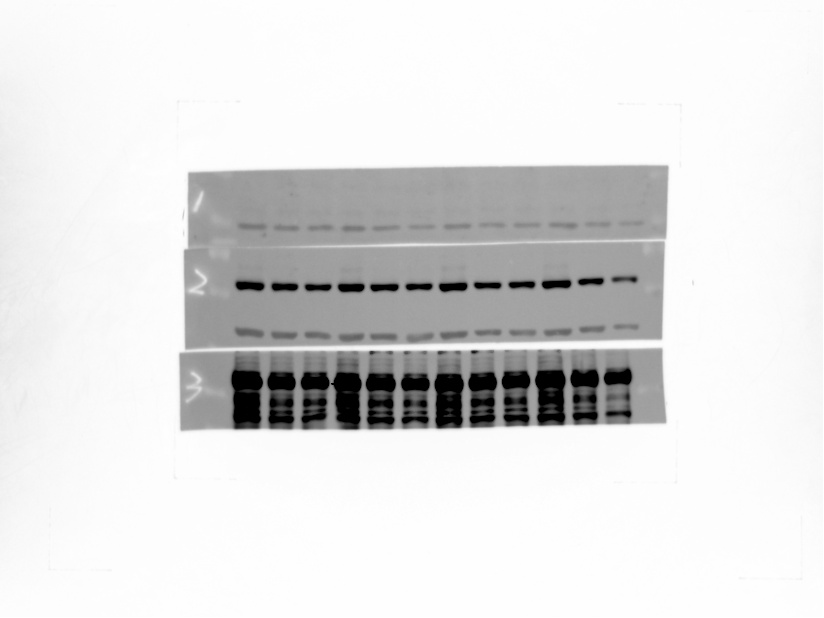

Supplement: Figure 4—figure supplement 1—source data 1. [file elife-92236-fig4-figsupp1-data1.zip › Figure_4-Figure Supplement_1-source_data_1/Figure_4-figure supplement_1_ source_data_1_ Figure_C_XIAP(right panel).jpg]

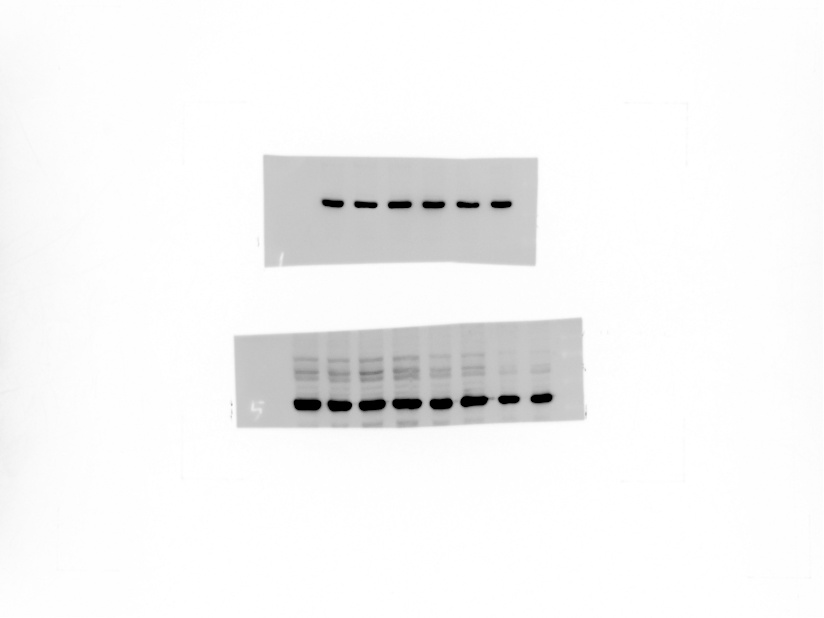

Supplement: Figure 4—figure supplement 1—source data 1. [file elife-92236-fig4-figsupp1-data1.zip › Figure_4-Figure Supplement_1-source_data_1/Figure_4-figure supplement_1_ source_data_1_ Figure_E_Actin.jpg]

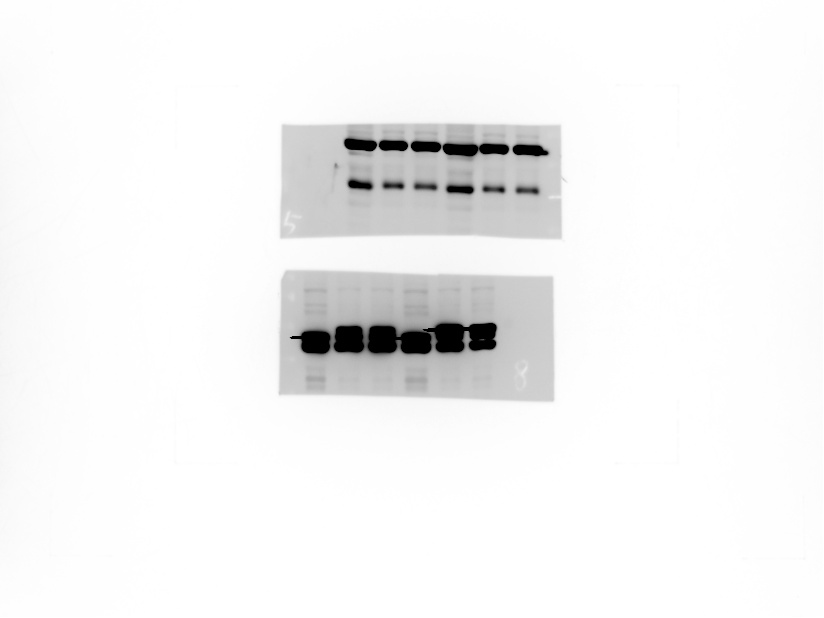

Supplement: Figure 4—figure supplement 1—source data 1. [file elife-92236-fig4-figsupp1-data1.zip › Figure_4-Figure Supplement_1-source_data_1/Figure_4-figure supplement_1_ source_data_1_ Figure_E_CCND3.jpg]

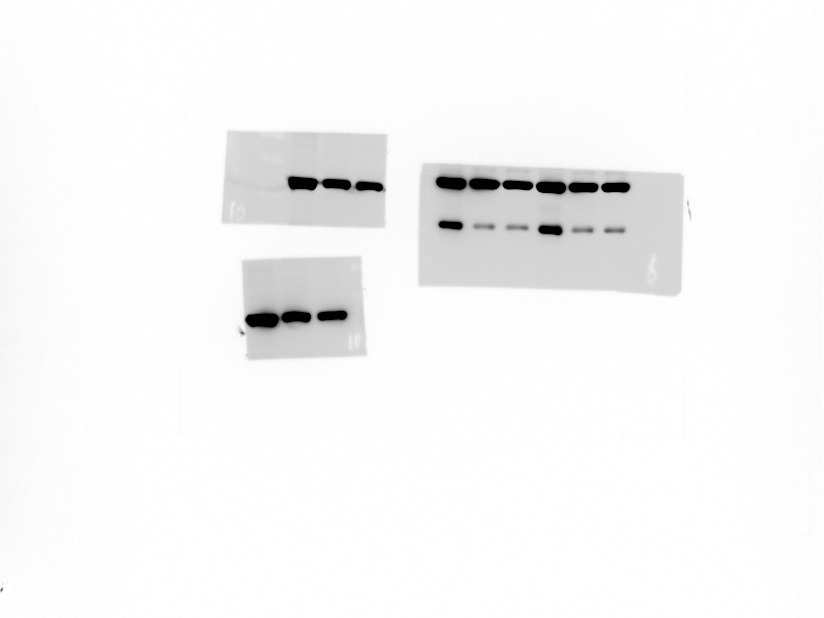

Supplement: Figure 4—figure supplement 1—source data 1. [file elife-92236-fig4-figsupp1-data1.zip › Figure_4-Figure Supplement_1-source_data_1/Figure_4-figure supplement_1_ source_data_1_ Figure_E_CDK4.jpg]

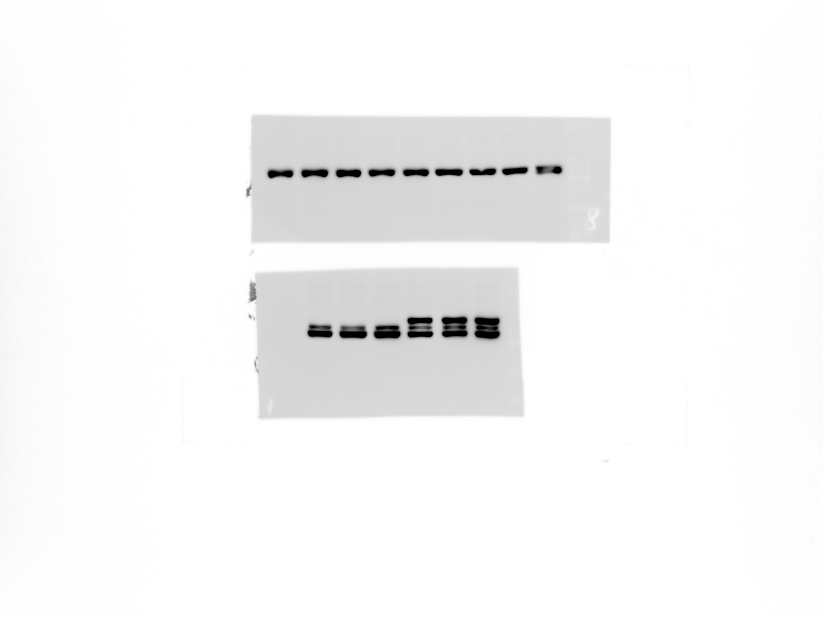

Supplement: Figure 4—figure supplement 1—source data 1. [file elife-92236-fig4-figsupp1-data1.zip › Figure_4-Figure Supplement_1-source_data_1/Figure_4-figure supplement_1_ source_data_1_ Figure_E_eIF4A1.jpg]

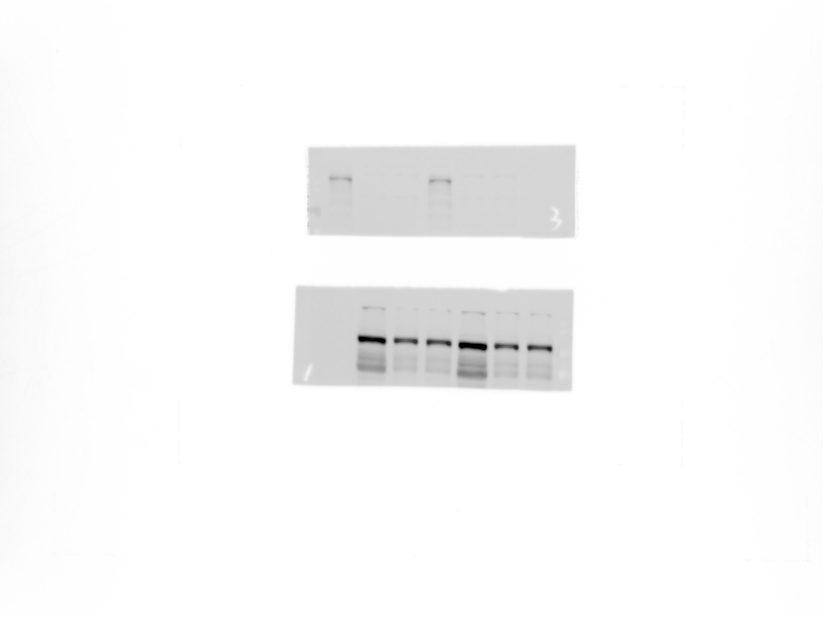

Supplement: Figure 4—figure supplement 1—source data 1. [file elife-92236-fig4-figsupp1-data1.zip › Figure_4-Figure Supplement_1-source_data_1/Figure_4-figure supplement_1_ source_data_1_ Figure_E_EZH2.jpg]

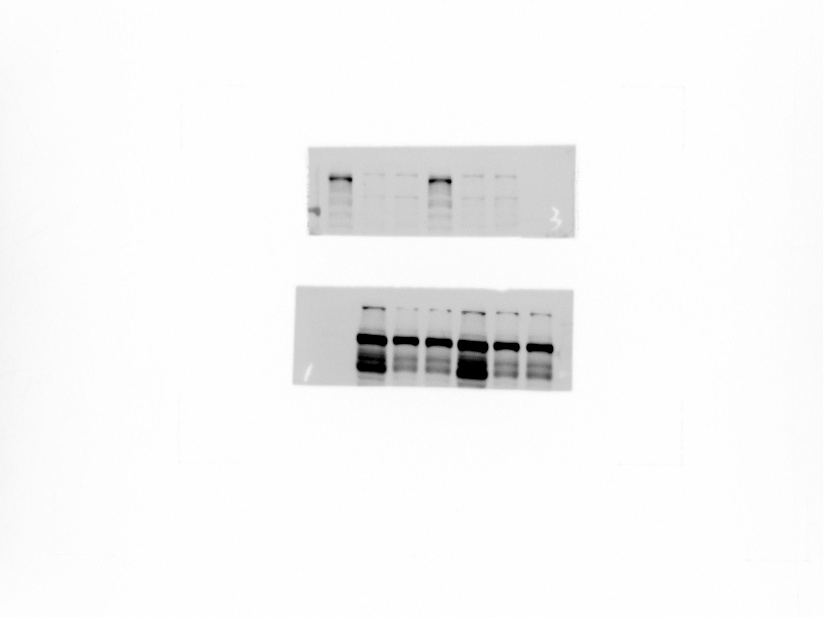

Supplement: Figure 4—figure supplement 1—source data 1. [file elife-92236-fig4-figsupp1-data1.zip › Figure_4-Figure Supplement_1-source_data_1/Figure_4-figure supplement_1_ source_data_1_ Figure_E_IBTK.jpg]

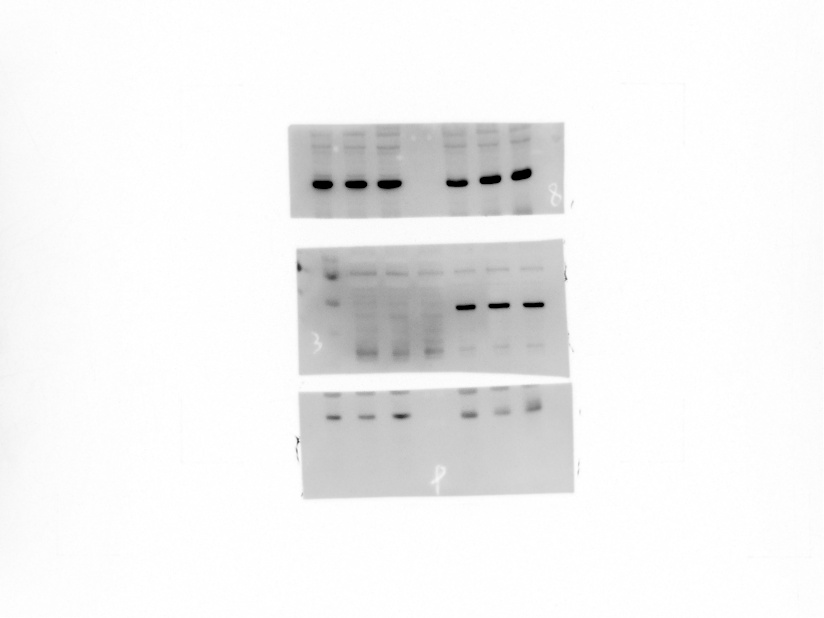

Supplement: Figure 4—figure supplement 1—source data 1. [file elife-92236-fig4-figsupp1-data1.zip › Figure_4-Figure Supplement_1-source_data_1/Figure_4-figure supplement_1_ source_data_1_ Figure_E_Myc.jpg]

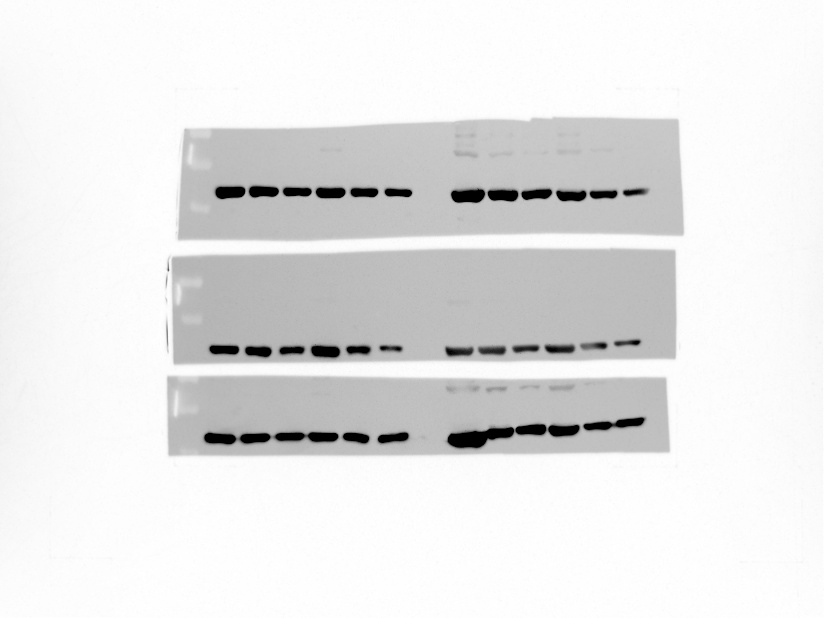

Supplement: Figure 4—figure supplement 2—source data 1. [file elife-92236-fig4-figsupp2-data1.zip › Figure_4-Figure Supplement_2_source_data_1/Figure_4-figure supplement_2_ source_data_1_ Figure_A_CASP7.jpg]

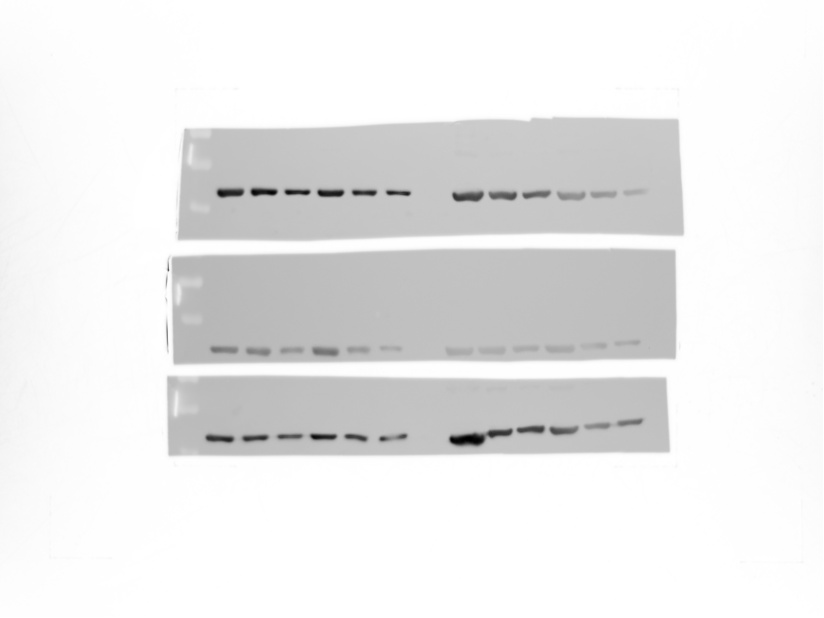

Supplement: Figure 4—figure supplement 2—source data 1. [file elife-92236-fig4-figsupp2-data1.zip › Figure_4-Figure Supplement_2_source_data_1/Figure_4-figure supplement_2_ source_data_1_ Figure_A_CASP9.jpg]

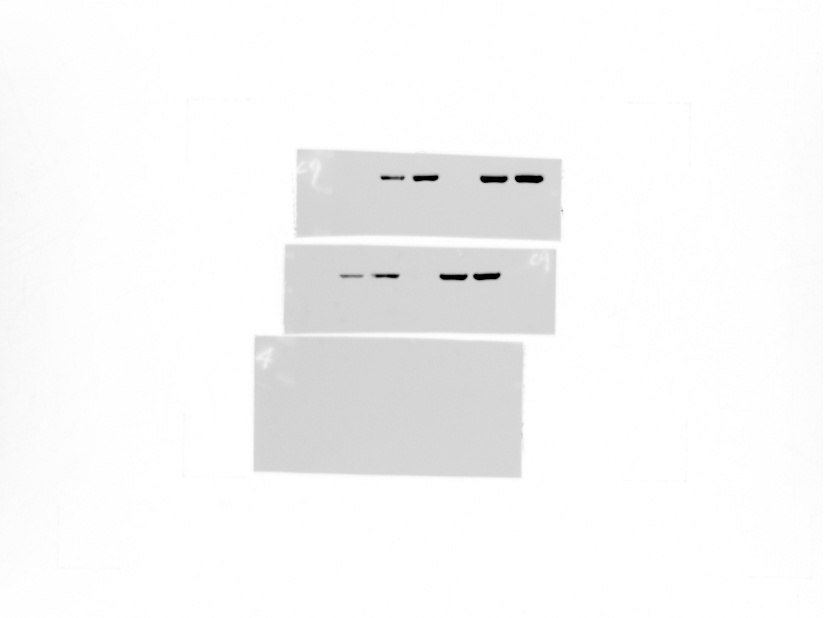

Supplement: Figure 4—figure supplement 2—source data 1. [file elife-92236-fig4-figsupp2-data1.zip › Figure_4-Figure Supplement_2_source_data_1/Figure_4-figure supplement_2_ source_data_1_ Figure_A_cl-CASP7.jpg]

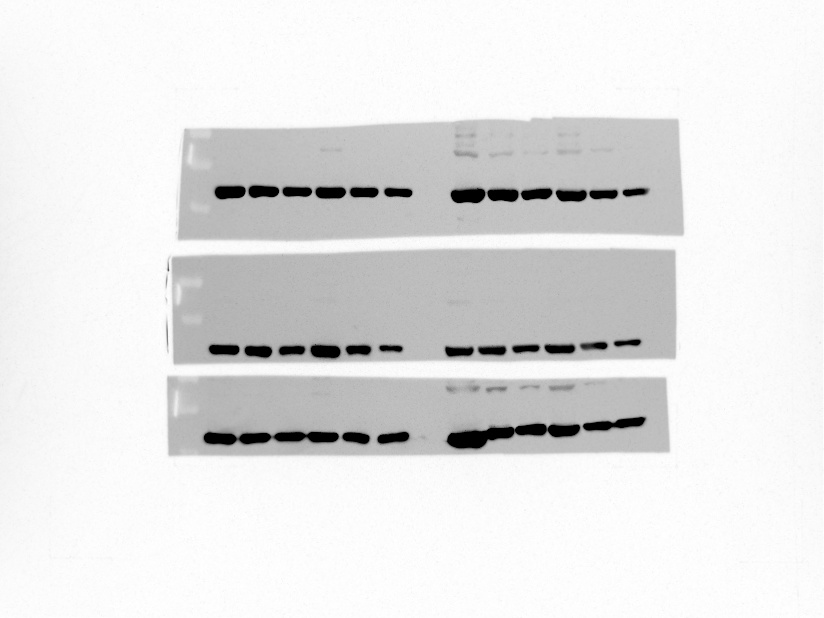

Supplement: Figure 4—figure supplement 2—source data 1. [file elife-92236-fig4-figsupp2-data1.zip › Figure_4-Figure Supplement_2_source_data_1/Figure_4-figure supplement_2_ source_data_1_ Figure_G_CASP9.jpg]

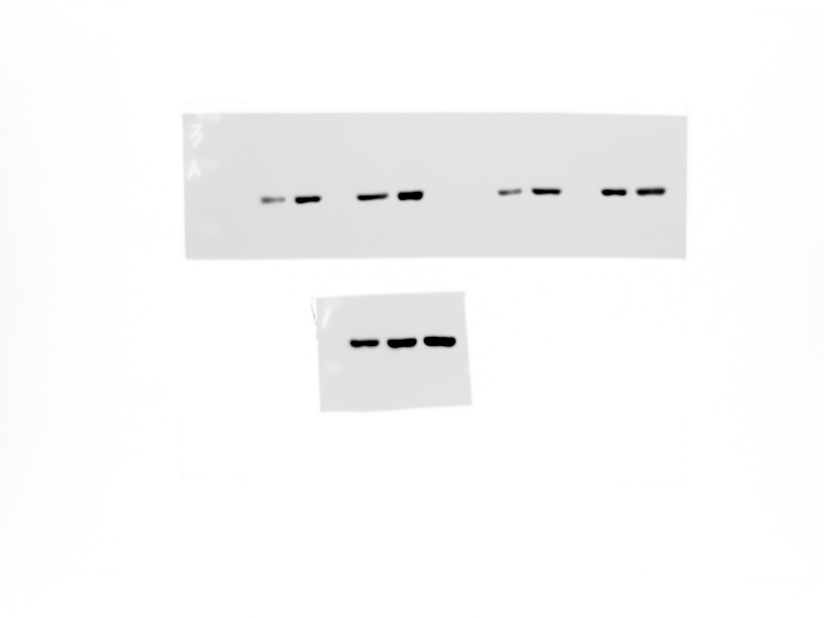

Supplement: Figure 4—figure supplement 2—source data 1. [file elife-92236-fig4-figsupp2-data1.zip › Figure_4-Figure Supplement_2_source_data_1/Figure_4-figure supplement_2_ source_data_1_ Figure_G_cl-CASP7.jpg]

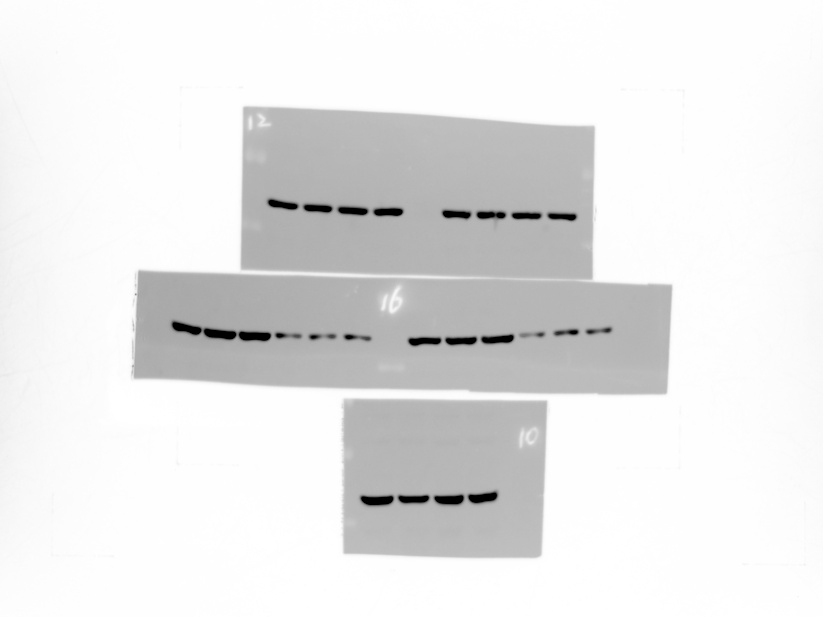

Supplement: Figure 4—figure supplement 2—source data 1. [file elife-92236-fig4-figsupp2-data1.zip › Figure_4-Figure Supplement_2_source_data_1/Figure_4-figure supplement_2_ source_data_1_ Figure_G_IBTK.jpg]

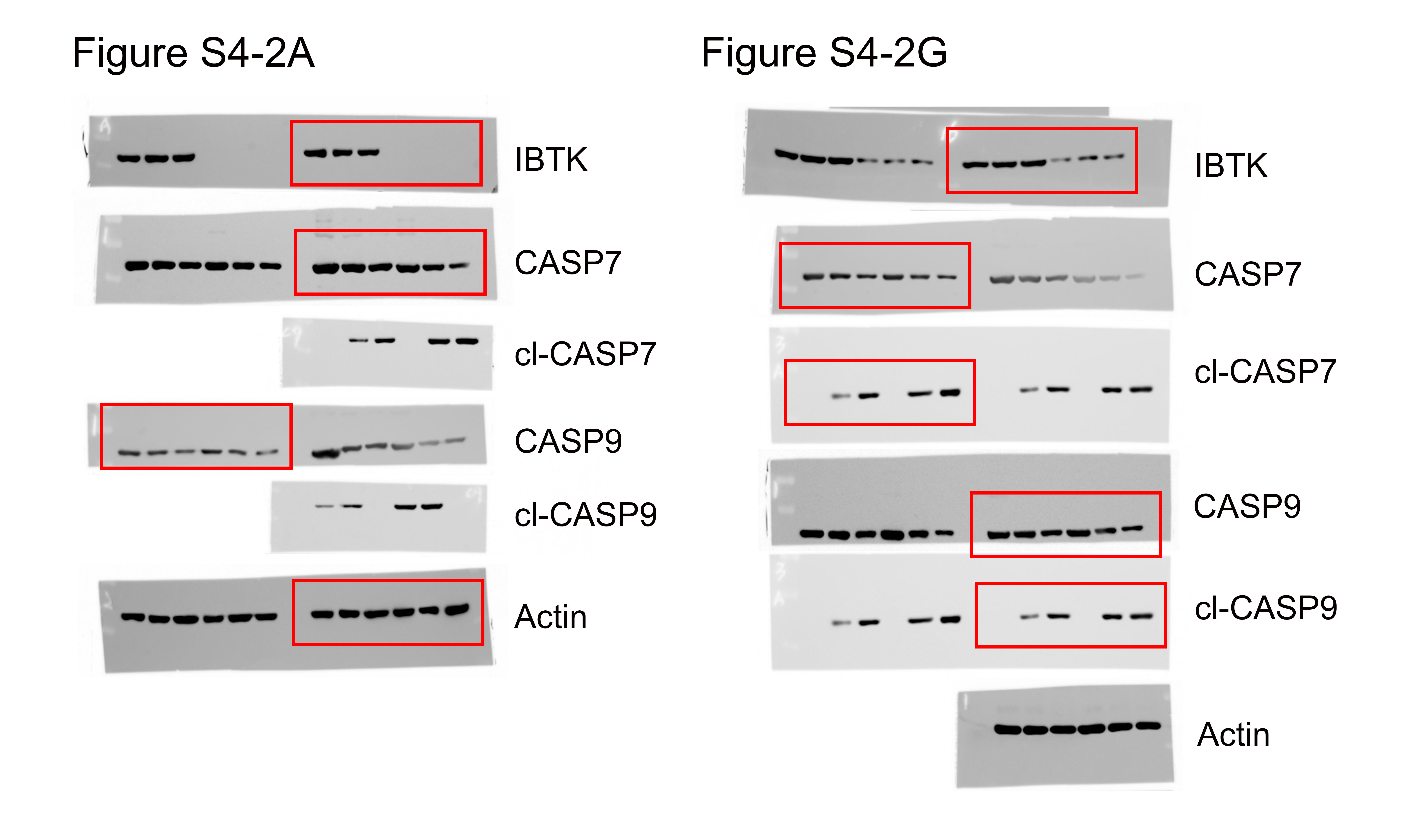

Supplement: Figure 4—figure supplement 2—source data 2. [file elife-92236-fig4-figsupp2-data2.zip › Figure_4-Figure Supplement_2-source_data_2.jpg]

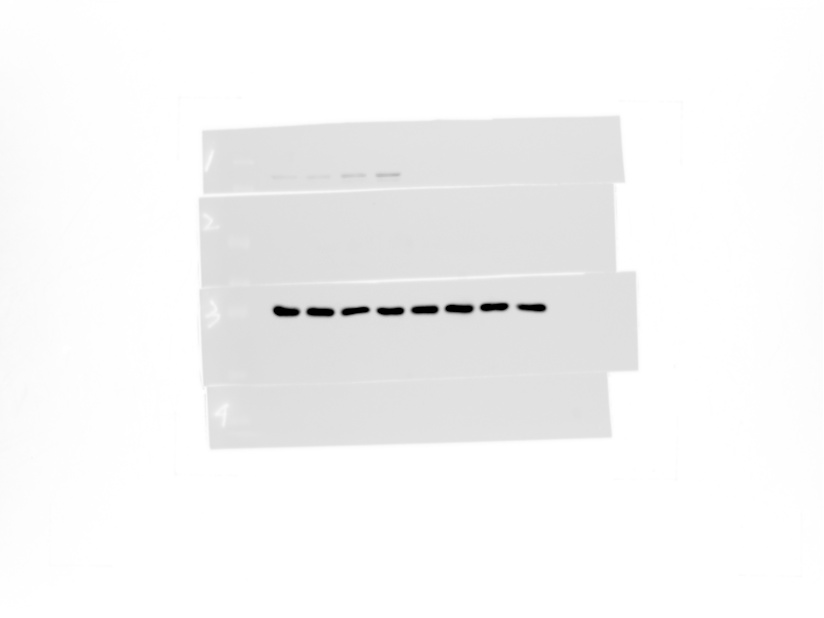

Supplement: Figure 4—figure supplement 3—source data 1. [file elife-92236-fig4-figsupp3-data1.zip › Figure_4-Figure Supplement_3_source_data_1/Figure_4-figure supplement_3_ source_data_1_ Figure_A_GAPDH.jpg]

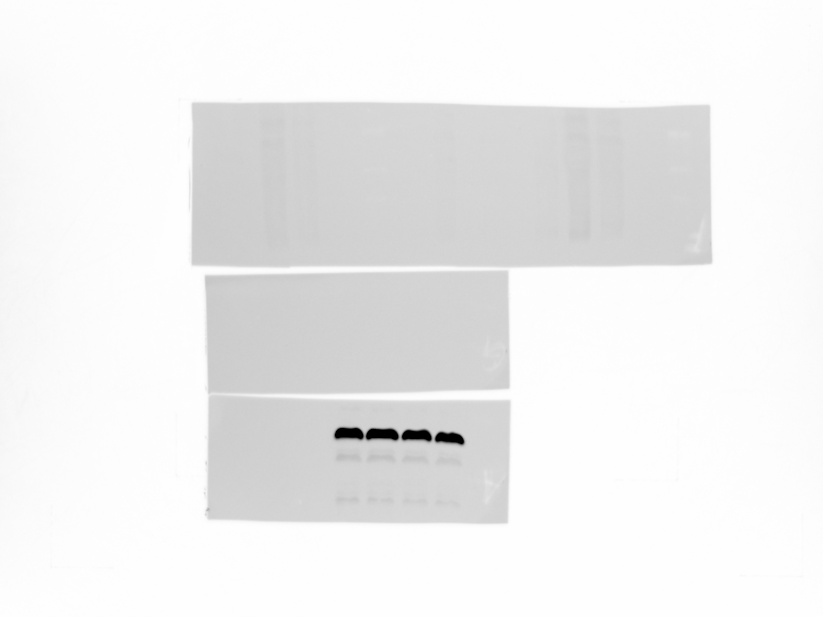

Supplement: Figure 4—figure supplement 3—source data 1. [file elife-92236-fig4-figsupp3-data1.zip › Figure_4-Figure Supplement_3_source_data_1/Figure_4-figure supplement_3_ source_data_1_ Figure_A_IBTK.jpg]

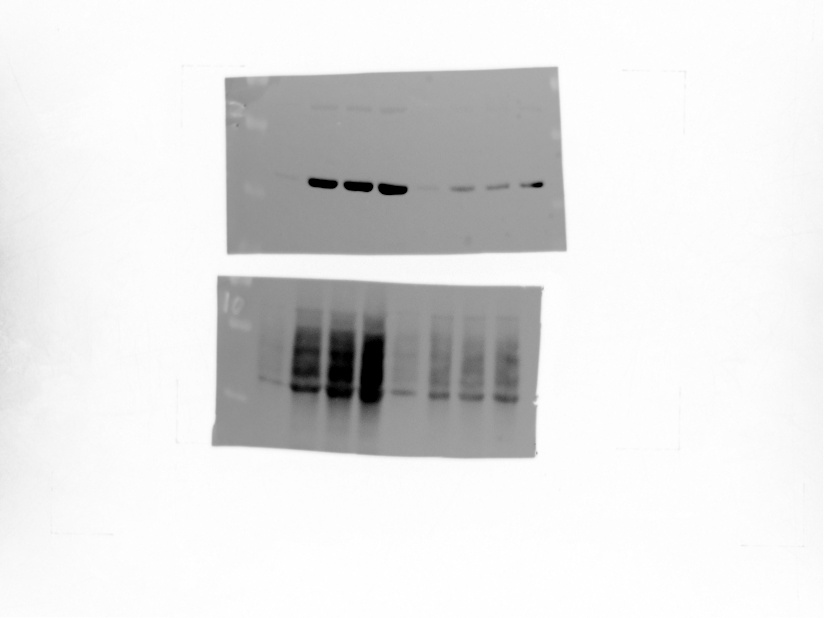

Supplement: Figure 4—figure supplement 3—source data 1. [file elife-92236-fig4-figsupp3-data1.zip › Figure_4-Figure Supplement_3_source_data_1/Figure_4-figure supplement_3_ source_data_1_ Figure_A_IRF1.jpg]
