# Supplementary figures and images for: mTORC1/S6K1 signaling promotes sustained oncogenic translation through modulating CRL3IBTK-mediated ubiquitination of eIF4A1 in cancer cells (part 3 of 3)
Source: eLife. 2024 May 13;12:RP92236. doi: 10.7554/eLife.92236 (PMC11090508; doi:10.7554/eLife.92236)

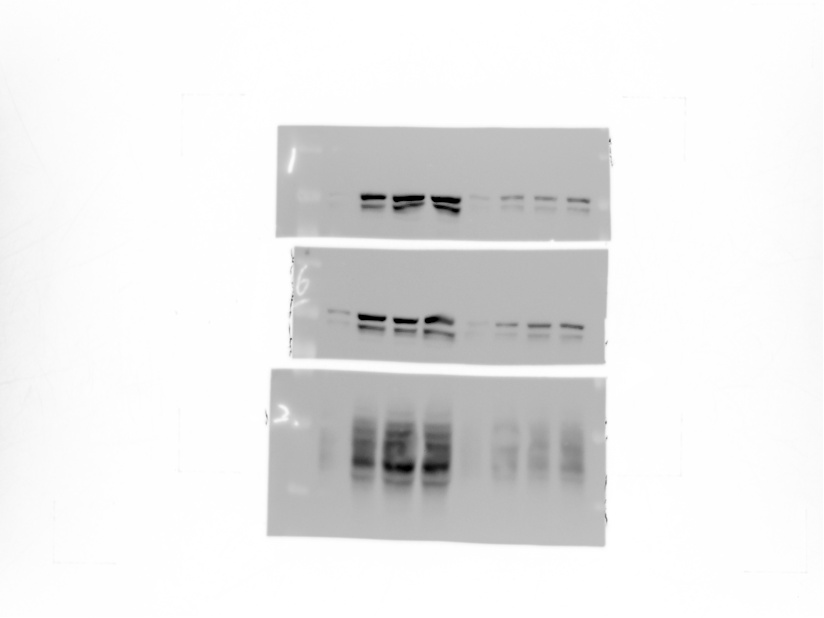

Supplement: Figure 4—figure supplement 3—source data 1. [file elife-92236-fig4-figsupp3-data1.zip › Figure_4-Figure Supplement_3_source_data_1/Figure_4-figure supplement_3_ source_data_1_ Figure_A_STAT1.jpg]

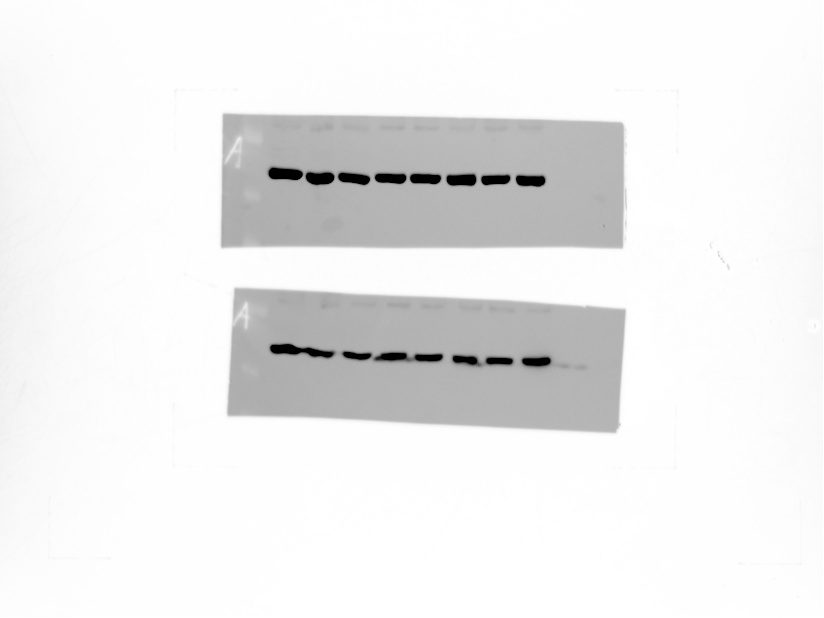

Supplement: Figure 4—figure supplement 3—source data 1. [file elife-92236-fig4-figsupp3-data1.zip › Figure_4-Figure Supplement_3_source_data_1/Figure_4-figure supplement_3_ source_data_1_ Figure_D_GAPDH.jpg]

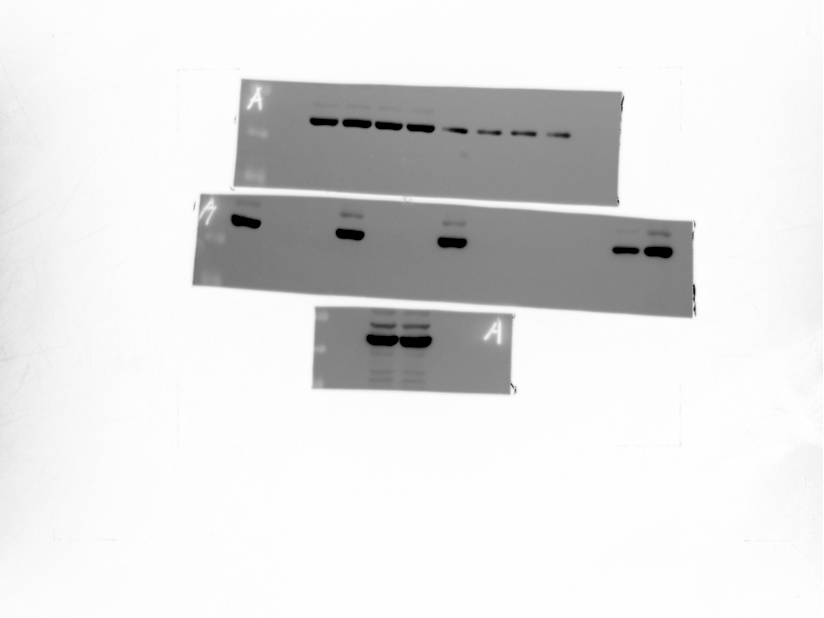

Supplement: Figure 4—figure supplement 3—source data 1. [file elife-92236-fig4-figsupp3-data1.zip › Figure_4-Figure Supplement_3_source_data_1/Figure_4-figure supplement_3_ source_data_1_ Figure_D_IBTK.jpg]

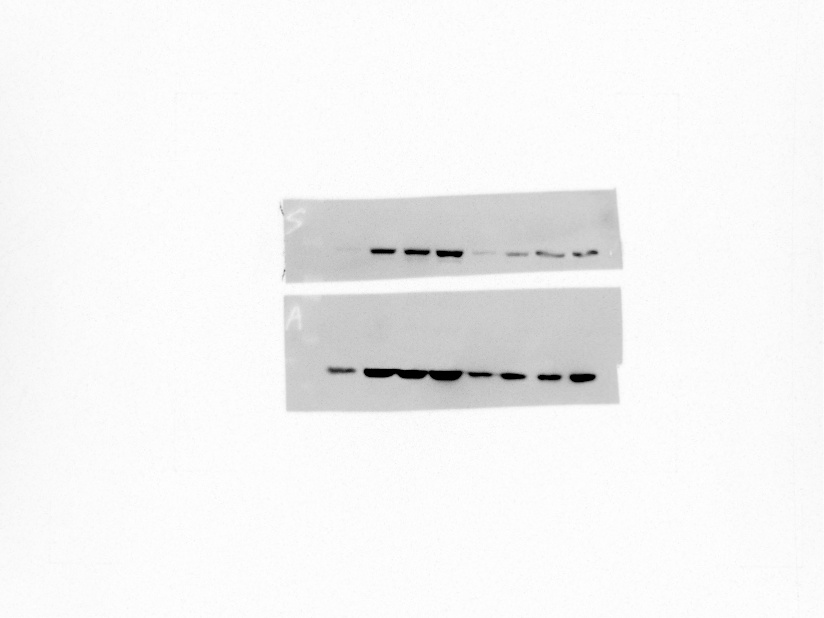

Supplement: Figure 4—figure supplement 3—source data 1. [file elife-92236-fig4-figsupp3-data1.zip › Figure_4-Figure Supplement_3_source_data_1/Figure_4-figure supplement_3_ source_data_1_ Figure_D_IRF1.jpg]

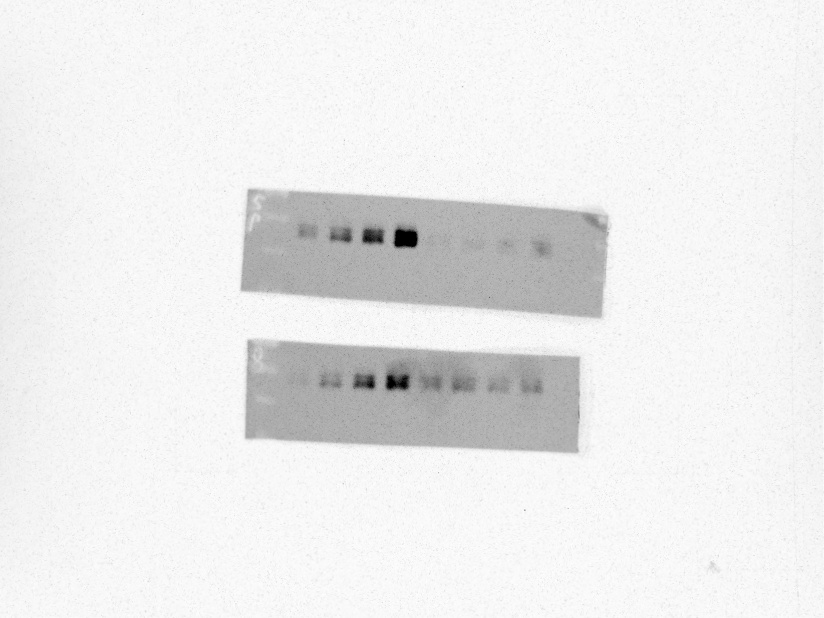

Supplement: Figure 4—figure supplement 3—source data 1. [file elife-92236-fig4-figsupp3-data1.zip › Figure_4-Figure Supplement_3_source_data_1/Figure_4-figure supplement_3_ source_data_1_ Figure_D_PD-L1.jpg]

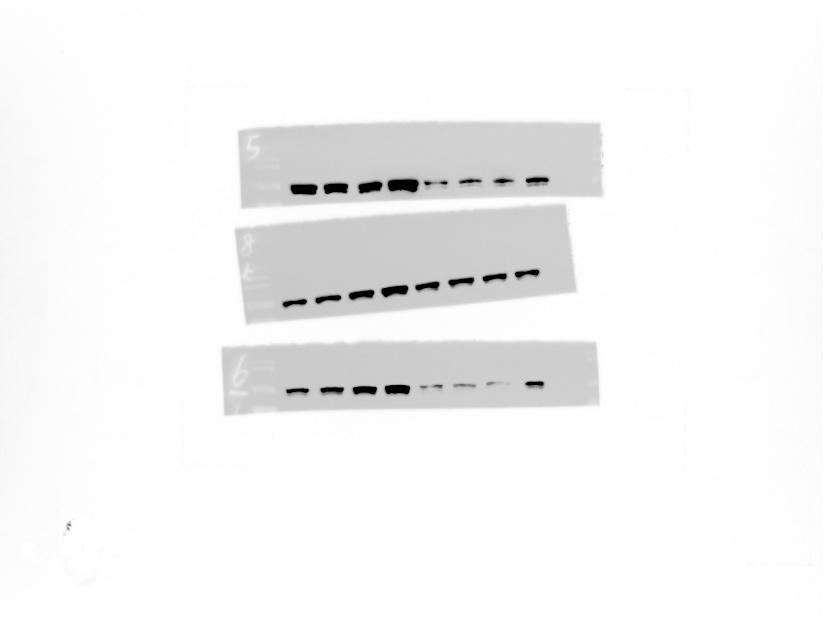

Supplement: Figure 4—figure supplement 3—source data 1. [file elife-92236-fig4-figsupp3-data1.zip › Figure_4-Figure Supplement_3_source_data_1/Figure_4-figure supplement_3_ source_data_1_ Figure_D_STAT1.jpg]

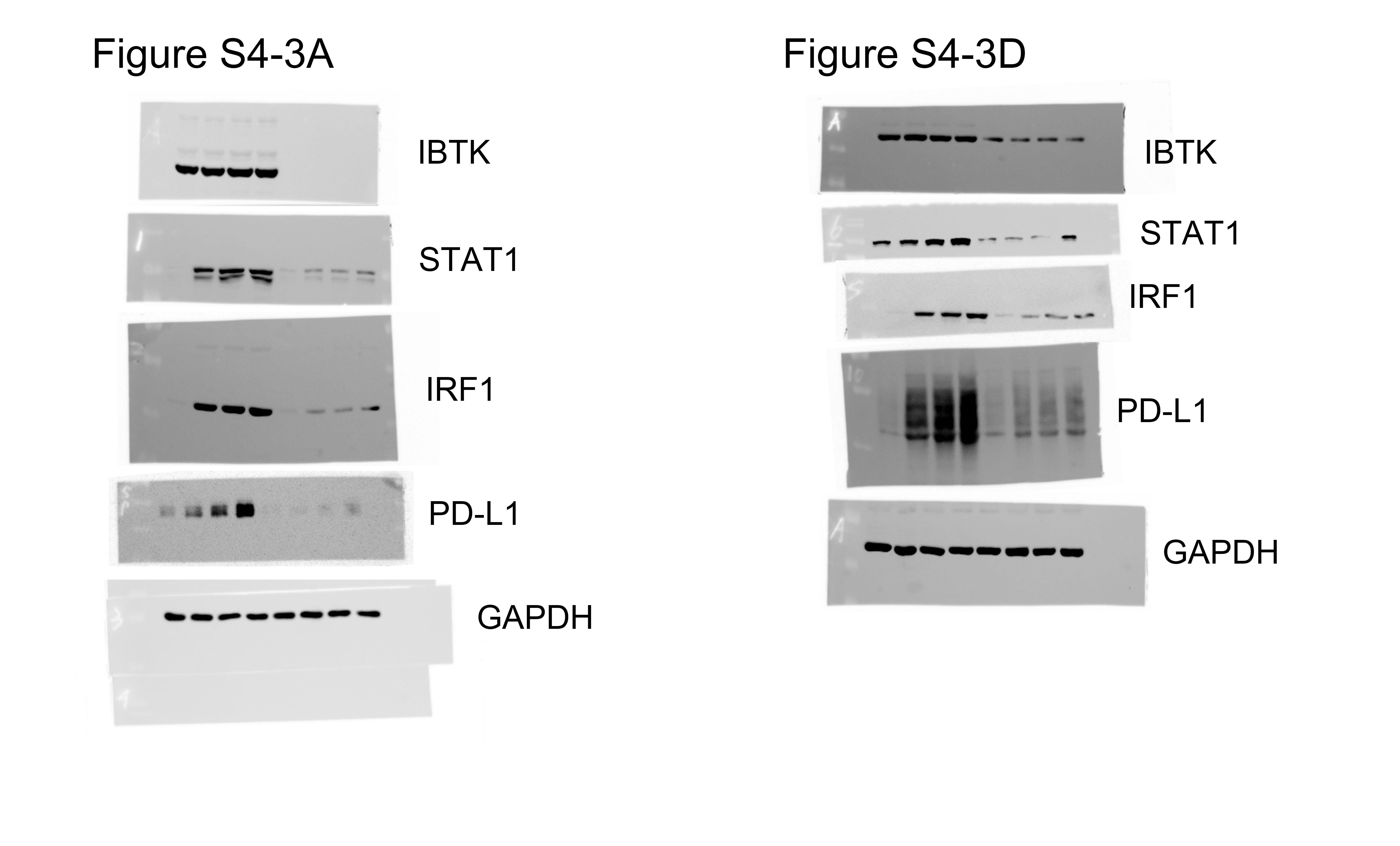

Supplement: Figure 4—figure supplement 3—source data 2. [file elife-92236-fig4-figsupp3-data2.zip › Figure_4-Figure Supplement_3-source_data_2.jpg]

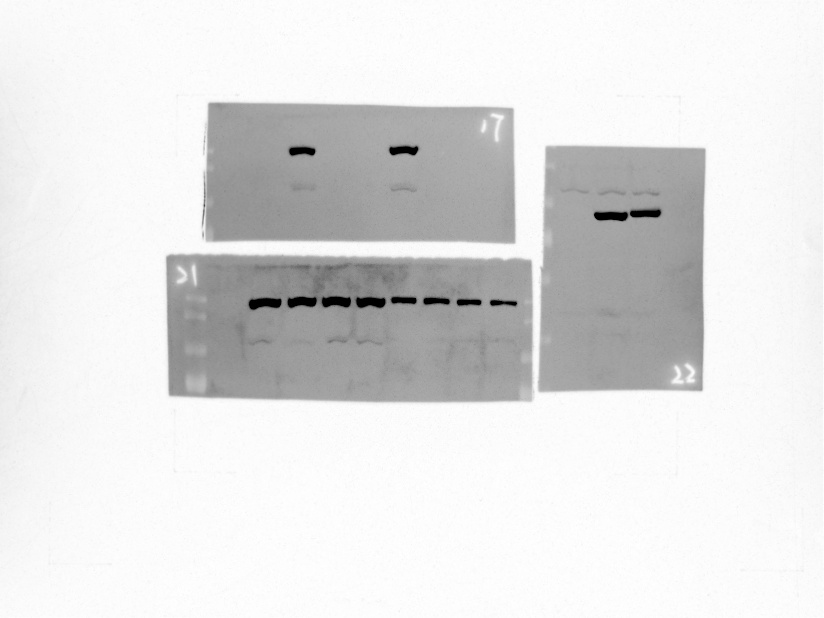

Supplement: Figure 5—source data 1. [file elife-92236-fig5-data1.zip › Figure_5-source_data_1/Figure_5-source_data_1_ Figure_5B_FLAG.jpg]

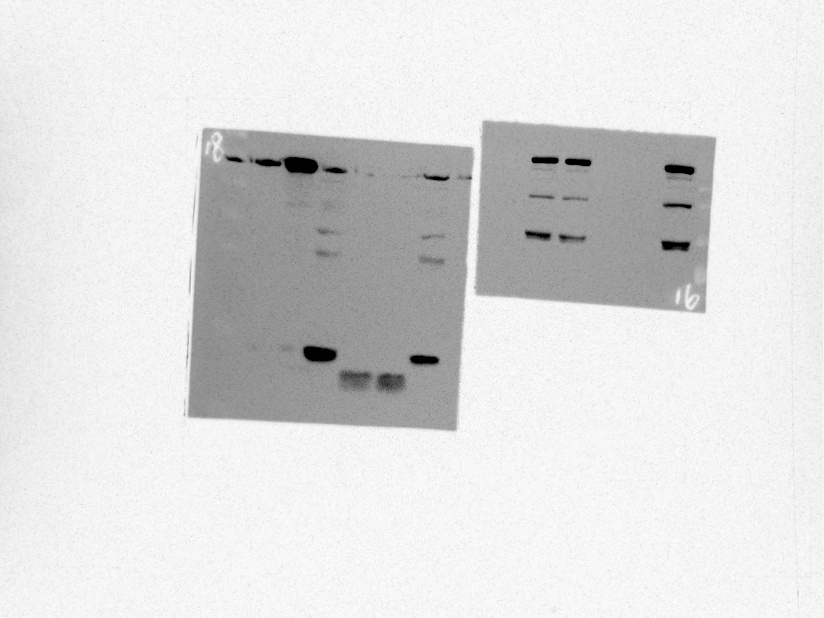

Supplement: Figure 5—source data 1. [file elife-92236-fig5-data1.zip › Figure_5-source_data_1/Figure_5-source_data_1_ Figure_5B_Myc.jpg]

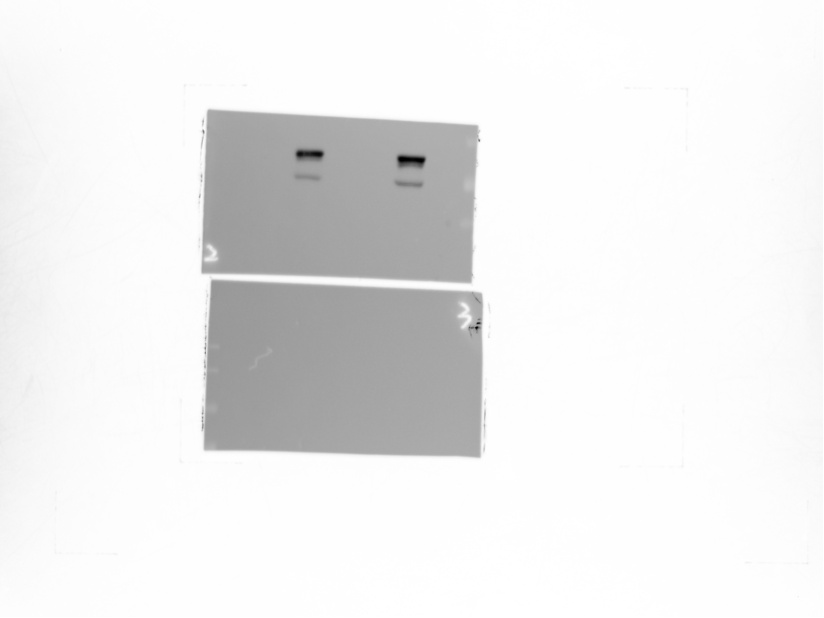

Supplement: Figure 5—source data 1. [file elife-92236-fig5-data1.zip › Figure_5-source_data_1/Figure_5-source_data_1_ Figure_5C_FLAG.jpg]

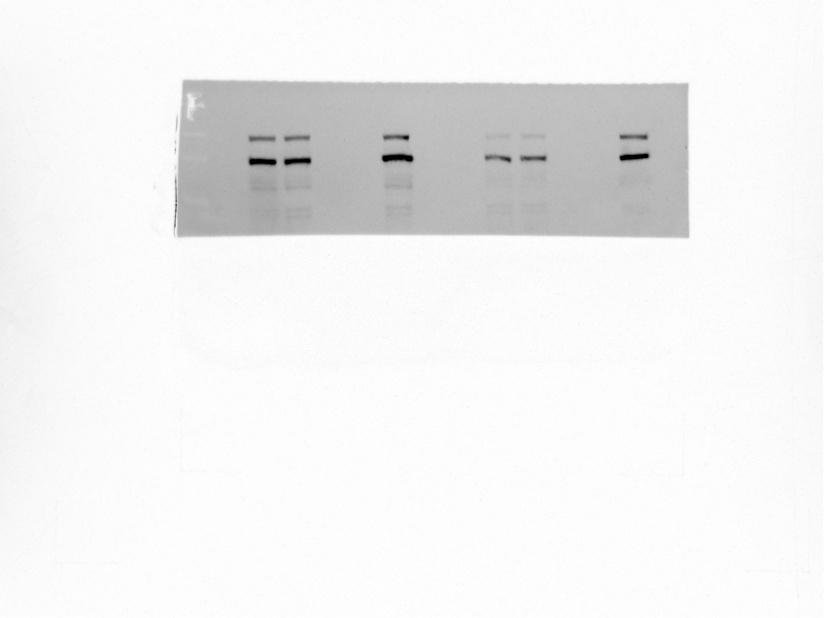

Supplement: Figure 5—source data 1. [file elife-92236-fig5-data1.zip › Figure_5-source_data_1/Figure_5-source_data_1_ Figure_5C_IBTK.jpg]

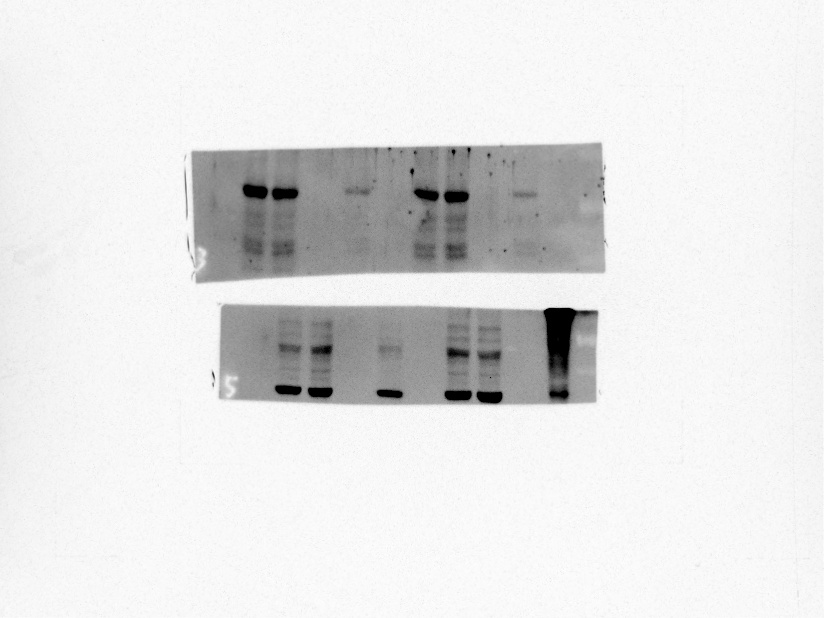

Supplement: Figure 5—source data 1. [file elife-92236-fig5-data1.zip › Figure_5-source_data_1/Figure_5-source_data_1_ Figure_5D_Deptor.jpg]

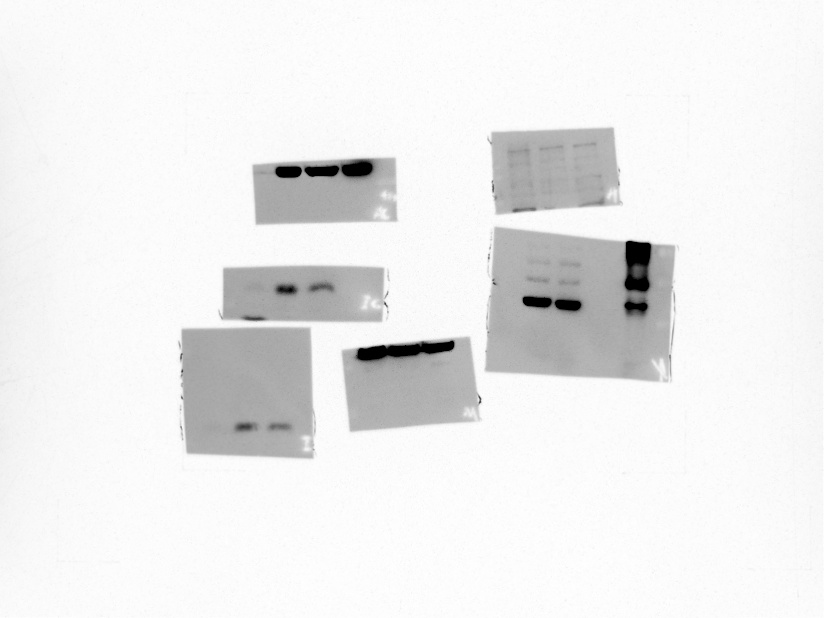

Supplement: Figure 5—source data 1. [file elife-92236-fig5-data1.zip › Figure_5-source_data_1/Figure_5-source_data_1_ Figure_5D_FLAG.jpg]

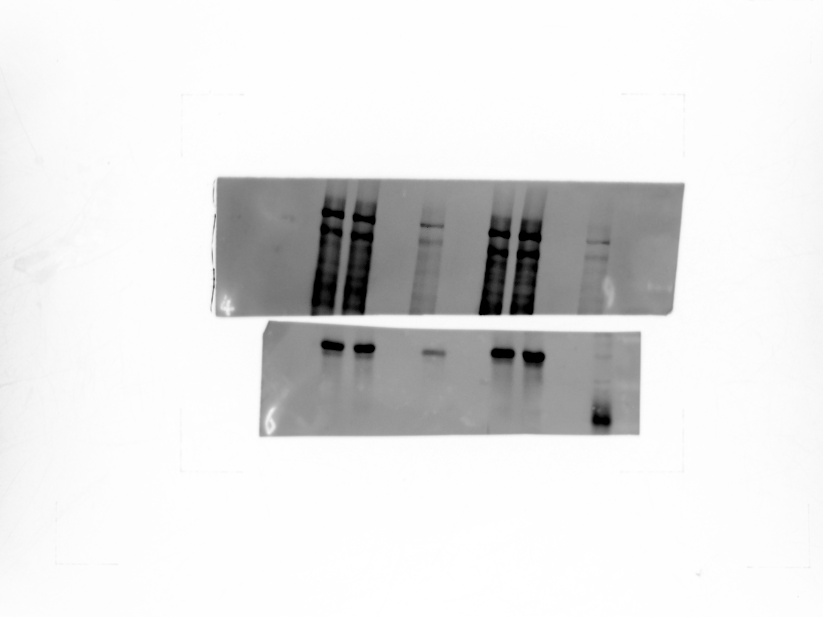

Supplement: Figure 5—source data 1. [file elife-92236-fig5-data1.zip › Figure_5-source_data_1/Figure_5-source_data_1_ Figure_5D_mLST8.jpg]

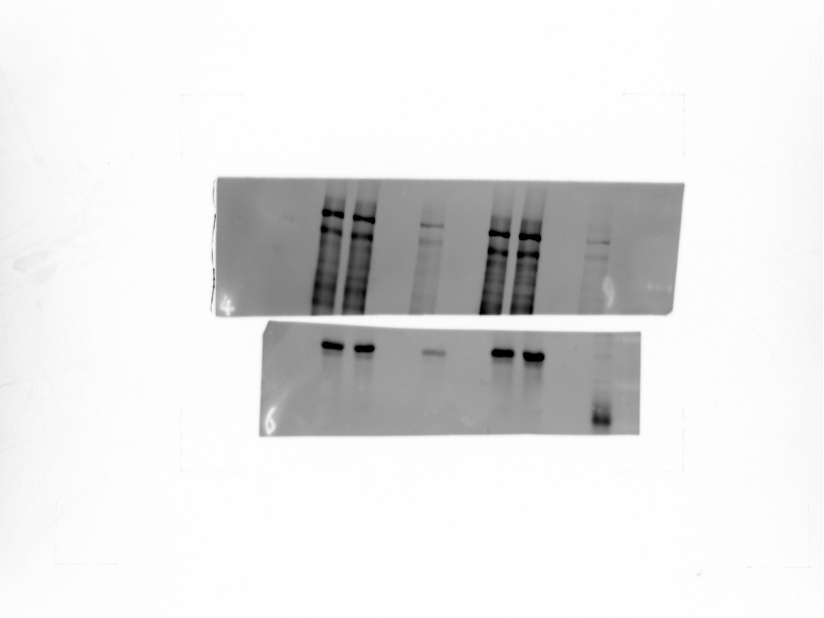

Supplement: Figure 5—source data 1. [file elife-92236-fig5-data1.zip › Figure_5-source_data_1/Figure_5-source_data_1_ Figure_5D_mTOR.jpg]

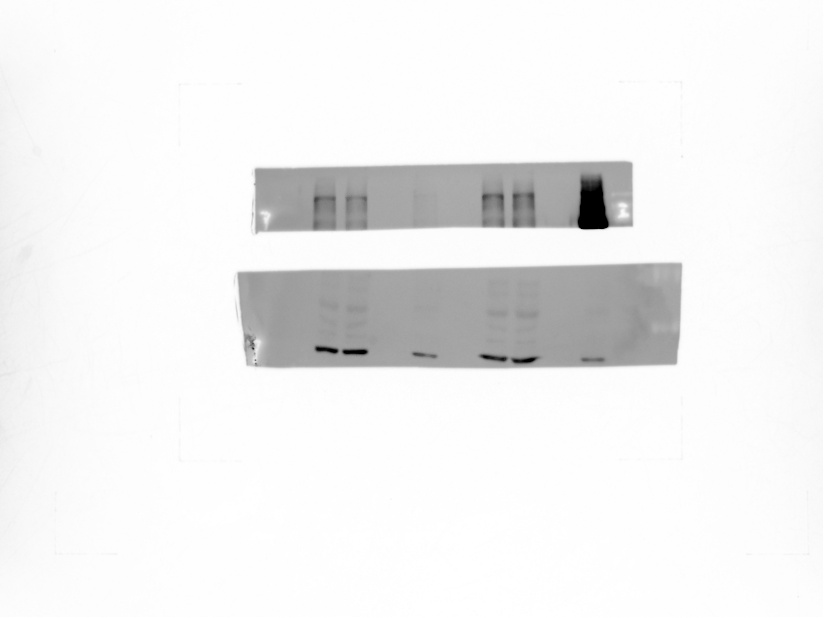

Supplement: Figure 5—source data 1. [file elife-92236-fig5-data1.zip › Figure_5-source_data_1/Figure_5-source_data_1_ Figure_5D_Raptor.jpg]

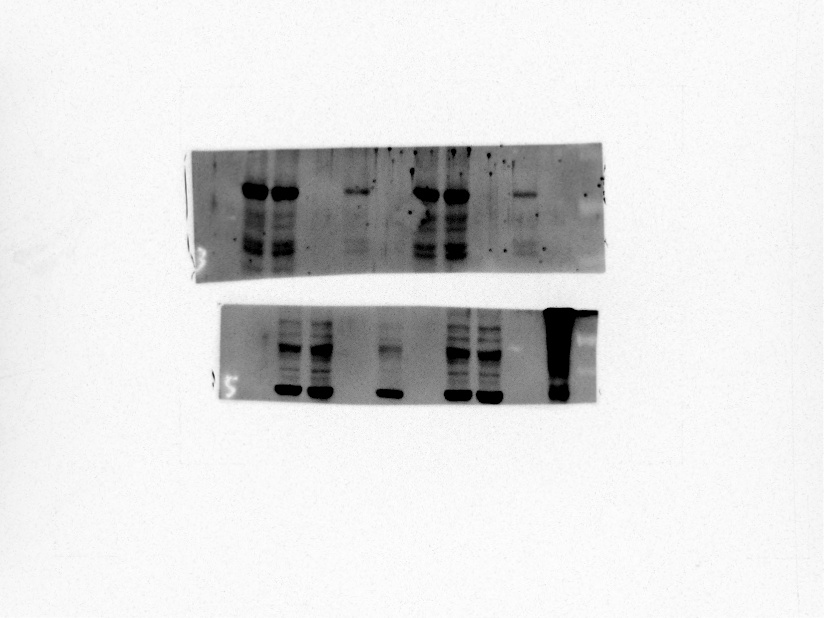

Supplement: Figure 5—source data 1. [file elife-92236-fig5-data1.zip › Figure_5-source_data_1/Figure_5-source_data_1_ Figure_5D_S6K1.jpg]

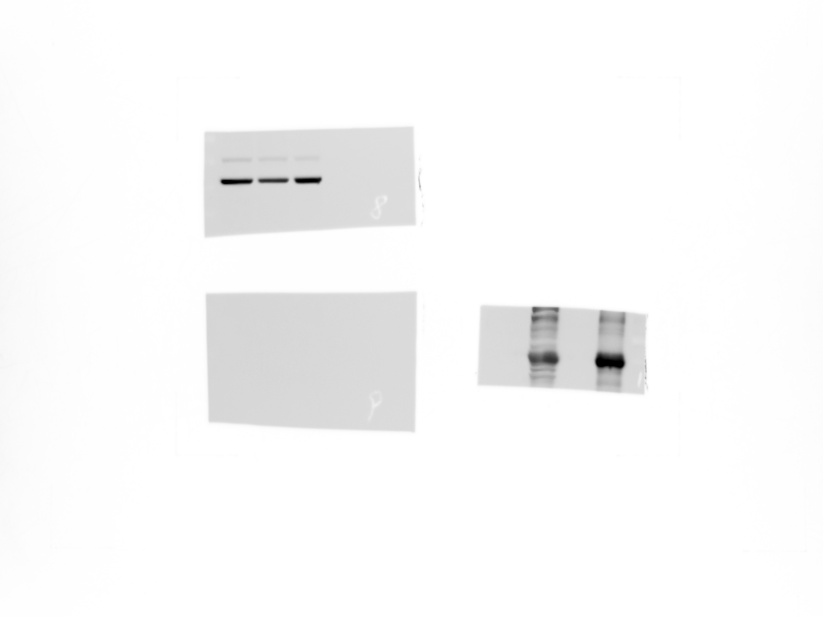

Supplement: Figure 5—source data 1. [file elife-92236-fig5-data1.zip › Figure_5-source_data_1/Figure_5-source_data_1_ Figure_5E_IBTK.jpg]

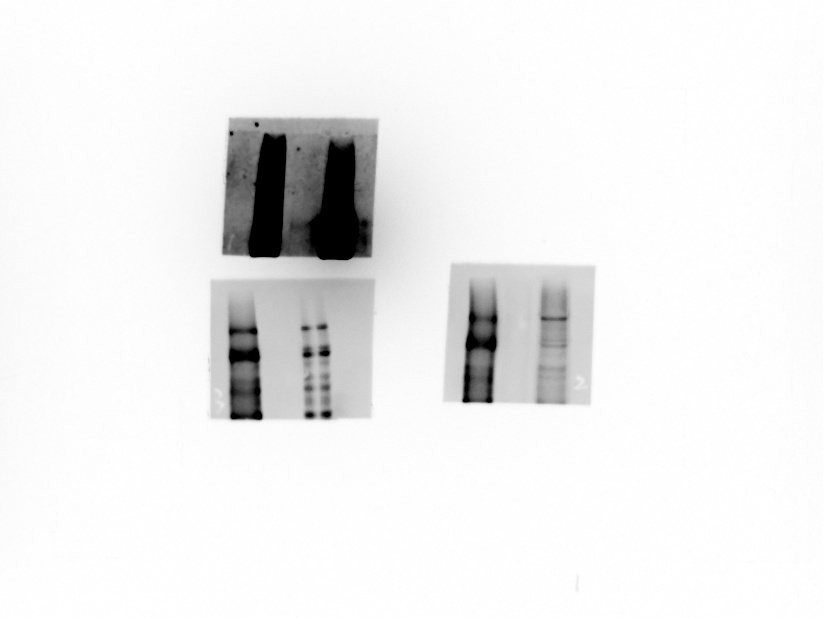

Supplement: Figure 5—source data 1. [file elife-92236-fig5-data1.zip › Figure_5-source_data_1/Figure_5-source_data_1_ Figure_5E_mTOR.jpg]

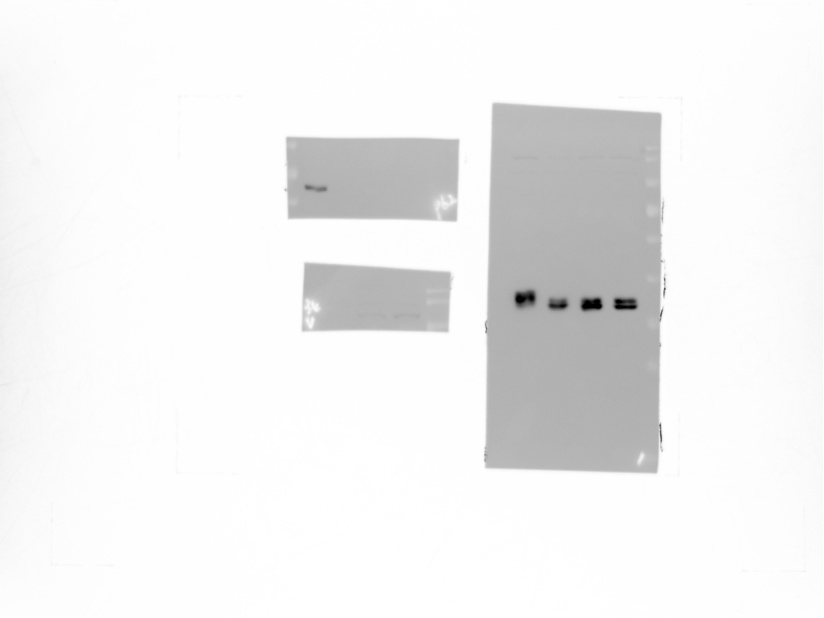

Supplement: Figure 5—source data 1. [file elife-92236-fig5-data1.zip › Figure_5-source_data_1/Figure_5-source_data_1_ Figure_5F_AA- FLAG.jpg]

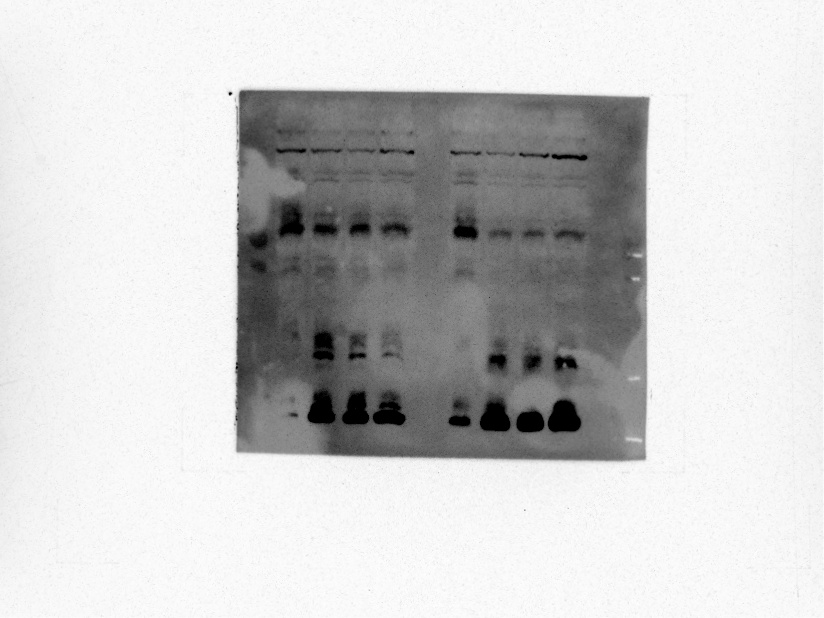

Supplement: Figure 5—source data 1. [file elife-92236-fig5-data1.zip › Figure_5-source_data_1/Figure_5-source_data_1_ Figure_5F_Phos-tag FLAG.jpg]

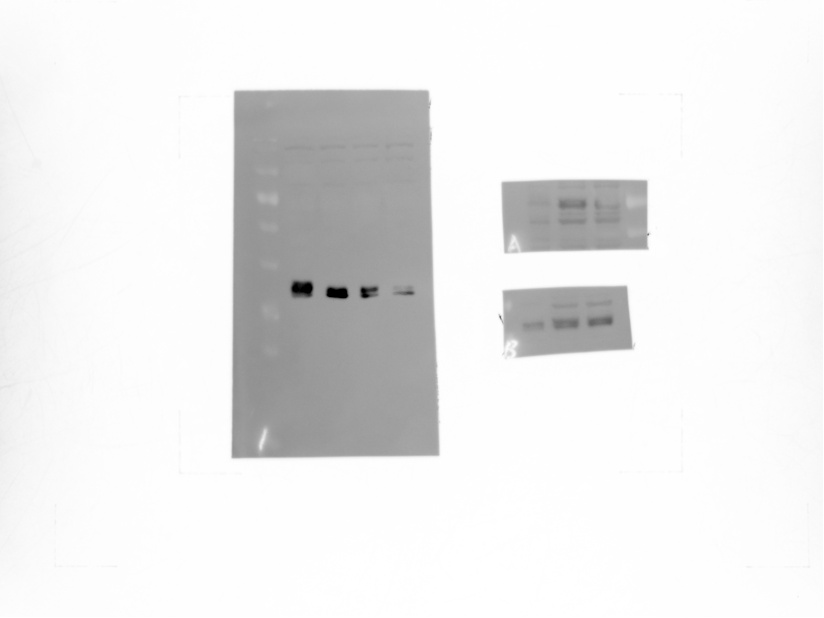

Supplement: Figure 5—source data 1. [file elife-92236-fig5-data1.zip › Figure_5-source_data_1/Figure_5-source_data_1_ Figure_5F_Rapa-FLAG.jpg]

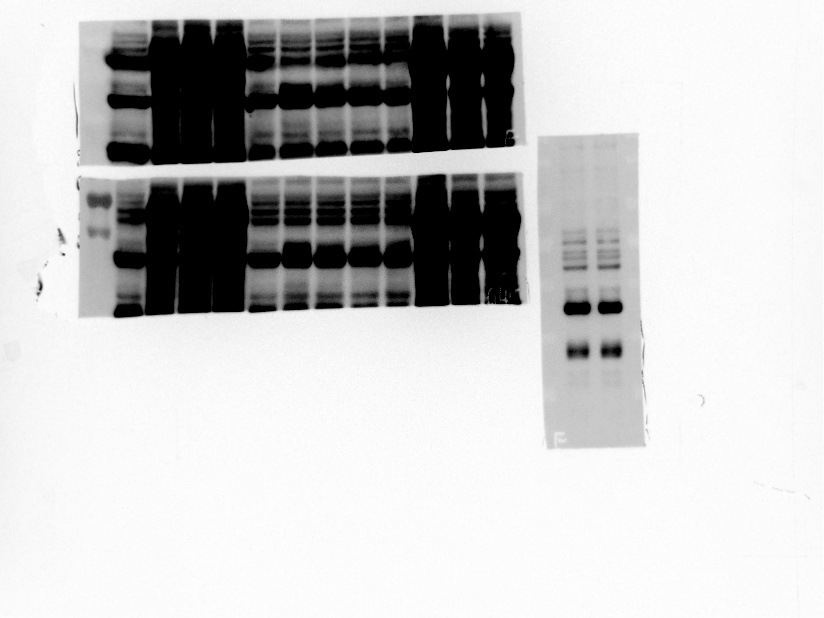

Supplement: Figure 5—source data 1. [file elife-92236-fig5-data1.zip › Figure_5-source_data_1/Figure_5-source_data_1_ Figure_5G_FLAG.jpg]

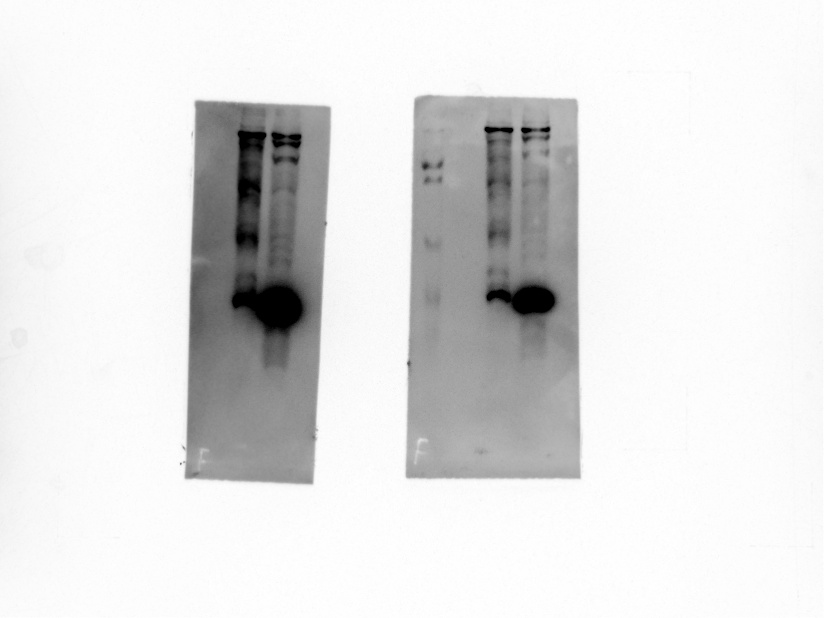

Supplement: Figure 5—source data 1. [file elife-92236-fig5-data1.zip › Figure_5-source_data_1/Figure_5-source_data_1_ Figure_5G_Phos-tag FLAG.jpg]

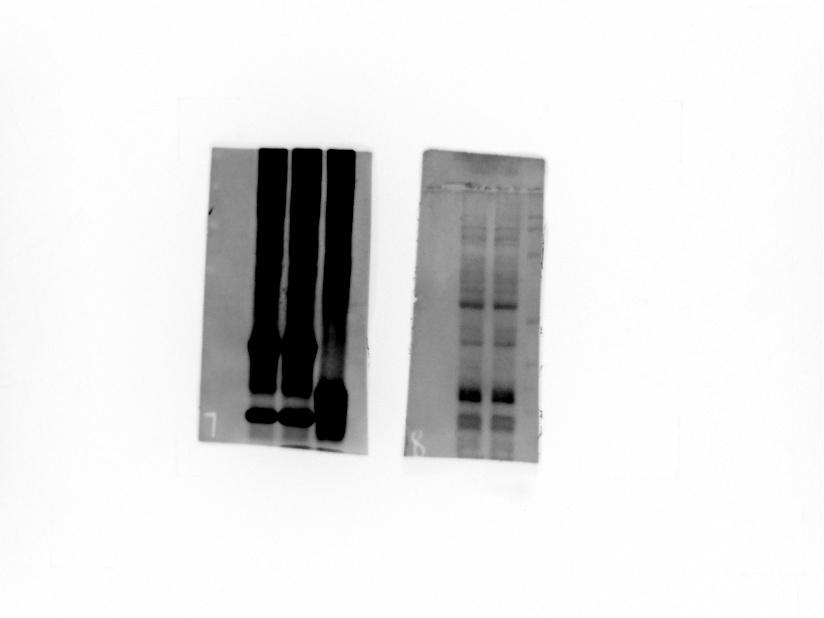

Supplement: Figure 5—source data 1. [file elife-92236-fig5-data1.zip › Figure_5-source_data_1/Figure_5-source_data_1_ Figure_5H_FLAG.jpg]

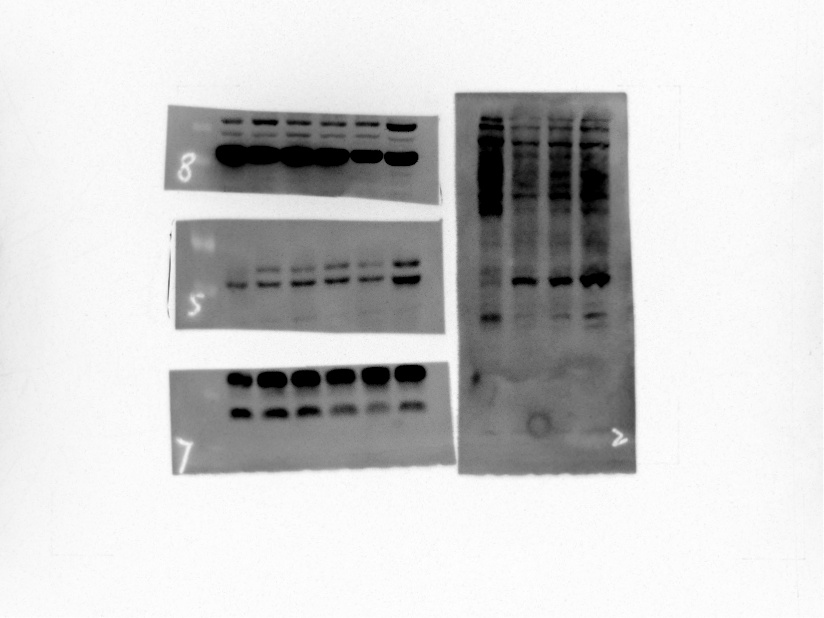

Supplement: Figure 5—source data 1. [file elife-92236-fig5-data1.zip › Figure_5-source_data_1/Figure_5-source_data_1_ Figure_5H_Phos-tag FLAG.jpg]

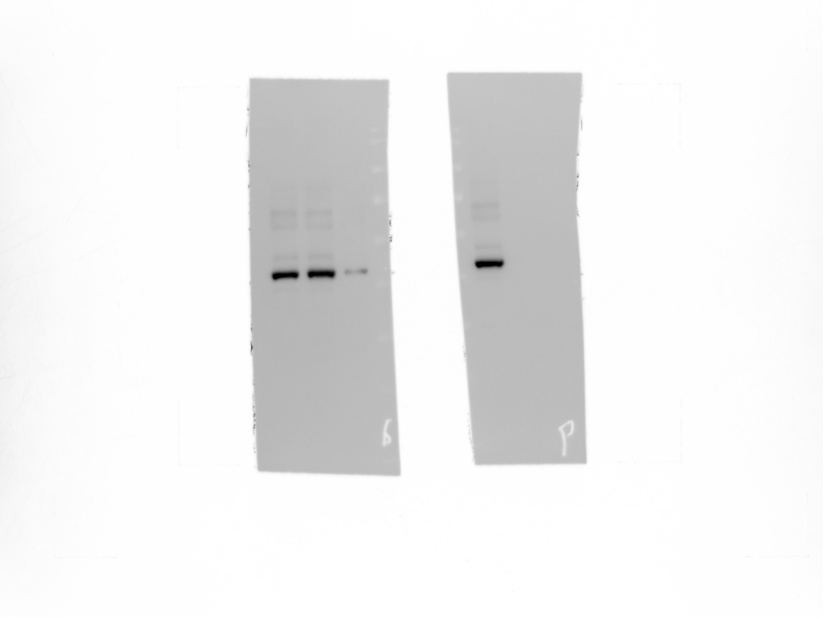

Supplement: Figure 5—source data 1. [file elife-92236-fig5-data1.zip › Figure_5-source_data_1/Figure_5-source_data_1_ Figure_5H_p-S6K1.jpg]

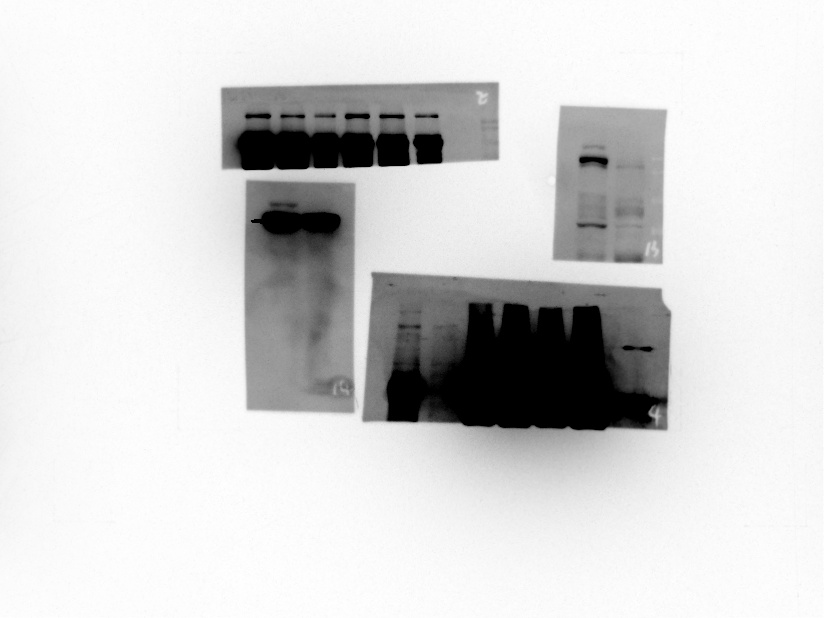

Supplement: Figure 5—source data 1. [file elife-92236-fig5-data1.zip › Figure_5-source_data_1/Figure_5-source_data_1_ Figure_5H_Raptor.jpg]

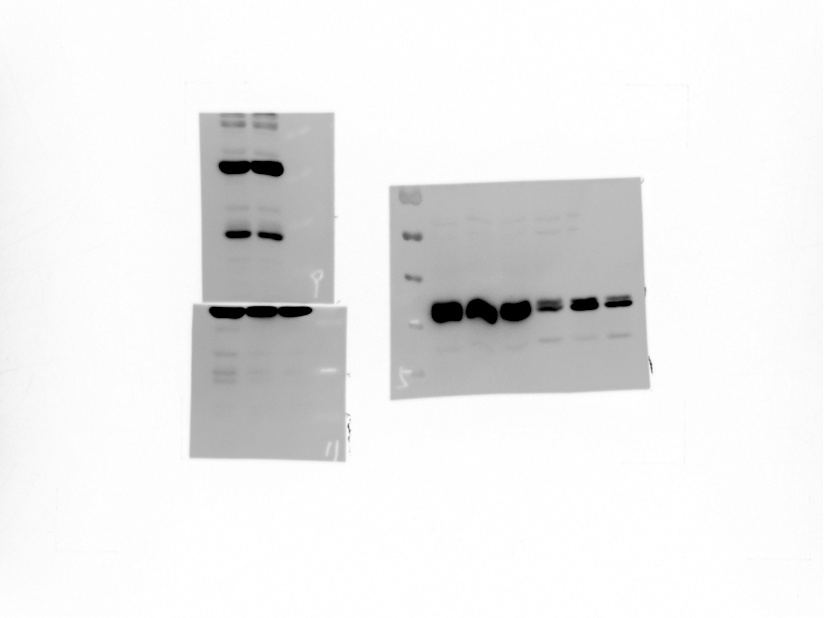

Supplement: Figure 5—source data 1. [file elife-92236-fig5-data1.zip › Figure_5-source_data_1/Figure_5-source_data_1_ Figure_5K_FLAG.jpg]

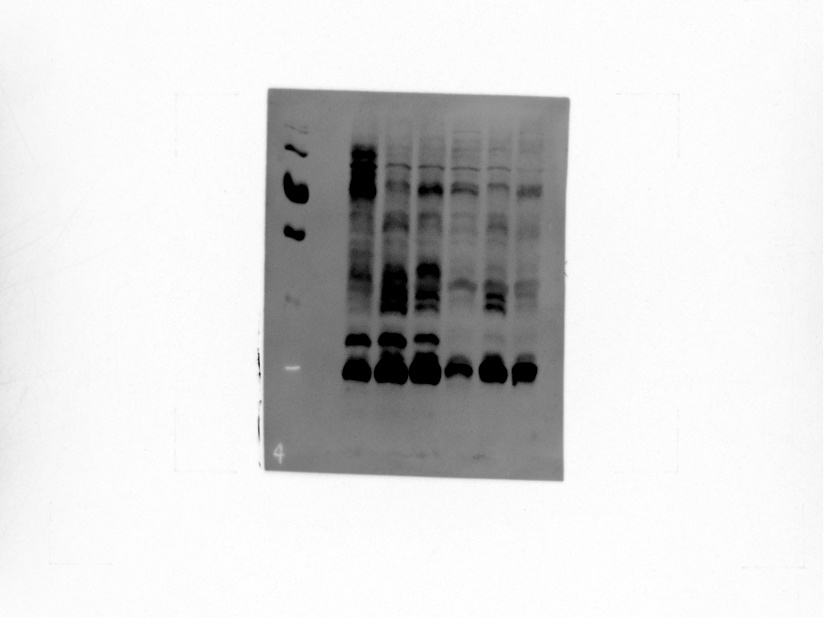

Supplement: Figure 5—source data 1. [file elife-92236-fig5-data1.zip › Figure_5-source_data_1/Figure_5-source_data_1_ Figure_5K_Phos-tag FLAG.jpg]

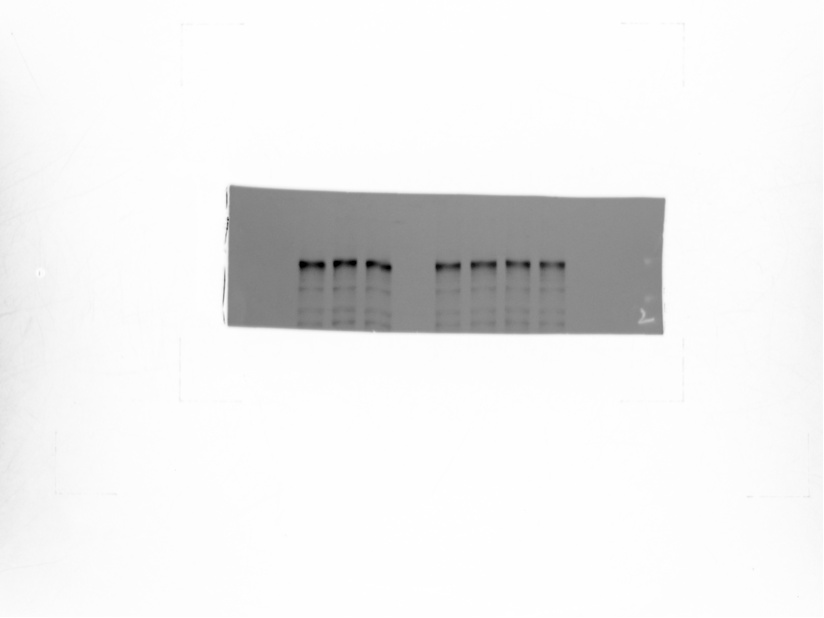

Supplement: Figure 5—source data 1. [file elife-92236-fig5-data1.zip › Figure_5-source_data_1/Figure_5-source_data_1_ Figure_5L_IP IBTK.jpg]

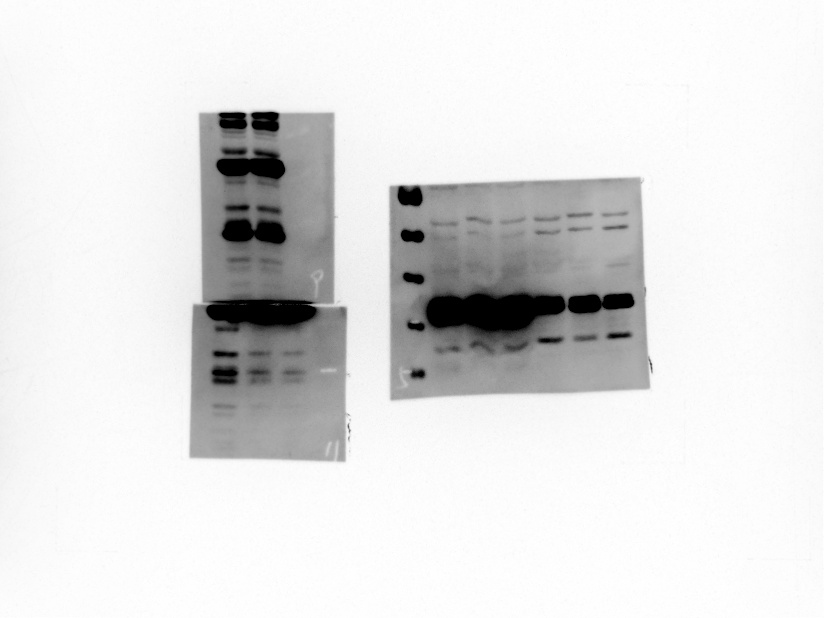

Supplement: Figure 5—source data 1. [file elife-92236-fig5-data1.zip › Figure_5-source_data_1/Figure_5-source_data_1_ Figure_5L_IP p-IBTK.jpg]

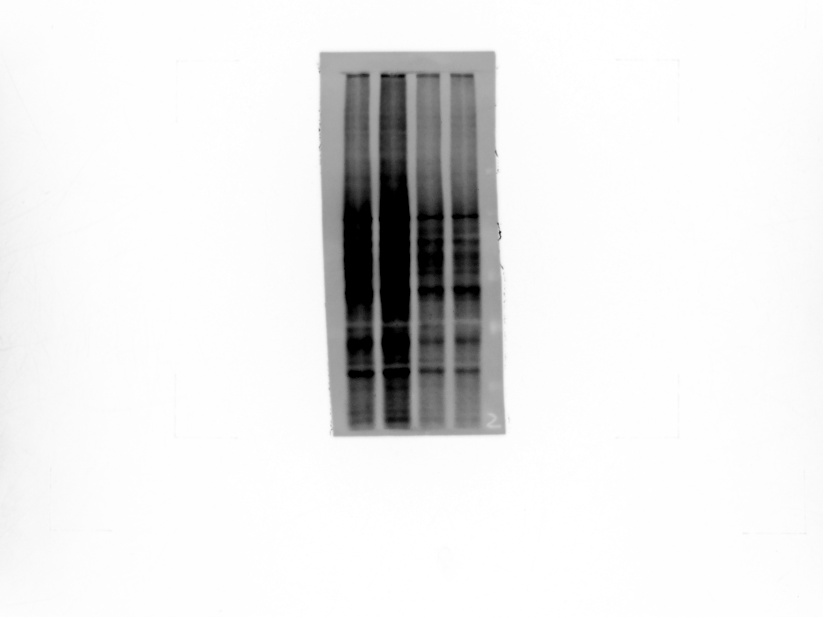

Supplement: Figure 5—source data 1. [file elife-92236-fig5-data1.zip › Figure_5-source_data_1/Figure_5-source_data_1_ Figure_5M_IP HA.jpg]

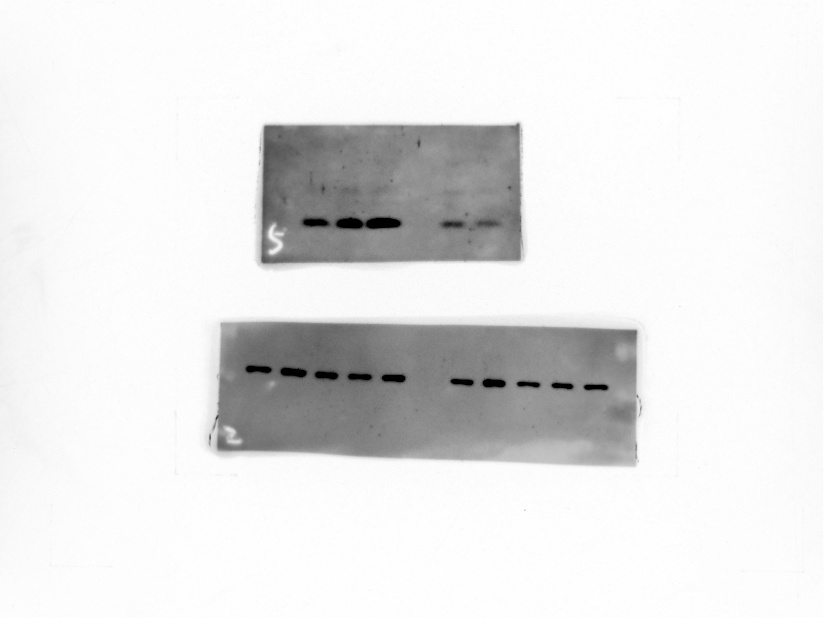

Supplement: Figure 5—source data 1. [file elife-92236-fig5-data1.zip › Figure_5-source_data_1/Figure_5-source_data_1_ Figure_5M_IP PA.jpg]

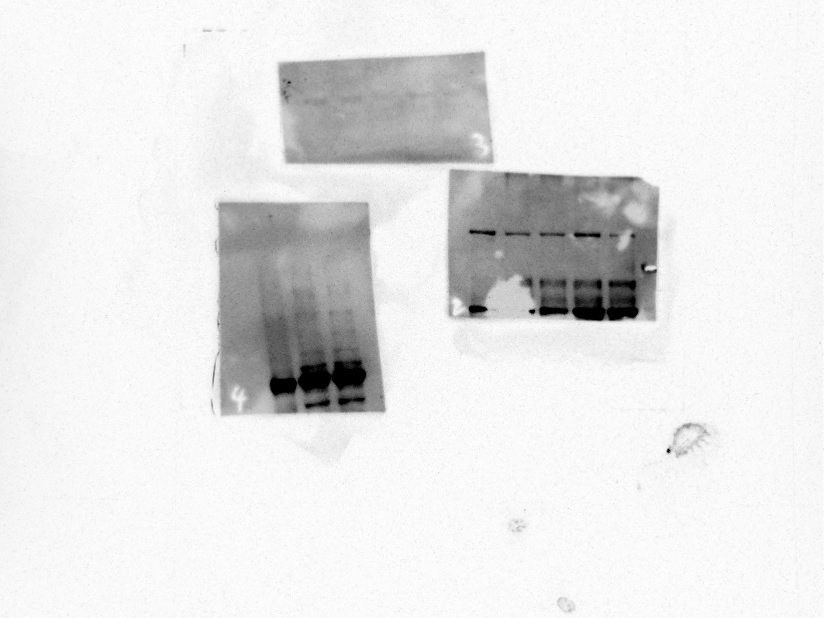

Supplement: Figure 5—source data 1. [file elife-92236-fig5-data1.zip › Figure_5-source_data_1/Figure_5-source_data_1_ Figure_5M_WCL FLAG.jpg]

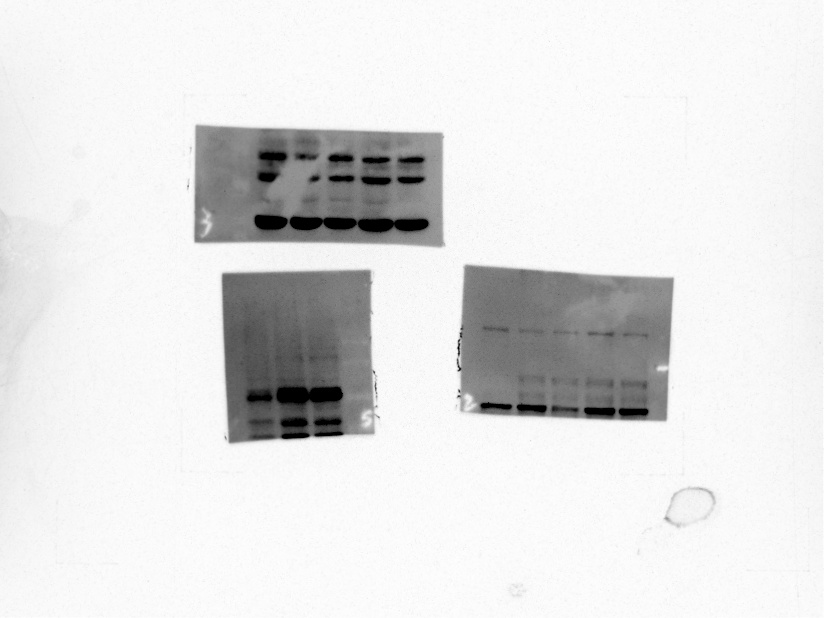

Supplement: Figure 5—source data 1. [file elife-92236-fig5-data1.zip › Figure_5-source_data_1/Figure_5-source_data_1_ Figure_5M_WCL PA.jpg]

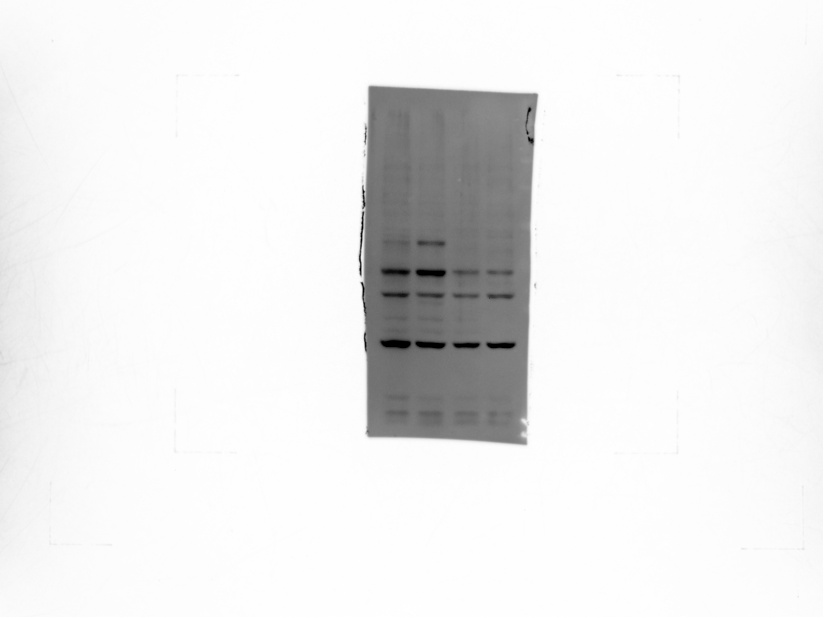

Supplement: Figure 5—source data 1. [file elife-92236-fig5-data1.zip › Figure_5-source_data_1/Figure_5-source_data_1_ Figure_5M_WCL p-S6K1.jpg]

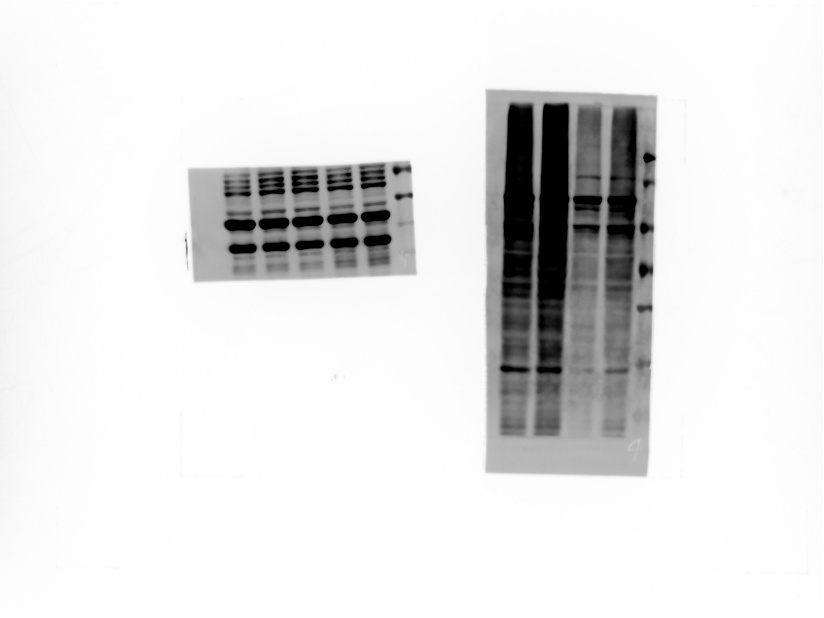

Supplement: Figure 5—source data 1. [file elife-92236-fig5-data1.zip › Figure_5-source_data_1/Figure_5-source_data_1_ Figure_5N_IP HA.jpg]

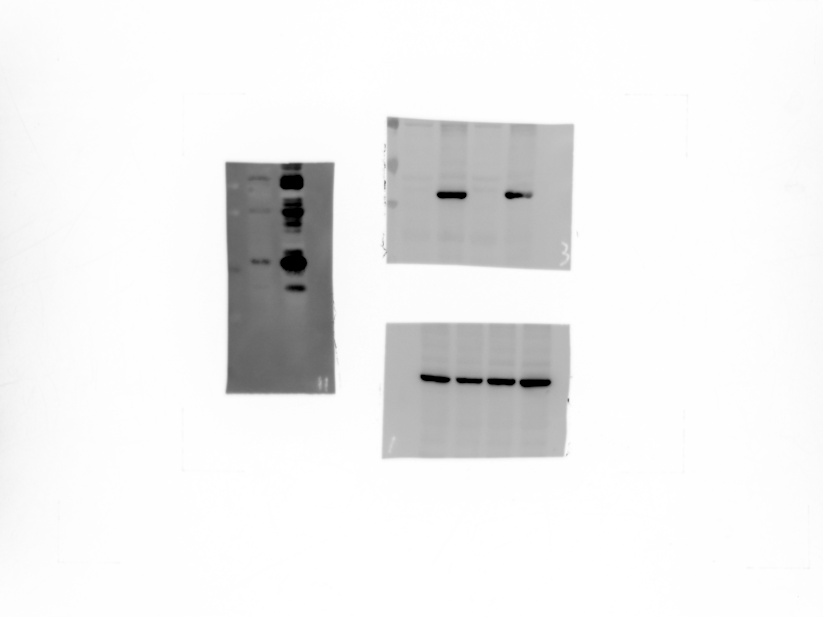

Supplement: Figure 5—source data 1. [file elife-92236-fig5-data1.zip › Figure_5-source_data_1/Figure_5-source_data_1_ Figure_5N_IP PA.jpg]

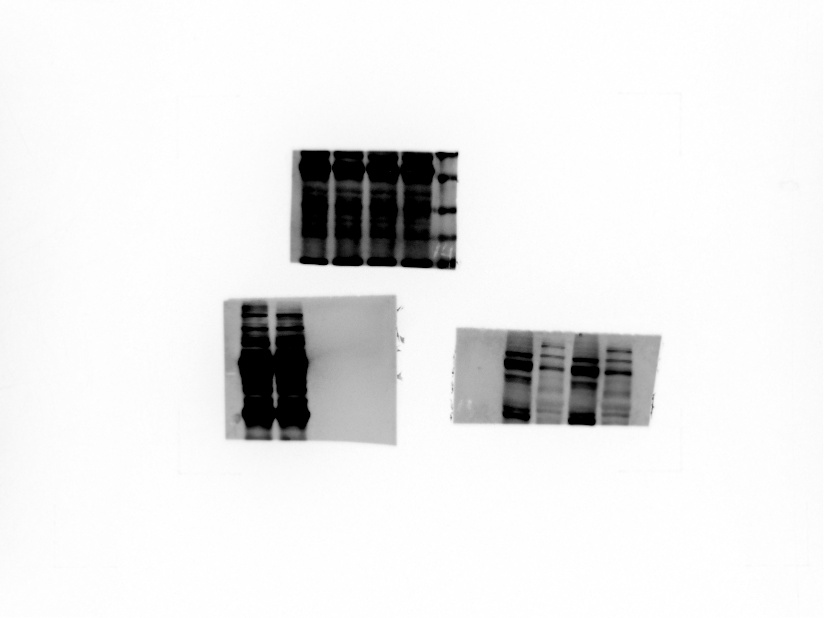

Supplement: Figure 5—source data 1. [file elife-92236-fig5-data1.zip › Figure_5-source_data_1/Figure_5-source_data_1_ Figure_5N_WCL FLAG.jpg]

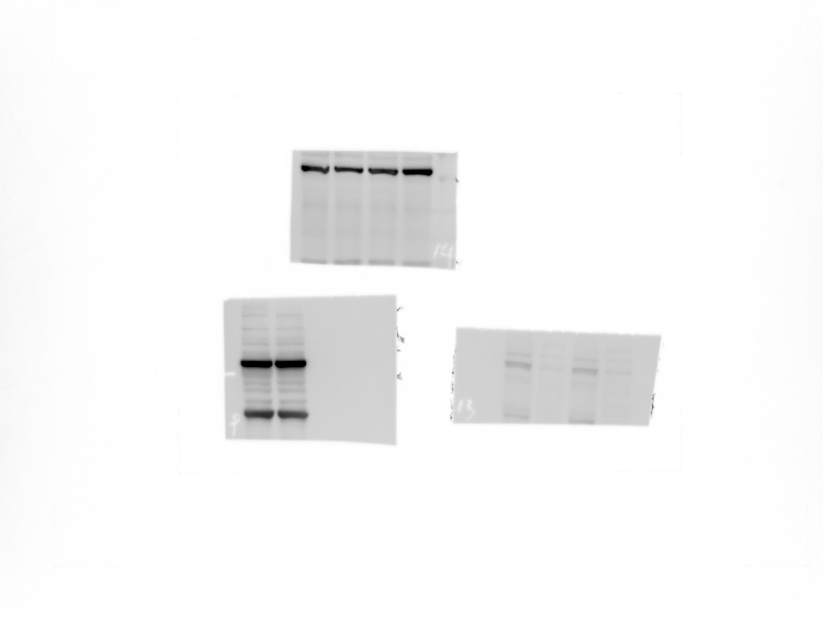

Supplement: Figure 5—source data 1. [file elife-92236-fig5-data1.zip › Figure_5-source_data_1/Figure_5-source_data_1_ Figure_5N_WCL PA.jpg]

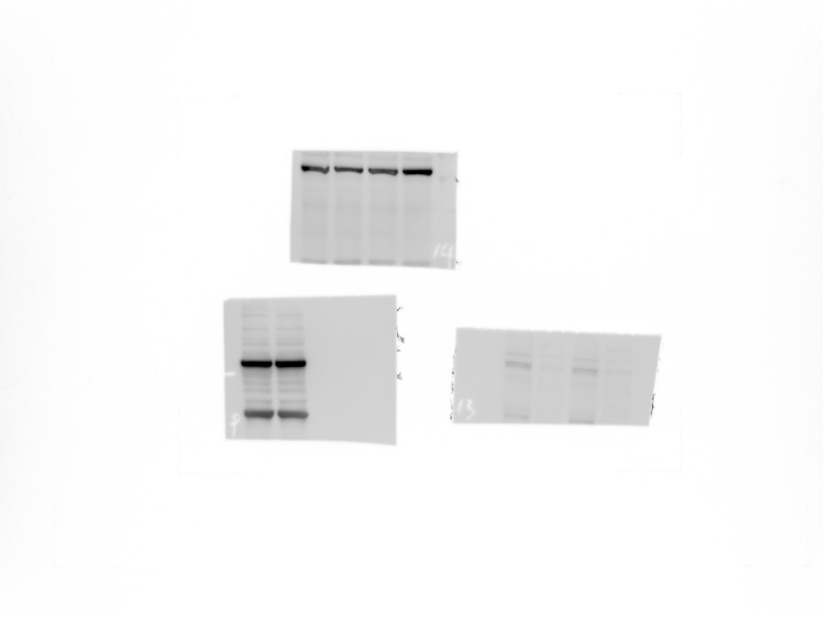

Supplement: Figure 5—source data 1. [file elife-92236-fig5-data1.zip › Figure_5-source_data_1/Figure_5-source_data_1_ Figure_5N_WCL p-S6K1.jpg]

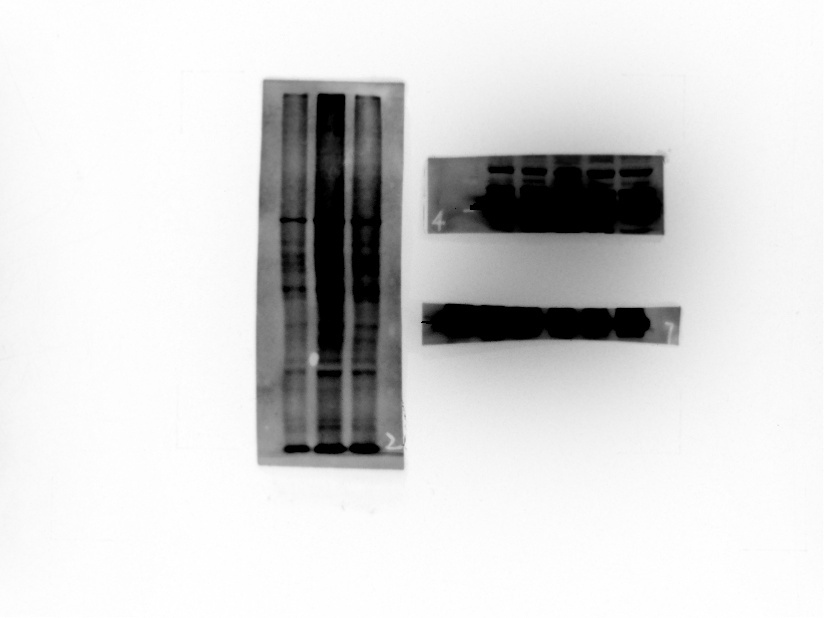

Supplement: Figure 5—source data 1. [file elife-92236-fig5-data1.zip › Figure_5-source_data_1/Figure_5-source_data_1_ Figure_5O_IP HA.jpg]

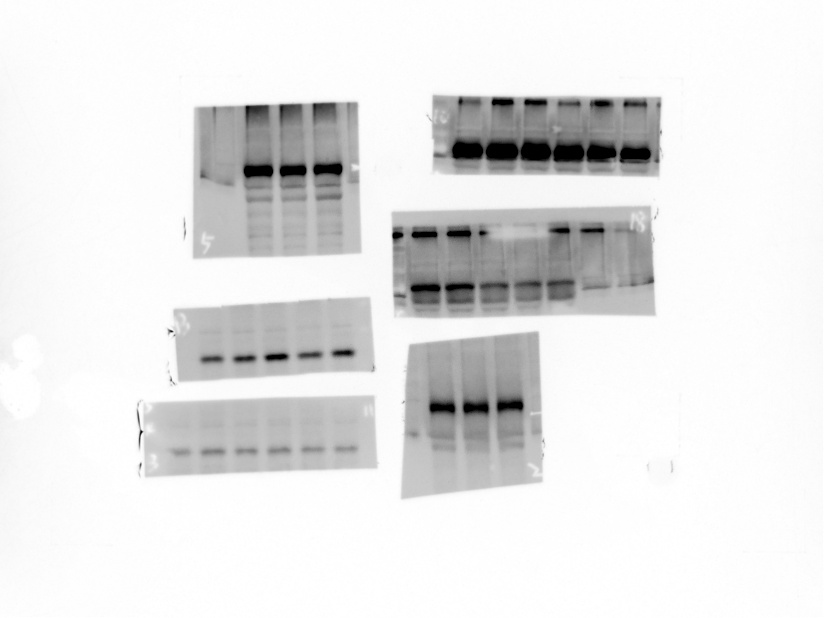

Supplement: Figure 5—source data 1. [file elife-92236-fig5-data1.zip › Figure_5-source_data_1/Figure_5-source_data_1_ Figure_5O_IP PA.jpg]

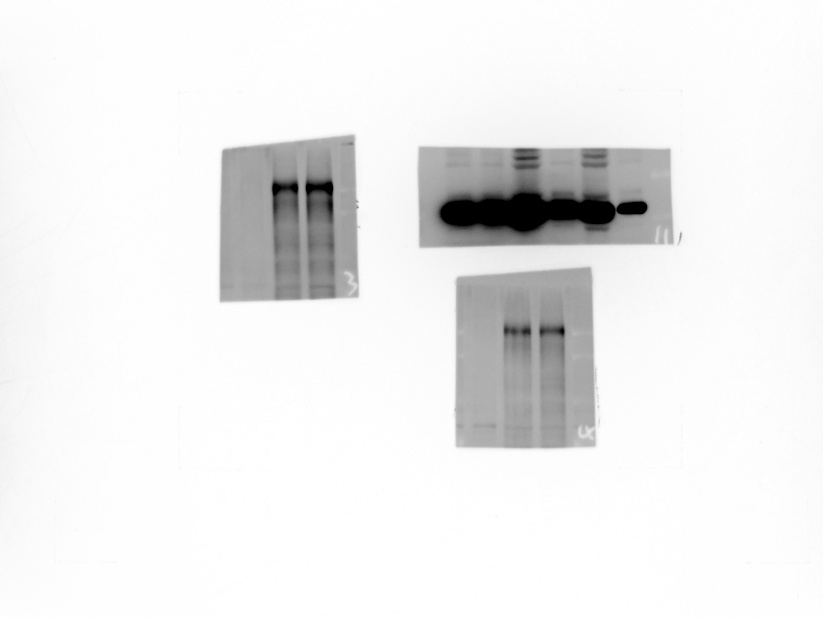

Supplement: Figure 5—source data 1. [file elife-92236-fig5-data1.zip › Figure_5-source_data_1/Figure_5-source_data_1_ Figure_5O_WCL FLAG.jpg]

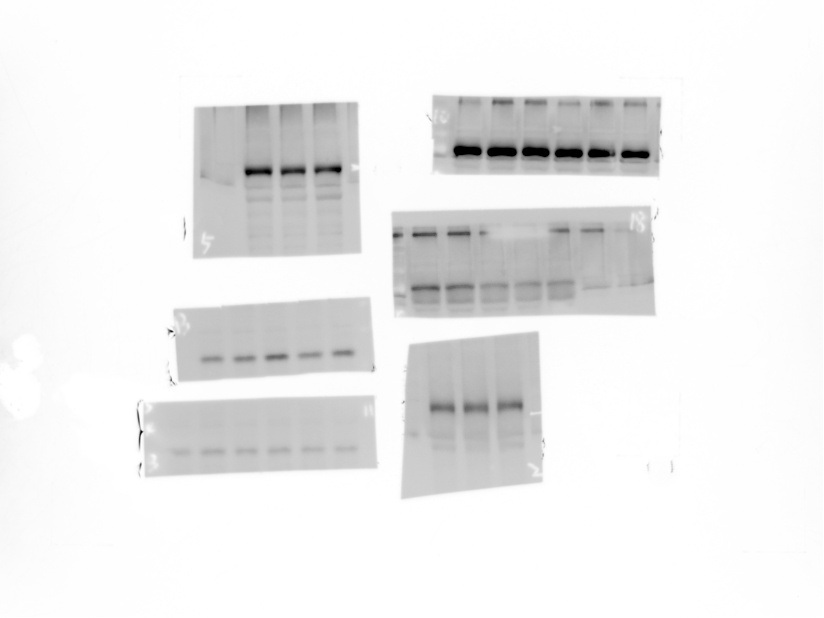

Supplement: Figure 5—source data 1. [file elife-92236-fig5-data1.zip › Figure_5-source_data_1/Figure_5-source_data_1_ Figure_5O_WCL PA.jpg]

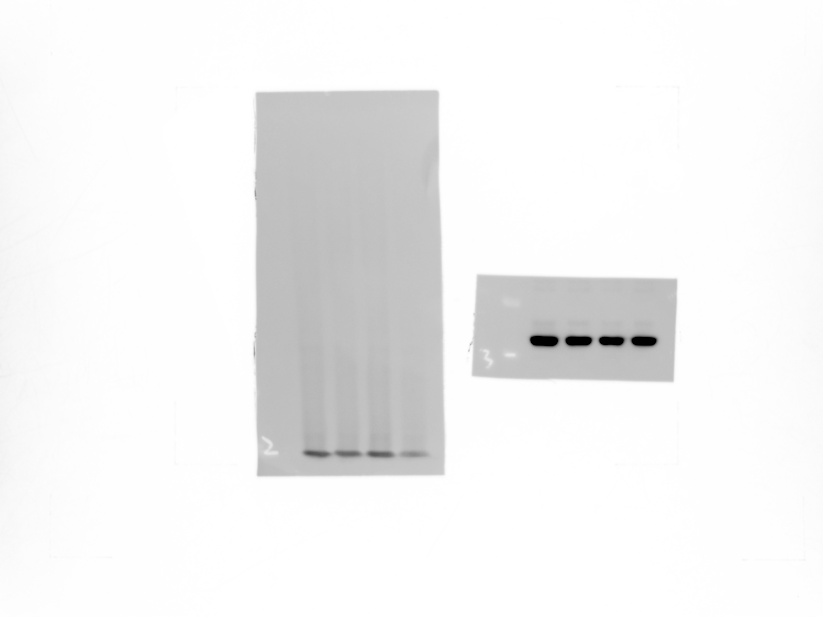

Supplement: Figure 6—source data 1. [file elife-92236-fig6-data1.zip › Figure_6-source_data_1/Figure_6-source_data_1_ Figure_6A_Actin.jpg]

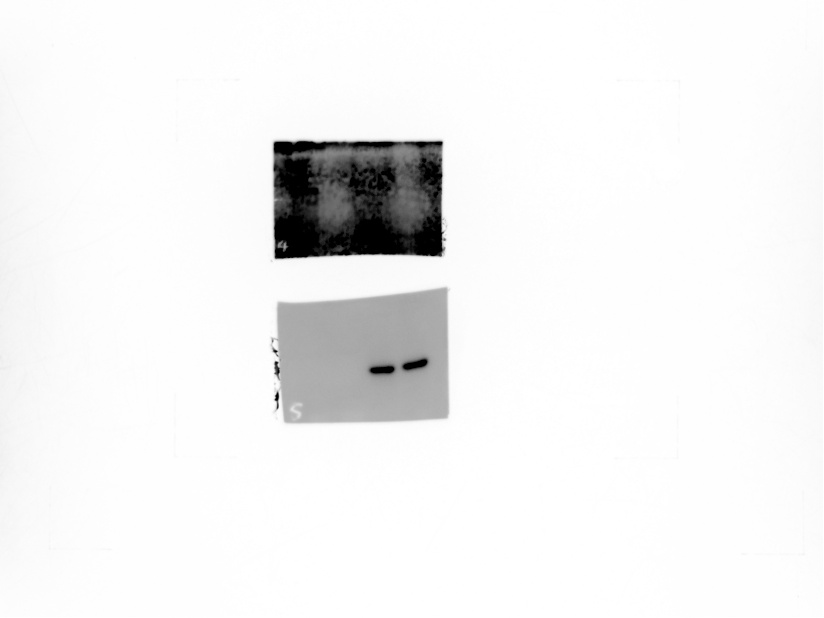

Supplement: Figure 6—source data 1. [file elife-92236-fig6-data1.zip › Figure_6-source_data_1/Figure_6-source_data_1_ Figure_6A_FLAG.jpg]

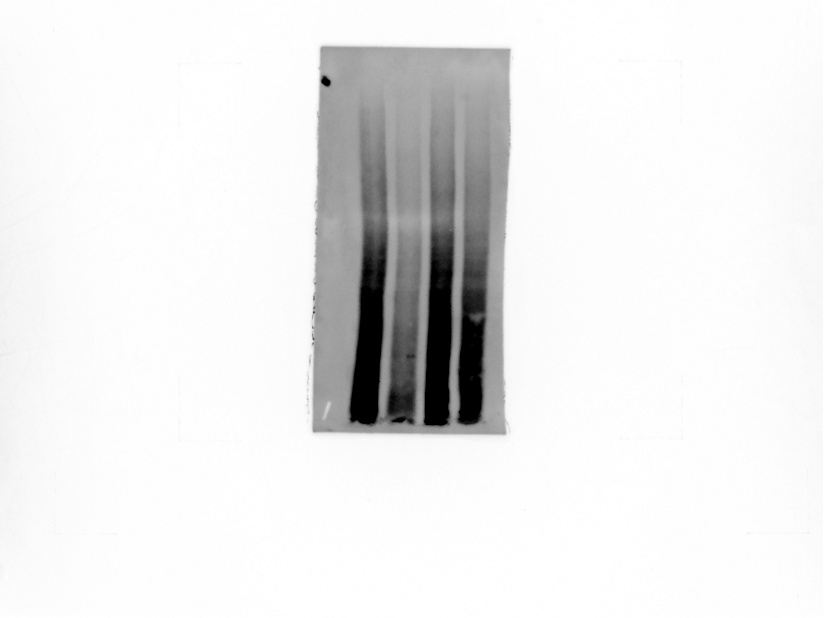

Supplement: Figure 6—source data 1. [file elife-92236-fig6-data1.zip › Figure_6-source_data_1/Figure_6-source_data_1_ Figure_6A_puromycin.jpg]

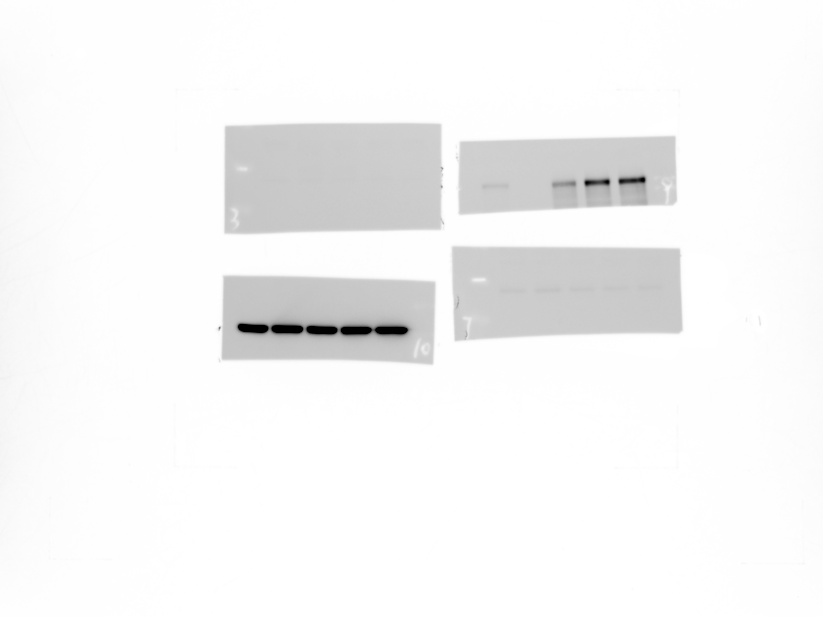

Supplement: Figure 6—source data 1. [file elife-92236-fig6-data1.zip › Figure_6-source_data_1/Figure_6-source_data_1_ Figure_6B_Actin.jpg]

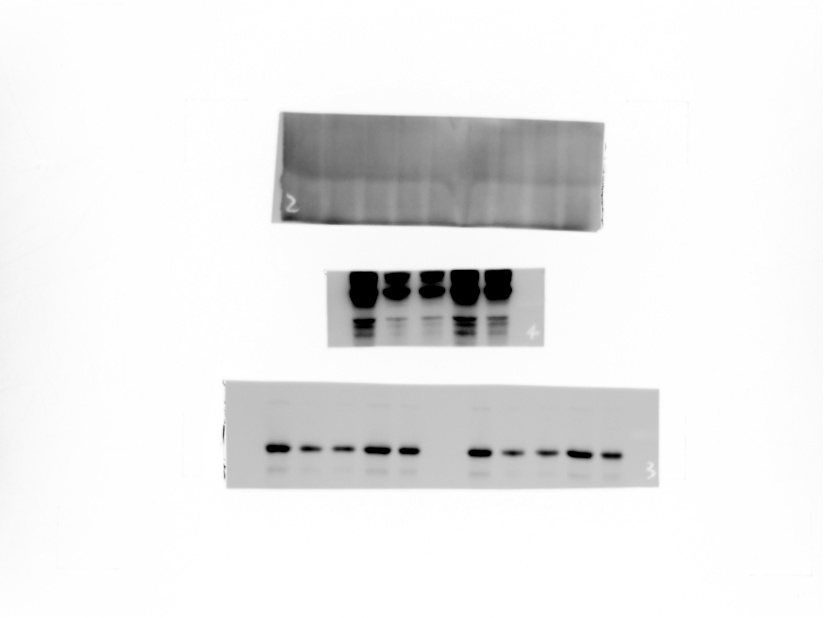

Supplement: Figure 6—source data 1. [file elife-92236-fig6-data1.zip › Figure_6-source_data_1/Figure_6-source_data_1_ Figure_6B_CDK6.jpg]

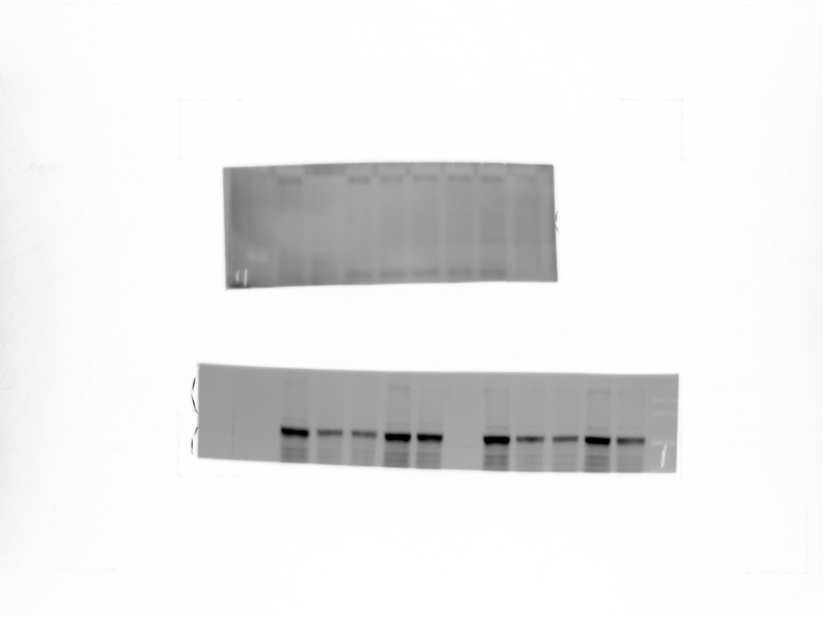

Supplement: Figure 6—source data 1. [file elife-92236-fig6-data1.zip › Figure_6-source_data_1/Figure_6-source_data_1_ Figure_6B_EZH2.jpg]

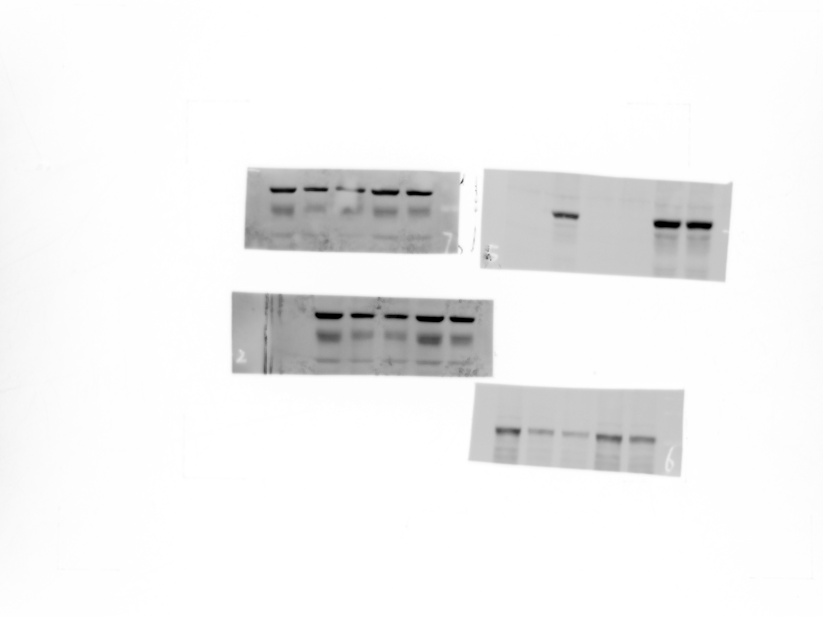

Supplement: Figure 6—source data 1. [file elife-92236-fig6-data1.zip › Figure_6-source_data_1/Figure_6-source_data_1_ Figure_6B_IBTK.jpg]

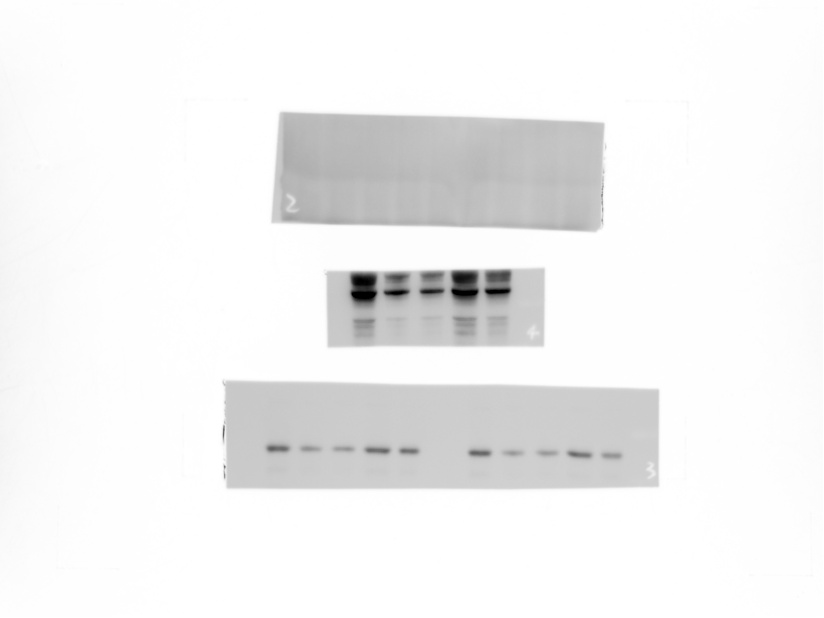

Supplement: Figure 6—source data 1. [file elife-92236-fig6-data1.zip › Figure_6-source_data_1/Figure_6-source_data_1_ Figure_6B_MYC.jpg]

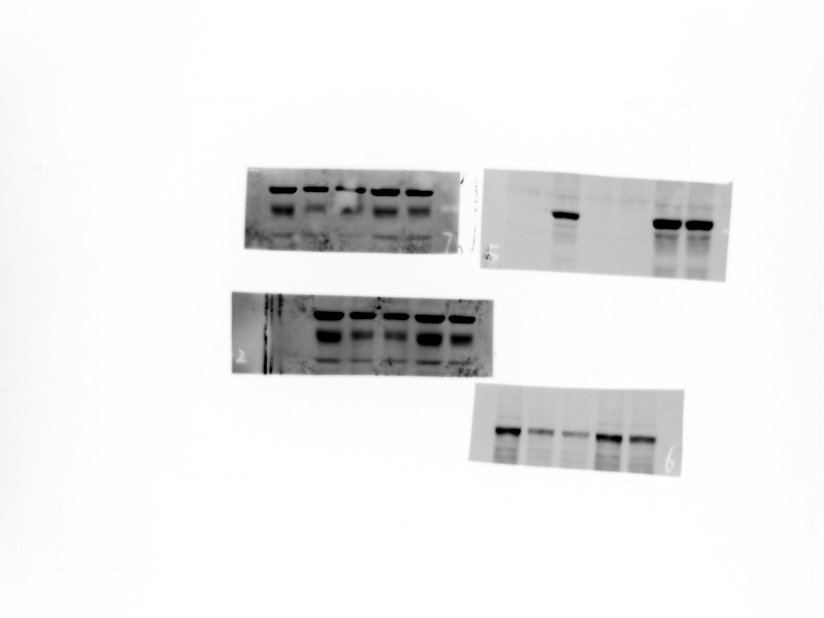

Supplement: Figure 6—source data 1. [file elife-92236-fig6-data1.zip › Figure_6-source_data_1/Figure_6-source_data_1_ Figure_6B_XIAP.jpg]

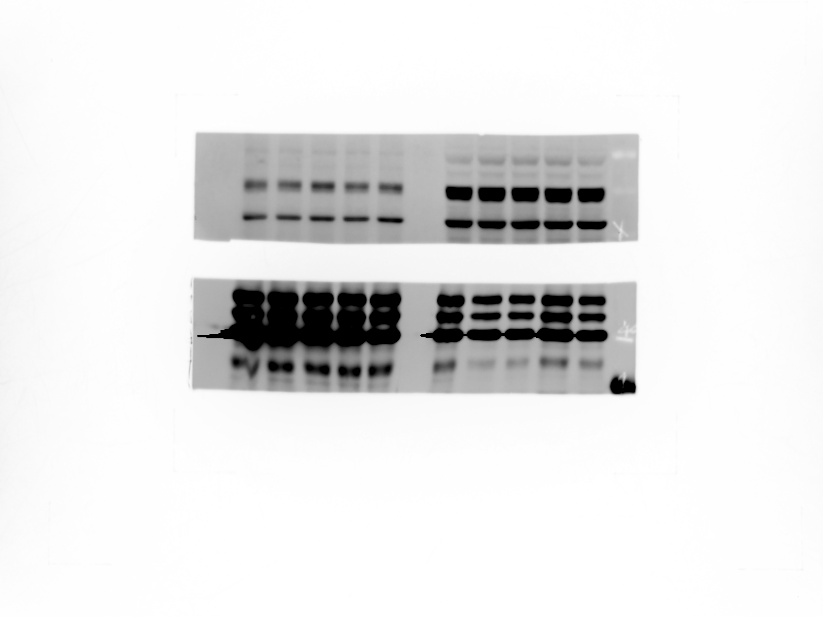

Supplement: Figure 6—source data 1. [file elife-92236-fig6-data1.zip › Figure_6-source_data_1/Figure_6-source_data_1_ Figure_6C_EIF4A1.jpg]

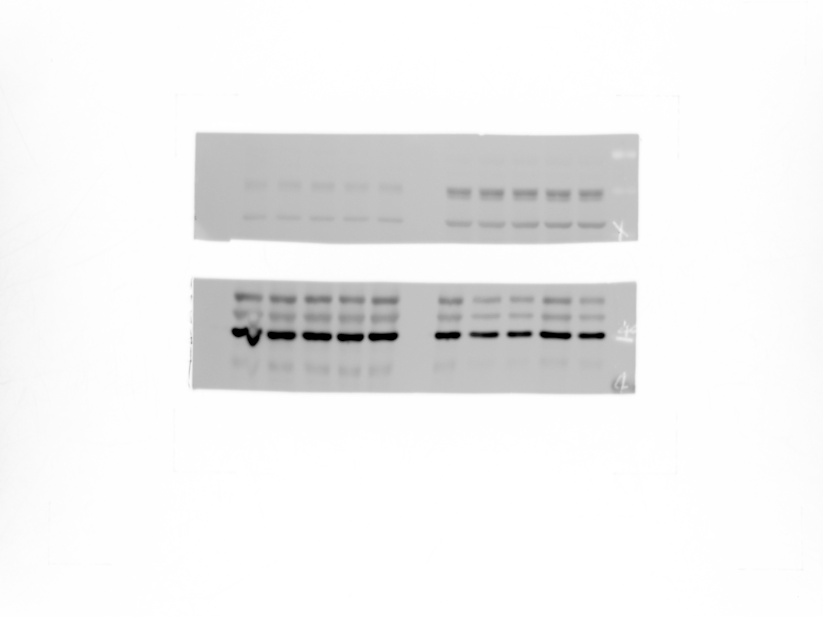

Supplement: Figure 6—source data 1. [file elife-92236-fig6-data1.zip › Figure_6-source_data_1/Figure_6-source_data_1_ Figure_6C_EIF4B.jpg]

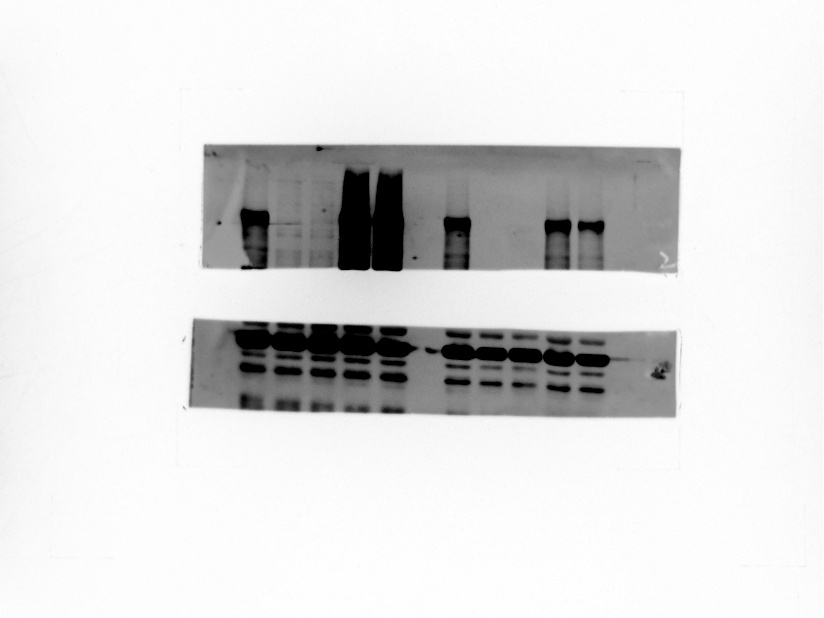

Supplement: Figure 6—source data 1. [file elife-92236-fig6-data1.zip › Figure_6-source_data_1/Figure_6-source_data_1_ Figure_6C_EIF4E.jpg]

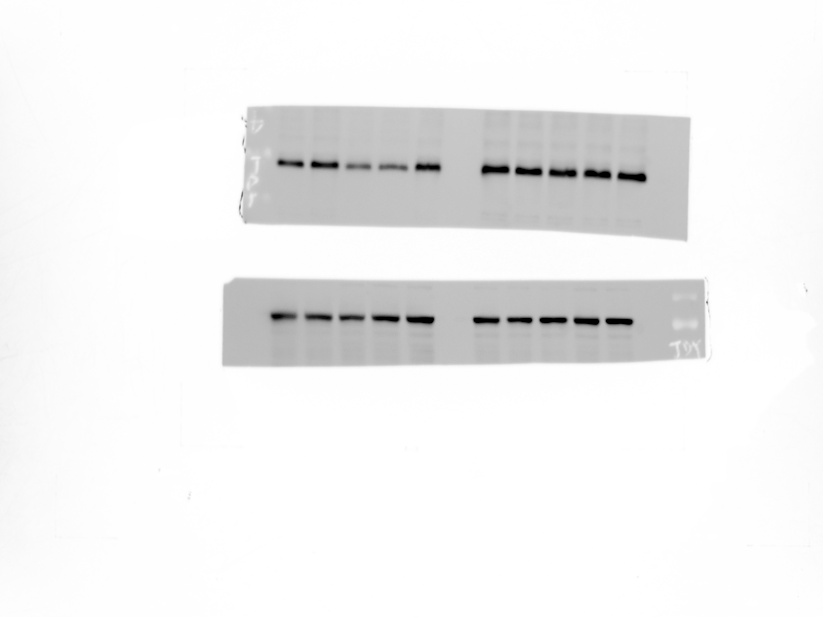

Supplement: Figure 6—source data 1. [file elife-92236-fig6-data1.zip › Figure_6-source_data_1/Figure_6-source_data_1_ Figure_6C_EIF4G.jpg]

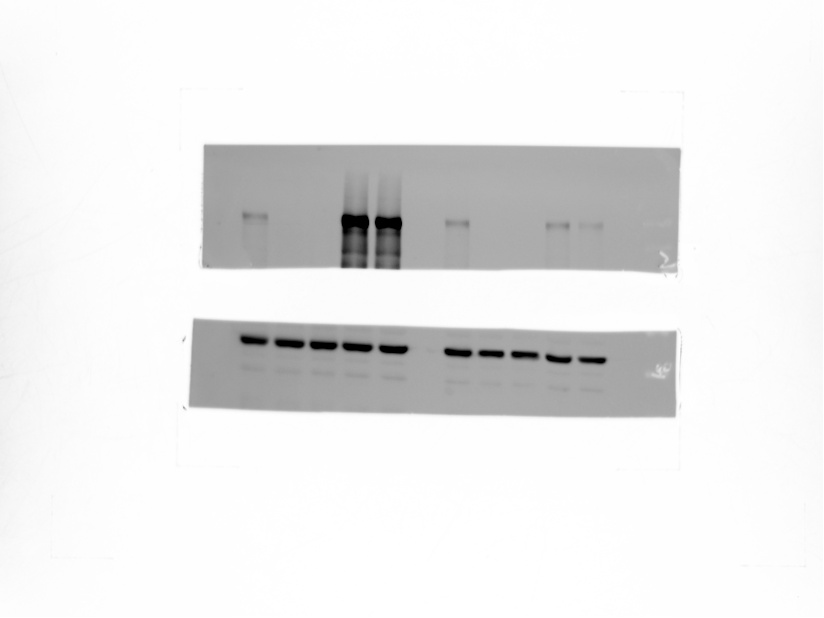

Supplement: Figure 6—source data 1. [file elife-92236-fig6-data1.zip › Figure_6-source_data_1/Figure_6-source_data_1_ Figure_6C_IBTK.jpg]

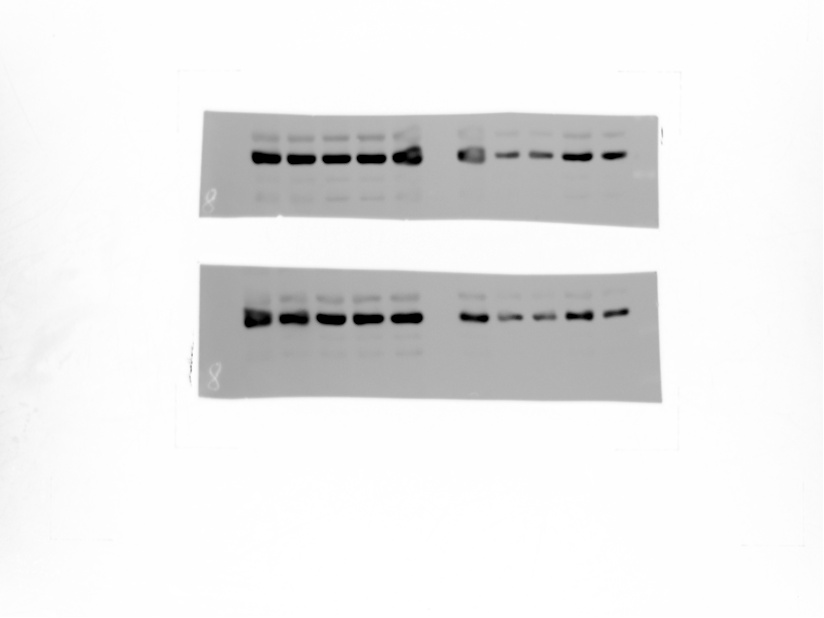

Supplement: Figure 6—source data 1. [file elife-92236-fig6-data1.zip › Figure_6-source_data_1/Figure_6-source_data_1_ Figure_6D_eIF4A1.jpg]

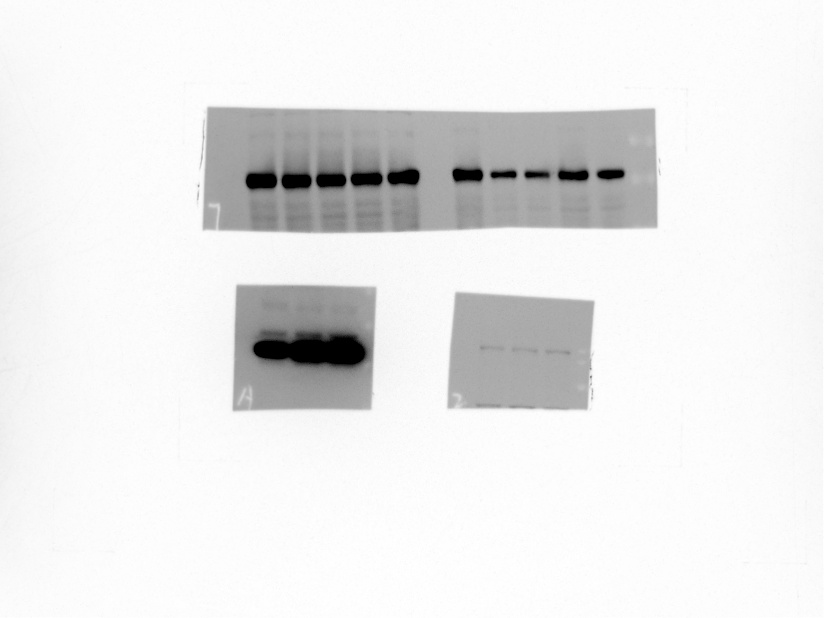

Supplement: Figure 6—source data 1. [file elife-92236-fig6-data1.zip › Figure_6-source_data_1/Figure_6-source_data_1_ Figure_6D_eIF4B.jpg]

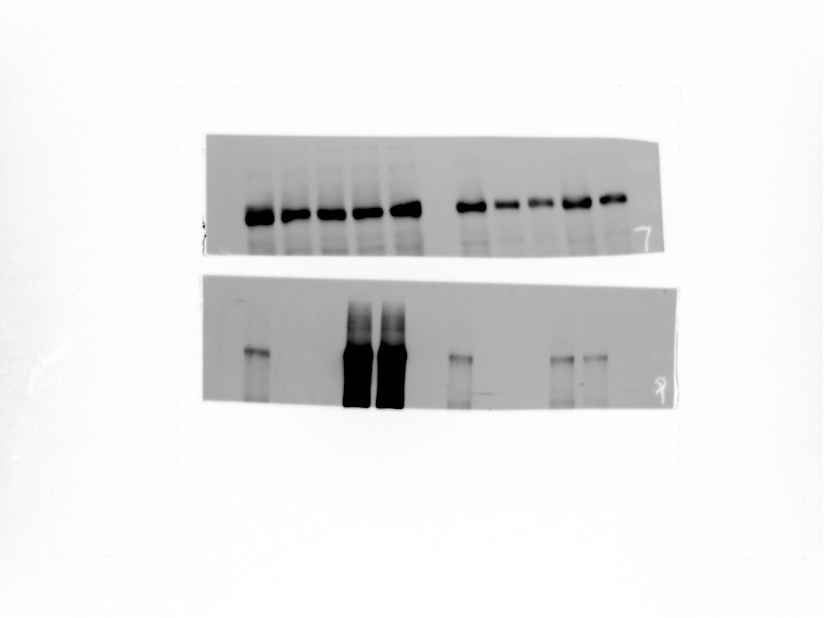

Supplement: Figure 6—source data 1. [file elife-92236-fig6-data1.zip › Figure_6-source_data_1/Figure_6-source_data_1_ Figure_6D_eIF4G.jpg]

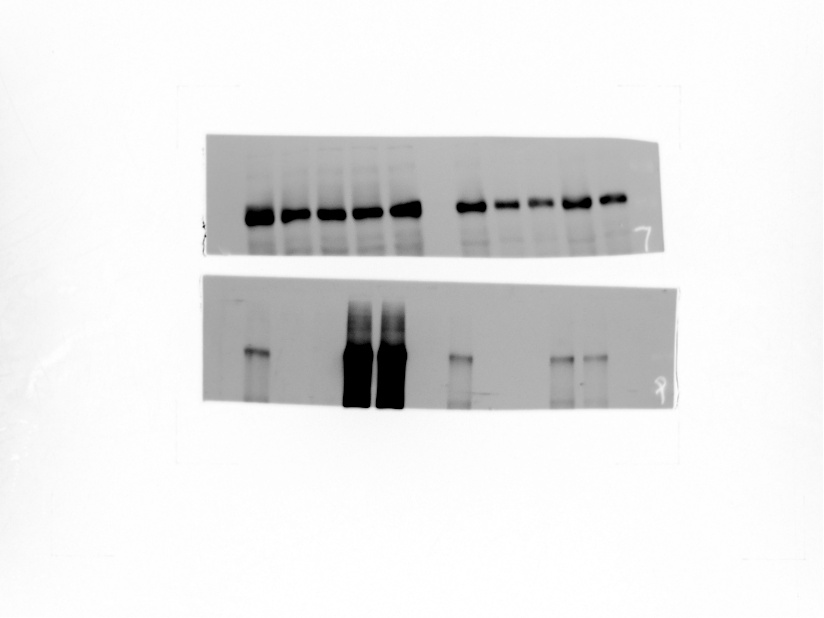

Supplement: Figure 6—source data 1. [file elife-92236-fig6-data1.zip › Figure_6-source_data_1/Figure_6-source_data_1_ Figure_6D_IBTK.jpg]

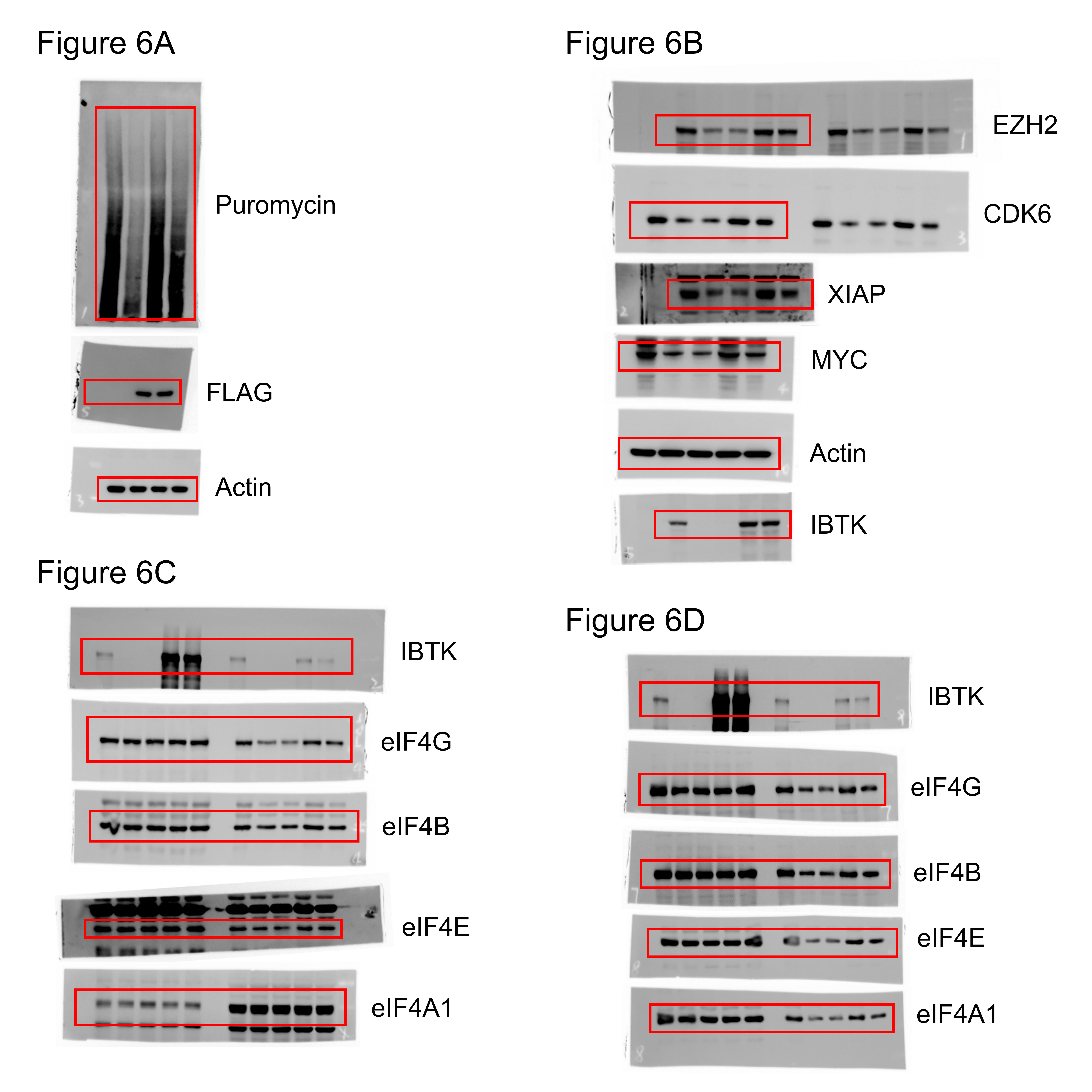

Supplement: Figure 6—source data 2. [file elife-92236-fig6-data2.zip › Figure_6-source_data_2.jpg]
